# scButterfly: a versatile single-cell cross-modality translation method via dual-aligned variational autoencoders

**DOI:** 10.1038/s41467-024-47418-x

**Published:** 2024-04-06

**Authors:** Yichuan Cao, Xiamiao Zhao, Songming Tang, Qun Jiang, Sijie Li, Siyu Li, Shengquan Chen

**Affiliations:** 1 School of Mathematical Sciences and LPMC, Nankai University, Tianjin, 300071 China; 2 MOE Key Laboratory of Bioinformatics and Bioinformatics Division of BNRIST, Department of Automation, Tsinghua University, 100084 Beijing, China; 3 School of Statistics and Data Science, Nankai University, Tianjin, 300071 China

**Keywords:** Computational models, Data integration, Data mining, Machine learning, RNA sequencing

## Abstract

Recent advancements for simultaneously profiling multi-omics modalities within individual cells have enabled the interrogation of cellular heterogeneity and molecular hierarchy. However, technical limitations lead to highly noisy multi-modal data and substantial costs. Although computational methods have been proposed to translate single-cell data across modalities, broad applications of the methods still remain impeded by formidable challenges. Here, we propose scButterfly, a versatile single-cell cross-modality translation method based on dual-aligned variational autoencoders and data augmentation schemes. With comprehensive experiments on multiple datasets, we provide compelling evidence of scButterfly’s superiority over baseline methods in preserving cellular heterogeneity while translating datasets of various contexts and in revealing cell type-specific biological insights. Besides, we demonstrate the extensive applications of scButterfly for integrative multi-omics analysis of single-modality data, data enhancement of poor-quality single-cell multi-omics, and automatic cell type annotation of scATAC-seq data. Moreover, scButterfly can be generalized to unpaired data training, perturbation-response analysis, and consecutive translation.

## Abstract (web summary)

Technical limitations of simultaneously multi-omics profiling lead to highly noisy multi-modal data and substantial costs. Here, authors proposed a versatile framework and data augmentation schemes, capable of single-cell cross-modality translation and multiple extensive applications.

## Introduction

Advances in single-cell sequencing technology have enabled a myriad of single-cell modalities, providing unprecedented opportunities to reveal the previously unknown cell heterogeneity. For example, single-cell RNA sequencing (scRNA-seq) can measure gene expression of individual cells to characterize transcriptional heterogeneity, and single-cell ATAC sequencing (scATAC-seq) can profile chromatin accessibility to capture the chromatin regulatory landscape that governs transcription. However, such single-modality data only capture one measurement and can lose essential information about how different layers of genomic regulation interact within individual cells^1^. To achieve a comprehensive view of single cells, various single-cell multi-omics protocols that can simultaneously profile multiple modalities in the same cell have been proposed^2–5^, promoting fundamental understanding of the molecular hierarchy from genome to phenome^6,7^. Nevertheless, widespread application of joint profiling is still impeded by the more sophisticated techniques, lower sensitivity and throughput, and higher noise and cost than single-modality profiling^1,8–10^. In addition, massive single-modality data accumulated in repositories require further integrative analysis with other modalities^11–13^. Therefore, accurate single-cell cross-modality translation is in pressing need to infer and synthesize paired multi-omics measurements, especially when the single-modality data is economical, reliable or technically feasible, or when the original samples of single-modality data are no longer available, as is often the case for clinical or archival samples^14^.

Several computational methods have been proposed for single-cell cross-modality translation. Typically, these methods embed the collected single-cell multi-omics data into a shared latent space and translate the data between different modalities in a supervised fashion. For example, BABEL uses two autoencoders to embed ATAC and RNA profiles respectively, and infers RNA and ATAC profiles by simultaneously minimizing two reconstruction losses and two cross-prediction losses^1^. Polarbear first trains a variational autoencoder (VAE) for each data modality and then stitches the frozen encoder of one modality with the frozen decoder of another modality via a fully connected layer to implement translation^8^. JAMIE develops joint VAEs for multi-modal imputation and embedding using cross-modal correspondence^15^. UnitedNet is an autoencoder-based method trained by alternating between joint group identification and cross-modal prediction^16^. Besides, Yang et al. proposed a cross-modal autoencoder framework that combines the encoder and decoder of modal-specific autoencoders and is mainly devoted to the translation between single-cell expression and imaging data^9^. CLUE extends from the multi-modal VAEs and uses the cross-encoders to construct latent representations from modality-incomplete observations^17^. For the translation from gene expression to protein expression, sciPENN integrates a sequence of feed-forward blocks with a recurrent neural network and performs end-to-end training^10^.

However, there are still many issues and constraints affecting single-cell cross-modality translation. First, the characteristics of most single-cell data, including high dimensionality and technical variation, make the modeling challenging. Besides, dropout events due to the loss of DNA material during library preparation require tailored denoising approaches for the large number of false zeros^18–20^. Second, an effective data augmentation scheme may further improve the translation performance, alleviate the issues of limited number of cells measured by multi-omics protocols and uncover cell heterogeneity in highly noisy data of complex tissues. Third, there is a prevalent challenging scenario where the single-modality data to be translated has inter-sample variations (e.g., batch effects) and even different biological contexts (e.g., novel cell types) compared to the data used for training. Although many previous methods attempted to address similar problems, this issue has not been satisfactorily resolved (Supplementary Text 1). Fourth, paired multi-modal single-cell profiles may be not available for model training, requiring diagonal strategies to learn a competent translator based on unpaired data^7^. Fifth, extensive applications of cross-modality translators have not been comprehensively and systematically evaluated, such as integrative analysis of single-modality data with its predicted profiles of other modalities, enhancement of single-cell multi-omics data, cell type annotation of challenging modalities such as scATAC-seq data, and consecutive translations among multiple modalities.

To address these challenges, we developed scButterfly, a versatile framework capable of single-cell cross-modality translation and multiple extensive applications. scButterfly first trains a masked VAE for each modality to learn the latent factors within individual modalities, and then dual-aligns the latent representations of different modalities simultaneously at the semantic level to learn cross-modality relationships. Moreover, we introduce a data augmentation scheme to increase training samples, facilitate the characterization of cell-to-cell variation, and enable diagonal training on unpaired data. Our study centers on the translation between chromatin and transcriptome profiles, with further investigation into the complex interplay between transcriptome and proteome profiles. Based on comprehensive experiments on multiple datasets, we demonstrate that scButterfly outperforms baseline methods for cross-modality translation with cell heterogeneity well preserved, and consistently performs well across diverse settings, even when the sequencing protocols vary substantially across datasets, when the multi-omics data for training is unpaired or sparse, or when the single-modality data to be translated was derived from different batches or contains novel cell types. Besides, scButterfly can preserve subtle cell types in the original data and reveal valuable biological insights by cell type-specific enrichment analysis. Furthermore, we demonstrate the extensive potential of scButterfly to enable integrative multi-omics analysis for single-modality data, enhance poor-quality single-cell multi-omics data, annotate cell types for scATAC-seq data automatically, achieve consecutive translations from epigenome to transcriptome to proteome, and open a possible avenue for the prediction of single-cell perturbation responses.

## Results

### Overview of the scButterfly model

**Fig. 1:**
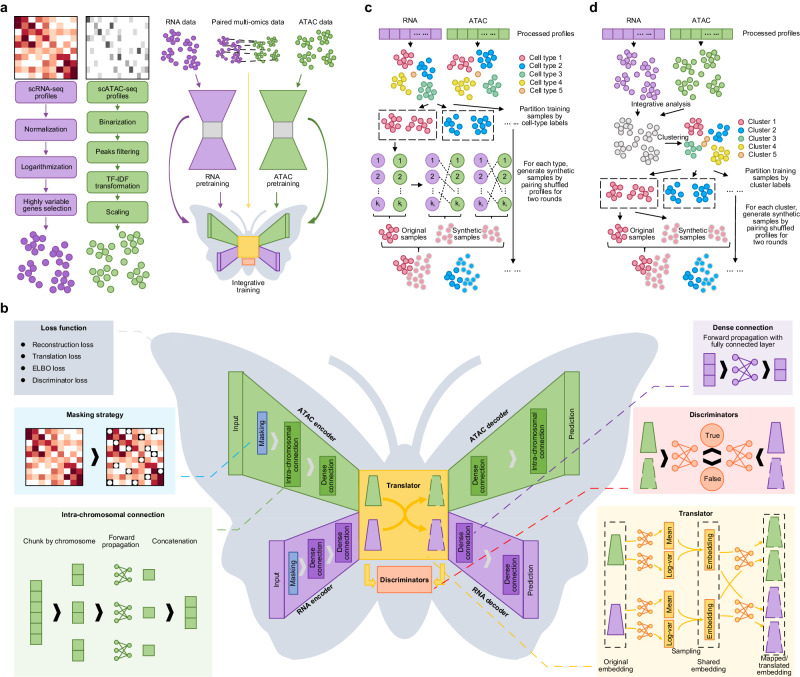
Overview of scButterfly. We take the translation between transcriptome and chromatin profiles as an example for illustration. **a** scButterfly pre-processes the data of each modality using the corresponding customary strategies, pretrains the encoders and decoders in a modality-specific manner, and performs model training with paired multi-modal data based on the pretrained parameters. **b** The basic scButterfly model (scButterfly-B) includes two encoders to project the pre-processed data into modality-specific latent spaces, a translator to translate between different modalities and map within each modality utilizing the multivariate Gaussian distributions in latent space, two modality-specific discriminators to distinguish the latent cell embeddings before and after translation and enable adversarial training, and two decoders to reconstruct the original high-dimensional cell representations of each of the two modalities using the embeddings translated or mapped by the translator. We introduce a masking strategy for the encoders to alleviate the noise influence of dropout events and prune the inter-chromosomal connections for the encoder and decoder of chromatin profiles to alleviate the computational burden and focus on intra-chromosomal biological patterns. **c** The data augmentation strategy of scButterfly for the scenario that cell-type labels of the training set are available. We generate samples by randomly pairing the transcriptome profile of a cell with the chromatin profile of another cell of the same type, resulting the variant regarded as scButterfly-T (Type). **d** The data augmentation strategy of scButterfly for the more general scenario that the training set is devoid of annotations. We perform integrative analysis to cluster the cells in training set and generate samples by randomly pairing according to the cluster labels, resulting the variant regarded as scButterfly-C (Cluster).

scButterfly is a generative adversarial model based on dual-aligned variational autoencoders. Taking the translation between scRNA-seq and scATAC-seq data as an example for illustration, the primary workflow of scButterfly encompasses data pre-processing, parameter pretraining, and model training (Fig. 1a). Regarding each of the different omics, we perform customary data pre-processing strategies accordingly (Methods), pre-train the parameters of encoder and decoder via an omics-specific VAE which has the same hyperparameters as that in scButterfly, and finally train scButterfly with paired multi-omics data based on the pre-trained parameters. As shown in Fig. 1b, the basic scButterfly model (scButterfly-B) consists of seven major modules, including two encoders, two decoders, a translator and two discriminators. Specifically, the encoders project scRNA-seq and scATAC-seq data into modality-specific spaces for cross-modality translation, a different problem from typical multi-omics data integration^7^, while the decoders map the translated latent representations back to the original feature space. We introduce a masking strategy for the encoders to alleviate the noise influence of dropout events and prune the inter-chromosomal connections for the encoder and decoder of scATAC-seq data to substantially reduce parameter space and focus on intra-chromosomal biological patterns (Methods), inspired by the insight that most chromatin accessibility interactions occur at an intra-chromosomal level^21^. The translator functions as a generator that generates the translated representations utilizing the learned multivariate Gaussian distributions in the latent space and feeds the generated results into decoders to fit true profiles via a supervised fashion (Methods). The two discriminators are employed to ensure proper alignment of the inputs and outputs of the translator in an omics-specific manner, competing in an adversarial scheme with the training of the translator (Methods).

To address the issue of limited number of cells with multi-omics for model training and to capture cell heterogeneity in the exceedingly noisy single-cell multi-omics data, we further propose a data augmentation scheme capable of catering to diverse application scenarios. The fundamental concept behind this scheme is that two cells of the same category possess similar biological characteristics, thus enabling the different omics of the two cells to match each other. For the scenario that the training set is equipped with cell-type labels, we generate samples by randomly pairing the transcriptome profile of a cell with the chromatin profile of another cell of the same type (Fig. 1c, Methods), and obtain the variant regarded as scButterfly-T (Type). For the more general scenario that the training set is devoid of annotations, we first perform integrative analysis for the training set and cluster cells in an unsupervised manner^22^, then generate samples by randomly pairing the transcriptome profile of a cell with the chromatin profile of another cell of the same cluster (Fig. 1d, Methods), and obtain the variant regarded as scButterfly-C (Cluster). In addition to performing cross-modality translation with biological implications, scButterfly can also be generalized to facilitate integrative multi-omics analysis, enhance single-cell multi-omics data, annotate cell types for scATAC-seq data, and predict single-cell perturbation responses.

### scButterfly enables cross-modality translation while preserving cell heterogeneity

**Fig. 2:**
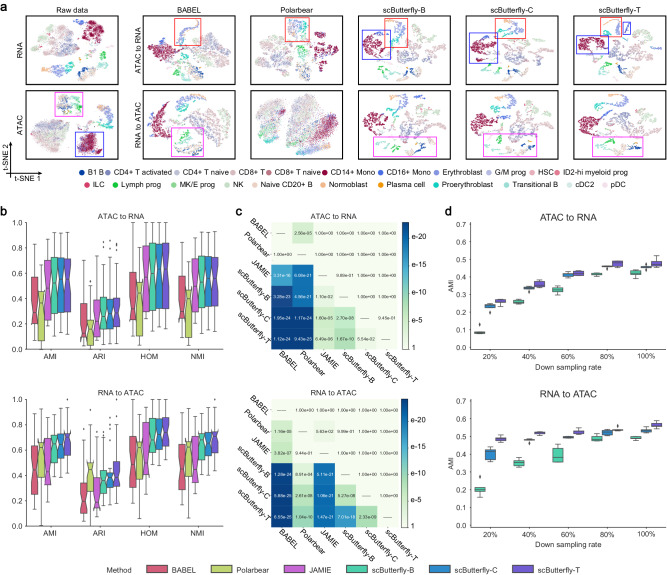
Cross-modality translation performance between epigenome and transcriptome. **a** t-SNE visualization of cells in the first test fold of five-fold cross-validation by cell on the BMMC dataset, using the raw profiles and the profiles translated by different methods. **b** Quantitative evaluation of the translated profiles for preserving cell heterogeneity. We evaluated the cross-validation performance on the seven datasets (BMMC, MB, CL, MCC, MK, MDS, and PBMC) (*n* = 35 cross-validations on seven datasets) via cell clustering with metrics of AMI, ARI, HOM, and NMI. In the boxplots, the center lines, box limits, whiskers and notches denote the median, upper and lower quartiles, 1.5× interquartile range and 95% confidence interval calculated using a Gaussian-based asymptotic approximation, respectively. Note that the boxes of JAMIE only contain 30 data points from six datasets since JAMIE encountered GPU memory errors on the BMMC dataset. **c** Heatmap of *p*-values of one-sided paired Wilcoxon signed-rank tests. Each value in the heatmap indicates the significance of the advantage of a method (row) over another method (column) (*n* = 140 evaluations on seven datasets via four metrics). For the tests associated with JAMIE, we only considered the six datasets where JAMIE performed successfully. **d** Evaluation of the cross-modality translation performance on dataset with different rate of random down sampling for cells, including five-fold cross-validation on the MCC dataset (*n* = 5 cross-validations on 9190 cells), via cell clustering with metrics of AMI, ARI, HOM and NMI. In the boxplots, the center lines, box limits, whiskers and notches denote the median, upper and lower quartiles, 1.5× interquartile range and 95% confidence interval calculated using a Gaussian-based asymptotic approximation, respectively. Source data are provided as a Source Data file.

We first use an extensive paired RNA and ATAC-seq data of bone marrow mononuclear cells (referred as the BMMC dataset) as a proof of concept to demonstrate the efficacy of scButterfly. The BMMC dataset, as a comprehensive multi-modal benchmark dataset, contains over 69,000 cells of 13 samples derived from 4 generation sites and 10 different donors^23^. We conducted five-fold cross-validation experiments by randomly splitting all cells into five folds and iteratively translating the chromatin profiles of cells in each fold to transcriptome profiles, and vice-versa, using the model trained with the remaining four folds. To test if the translated profiles contain biologically interpretable cell heterogeneity, we evaluated the translation performance by various downstream analysis tasks (that is, dimensionality reduction, cell clustering, differential expression and accessibility analysis, etc.)^7^. We compared the performance of scButterfly to BABEL, Polarbear, and JAMIE with default settings (Methods). Taking the first test fold as an example, the RNA profiles translated from ATAC profiles by scButterfly can effectively dissect cellular heterogeneity as shown in the t-Distributed Stochastic Neighbor Embedding (t-SNE) visualization (Fig. 2a). Specifically, all the three variants of scButterfly, including the basic model (scButterfly-B), as well as those based on data augmentation by integrative clustering (scButterfly-C) or cell-type labels (scButterfly-T) in the training set, were capable of distinguishing the stages of erythropoiesis, i.e., proerythroblasts, normoblasts and erythroblasts (marked with red boxes), while BABEL and Polarbear, two state-of-the-art methods for the translation between transcriptome and chromatin profiles, failed to characterize these three important cell types. Note that JAMIE encountered an error on the BMMC dataset due to exceeding the GPU memory limit (48GB, NVIDIA RTX A6000). Besides, we note that all the three variants of scButterfly successfully identified CD14+ Mono and CD16+ Mono (marked with blue boxes), which can be hardly dissected via the raw scATAC-seq data, indicating that scButterfly can fully leverage the information from multiple modalities in the training set while translating the test data from one modality to another. Analogously, the ATAC profiles translated from RNA profiles by scButterfly also preserved cell heterogeneity well (Fig. 2a). For instance, using the ATAC profiles predicted by scButterfly, we can effectively capture the cell types of Transitional B, Lymph prog, Naive CD20 + B, and B1 B (marked with purple boxes), whereas the ATAC profiles predicted by BABEL and even the raw chromatin profiles can hardly distinguish B1 B from Naive CD20 + B, and Polarbear exhibited insufficient translation capacity in this scenario. Collectively, the profiles translated by scButterfly can proficiently characterize cell-to-cell variation and have the potential to mitigate noise present in the original modality to facilitate the identification of cell types.

To quantitatively demonstrate the advantage of scButterfly for cross-modality translation, we further performed cell clustering based on dimensionality reduction results of the translated profiles, and assessed the performance by adjusted mutual information (AMI), adjusted Rand index (ARI), homogeneity score (HOM), and normalized mutual information (NMI) as suggested in refs. ^24–26^ (Methods) for both directions of translation to reveal more biological insights (Supplementary Text 2). The clustering performance on each fold in the cross-validation experiments again indicated that scButterfly significantly outperformed the baseline methods for cross-modality translation while preserving cell heterogeneity (Supplementary Figs. 1, 2). Moreover, we collected six additional datasets with jointly profiled RNA and ATAC data, including the MCC dataset profiled from adult mouse cerebral cortex using SNARE-seq^4^, the MB dataset profiled from adult mouse brain using SHARE-seq^2^, the PBMC dataset of peripheral blood mononuclear cells profiled by 10x-Multiome, the MK dataset profiled from adult mouse kidney using sci-CAR^3^, the CL dataset profiled from multiple cell lines using scCAT-seq^5^, and the MDS dataset profiled from adult mouse dorsal skin using SHARE-seq^2^, to further comprehensively evaluate the performance of scButterfly (Supplementary Fig. 3). As shown in Fig. 2b and Supplementary Fig. 4, scButterfly-B achieved better performance than the baseline methods in both directions of cross-modality translation and efficiently characterize cellular heterogeneity, especially for the translation from transcriptome to epigenome (Supplementary Text 3). One-sided paired Wilcoxon signed-rank tests also demonstrated the significant advantages of scButterfly-B for translation while preserving cell heterogeneity (Fig. 2c). Besides, we noticed that scButterfly-C yielded slightly better performance than scButterfly-B. Although data augmentation with scButterfly-T may bring some additional noise (Supplementary Text 4), it further improved the performance. The results were as expected, since scButterfly-C utilized integrative clustering for data augmentation, while scButterfly-T further expanded upon this by leveraging cell-type labels in the training set for data augmentation, suggesting that we can perform diverse data augmentation strategies to enhance the translation performance according to practical circumstances, namely, whether or not the training set was labeled.

Given that single-cell multi-omics data usually contains a limited number of cells, computational methods need to be capable of learning from a small number of samples. To demonstrate the contribution of data augmentation in addressing this issue, we took the MCC dataset as an example, randomly selected 20–100% of cells from the original data, and compared the performance of three variants of scButterfly. As shown in Fig. 2d and Supplementary Fig. 5, both scButterfly-C and scButterfly-T exhibited improvement compared to scButterfly-B, especially for the scenarios with limited number of cells, indicating the effectiveness of data augmentation strategies in mitigating the limitations of restricted sample size in multi-omics data. Furthermore, we conducted analyses to investigate the impact of the augmented sample counts on the translation performance. scButterfly could achieve the best performance when augmenting the dataset to three times the original dataset size, indicating this choice is suitable and robust (Supplementary Text 5).

In addition, multi-omics data often exhibits varieties in feature dimensions and sequencing techniques, which means that keeping strong robustness to feature selection and hyperparameters is crucial in cross-modality translation. To mimic the protocols with different counts of features, we took the CL dataset as an example, randomly selected 20%, 40%, 60%, 80%, and 100% of the features in the original data for both two omics, and conducted five-fold cross-validation. We compared three variants of scButterfly with other baseline methods. As shown in Supplementary Fig. 6, scButterfly achieved optimal performance under almost all settings of feature counts, underscoring its superior capability in handling multi-omics data at various dimensions. Additionally, scButterfly also demonstrated satisfactory robustness to high-level noise (Supplementary Text 6) and model hyperparameters, including the training epochs, patience of early stop, and the loss weights (Supplementary Text 7). These results imply that scButterfly could consistently achieve outstanding performance under dimensional diversity and technical variation.

Besides, scButterfly also demonstrated promising translation performance in terms of numerical accuracy evaluated by several correlation metrics (Supplementary Text 8). Taken together, these results indicate that scButterfly is applicable for cross-modality translation on datasets generated from different species and protocols, and with various sizes, dimensions, numbers of batches or cell types, proportions of the major type, degrees of cell-type imbalance, and levels of sparsity (Supplementary Fig. 3). Moreover, the inferred profiles can effectively dissect cellular heterogeneity to facilitate downstream cell type identification.

### scButterfly effectively translates data of novel contexts and reveals biological insights

**Fig. 3:**
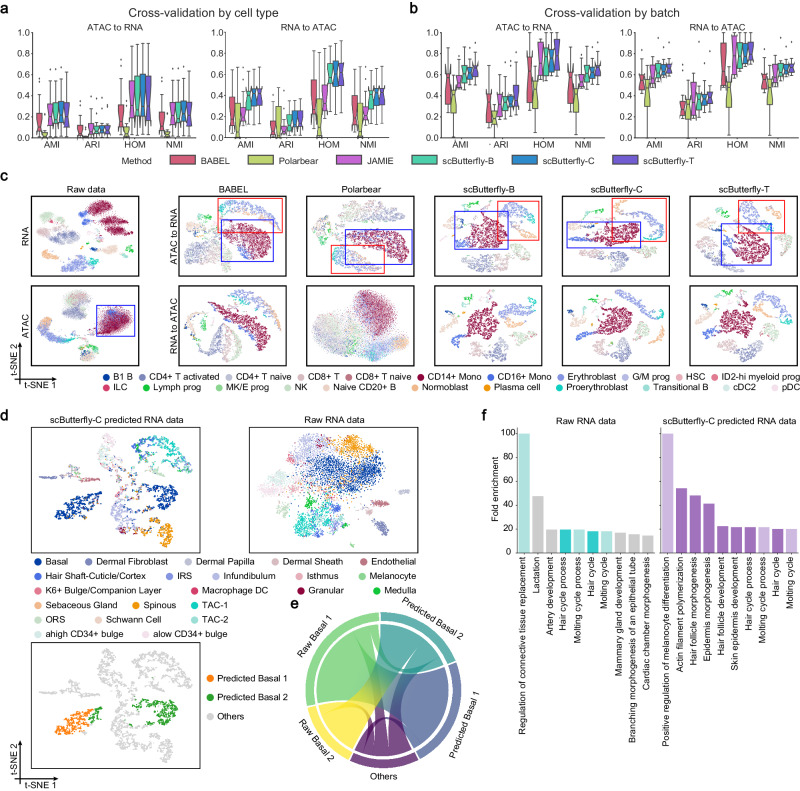
Translation performance for data of novel contexts and biological insights revealed by scButterfly. Quantitative evaluation of the translated profiles for preserving cell heterogeneity in three-fold cross-validation by cell type on the MB, MCC, MK and PBMC datasets (*n* = 12 cross-validations on four datasets) (**a**) and four-fold cross-validation by batch on e BMMC, CL and MDS datasets (*n* = 12 cross-validations on three datasets) (**b**), via cell clustering with metrics of AMI, ARI, HOM and NMI. In the boxplots, the center lines, box limits, whiskers and notches denote the median, upper and lower quartiles, 1.5× interquartile range and 95% confidence interval calculated using a Gaussian-based asymptotic approximation, respectively. For cross-validation by batch, the boxes of JAMIE only contain 8 data points from two datasets since JAMIE encountered GPU memory errors on the BMMC dataset. **c** t-SNE visualization of cells in the first test fold of cross-validation by batch on the BMMC dataset, using the raw profiles and the profiles translated by different methods. **d** t-SNE visualization of cells in the first test fold of cross-validation by batch on the MDS dataset, using the scButterfly-C-predicted (left) and raw RNA profiles (right), as well as illustration of the two subgroups of basal cells identified based on the scButterfly-C-predicted RNA profiles (left-bottom). **e** Chord diagram of the overlapping relationship between the two subgroups of basal cells identified based on the scButterfly-C-predicted RNA profiles and the two subgroups identified based on the raw RNA profiles. **f** Biology process terms enriched by the top 40 differentially expressed genes (DEGs) between the two subgroups identified based on the raw (left) or scButterfly-C-predicted (right) RNA profiles, respectively. The height of each bar denotes the fold enrichment and the color indicates the correlation between the item and basal cells according to the literature (dark colors indicate high correlation, light colors indicate relatively weak correlation, and gray indicates no correlation). Source data are provided as a Source Data file.

Considering that the profiles to be translated may originate from biological contexts different from the training set and contain novel cell types, we further assess the performance of various methods for cross-modality translation of novel cell types by randomly splitting cells into three folds by cell type to implement cross-validation experiments. That is to say, there is no intersection between the cell types in the testing set and the cell types in the training set. We used the four single-batch datasets (MB, MCC, MK, and PBMC) for the assessment. As shown in Fig. 3a, under this challenging out-of-sample translation, the overall performance of all methods has dramatically decreased compared to that of the conventional experiments of cross-validation by cell (Fig. 2b), which was as excepted considering the limitation shared by most machine learning approaches, i.e., test samples deviating too far from the training set often demonstrate poor predictive performance. Even that, for the cross-modality translation of unseed cell types, all the three variants of scButterfly outperformed other methods for characterizing cellular heterogeneity in either inferring RNA expression from ATAC profiles or vice versa (Fig. 3a and Supplementary Figs. 7, 8, 9a), suggesting that scButterfly can effectively recognize the complex relationships between cells in different biological contexts rather than simply memorizing a similar cell seen during training.

Given that technical variations such as inter-sample variations of batch/donor effects can constitute hindrances to cross-modality translation, we next considered a more prevalent challenge that translates profiles of different batches from the similar biological system as the training set. Using three multi-batch datasets (BMMC, CL, and MDS), we performed four-fold cross-validation by randomly grouping cells by batch. To be specific, we performed translation on the data from one batch (the MDS and CL datasets) or site (the BMMC dataset) while training the model on the remaining batches or sites to ensure that the training and test datasets included completely different batches or sites. As shown in Fig. 3b and Supplementary Figs. 9b, 10, 11, scButterfly achieved better performance than baseline methods. Note that JAMIE again encountered a memory error on the BMMC dataset. Moreover, the results again illustrated the overall utility of exploiting data augmentation to aid in predicting cross-modality profiles. Taking the first test fold in the BMMC dataset as an example again, scButterfly can not only preserve subtle cell types in the data to be translated, but also leverage the learned biological relationships between modalities to mitigate the impact of noise and identify the cell types that can be hardly separated via the raw data before translation (Fig. 3c). For instance, using the transcriptome profiles translated from chromatin profiles, all the three variants of scButterfly successfully distinguished the stages of erythropoiesis (marked with red boxes), and identified CD14+ Mono and CD16+ Mono (marked with blue boxes) that can be hardly dissected via the raw scATAC-seq data, while BABEL and Polarbear only separated these cell types moderately, again indicating the advantages of scButterfly in this general cross-modality translation scenario.

Interestingly, we noticed that the RNA profiles predicted by scButterfly-C have the potential to identify cell subtypes and provide functional insights into the identified cell subpopulations. Specifically, on the MDS dataset, we performed four-fold cross-validation by batch and illustrated with the cells in the first test fold as an example, which solely comes from a single batch (Supplementary Fig. 12). The predicted RNA profiles separated the basal cells into two groups, while it was hard to dissect the basal cells on the t-SNE visualization using the raw RNA profiles (Fig. 3d). We then performed Leiden clustering with default resolution^27^ on the predicted RNA profiles and identified two subgroups of basal cells (Predicted Basal 1 and 2) based on the clustering results and the patterns of expression (Supplementary Fig. 13a, c). Using the same approach, we also identified two subgroups of basal cells on the raw RNA profiles (Raw Basal 1 and 2) based on the clustering results (Supplementary Fig. 13b), and 56.850% of cells between these two subgroups and the two of the predicted RNA profiles mapped one-to-one (Fig. 3e). We next used one-sided Wilcoxon rank-sum test in Scanpy^27^ to find the top 40 differentially expressed genes (DEGs) between the two subgroups of the raw or predicted RNA profiles, respectively, and performed gene ontology enrichment^28,29^ using the identified DEGs. As shown in Fig. 3f, the DEGs of the raw data were insufficient in revealing basal cell-associated biological processes, suggesting that the two subgroups identified in the raw RNA data can reveal only limited biological insights. However, based on the DEGs of scButterfly-C, seven out of the top ten significantly enriched biological processes were related to basal cells, especially for morphogenesis, development, and cell cycle. Previous studies indicated that basal cells may contain a subtype named proliferative basal cells (PBC), which usually highly express the genes associated with the positive regulation of the cell cycle^30,31^. In non-proliferative basal cells, *Krt14* is usually highly expressed, while PBC often shows higher expression of *Mki67* and *Top2a*. As shown in Supplementary Fig. 14, in the scButterfly-C translated profiles, *Mki67* and *Top2a* were higher expressed in Predicted Basal 2 compared to Predicted Basal 1, and contrarily, *Krt14* was higher expressed in Predicted Basal 1. This observation indicted that scButterfly holds great potential in the identification of cell subtypes, as well as in providing functional insights into the identified cell subpopulations.

In addition, we noticed that the translated ATAC profiles of scButterfly could be used to identify cell type-specific peaks and reveal transcription factors (TFs) regulatory relationships. Specifically, on the PBMC dataset, we used EpiScanpy^32^ to identify cell type-specific differentially accessible peaks (DAPs) and background peaks in the scButterfly-C translated profiles. We then performed single-nucleotide polymorphisms (SNPs) enrichment analysis using SNPsea^33^ to obtain tissues explicitly affected by identified DAPs. As shown in Supplementary Fig. 15, DAPs associated significantly with their cell types among the three subgroups of CD4 + T cells, three different subtypes of B cells, nature killer (NK) cells, and plasmacytoid dendritic cells (pDC). In contrast, background peaks exhibited less significant enrichment, indicating that scButterfly-C translated ATAC profiles could be used to effectively identify cell type-specific peaks and provide insights into cellular heterogeneity in related tissues. Furthermore, we performed TF regulatory network inference using DeepTFni^34^ on the translated profiles and identified cell type-specific TFs. The scButterfly-C translated profiles accurately enriched *RARγ* and *FOXP3* as specific TFs separately for CD8_Naive^35^ and CD4_Naive^36^, Additionally, *MEF2D* and *SP1* were also identified in the TF-TF adjacency matrix of CD14_Mono, where *MEF2D* has been shown to synergistically interact with *SP1* to activate the *CD14* promoter^37^. These results suggest scButterfly could help reveal cell type-specific transcription factor regulatory relationships and further provide biological insights into the transcription landscape.

### scButterfly facilitates integrative analysis, data enhancement, and cell type annotation

**Fig. 4:**
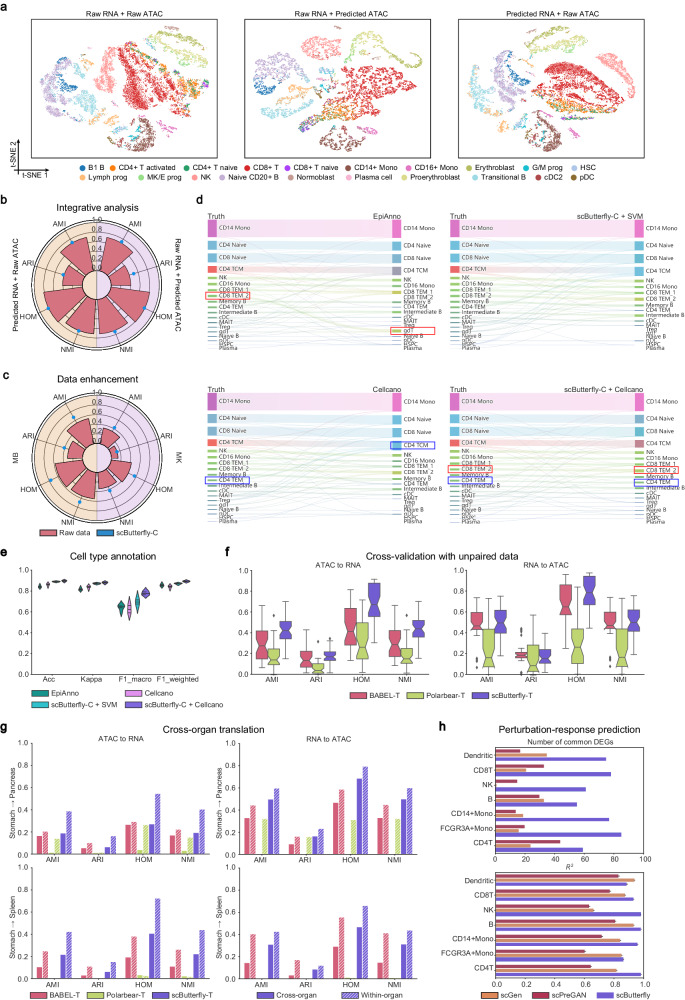
Extensive applications of scButterfly. **a** t-SNE visualization of cells in an independent site of the BMMC dataset, via multi-modal data integration based on the raw data (left), the raw RNA and scButterfly-C-predicted ATAC data (middle), and the scButterfly-C-predicted RNA and raw ATAC data (right). Quantitative evaluation of multi-modal data integration (**b**) and data enhancement (**c**) performance via cell clustering with metrics of AMI, ARI, HOM, and NMI, based on different multi-modal data in **a**, i.e., the complete raw multi-modal data or the multi-modal data with computationally synthesized missing modalities (**b**), and the raw and predicted multi-modal data, on the MK and MB datasets (**c**), respectively. **d** Illustration of the cell type annotation results for scATAC-seq data of the first fold in PBMC dataset by the well-tailored methods and the combinations of scButterfly-C with different classifiers. **e** Cell type annotation performance evaluated by Acc, Kappa, F1-macro, and F1-weighted in five-fold cross-validation experiments for scATAC-seq data of the PBMC dataset. **f** Evaluation of the cross-modality translation performance for unpaired data, including five-fold cross-validation experiments in eight unpaired datasets (*n* = 40 cross-validations on eight datasets), via cell clustering with metrics of AMI, ARI, HOM and NMI. Note that JAMIE encountered GPU memory errors on all the datasets except the UP_HK dataset. In the boxplots, the center lines, box limits, whiskers and notches denote the median, upper and lower quartiles, 1.5× interquartile range and 95% confidence interval calculated using a Gaussian-based asymptotic approximation, respectively. **g** Quantitative evaluation of cross-organ translation performance from the UP_stomach dataset to the UP_pancreas or UP_spleen dataset, as well as the median performance of the within-organ translation for the UP_pancreas or UP_spleen dataset in **f**. **h** Performance of single-cell perturbation-response prediction on the PT_PBMC dataset, evaluated by the number of common DEGs and the squared Pearson correlation (*R*^2^) for mean gene expression of the top 100 real DEGs (Methods). Each bar in the subplot of *R*^2^ contains 100 data points of *R*^2^ values estimated using random subsampling at 80%. The center values and error bars denote the mean and standard deviation, respectively. Source data are provided as a Source Data file.

We next demonstrate the advantage of scButterfly for more extensive applications. Given that the integrative analysis of different modalities is critical for studying cellular heterogeneity from comprehensive perspectives^6,7,22^, we tested the performance of scButterfly for facilitating multi-omics analysis by computationally synthesizing the missing modalities. Taking the BMMC dataset as an example again, we randomly selected three sites for model training and translated the RNA and ATAC profiles, respectively, in the remaining site that contains batches independent of the batches for training. For the remaining site, we used the integration of raw RNA and ATAC profiles as the baseline, and evaluated the integration of raw RNA with predicted ATAC profiles as well as the integration of raw ATAC with predicted RNA profiles. We adopted scButterfly-C for translation to ensure the generality since it does not require additional cell annotations. Based on the widely-used MultiVI method for multi-modal data integration^22^, either the integration of raw RNA with predicted ATAC or the integration of raw ATAC with predicted RNA can effectively dissect cellular heterogeneity and was competitive with the integration of raw RNA and raw ATAC (Fig. 4a). We also quantitatively assessed the performance of scButterfly-C for integrative analysis by cell clustering, and no matter which modality is available, scButterfly-C achieved comparable and even slightly better cell type identification performance than using the raw multi-omics data (Fig. 4b). The results suggest that scButterfly offers valuable insights when only one modality is experimentally available, making it possible to reanalyze the massive single-modality data generated by cell atlas consortiums^11–13^ in an integrative manner.

Considering the additional precautions required by single-cell multi-omics approaches can lead to increased noise and dropout and make data analysis challenging^1^, we further investigate the potential of scButterfly to enhance the existing single-cell multi-omics data. We took the MK and MB datasets as examples, since MK exhibits the highest transcriptome data sparsity and MB exhibits the highest chromatin data sparsity among the collected datasets (Supplementary Fig. 3). For each of the datasets, we used the entire raw data to train scButterfly-C and then used the trained model to predict RNA and ATAC profiles based on the raw data, i.e., we utilized the same dataset for both training and testing to obtain the enhanced data. As shown in Fig. 4c, compared to using the raw data, utilizing the enhanced data can better characterize cell subpopulations and achieve superior cell clustering performance on both of the two datasets. The results indicate that scButterfly can extract reliable insights from highly noisy single-cell multi-omics data, suggesting a new perspective to data enhancement for the emerging area of multi-modal profiling in an unsupervised manner.

Given that the limited guideposts and assay-specific challenges are still substantial hurdles in cell type annotation of scATAC-seq data although several methods have been proposed^38,39^, we delve deeper into the capabilities of scButterfly in aiding automatic annotation of cell types in scATAC-seq data. Following the studies of EpiAnno^38^ and Cellcano^39^, we also took the PBMC dataset as an example and conducted cross-validation by randomly splitting all cells into five folds and iteratively typing the cells in each fold using the model trained with the remaining four folds. To better simulate the general scenario for ATAC data automatic annotation, we split the datasets into three parts: multi-omics profiles without labels, labeled RNA-only profiles and ATAC-only profiles to be annotated. We trained scButterfly-C using the multi-omics training set, translated the ATAC-only profiles in the test set into RNA profiles via the trained scButterfly-C, trained a classifier using the RNA-only training set, and finally annotated the predicted RNA profiles of the test set via the trained classifier. We adopted the support vector machine (SVM) suggested by the benchmark study^40^ as the classifier, and obtained scButterfly-C + SVM. As shown in Fig. 4d, e and Supplementary Figs. 16, 17, our method can accurately annotate cell types, especially subtypes, and achieve higher accuracy, Cohen’s kappa, macro and weighted F1 scores (Methods) compared with state-of-the-art methods. Besides, we noticed Cellcano also uses gene-level summaries as inputs of their proposed classifier, we thus further replaced SVM with the Cellcano classifier and the results demonstrate that the combination of scButterfly with the advanced classifier can further promote the annotation performance (Fig. 4d, e and Supplementary Figs. 16, 17), highlighting the remarkable potential of scButterfly to annotate cell types in challenging modalities such as scATAC-seq data.

### scButterfly can be generalized to unpaired data training and perturbational analysis

The majority of effort has been focused on cross-modality translation with an assumption that the training samples in each modality are sufficient and complete. In prevalent applications, however, this assumption does not always hold and the diagonal analysis of unpaired data, which are not jointly profiled in the same cells, is regarded as a more challenging task than the analysis of paired data^7^. We collected eight unpaired datasets of transcriptome and chromatin, including the UP_HK dataset profiled from adult human kidney^41^, the UP_MPMC dataset profiled from mouse primary motor cortex^42^, and six datasets (UP_eye, UP_muscle, UP_pancreas, UP_spleen, UP_stomach, UP_thymus) profiled from different human fetal organs^43,44^. We performed five-fold cross-validation to test the generality of scButterfly to the diagonal analysis of unpaired data. Specifically, we constructed paired training samples by randomly pairing the RNA profile of a cell with the ATAC profile of another cell of the same type in the training set (Methods). For the multi-batch datasets, we directly paired the profiles from different batches without batch correction (Supplementary Text 9) and obtained models of BABEL-T, Polarbear-T, JAMIE-T, and scButterfly-T by training the methods with the pseudo-paired samples. Note that JAMIE encountered GPU memory errors on all the datasets except the UP_HK dataset. As shown in Fig. 4f and Supplementary Figs. 18, 19, regardless of whether based on the predicted transcriptome profiles or the predicted chromatin profiles, scButterfly achieved a significantly higher cell population identification accuracy than other methods, indicating the advantages of scButterfly on unpaired data even other methods also use the same training strategy.

We next test if scButterfly can be generalized to cross-organ translation. We adopted the entire unpaired UP_stomach dataset as the training set, used the same training strategy as above, and assessed the performance on the entire UP_pancreas and UP_spleen datasets, respectively. As shown in Fig. 4g, the performance of cross-organ translation exhibits a pronounced disadvantage in comparison to the median performance of the above within-organ five-fold cross-validation (the striped bars), which can be attributed to the notable variations in the biological context across different organs^43,44^. Notwithstanding, we note that scButterfly again consistently outperformed the baseline methods in the profoundly demanding task, further underscoring the potential and generality of scButterfly for cross-organ translation.

We further study the potential of scButterfly for single-cell perturbation-response prediction. Single-cell perturbation-response screens enable the exploration of molecular and phenotypic responses to different perturbations, elucidating the fundamental mechanisms governing biological processes^45^. Nonetheless, acquiring perturbed cells often poses a significant challenge in numerous scenarios^46^. Generative modeling of perturbation response can therefore expand the capabilities of in silico experimentation. As cells are commonly destroyed during the measurement process, it leads to the generation of unpaired distributions encompassing perturbed and non-perturbed cells and the task of single-cell perturbation-response prediction thus requires unpaired data training. We used the PT_PBMC dataset that has been used by both scGen^45^ and scPreGAN^46^, two state-of-the-art methods, as a proof of concept to demonstrate the effectiveness of scButterfly for perturbational analysis. The PT_PBMC dataset includes seven cell types of control and interferon-beta-stimulated human peripheral blood mononuclear cells^47^. We regarded the transcriptome profiles of control and stimulated cells as two modalities, and proposed a strategy based on optimal transport^48^ to match the two groups of cells for generating paired training samples since these two modalities typically exhibit substantial biological differences, which are absent in the aforementioned unpaired single-cell multi-omics data. To be specific, we used optimal transport to obtain the optimal coupling matrix by minimizing the Wasserstein distance between the control and stimulated cells for each cell type, selected the stimulated cell with the largest weight for each control cell, and finally matched these two cells as a paired training sample (Methods). Following the existing studies^45,46^, we evaluated the performance for the challenging out-of-sample prediction that uses the data of a cell type for testing and uses the data of remaining cell types for training, similar to the aforementioned cross-modality translation of novel cell types. We adopted the two metrics universally used in existing studies to assess the performance (Methods)^45,46^. As shown in Fig. 4h, the number of common DEGs of the top 100 (real) DEGs between the control data and the real stimulated data versus the top 100 (predicted) DEGs between the control data and the stimulated data predicted by scButterfly exceeded significantly that of the state-of-art methods. Moreover, we randomly sampled 80% of the test data with replacement 100 times^45^, and computed the squared Pearson correlation (*R*^2^) for mean gene expression of the top 100 (real) DEGs between predicted and real stimulated data. The results illustrated that the transcriptome profiles predicted by scButterfly-B correlated well with the ground truth across different cell types (the mean *R*^2^ of each cell type consistently surpassed 0.85) and scButterfly-B achieved the overall best performance (Fig. 4h), elucidating the promising potential of scButterfly in single-cell perturbational investigations.

### scButterfly enables consecutive translation from epigenome to transcriptome to proteome

**Fig. 5:**
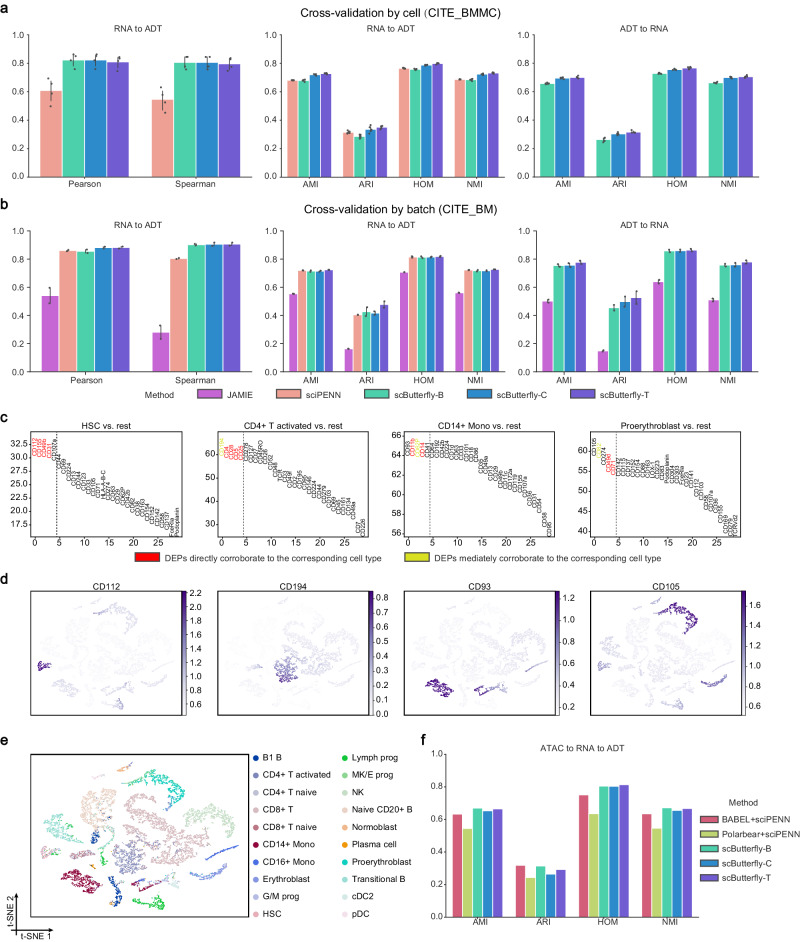
Consecutive translation from epigenome to transcriptome to proteome. Quantitative evaluation of the translation performance between transcriptome and proteome profiles in five-fold cross-validation by cell on the CITE_BMMC dataset (*n* = 5 cross-validations on 90261 cells) (**a**) and two-fold cross-validation by batch on the CITE_BM dataset (*n* = 2 cross-validations on 30672 cells) (**b**), via cell clustering with metrics of AMI, ARI, HOM and NMI. The height of each bar denotes the median value of each metric and the error bars show 95% confidence interval. Note that JAMIE encountered GPU memory errors on the CITE_BMMC dataset. **c** Differentially expressed proteins (DEPs) of different cell types in the proteome profiles that were consecutively translated from epigenome to transcriptome and then to proteome based on the BMMC and CITE_BMMC datasets. The DEPs were obtained via one-sided Wilcoxon rank-sum tests. **d** t-SNE visualization of cells in the first test fold (with batches independent with the batches for training) of BMMC dataset based on the proteome profiles consecutively translated by scButterfly-C. The scButterfly-C-predicted expression levels of the top DEP of each cell type are projected onto the t-SNE visualization. **e** t-SNE visualization (colored by cell type) of cells in the first test fold of BMMC dataset based on the proteome profiles consecutively translated by scButterfly-C. **f** Quantitative evaluation of the consecutive translation performance from epigenome to transcriptome to proteome, via cell clustering with metrics of AMI, ARI, HOM and NMI. Note that JAMIE failed to perform consecutive translation due to GPU memory errors. Source data are provided as a Source Data file.

In addition to epigenome and transcriptome, proteome provides valuable insights into various aspects of cellular function and regulation, such as protein interactions and cellular signaling^49^. Although the advanced cellular indexing of transcriptomes and epitopes by sequencing (CITE-seq) allows simultaneous profiling of RNA gene expression along with cell surface proteins^23^, it remains expensive to generate such data, and methods like sciPENN have thus been proposed to make protein predictions for scRNA-seq data^10^. Besides, the background signal, acting as a major component of noise in proteome profiles^50^, poses a challenge different from the translation between transcriptome and epigenome (Supplementary Text 10). scButterfly, as a flexible and extensive framework, can be generalized to translate between transcriptome and proteome (Methods). We collected a CITE_BMMC dataset that consists of 90,261 human bone marrow mononuclear cells with joint profiles of gene expression and 134 surface proteins using antibody-derived tags (ADT)^23^ and a CITE_BM dataset that consists of 30,672 scRNA-seq profiles measured alongside a panel of 25 antibodies from human bone marrow^51^. We again conducted five-fold cross-validation experiments on each of the datasets, and evaluated the translation performance by cell clustering accuracy and by numerical accuracy of ADT data based on Pearson and Spearman correlation coefficients due to the low dimensionality and dense nature of ADT data. As shown in Fig. 5a and Supplementary Fig. 20a, all the three variants of scButterfly achieved comparable and slightly better performance than the state-of-the-art sciPENN method and significantly outperformed JAMIE. As shown in Supplementary Fig. 21, the three variants of scButterfly accurately preserved the cell type-specific patterns in the original ADT data. Besides, scButterfly also enabled the translation from proteome to transcriptome and yielded satisfactory performance. We also considered the more prevalent challenge that translating profiles from different batches, and performed cross-validation by randomly splitting the cells by batch (Methods). As shown in Fig. 5b and Supplementary Fig. 20b, all the three variants of scButterfly again demonstrated comparable performance and the general scButterfly-C method, which augments data by unsupervised cell clustering, showcased slight improvements over sciPENN and obvious improvements over JAMIE, underscoring the versatility of scButterfly in facilitating the translation between transcriptome and proteome.

We next investigate the capacity of scButterfly in consecutive translation from epigenome to transcriptome to proteome. Taking the BMMC and CITE_BMMC datasets as an example, we randomly split cells in the BMMC dataset into four folds by batch, trained translators from epigenome to transcriptome using the last three folds, trained translators from transcriptome to proteome using the entire CITE_BMMC dataset, and finally translated the chromatin profiles of the first test fold in the BMMC dataset to RNA and then to ADT via the trained translators. Based on the ADT profiles predicted by the general scButterfly-C method, we conducted one-sided Wilcoxon rank-sum tests to find differentially expressed proteins (DEPs) of each type^27^. As shown in Fig. 5c and Supplementary Fig. 22, most of the top five DEPs have good agreement with previous studies. On average, 2.4 and 1.05 out of the top five DEPs can be directly (marked as red) and mediately (marked as yellow) corroborated by the literature. For instance, CD112 has been confirmed to play a crucial role in inducing epigenetic changes and defining an alternative state of human long term hematopoietic stem cell (LT-HSC)^52^, while CD194 (CCR4) is discovered to express in the CD45RA^−^CD4^+^ memory or activated T cells^53^. The expression of DEPs also showed cell type-specific and compact patterns on t-SNE visualizations (Fig. 5d, e and Supplementary Figs. 23, 24), suggesting that scButterfly can effectively recover expression trends of specific protein biomarkers. Moreover, the top DEPs that currently lack literature support also exhibited pronounced cell type-specific expression patterns, such as CD93 of CD14^+^ monocyte and CD105 of proerythroblast (Fig. 5c–e), indicating the potential of scButterfly to decipher unexplored markers. Due to the absence of a direct approach for accomplishing the consecutive translation from epigenome to transcriptome to proteome, we quantitatively compared the performance of scButterfly against a combination of state-of-the-art methods of the two translation tasks, namely BABEL + sciPENN and Polarbear + sciPENN. As shown in Fig. 5e, f, the ADT profiles predicted by scButterfly gave a clear elucidation of nuanced differences among cells and provided overall superior cell clustering performance than baselines methods (ARI scores may be biased given the imbalanced nature of cell types^25,54^). Taken together, the consecutive translation capability of scButterfly not only empowers the translation between two modalities that are inherently difficult to profile simultaneously, but also offers remarkable biological implications.

## Discussion

scButterfly is a versatile single-cell cross-modality translation method based on dual-aligned variational autoencoders and data augmentation schemes. Through comprehensive experiments on multiple datasets generated with different protocols, and of divergent sizes, dimensions and qualities, we validated the superior performance of scButterfly over baseline methods in preserving cell heterogeneity during cross-modality translation, and demonstrated the advantages of scButterfly for translating datasets with novel cell types or inter-sample variations while revealing biological insights. Besides, we showed the extensive applications of scButterfly for integrative multi-omics analysis of single-modality data, data enhancement of poor-quality single-cell multi-omics, and automatic cell type annotation of scATAC-seq data. Additionally, our innovative data augmentation and optimal transport strategies further enable diagonal model training on unpaired multi-omics data and facilitate the analysis of single-cell perturbation responses, respectively. Moreover, we demonstrated the capacity of scButterfly in consecutive translation from epigenome to transcriptome to proteome and the potential of scButterfly to decipher cell type-specific biomarkers. We envision our proposed method will facilitate more comprehensive and cost-effective single-cell multi-modalities analysis.

Certainly, improvements may be explored in the future. We provide several directions for further improving scButterfly. Firstly, we can incorporate the cell heterogeneity information derived from public bulk omics data and/or annotated cell atlas to facilitate the characterization of cell-to-cell variation. Besides, we can also incorporate prior knowledge of gene regulatory mechanisms to bridge different modalities. Secondly, given the extensive applications of scButterfly, we can extend it to flexibly accommodate additional modalities, such as spatial transcriptomics^55^ and single-cell Hi-C data^56^, since more single-cell modalities become available and scButterfly is designed as a generalizable framework for quick adoption to particular scenarios in a modular manner. Thirdly, advanced machine learning techniques with adaptive and interpretable model parameters can be considered to explore novel avenues for boosting the performance of scButterfly, especially for the cross-cell-type and cross-organ translation.

## Methods

### The basic model of scButterfly

The basic model of scButterfly (scButterfly-B) is based on a dual-aligned variational autoencoder framework. Taking the translation between scRNA-seq and scATAC-seq data as an example, scButterfly-B consists of seven primary components (Fig. 1b): RNA encoder and ATAC encoder networks denoted as En_r_ and En_a_, RNA decoder and ATAC decoder networks denoted as De_r_ and De_a_, a translator T and two discriminators Dis_r_ and Dis_a_. Given pre-processed paired training data of scRNA-seq ${{{{{{\bf{X}}}}}}}_{{{{{{\rm{r}}}}}}}\in {{\mathbb{R}}}^{n\times {I}_{{{{{{\rm{r}}}}}}}}$ and scATAC-seq ${{{{{{\bf{X}}}}}}}_{{{{{{\rm{a}}}}}}}\in {{\mathbb{R}}}^{n\times {I}_{{{{{{\rm{a}}}}}}}}$, where *n* denotes the number of cells and *I*_r_/*I*_a_ denotes the feature dimensions of RNA/ATAC data, scButterfly-B translates the input **X**_r_ to chromatin profiles by ${{{{{\rm{D}}}}}}{{{{{{\rm{e}}}}}}}_{{{{{{\rm{a}}}}}}}\left({{{{{{\rm{T}}}}}}}_{{{{{{\rm{r}}}}}}\to {{{{{\rm{a}}}}}}}\left({{{{{\rm{E}}}}}}{{{{{{\rm{n}}}}}}}_{{{{{{\rm{r}}}}}}}\left({{{{{{\bf{X}}}}}}}_{{{{{{\rm{r}}}}}}}\right)\right)\right)$, and translates the input **X**_a_ to transcriptome profiles by ${{{{{\rm{D}}}}}}{{{{{{\rm{e}}}}}}}_{{{{{{\rm{r}}}}}}}\left({{{{{{\rm{T}}}}}}}_{{{{{{\rm{a}}}}}}\to {{{{{\rm{r}}}}}}}\left({{{{{\rm{E}}}}}}{{{{{{\rm{n}}}}}}}_{{{{{{\rm{a}}}}}}}\left({{{{{{\bf{X}}}}}}}_{{{{{{\rm{a}}}}}}}\right)\right)\right)$. Details of each component in scButterfly-B is as follows.

Encoders of En_r_ and En_a_ are responsible for embedding RNA and ATAC inputs into low-dimensional representations, respectively. In En_r_, we utilize fully connected layers with the LeakyReLU activation function to map the input gene expression vector ${{{{{{\bf{X}}}}}}}_{{{{{{\rm{r}}}}}}}^{k}$ of the *k*-th cell $\left(k\le n\right)$ to 256 dimensions and subsequently to 128 dimensions. The activation function is defined as ${{{{{\rm{LeakyReLU}}}}}}\left(x\right)=x{I}_{x\ge 0}+{ax}{I}_{x < 0}$, where *a* is set to 0.01 to address the vanishing gradient problem. In En_a_, inspired by the insight that most chromatin accessibility interactions occur at an intra-chromosomal level^21^ and to alleviate the computational burden, we prune the inter-chromosomal connections and focus on intra-chromosomal biological patterns by mapping the profiles of each chromosome in the input ${{{{{{\bf{X}}}}}}}_{{{{{{\rm{a}}}}}}}^{k}$ to a 32-dimensional space and then projecting the concatenation results onto a 128-dimensional latent space using fully connected layers with LeakyReLU. Both En_r_ and En_a_ use a drop-out mechanism with a probability of 0.1 for all latent layers. Additionally, we adopt a masking strategy for the input data by randomly setting 50% of the elements in **X**_r_ and 30% of the elements in **X**_a_ to zero, inspired by the advanced masked autoencoders (MAE)^57^.

The translator T in latent space facilitates the translation between different modalities and the end-to-end mapping within each of the individual modalities (Fig. 1b). We first map the input En_r_(**X**_r_) from the RNA encoder into 128-dimensional mean vector ${{{{{{\bf{X}}}}}}}_{{{{{{\rm{mean}}}}}}}^{{{{{{\rm{r}}}}}}}$ and log-variance vector ${{{{{{\bf{X}}}}}}}_{{{{{\mathrm{var}}}}}}^{{{{{{\rm{r}}}}}}}$ with two blocks of fully connected layer and LeakyReLU, respectively. Then, based on the assumption of variational autoencoders, the translator T obtains the latent embedding by sampling from the multivariate Gaussian distribution with ${{{{{{\bf{X}}}}}}}_{{{{{{\rm{mean}}}}}}}^{{{{{{\rm{r}}}}}}}$ and ${{{{{{\bf{X}}}}}}}_{{{{{\mathrm{var}}}}}}^{{{{{{\rm{r}}}}}}}$ as follows:

1 $${{{{{{\bf{X}}}}}}}_{{{{{{\rm{embed}}}}}}}={{{{{{\bf{X}}}}}}}_{{{{{{\rm{mean}}}}}}}^{{{{{{\rm{r}}}}}}}+{e}^{\frac{{{{{{{\bf{X}}}}}}}_{{{{{\mathrm{var}}}}}}^{{{{{{\rm{r}}}}}}}}{2}}\times {{{{{\bf{eps}}}}}}{{{{{\mathscr{ \sim }}}}}}{{{{{\mathscr{N}}}}}}\left({{{{{\bf{0}}}}}},{{{{{\bf{I}}}}}}\right).$$

Ultimately, 128-dimensional ${{{{{{\rm{T}}}}}}}_{{{{{{\rm{r}}}}}}\to {{{{{\rm{a}}}}}}}\left({{{{{\rm{E}}}}}}{{{{{{\rm{n}}}}}}}_{{{{{{\rm{r}}}}}}}\left({{{{{{\bf{X}}}}}}}_{{{{{{\rm{r}}}}}}}\right)\right)$ and ${{{{{{\rm{T}}}}}}}_{{{{{{\rm{r}}}}}}\to {{{{{\rm{r}}}}}}}\left({{{{{\rm{E}}}}}}{{{{{{\rm{n}}}}}}}_{{{{{{\rm{r}}}}}}}\left({{{{{{\bf{X}}}}}}}_{{{{{{\rm{r}}}}}}}\right)\right)$, namely the translated ATAC embeddings and the mapped RNA embeddings, are generated from **X**_embed_ with two blocks of fully connected layer and LeakyReLU, respectively. These translated/mapped embeddings are treated as the input for decoders. Analogously, we use another two blocks to map the input En_a_(**X**_a_) from the ATAC encoder into 128-dimensional mean vector ${{{{{{\bf{X}}}}}}}_{{{{{{\rm{mean}}}}}}}^{{{{{{\rm{a}}}}}}}$ and log-variance vector ${{{{{{\bf{X}}}}}}}_{{{{{\mathrm{var}}}}}}^{{{{{{\rm{a}}}}}}}$, respectively, and generate the 128-dimensional translated RNA embeddings ${{{{{{\rm{T}}}}}}}_{{{{{{\rm{a}}}}}}\to {{{{{\rm{r}}}}}}}\left({{{{{\rm{E}}}}}}{{{{{{\rm{n}}}}}}}_{{{{{{\rm{a}}}}}}}\left({{{{{{\bf{X}}}}}}}_{{{{{{\rm{a}}}}}}}\right)\right)$ and mapped ATAC embeddings ${{{{{{\rm{T}}}}}}}_{{{{{{\rm{a}}}}}}\to {{{{{\rm{a}}}}}}}\left({{{{{\rm{E}}}}}}{{{{{{\rm{n}}}}}}}_{{{{{{\rm{a}}}}}}}\left({{{{{{\bf{X}}}}}}}_{{{{{{\rm{a}}}}}}}\right)\right)$ using the same blocks of RNA-to-RNA mapping and RNA-to-ATAC translation, respectively (Fig. 1b).

Discriminators of Dis_r_ and Dis_a_ aim to distinguish between the original embeddings and the translated embeddings, enabling adversarial training to improve the similarity between the translated embeddings and the original embeddings^58,59^. Demonstrating with the discriminator for RNA embeddings as a specific instance, Dis_r_ accepts original RNA embeddings ${{{{{\rm{E}}}}}}{{{{{{\rm{n}}}}}}}_{{{{{{\rm{r}}}}}}}({{{{{{\bf{X}}}}}}}_{{{{{{\rm{r}}}}}}})$ and translated embeddings ${{{{{{\rm{T}}}}}}}_{{{{{{\rm{a}}}}}}\to {{{{{\rm{r}}}}}}}\left({{{{{\rm{E}}}}}}{{{{{{\rm{n}}}}}}}_{{{{{{\rm{a}}}}}}}\left({{{{{{\bf{X}}}}}}}_{{{{{{\rm{a}}}}}}}\right)\right)$ with equal probability during the scButterfly-B training, expected to provide judgement based on the following equation:

2 $$\left\{\begin{array}{c}{{{{{\rm{Di}}}}}}{{{{{{\rm{s}}}}}}}_{{{{{{\rm{r}}}}}}}\left({{{{{{\rm{T}}}}}}}_{{{{{{\rm{a}}}}}}\to {{{{{\rm{r}}}}}}}\left({{{{{\rm{E}}}}}}{{{{{{\rm{n}}}}}}}_{{{{{{\rm{a}}}}}}}\left({{{{{{\bf{X}}}}}}}_{{{{{{\rm{a}}}}}}}\right)\right)\right)=0\\ {{{{{\rm{Di}}}}}}{{{{{{\rm{s}}}}}}}_{{{{{{\rm{r}}}}}}}\left({{{{{\rm{E}}}}}}{{{{{{\rm{n}}}}}}}_{{{{{{\rm{r}}}}}}}\left({{{{{{\bf{X}}}}}}}_{{{{{{\rm{r}}}}}}}\right)\right)=1\end{array}.\right.$$

Similarly, the discriminator Dis_a_ for ATAC embeddings is designed to differentiate ${{{{{\rm{E}}}}}}{{{{{{\rm{n}}}}}}}_{{{{{{\rm{a}}}}}}}\left({{{{{{\bf{X}}}}}}}_{{{{{{\rm{a}}}}}}}\right)$ and ${{{{{{\rm{T}}}}}}}_{{{{{{\rm{r}}}}}}\to {{{{{\rm{a}}}}}}}\left({{{{{\rm{E}}}}}}{{{{{{\rm{n}}}}}}}_{{{{{{\rm{r}}}}}}}\left({{{{{{\bf{X}}}}}}}_{{{{{{\rm{r}}}}}}}\right)\right)$. For the network structures of Dis_r_ and Dis_a_, we separately employ a two-layer network: a 128-dimensional latent block of fully connected layer with LeakyReLU and an output Sigmoid layer (Fig. 1b).

Decoders of De_r_ and De_a_ are responsible for reconstructing the original high-dimensional representations of transcriptome and chromatin profiles, respectively, based on the mapped and translated embeddings in latent space (Fig. 1b). De_r_ follows an inverse process of En_r_ by decoding the embeddings from 128 dimensions to 256 dimensions and to the original space of transcriptome profiles with fully connected layers and LeakyReLU. Similarly, De_a_ inverts the process of En_a_ but adopts a Sigmoid activation in the output layer given the near-binary nature of chromatin profiles. We also adopt a drop-out mechanism with a probability of 0.1 for all the latent layers of De_r_ and De_a_.

### The training procedure of scButterfly

Based on the architecture of the scButterfly-B model, we propose the use of a step-wise training strategy, consisting of a pretraining phase and an integrative training phase (Fig. 1a). Using the translation between transcriptome and chromatin profiles as an example again, in the pretraining phase, we first independently train the RNA encoder En_r_ with its corresponding decoder De_r_, as well as the ATAC encoder En_a_ with its decoder De_a_. During the integrative training phase, we then initialize the parameters of the encoders and decoders with the pre-trained parameters, and train the scButterfly-B model using both modalities simultaneously to capture interdependencies between modalities. The detailed process is as follows.

For the pretraining phase, we mainly focus on the reconstruction loss and evidence lower bound (ELBO) loss for variational inference. Translators are incorporated just for the end-to-end mapping within each of the individual modalities to maintain consistency with the integrative training phase. In each iteration, processed RNA profiles **X**_r_ is subsequently forward propagation through encoder En_r_, translator T_r→r_ and decoder De_r_, resulting the reconstructed ${{{{{{\bf{X}}}}}}}_{{{{{{\rm{r}}}}}}}^{{{{{{\rm{pred}}}}}}}={{{{{\rm{D}}}}}}{{{{{{\rm{e}}}}}}}_{{{{{{\rm{r}}}}}}}\left({{{{{{\rm{T}}}}}}}_{{{{{{\rm{r}}}}}}\to {{{{{\rm{r}}}}}}}\left({{{{{\rm{E}}}}}}{{{{{{\rm{n}}}}}}}_{{{{{{\rm{r}}}}}}}\left({{{{{{\bf{X}}}}}}}_{{{{{{\rm{r}}}}}}}\right)\right)\right)$. We use the mean square error (MSE) loss as reconstruction loss, which could be calculated as:

3 $${{{{{{\rm{L}}}}}}}_{{{{{{\rm{r}}}}}}}\left({{{{{{\bf{X}}}}}}}_{{{{{{\rm{r}}}}}}}^{{{{{{\rm{pred}}}}}}},{{{{{{\bf{X}}}}}}}_{{{{{{\rm{r}}}}}}}\right)={{{{{\rm{MSE}}}}}}\left({{{{{{\bf{X}}}}}}}_{{{{{{\rm{r}}}}}}}^{{{{{{\rm{pred}}}}}}},{{{{{{\bf{X}}}}}}}_{{{{{{\rm{r}}}}}}}\right)=\frac{1}{n}\mathop{\sum }\limits_{k=1}^{n}{{{{{\rm{|}}}}}}{{{{{{\bf{X}}}}}}}_{{{{{{\rm{r}}}}}}}^{{{{{{\rm{pred}}}}}}}[k]-{{{{{{\bf{X}}}}}}}_{{{{{{\rm{r}}}}}}}{\left[k\right]{{{{{\rm{|}}}}}}}^{2}.$$

The ELBO loss could be described as the Kullback-Leibler divergence between the shared embedding ${{{{{{\bf{X}}}}}}}_{{{{{{\rm{embed}}}}}}}^{{{{{{\rm{r}}}}}}\_{{{{{\rm{pretrain}}}}}}}$ of T_r→r_ and normal multivariate Gaussian distribution:

4 $${{{{{{\rm{L}}}}}}}_{{{{{{\rm{ELBO}}}}}}}\left({{{{{{\bf{X}}}}}}}_{{{{{{\rm{embed}}}}}}}^{{{{{{\rm{r}}}}}}\_{{{{{\rm{pretrain}}}}}}}\right)=	{{{{{\rm{KL}}}}}}({{{{{{\bf{X}}}}}}}_{{embed}}^{{{{{{\rm{r}}}}}}\_{{{{{\rm{pretrain}}}}}}}\big|\big|{{{{{\mathcal{N}}}}}}\left({{{{{\bf{0}}}}}},\, {{{{{\bf{I}}}}}}\right))\\=	-\frac{1}{2}(1+{{{{{{\bf{X}}}}}}}_{{{{{\mathrm{var}}}}}}^{{{{{{\rm{r}}}}}}\_{{{{{\rm{pretrain}}}}}}}-{({{{{{{\bf{X}}}}}}}_{{{{{{\rm{mean}}}}}}}^{{{{{{\rm{r}}}}}}\_{{{{{\rm{pretrain}}}}}}})}^{2}-{e}^{{{{{{{\bf{X}}}}}}}_{{{{{\mathrm{var}}}}}}^{{{{{{\rm{r}}}}}}\_{{{{{\rm{pretrain}}}}}}}}),$$

where ${{{{{{\bf{X}}}}}}}_{{{{{{\rm{mean}}}}}}}^{{{{{{\rm{r}}}}}}\_{{{{{\rm{pretrain}}}}}}}$ and ${{{{{{\bf{X}}}}}}}_{{{{{\mathrm{var}}}}}}^{{{{{{\rm{r}}}}}}\_{{{{{\rm{pretrain}}}}}}}$ denote the mean and log-variance vectors obtained from T_r_, respectively. We train En_r_, T_r→r_ and De_r_ with a combination of the two parts of loss as follows:

5 $${{{{{{\rm{L}}}}}}}_{{{{{{\rm{r}}}}}}}^{{{{{{\rm{pretrain}}}}}}}={w}_{{{{{{\rm{r}}}}}}}{{{{{{\rm{L}}}}}}}_{{{{{{\rm{r}}}}}}}\left({{{{{{\bf{X}}}}}}}_{{{{{{\rm{r}}}}}}}^{{{{{{\rm{pred}}}}}}},{{{{{{\bf{X}}}}}}}_{{{{{{\rm{r}}}}}}}\right)+{w}_{{{{{{\rm{ELBO}}}}}}}{{{{{{\rm{L}}}}}}}_{{{{{{\rm{ELBO}}}}}}}\left({{{{{{\bf{X}}}}}}}_{{{{{{\rm{embed}}}}}}}^{{{{{{\rm{r}}}}}}\_{{{{{\rm{pretrain}}}}}}}\right).$$

We set w_r_ = 1 and ${w}_{{{{{{\rm{ELBO}}}}}}}=\frac{20}{{I}_{{{{{{\rm{r}}}}}}}}$, and train the networks for 100 epochs. We apply the Adam optimizer^60^ with a learning rate of 0.001 and early-stop with patience of 50 epochs. The strategy for ATAC pretraining is nearly identical to RNA pretraining, with the only difference being the use of binary cross-entropy (BCE) reconstruction loss, denoted as L_a_, instead of L_r_. With the reconstructed output ${{{{{{\bf{X}}}}}}}_{{{{{{\rm{a}}}}}}}^{{{{{{\rm{pred}}}}}}}={{{{{\rm{D}}}}}}{{{{{{\rm{e}}}}}}}_{{{{{{\rm{a}}}}}}}\left({{{{{{\rm{T}}}}}}}_{{{{{{\rm{a}}}}}}\to {{{{{\rm{a}}}}}}}\left({{{{{\rm{E}}}}}}{{{{{{\rm{n}}}}}}}_{{{{{{\rm{a}}}}}}}\left({{{{{{\bf{X}}}}}}}_{{{{{{\rm{a}}}}}}}\right)\right)\right)$, L_a_ can be calculated by:

6 $${{{{{{\rm{L}}}}}}}_{{{{{{\rm{a}}}}}}}\left({{{{{{\bf{X}}}}}}}_{{{{{{\rm{a}}}}}}}^{{{{{{\rm{pred}}}}}}},{{{{{{\bf{X}}}}}}}_{{{{{{\rm{a}}}}}}}\right)=	{{{{{\rm{BCE}}}}}}\left({{{{{{\bf{X}}}}}}}_{{{{{{\rm{a}}}}}}}^{{{{{{\rm{pred}}}}}}},{{{{{{\bf{X}}}}}}}_{{{{{{\rm{a}}}}}}}\right)\\=	-\mathop{\sum }\limits_{k=1}^{n}({{{{{{\bf{X}}}}}}}_{{{{{{\rm{a}}}}}}}\left[k\right]\log ({{{{{{\bf{X}}}}}}}_{{{{{{\rm{a}}}}}}}^{{{{{{\rm{pred}}}}}}}[k])+(1-{{{{{{\bf{X}}}}}}}_{{{{{{\rm{a}}}}}}}\left[k\right])\log (1-{{{{{{\bf{X}}}}}}}_{{{{{{\rm{a}}}}}}}^{{{{{{\rm{pred}}}}}}}[k])).$$

For the integrative training, we further calculate the discriminator loss L_Dis_, utilizing the BCE loss L_D_ for the classification of discriminators and implementing the soft labels technique^61^. Specifically, we first sample the smoothed positive label ${l}_{{{{{{\rm{pos}}}}}}}\sim {{{{{\rm{U}}}}}}\left[{{{{\mathrm{0.8,1}}}}}\right]$ and negative label ${l}_{{{{{{\rm{neg}}}}}}}\sim {{{{{\rm{U}}}}}}\left[{{{{\mathrm{0,0.2}}}}}\right]$, respectively for the labels 1 and 0. Subsequently, we derive the loss for the discriminator L_Dis_ as follow:

7 $${{{{{{\rm{L}}}}}}}_{{{{{{\rm{Dis}}}}}}}=	{{{{{{\rm{L}}}}}}}_{{{{{{\rm{D}}}}}}}\left({{{{{\rm{Di}}}}}}{{{{{{\rm{s}}}}}}}_{{{{{{\rm{a}}}}}}}\left({{{{{{\rm{T}}}}}}}_{{{{{{\rm{r}}}}}}\to {{{{{\rm{a}}}}}}}\left({{{{{{\rm{En}}}}}}}_{{{{{{\rm{r}}}}}}}\left({{{{{{\bf{X}}}}}}}_{{{{{{\rm{r}}}}}}}\right)\right)\right),{l}_{{{{{{\rm{neg}}}}}}}\right)+{{{{{{\rm{L}}}}}}}_{{{{{{\rm{D}}}}}}}\left({{{{{\rm{Di}}}}}}{{{{{{\rm{s}}}}}}}_{{{{{{\rm{a}}}}}}}\left({{{{{{\rm{En}}}}}}}_{{{{{{\rm{a}}}}}}}\left({{{{{{\bf{X}}}}}}}_{{{{{{\rm{a}}}}}}}\right)\right),{l}_{{{{{{\rm{pos}}}}}}}\right)\\ 	+ {{{{{{\rm{L}}}}}}}_{{{{{{\rm{D}}}}}}}\left({{{{{\rm{Di}}}}}}{{{{{{\rm{s}}}}}}}_{{{{{{\rm{r}}}}}}}\left({{{{{{\rm{T}}}}}}}_{{{{{{\rm{a}}}}}}\to {{{{{\rm{r}}}}}}}\left({{{{{{\rm{En}}}}}}}_{{{{{{\rm{a}}}}}}}\left({{{{{{\bf{X}}}}}}}_{{{{{{\rm{a}}}}}}}\right)\right)\right),{l}_{{{{{{\rm{neg}}}}}}}\right)+{{{{{{\rm{L}}}}}}}_{{{{{{\rm{D}}}}}}}\left({{{{{\rm{Di}}}}}}{{{{{{\rm{s}}}}}}}_{{{{{{\rm{r}}}}}}}\left({{{{{{\rm{En}}}}}}}_{{{{{{\rm{r}}}}}}}\left({{{{{{\bf{X}}}}}}}_{{{{{{\rm{r}}}}}}}\right)\right),{l}_{{{{{{\rm{pos}}}}}}}\right).$$

For updating the discriminators, we employ an SGD optimizer^62^ with a learning rate of 0.005.

For each iteration, we train the encoders, translator, and decoders, after the updating of discriminators. Finally, we can obtain two reconstruction results and two translation results as follows:

8 $$\begin{array}{c}{{{{{{\bf{X}}}}}}}_{{{{{{\rm{r}}}}}}\to {{{{{\rm{r}}}}}}}^{{{{{{\rm{pred}}}}}}}={{{{{\rm{D}}}}}}{{{{{{\rm{e}}}}}}}_{{{{{{\rm{r}}}}}}}\left({{{{{{\rm{T}}}}}}}_{{{{{{\rm{r}}}}}}\to {{{{{\rm{r}}}}}}}\left({{{{{\rm{E}}}}}}{{{{{{\rm{n}}}}}}}_{{{{{{\rm{r}}}}}}}\left({{{{{{\bf{X}}}}}}}_{{{{{{\rm{r}}}}}}}\right)\right)\right)\\ {{{{{{\bf{X}}}}}}}_{{{{{{\rm{r}}}}}}\to {{{{{\rm{a}}}}}}}^{{{{{{\rm{pred}}}}}}}={{{{{\rm{D}}}}}}{{{{{{\rm{e}}}}}}}_{{{{{{\rm{a}}}}}}}\left({{{{{{\rm{T}}}}}}}_{{{{{{\rm{r}}}}}}\to {{{{{\rm{a}}}}}}}\left({{{{{\rm{E}}}}}}{{{{{{\rm{n}}}}}}}_{{{{{{\rm{r}}}}}}}\left({{{{{{\bf{X}}}}}}}_{{{{{{\rm{r}}}}}}}\right)\right)\right)\\ {{{{{{\bf{X}}}}}}}_{{{{{{\rm{a}}}}}}\to {{{{{\rm{r}}}}}}}^{{{{{{\rm{pred}}}}}}}={{{{{\rm{D}}}}}}{{{{{{\rm{e}}}}}}}_{{{{{{\rm{r}}}}}}}\left({{{{{{\rm{T}}}}}}}_{{{{{{\rm{a}}}}}}\to {{{{{\rm{r}}}}}}}\left({{{{{\rm{E}}}}}}{{{{{{\rm{n}}}}}}}_{{{{{{\rm{a}}}}}}}\left({{{{{{\bf{X}}}}}}}_{{{{{{\rm{a}}}}}}}\right)\right)\right)\\ {{{{{{\bf{X}}}}}}}_{{{{{{\rm{a}}}}}}\to {{{{{\rm{a}}}}}}}^{{{{{{\rm{pred}}}}}}}={{{{{\rm{D}}}}}}{{{{{{\rm{e}}}}}}}_{{{{{{\rm{a}}}}}}}\left({{{{{{\rm{T}}}}}}}_{{{{{{\rm{a}}}}}}\to {{{{{\rm{a}}}}}}}\left({{{{{\rm{E}}}}}}{{{{{{\rm{n}}}}}}}_{{{{{{\rm{a}}}}}}}\left({{{{{{\bf{X}}}}}}}_{{{{{{\rm{a}}}}}}}\right)\right)\right),\end{array}$$

and train the encoders, translator, and decoders with a combination of reconstruction loss, translation loss, ELBO loss, and the recalculated discriminator loss with updated discriminators:

9 $${{{{{\rm{Loss}}}}}}=	{w}_{{{{{{\rm{r}}}}}}}\left({{{{{{\rm{L}}}}}}}_{{{{{{\rm{r}}}}}}}\left({{{{{{\bf{X}}}}}}}_{{{{{{\rm{r}}}}}}\to {{{{{\rm{r}}}}}}}^{{{{{{\rm{pred}}}}}}},{{{{{{\bf{X}}}}}}}_{{{{{{\rm{r}}}}}}}\right)+{{{{{{\rm{L}}}}}}}_{{{{{{\rm{r}}}}}}}\left({{{{{{\bf{X}}}}}}}_{{{{{{\rm{a}}}}}}\to {{{{{\rm{r}}}}}}}^{{{{{{\rm{pred}}}}}}},{{{{{{\bf{X}}}}}}}_{{{{{{\rm{r}}}}}}}\right)\right)+{w}_{{{{{{\rm{a}}}}}}}\left({{{{{{\rm{L}}}}}}}_{{{{{{\rm{a}}}}}}}\left({{{{{{\bf{X}}}}}}}_{{{{{{\rm{a}}}}}}\to {{{{{\rm{a}}}}}}}^{{{{{{\rm{pred}}}}}}},{{{{{{\bf{X}}}}}}}_{{{{{{\rm{a}}}}}}}\right){+{{{{{\rm{L}}}}}}}_{{{{{{\rm{a}}}}}}}\left({{{{{{\bf{X}}}}}}}_{{{{{{\rm{r}}}}}}\to {{{{{\rm{a}}}}}}}^{{{{{{\rm{pred}}}}}}},{{{{{{\bf{X}}}}}}}_{{{{{{\rm{a}}}}}}}\right)\right)\\ 	+{w}_{{{{{{\rm{ELBO}}}}}}}\left({{{{{{\rm{L}}}}}}}_{{{{{{\rm{ELBO}}}}}}}\left({{{{{{\bf{X}}}}}}}_{{{{{{\rm{embed}}}}}}}^{{{{{{\rm{r}}}}}}}\right)+{{{{{{\rm{L}}}}}}}_{{{{{{\rm{ELBO}}}}}}}\left({{{{{{\bf{X}}}}}}}_{{{{{{\rm{embed}}}}}}}^{{{{{{\rm{a}}}}}}}\right)\right)-{w}_{{{{{{\rm{dis}}}}}}}{{{{{{\rm{L}}}}}}}_{{{{{{\rm{Dis}}}}}}},$$

where ${{{{{{\bf{X}}}}}}}_{{{{{{\rm{embed}}}}}}}^{{{{{{\rm{r}}}}}}}$ and ${{{{{{\bf{X}}}}}}}_{{{{{{\rm{embed}}}}}}}^{{{{{{\rm{a}}}}}}}$ denote the shared embeddings of RNA and ATAC, respectively, L_Dis_ is recalculated using the updated discriminator for each mini-batch, and the weights of *w*_r_, *w*_a_, *w*_ELBO_ and *w*_dis_ are set to 1, 2, $\frac{20}{{I}_{{{{{{\rm{r}}}}}}}}+\frac{20}{{I}_{{{{{{\rm{a}}}}}}}}$, and 1, respectively. We update the encoders, translator, and decoders using the Adam optimizer^60^ with a learning rate of 0.001. The integrative training consists of 200 epochs with early-stop patience of 50 epochs.

### The data augmentation strategy of scButterfly

To further enhance the translation performance and address the challenges posed by the limited number of training cells and the high noise in multi-omics data, we introduce two variants of the scButterfly-B model, namely scButterfly-T (Type) and scButterfly-C (Cluster), for the scenarios with and without cell-type labels, respectively. scButterfly-T and scButterfly-C share the same model structure as scButterfly-B, while additionally employing data augmentation strategies to generate synthetic samples before model training. The generated samples could effectively simulate the original dataset and alleviate the issues of limited number of cells measured by multi-omics protocols (Supplementary Text 11).

For scButterfly-T, we partition all training samples by cell-type label, and for each cell type, generate artificial samples by pairing profiles of one modality of the randomly shuffled cells with profiles of another modality of the non-shuffled cells (Fig. 1c). To strike a balance between efficiency and performance, we perform two rounds of shuffling, resulting a total of twice as many synthetic samples as the original training set. The additional samples are mixed with the original training samples, resulting in a training dataset that is three times as large as the original dataset, for both the pretraining and integrative training phases.

In scenarios where cell type annotation is unavailable, scButterfly-C serves as a more general alternative approach by leveraging multi-omics integration techniques (Fig. 1d). scButterfly-C first trains a MultiVI^22^ model with default settings to obtain joint cell embeddings based on scRNA-seq and scATAC-seq profiles. Subsequently, Leiden^63^ clustering is used to obtain cluster labels to compensate for the lack of cell type annotation. scButterfly-C employs a high clustering resolution of three to ensure high purity of cell types within each cluster. Finally, for each Leiden cluster, scButterfly-C generates artificial samples by pairing profiles of one modality of the randomly shuffled cells with profiles of another modality of the non-shuffled cells for two rounds, also resulting in a training dataset that is three times as large as the original dataset, for both the pretraining and integrative training phases. scButterfly is a flexible framework that could also incorporate with other methods, such as detected anchors between the two modalities with Seurat, for data augmentation (Supplementary Text 12).

### Data collection, pre-processing, and post-processing

We collected multiple datasets with different modalities, species, tissues, and protocols to evaluate the performance of the scButterfly model from a comprehensive perspective. For the translation between transcriptome and chromatin profiles, we collected seven paired RNA and ATAC datasets: the BMMC dataset composed of bone marrow mononuclear cells from 10 healthy human donors by 10x-Multiome^23^, the MB and MDS datasets of adult mouse brain and dorsal skin, respectively, profiled by SHARE-seq^2^, the MK dataset of adult mouse kidney profiled by sci-CAR^3^, the MCC dataset of adult mouse cerebral cortices profiled by SNARE-seq^4^, the CL dataset of multiple cancer cell lines profiled by scCAT-seq^5^, and the PBMC dataset of peripheral blood mononuclear cells profiled by 10x-Multiome (Supplementary Fig. 3).

Additionally, we considered the diagonal analysis of unpaired data and collected eight unpaired multi-omics RNA and ATAC datasets: the UP_HK dataset of snRNA-seq and snATAC-seq profiles from five healthy human kidney samples^41^, the UP_MPMC dataset of 10x RNA v3 and snATAC-seq profiles from mouse primary motor cortex^42^, the UP_eye, UP_muscle, UP_pancreas, UP_spleen, UP_stomach, UP_thymus datasets of sci-RNA-seq^43^ and sci-ATAC-seq^44^ profiles from various human fetal organs (Supplementary Fig. 3).

We further extended scButterfly to single-cell perturbation-response prediction and collected the PT_PBMC dataset of seven cell types of control and interferon-beta-stimulated human peripheral blood mononuclear cells^47^. In addition to epigenome and transcriptome, we also investigated the translation between transcriptome and proteome profiles with two datasets: the CITE_BMMC dataset of bone marrow mononuclear cells from the same human donors as the BMMC dataset^23^ and the CITE_BM dataset of scRNA-seq profiles alongside a panel of antibodies from human bone marrow^51^ (Supplementary Fig. 3).

We provide a summary of the above-mentioned datasets (Supplementary Fig. 3), including more detailed information such as the number of cells, features, batches and cell types, as well as the imbalance of cell types, sparsity, protocol and species of these datasets.

For data pre-processing, we applied conventional methods specific to different modalities (Fig. 1a). For the count matrices of scRNA-seq profiles, we normalized the total count of each cell to have the same values equal to the median of total counts for cells before normalization, logarithmized the normalized values with an adding offset of one, and selected the top 3000 highly variable genes (HVGs)^27,51^ for scButterfly training and downstream analysis.

For the count matrices of scATAC-seq profiles, we binarized the matrices, filtered out the peaks activated in less than 0.5% of all cells, and performed term frequency-inverse document frequency (TF-IDF) transformation as follows^25,38,64^:

10 $${{{{{\bf{TF}}}}}}\left[i\right]\left[j\right]=	\frac{{{{{{{\bf{X}}}}}}}_{{{{{{\rm{a}}}}}}}^{{{{{{\rm{bin}}}}}}}\left[i\right]\left[j\right]}{{\sum }_{k=1}^{{I}_{{{{{{\rm{a}}}}}}}}{{{{{{\bf{X}}}}}}}_{{{{{{\rm{a}}}}}}}^{{{{{{\rm{bin}}}}}}}\left[i\right]\left[k\right]}\\ {{{{{\bf{IDF}}}}}}\left[i\right]\left[j\right]=	{log} \left(1+\frac{n}{{\sum }_{k=1}^{n}{{{{{{\bf{X}}}}}}}_{{{{{{\rm{a}}}}}}}^{{{{{{\rm{bin}}}}}}}\left[k\right]\left[j\right]}\right)\\ {{{{{{\bf{X}}}}}}}_{{{{{{\rm{a}}}}}}}^{{{{{{\rm{TFIDF}}}}}}}=	{{{{{\bf{TF}}}}}}\times {{{{{\bf{IDF}}}}}},$$

where ${{{{{{\bf{X}}}}}}}_{{{{{{\rm{a}}}}}}}^{{{{{{\rm{bin}}}}}}}$ and ${{{{{{\bf{X}}}}}}}_{{{{{{\rm{a}}}}}}}^{{{{{{\rm{TFIDF}}}}}}}$ are the matrices before and after TF-IDF transformation, respectively. The matrix was then scaled to the range of [0,1] by dividing the elements by the maximum *S* of the matrix.

For the perturbation-response dataset of PT_PBMC, we retrieved the processed data in scGen^45^ and performed no additional processes to maintain the consistency in evaluation.

For the count matrices of proteome profiles, we performed the centered log ratio (CLR) transformation across cells^51^, with the formula as follows:

11 $${{{{{\rm{CLR}}}}}}\left({{{{{\bf{x}}}}}}\right)=log \left(\frac{{x}_{1}}{{{{{{\rm{g}}}}}}\left({{{{{\bf{x}}}}}}\right)},\ldots \ldots,\frac{{x}_{n}}{{{{{{\rm{g}}}}}}\left({{{{{\bf{x}}}}}}\right)}\right),$$

where ${{{{{\bf{x}}}}}}=({x}_{1},\ldots \ldots,{x}_{n})$ represents the count vector of protein epitopes for each cell, and g(**x**) denotes the geometric mean of (*x*_1_,……,*x*_*n*_).

For data post-processing, we set the values in the predicted RNA and ATAC matrices to zeros if they fell below the threshold of 1e-4, considering the high sparsity nature of original profiles. Additionally, we devised a method to recover count matrices from ATAC predictions, to ensure downstream methods that require count matrices, such as MultiVI^22^, could utilize the count matrices as input. Specifically, the inverse process involves reversing the scaling and TF-IDF transformation in the pre-processing stage:

12 $${{{{{{\bf{X}}}}}}}_{{{{{{\rm{r}}}}}}\to {{{{{\rm{a}}}}}}}^{{{{{{\rm{count}}}}}}}\left[i\right]\left[j\right]={{{{{{\bf{X}}}}}}}_{{{{{{\rm{r}}}}}}\to {{{{{\rm{a}}}}}}}^{{{{{{\rm{sparse}}}}}}}\left[i\right]\left[j\right]\times S/{{{{{\bf{IDF}}}}}}\left[i\right]\left[j\right]\times \mathop{\sum }\limits_{k=1}^{{I}_{{{{{{\rm{a}}}}}}}}{{{{{{\bf{X}}}}}}}_{{{{{{\rm{a}}}}}}}^{{{{{{\rm{bin}}}}}}}\left[i\right]\left[k\right],$$

where ${{{{{{\bf{X}}}}}}}_{{{{{{\rm{r}}}}}}\to {{{{{\rm{a}}}}}}}^{{{{{{\rm{sparse}}}}}}}$ represents the sparse version of the output ${{{{{{\bf{X}}}}}}}_{{{{{{\rm{r}}}}}}\to {{{{{\rm{a}}}}}}}^{{{{{{\rm{pred}}}}}}}$ of scButterfly, *S* is the scale factor in pre-processing, **IDF** and ${\sum }_{k=1}^{{I}_{{{{{{\rm{a}}}}}}}}{{{{{{\bf{X}}}}}}}_{{{{{{\rm{a}}}}}}}^{{{{{{\rm{bin}}}}}}}\left[i\right]\left[k\right]$ are the matrices in TF-IDF transformation in pre-processing. We finally set the elements of ${{{{{{\bf{X}}}}}}}_{{{{{{\rm{r}}}}}}\to {{{{{\rm{a}}}}}}}^{{{{{{\rm{count}}}}}}}$ whose value was greater than both the corresponding column and row means to ones, and to zeros otherwise^1^, resulting binarized count matrices for downstream analysis.

### Unpaired data training with scButterfly

In addition to translating paired multi-modal profiles, scButterfly can also perform the more challenging diagonal analysis of unpaired data using a similar method as the data augmentation strategy of scButterfly-T. Taking the translation between transcriptome and chromatin profiles as an example, we first identify the shared cell types of unpaired scRNA-seq and scATAC-seq single-modal datasets. Suppose that there are RNA profiles ${{{{{{\bf{X}}}}}}}_{{{{{{\rm{r}}}}}}}\in {{\mathbb{R}}}^{{n}_{{{{{{\rm{r}}}}}}}\times {I}_{{{{{{\rm{r}}}}}}}}$ and ATAC profiles ${{{{{{\bf{X}}}}}}}_{{{{{{\rm{a}}}}}}}\in {{\mathbb{R}}}^{{n}_{{{{{{\rm{a}}}}}}}\times {I}_{{{{{{\rm{a}}}}}}}}$ with *n*_r_ and *n*_a_ cells respectively. Let *m* represent the count of shared cell types, and for type $i\le m$, let ${s}_{i}^{{{{{{\rm{r}}}}}}}$ and ${s}_{i}^{{{{{{\rm{a}}}}}}}$ separately denote the proportion of this type in **X**_r_ and **X**_a_. Then the proportion of sampling for type *i* is given by:

13 $${s}_{i}=\frac{{s}_{i}^{{{{{{\rm{r}}}}}}}+{s}_{i}^{{{{{{\rm{a}}}}}}}}{{\sum }_{k=1}^{m}\left({s}_{k}^{{{{{{\rm{r}}}}}}}+{s}_{k}^{{{{{{\rm{a}}}}}}}\right)},$$

which represents the normalized average proportion of type *i*. Next, we sample $\frac{{{s}_{i}}(n_{{{{{{\rm{r}}}}}}}+{n}_{{{{{{\rm{a}}}}}}})}{2}$ paired profiles for model training by randomly matching the RNA profile of one cell of type *i* with the ATAC profile of another cell of type *i*. Although the training process only focuses on the shared cell types between two single-modal datasets, we included all cell types of each single-modal dataset in the testing phase.

### Single-cell perturbation-response prediction with scButterfly

The scButterfly framework can be generalized to translate between transcriptome profiles before and after perturbation, enabling the prediction of single-cell perturbation responses. In terms of the model architecture, we replace the ATAC encoder and decoder with the RNA encoder and decoder, respectively. One pair of RNA encoder and decoder is dedicated to modeling gene expression data from the control group, while another pair of RNA encoder and decoder is responsible for modeling gene expression data from the stimulated group.

For model training, we utilize optimal transport to match cells and generate paired training samples^48^. Specifically, we first divide cells into different groups based on their cell types and perform principal component analysis (PCA) to reduce the dimension to 50 for each group. Denoting the number of cell types as *m*, then for type $k\le m$, we calculate the Euclidean distance cost matrix **M**_*k*_ between the 50-dimensional representations of control and stimulated data. In the absence of prior knowledge, a uniform distribution assumption is made for both the control and stimulated groups, represented by weight vectors ${{{{{{\bf{w}}}}}}}_{k}^{{{{{{\rm{ctr}}}}}}}{{{{{\boldsymbol{=}}}}}}\left(\frac{1}{{n}_{k}^{{{{{{\rm{ctr}}}}}}}}{{{{{\boldsymbol{,}}}}}}\frac{1}{{n}_{k}^{{{{{{\rm{ctr}}}}}}}}{{{{{\boldsymbol{,}}}}}}{{{{{\boldsymbol{.}}}}}}{{{{{\boldsymbol{.}}}}}}{{{{{\boldsymbol{.}}}}}}{{{{{\boldsymbol{,}}}}}}\frac{1}{{n}_{k}^{{{{{{\rm{ctr}}}}}}}}\right)\in {{\mathbb{R}}}^{1\times {n}_{k}^{{{{{{\rm{ctr}}}}}}}}$ and ${{{{{{\bf{w}}}}}}}_{k}^{{{{{{\rm{sti}}}}}}}{{{{{\boldsymbol{=}}}}}}\left(\frac{1}{{n}_{k}^{{{{{{\rm{sti}}}}}}}}{{{{{\boldsymbol{,}}}}}}\frac{1}{{n}_{k}^{{{{{{\rm{sti}}}}}}}}{{{{{\boldsymbol{,}}}}}}{{{{{\boldsymbol{.}}}}}}{{{{{\boldsymbol{.}}}}}}{{{{{\boldsymbol{.}}}}}}{{{{{\boldsymbol{,}}}}}}\frac{1}{{n}_{k}^{{{{{{\rm{sti}}}}}}}}\right)\in {{\mathbb{R}}}^{1\times {n}_{k}^{{{{{{\rm{sti}}}}}}}}$, where ${n}_{k}^{{{{{{\rm{ctr}}}}}}}$ and ${n}_{k}^{{{{{{\rm{sti}}}}}}}$ denote the cell counts of control and stimulated groups of type *k*, respectively. Then the optimal transport problem could be formulated as the Earth Movers Distance (EMD) problem:

14 $${{{{{{\boldsymbol{\gamma }}}}}}}_{k}=\mathop{{{{{\rm{argmin}}}}}}\limits_{{{{{{\boldsymbol{\gamma }}}}}}} < {{{{{\boldsymbol{\gamma }}}}}},{{{{{{{\bf{M}}}}}}}_{k}} > _{{{{{{\rm{F}}}}}}} \\ s.t.\left\{\begin{array}{c}{{{{{\boldsymbol{\gamma }}}}}}\cdot {{{{{\bf{1}}}}}}={{{{{{\bf{w}}}}}}}_{k}^{{{{{{\rm{ctr}}}}}}}\\ {{{{{{\boldsymbol{\gamma }}}}}}}^{{{{{{\boldsymbol{T}}}}}}} \cdot {{{{{\bf{1}}}}}}={{{{{{\bf{w}}}}}}}_{k}^{{{{{{\rm{sti}}}}}}}\\ {{{{{\boldsymbol{\gamma }}}}}}{{\ge }}{{{{{\bf{0}}}}}}\hfill\end{array}\right.,$$

where **γ**_*k*_ is the optimal transport matrix for control and stimulated data of type *k*,$< {{{{{\boldsymbol{\gamma }}}}}}{{{{{\boldsymbol{,}}}}}}{{{{{{{\bf{M}}}}}}}_{k} > }_{{{{{{\rm{F}}}}}}}$ is the Frobenius inner product, defined as ${\sum }_{i=1}^{{n}_{k}^{{{{{{\rm{ctr}}}}}}}}{\sum }_{j=1}^{{n}_{k}^{{{{{{\rm{sti}}}}}}}}{{{{{\boldsymbol{\gamma }}}}}}[i][j]{{{{{{\bf{M}}}}}}}_{k}[i][j]$. We use the algorithm in ref. ^48^ to solve this problem. Finally, for cell type *k*, we select the stimulated cell with the highest value in **γ**_*k*_ for each cell in the control group, thereby generating paired samples by pairing each control profile with its corresponding stimulated profile to train scButterfly. Note that some stimulated cells may be paired with multiple control cells, however, this kind of reusing will not significantly affect the prediction performance of scButterfly (Supplementary Text 13).

### Translation between transcriptome and proteome with scButterfly

scButterfly can be easily extended to facilitate the translation between transcriptome and proteome profiles through specific modifications to the model structure and training strategy. In terms of the model structure, we maintain the consistency in the RNA encoder, RNA decoder, translator, and discriminators as the translation between transcriptome and chromatin profiles. For the ADT (Antibody-Tagged Detection) encoder, the processed data is projected into latent space via two blocks of 128-dimensional fully connected layer and LeakyReLU. The ADT decoder performs the reverse process by recovering the mapped/translated embeddings to the original dimension. Given the relatively high quality of ADT data, we do not use the masking strategy for ADT input. We also discussed the impact of different embedding dimensions on translation performance in Supplementary Text 14.

During the model training phase, we follow the same procedure as the translation between transcriptome and chromatin profiles. However, due to the generally lower dimensionality of ADT profiles in comparison to RNA or ATAC profiles, we assign a constant weight of $\frac{1}{150}$ to the KL divergence term associated with the ADT component. This precautionary measure is taken to mitigate the potential occurrence of posterior collapse, which can happen if the weight for the ELBO term is set too high and the variational posterior distribution closely matches the prior for a subset of latent variables^65^.

For the data augmentation strategy, no modifications are made for the scButterfly-T variant. However, for the scButterfly-C variant, instead of MultiVI^22^, we employ totalVI^66^, which is a multi-omics integration method specifically designed for transcriptome and proteome profiles. Specifically, we train totalVI with default settings to obtain joint embeddings of paired RNA and ADT profiles, then perform Leiden clustering^63^ with a resolution of three based on the cell embeddings, and finally augment the dataset and train scButterfly-C using the same approach as scButterfly-C for the translation between RNA and ATAC profiles.

### Evaluation metrics

To quantitatively evaluate the cell heterogeneity preserved in translated profiles, we first performed PCA to reduce the dimensionality of translated profiles to 50, then performed cell clustering by the Leiden algorithm with default resolution of one^63^ based on the dimensionality reduction results, and finally assessed the clustering results by four widely-used metrics^24–26^, including adjusted Rand index (ARI), adjusted mutual information (AMI), normalized mutual information (NMI), and homogeneity (HOM). Rand index (RI) computes a similarity measure between the cluster labels and the cell-type labels. ARI is adjusted based on RI and accounts for chance agreement. Mutual information (MI) quantifies the correlation between the cluster labels and the cell-type labels. NMI is a normalized variant of MI, while AMI further considers chance agreement based on MI. HOM measures the purity of cell types within each cluster, and it equals one if all the cells within the same cluster belong to the same cell type. Note that the sizes of cell populations in most single-cell data are unbalanced and AMI is more appropriate in most cases since it is preferred when the sizes of clusters are unbalanced, while ARI is preferred when the clusters have nearly equal-sizes^54^.

To evaluate the performance of cell type annotation, we adopted four metrics as suggested by recent studies^38,39^, including accuracy (Acc), Cohen’s kappa value (Kappa), F1-macro, and F1-weighted. Acc provides a direct measure of the agreement between the annotated and ground-truth cell-type labels. Kappa takes chance agreement into consideration and is particularly suitable for non-ordinal categorical variables. Both F1-macro and F1-weighted are derived from F1-score: F1-macro is the arithmetic mean of the F1-scores for all cell-type labels, while F1-weighted is a weighted sum of the F1-scores. Compared to Acc, F1-macro and F1-weighted give relatively equal attention to both common and rare cell types, providing a more comprehensive evaluation of the annotation performance.

To evaluate the performance of single-cell perturbation-response prediction, we adopted two metrics that were universally used in existing studies^45,46^. First, we performed differential gene expression analysis between the control data and the real stimulated data, resulting real differentially expressed genes (DEGs), and between the control data and the predicted stimulated data, resulting predicted DEGs. To assess the capability of preserving the biological variance in real data, we then counted the number of common DEGs of the top 100 real DEGs versus the top 100 predicted DEGs. Second, to examine the consistency between the predicted perturbation responses and the ground truth responses, we randomly sampled 80% of the test data with replacement 100 times and computed the squared Pearson correlation (*R*^2^) for mean gene expression of the top 100 real DEGs between predicted and real stimulated data.

For the translation between transcriptome and proteome profiles, in addition to the above four clustering metrics for evaluating the cell heterogeneity preserved in translated profiles, we further evaluated the translated proteome profiles from a numerical accuracy standpoint, given the low dimensionality and dense nature of ADT data^10,66^. Specifically, for the real and predicted protein expression level of each cell, we investigated the correlation coefficients via Pearson correlation and Spearman correlation, respectively.

More detailed mathematical equations and formulas for the aforementioned metrics are provided in Supplementary Text 15.

### Visualization

For data visualization, we performed PCA to reduce the dimensionality of translated profiles to 50 and then adopted the t-SNE^67^ method to further reduce the dimension to two. Cells in the visualization could be colored by cell-type labels, batch indices, or clustering labels.

### Baseline methods

For the translation between transcriptome and chromatin profiles, we compared the performance of scButterfly against three state-of-the-art methods, including BABEL^1^, Polarbear^8^, and JAMIE^15^. We implemented BABEL and JAMIE using their respective GitHub repositories with the default parameter settings. For Polarbear, we implemented it used the default parameters on the NGC docker with pre-compiled TensorFlow v1.15. Because of the extremely high dimension of chromatin profiles (1,050,819 peaks) in the datasets of UP_eye, UP_muscle, UP_pancreas, UP_spleen, UP_stomach and UP_thymus, we additionally adapted the same features selection strategy as scButterfly when implementing baseline methods or encountered memory errors otherwise. Note that we did not consider the cross-modal autoencoder^9^ and UnitedNet^16^ for comparison because the former is mainly designed for translation between scRNA-seq data and chromatin images and does not provide the source code for its intricate data processing steps, while the latter does not provide the guideline for number determination of the features to be selected as well as the code for data processing and performs translation between epigenome and transcriptome in a supervised manner that requires ground-truth cell-group identification.

For the cell type annotation of scATAC-seq data, we compared the performance of scButterfly with the state-of-the-art EpiAnno^38^ and Cellcano^39^ methods. For the prediction of single-cell perturbation responses, we compared the performance of scButterfly with the advanced scGen^45^ and scPreGAN^46^ methods. For the translation between transcriptome and proteome profiles, we compared the performance of scButterfly with the latest sciPENN^10^ and JAMIE^15^ methods. Note that we implemented the above baseline methods following their tutorials and with their default settings.

### Reporting summary

Further information on research design is available in the Nature Portfolio Reporting Summary linked to this article.

### Supplementary information

Supplementary Information

Peer Review File

Reporting Summary

### Source data

Source Data files

## Data Availability

All relevant data supporting the key findings of this study are available within the article and its Supplementary Information files. The BMMC and CITE_BMMC datasets were collected from NCBI Gene Expression Omnibus (GEO) with the accession number GSE194122^23^ [https://www.ncbi.nlm.nih.gov/geo/query/acc.cgi?acc=GSE194122]. The MB and MDS datasets can be accessed in GEO with the accession number GSE140203^2^ [https://www.ncbi.nlm.nih.gov/geo/query/acc.cgi?acc=GSE140203]. The CL dataset was collected from the supplementary data 3 and 4 of previous study^5^ [https://www.nature.com/articles/s41467-018-08205-7]. The MCC dataset is available at GEO with the accession number GSE126074^4^ [https://www.ncbi.nlm.nih.gov/geo/query/acc.cgi?acc=GSE126074]. The MK dataset was collected from GEO with the accession number GSE117089^3^ [https://www.ncbi.nlm.nih.gov/geo/query/acc.cgi?acc=GSE117089]. The PBMC dataset can be accessed at https://support.10xgenomics.com/single-cell-gene-expression/datasets/3.0.0/pbmc_10k_v3. The UP_HK dataset was collected from GEO with the accession number GSE151302^41^ [https://www.ncbi.nlm.nih.gov/geo/query/acc.cgi?acc=GSE151302]. The UP_MPMC dataset is available in the NeMO archive (RRID: SCR_016152)^42^ [https://assets.nemoarchive.org/dat-ch1nqb7]. The six unpaired datasets from human fetal atlas (UP_eye, UP_muscle, UP_pancreas, UP_spleen, UP_stomach, UP_thymus) were derived from two studies^43,44^ [https://descartes.brotmanbaty.org/]. The CITE_BM dataset can be accessed in GEO with the accession number GSE128639^51^ [https://www.ncbi.nlm.nih.gov/geo/query/acc.cgi?acc=GSE128639]. The PT_PBMC dataset was collected from https://github.com/theislab/scgen-reproducibility^45^. Source data are provided with this paper.

## Code availability

scButterfly algorithm is implemented in Python based on the PyTorch framework. The MIT-licensed scButterfly software including detailed documents and tutorials is freely available at https://github.com/BioX-NKU/scButterfly^68^. The source code for reproduction is available at https://github.com/BioX-NKU/scButterfly_source^68^.
