# Supplementary material for: scButterfly: a versatile single-cell cross-modality translation method via dual-aligned variational autoencoders
Source: Nat Commun. 2024 Apr 6;15:2973. doi: 10.1038/s41467-024-47418-x (PMC10998864; doi:10.1038/s41467-024-47418-x)
Supplement: Supplementary file 1 — Supplementary Information [file 41467_2024_47418_MOESM1_ESM.pdf]

Supplementary Information for

scButterfly: a versatile single-cell cross-modality translation method via dual-aligned variational autoencoders

Yichuan Cao[1], Xiamiao Zhao[1], Songming Tang[1], Qun Jiang[2], Sijie Li[1], Siyu Li[3] and Shengquan Chen[1,*]

[1] School of Mathematical Sciences and LPMC, Nankai University, Tianjin 300071, China

[2] MOE Key Laboratory of Bioinformatics and Bioinformatics Division of BNRIST, Department of Automation, Tsinghua University, Beijing 100084, China

[3] School of Statistics and Data Science, Nankai University, Tianjin 300071, China

[*] Corresponding author: chenshengquan@nankai.edu.cn

Contents

Supplementary Texts 5
Supplementary Text 1 5
Supplementary Text 2 9
Supplementary Text 3 12
Supplementary Text 4 14
Supplementary Text 5 16
Supplementary Text 6 17
Supplementary Text 7 18
Supplementary Text 8 20
Supplementary Text 9 22
Supplementary Text 10 25
Supplementary Text 11 29
Supplementary Text 12 31
Supplementary Text 13 33
Supplementary Text 14 35
Supplementary Text 15 37
Supplementary Figures 40
Supplementary Figure 1 40
Supplementary Figure 2 41
Supplementary Figure 3 42
Supplementary Figure 4 43
Supplementary Figure 5 44
Supplementary Figure 6 45
Supplementary Figure 7 46
Supplementary Figure 8 47
Supplementary Figure 9 48
Supplementary Figure 10 49
Supplementary Figure 11 50
Supplementary Figure 12 51

Supplementary Figure 13 .... **52**
Supplementary Figure 14 .... **53**
Supplementary Figure 15 .... **54**
Supplementary Figure 16 .... **55**
Supplementary Figure 17 .... **56**
Supplementary Figure 18 .... **57**
Supplementary Figure 19 .... **58**
Supplementary Figure 20 .... **59**
Supplementary Figure 21 .... **60**
Supplementary Figure 22 .... **61**
Supplementary Figure 23 .... **62**
Supplementary Figure 24 .... **63**
Supplementary Figure 25 .... **64**
Supplementary Figure 26 .... **65**
Supplementary Figure 27 .... **66**
Supplementary Figure 28 .... **67**
Supplementary Figure 29 .... **68**
Supplementary Figure 30 .... **69**
Supplementary Figure 31 .... **70**
Supplementary Figure 32 .... **71**
Supplementary Figure 33 .... **72**
Supplementary Figure 34 .... **73**
Supplementary Figure 35 .... **74**
Supplementary Figure 36 .... **75**
Supplementary Figure 37 .... **76**
Supplementary Figure 38 .... **77**
Supplementary Figure 39 .... **78**
Supplementary Figure 40 .... **79**
Supplementary Figure **80**
Supplementary Tables **81**

Supplementary Table 1 .... **81**
Supplementary Table 2 .... **82**
Supplementary References **83**

Supplementary Texts

Supplementary Text 1: Discussion of the potential limitations with previous methods concerning batch effects for cross-modality translation

The issues of unseen batches or unseen cell types in prediction tasks are essentially the result of differences in the distribution of training and testing samples, which has been widely investigated in machine learning as the problem of dataset shift[1-3]. The dataset shift in biological datasets has also been extensively studied, especially in the context of batch correction[4-6] and analysis with unseen cell types[7-9]. In the case of cross-modality translation problems, only a few methods including BABEL and Polarbear have taken into account the translation for cells from different batches and cell types, which better aligns with real-world scenarios. However, we found that these issues haven't yet been fully resolved and remain to be challenging. Specifically, we conducted four-fold cross-validation by batch on the three multi-batch datasets (BMMC, CL, and MDS) and three-fold cross-validation by cell type on the other four datasets (MB, MCC, MK, and PBMC). As shown in Fig. 3ab, both BABEL and Polarbear struggled to maintain reasonable performance when dealing with unseen batches or unseen cell types. Contrarily, scButterfly model outperformed BABEL and Polarbear (Fig. 3ab and Supplementary Figs. 7-11), indicating the advantage of scButterfly in the cross-modality translation scenario with unseen batches or unseen cell types. This result demonstrated that existing methods have not adequately addressed this problem of translating on datasets with dataset shift.

In addition, it is an optional scheme to perform batch correction before training, enabling cross-modality translation on unseen batches. However, we believe that there are some other

potential issues with the existing batch correction methods[4, 5]. These issues have been raised in many batch correction studies and could be summarized as follows: (1) many widely-used batch correction methods output dimensionality-reduced embeddings to correct batch effects[10-14], but scButterfly cannot directly apply these dimensionality-reduced embeddings for cross-modality translation. This means that scButterfly cannot be easily combined with these advanced batch correction methods to achieve the optimal batch correction performance. (2) recent researches have pointed out that batch correction methods need to balance between correcting batch effects and preserving biological heterogeneity[4, 5, 15]. Although the batch correction methods effectively reduce the batch effects, they also lead to some loss of bio-conservation. Besides, since the data augmentation strategy already enabled scButterfly to be less affected by batch effects (Supplementary Text 9), the improvement brought by adding a batch correction step may not be able to compensate for the impact of biological heterogeneity loss on the translation performance. These issues result in the combination of batch effect correction methods and scButterfly being unable to achieve better performance. We utilized existing batch correction methods to perform batch correction on the training and testing datasets and trained scButterfly on the corrected data. Specifically, we performed batch correction using scGen and pyComBat separately for RNA and ATAC data on the BMMC, CL, and MDS datasets and trained the basic scButterfly model for translation. As suggested in recent research[5], scGen[16] and pyComBat[17] could separately achieve the best overall performance among the batch effect correction methods which could output the corrected original dimension count matrix. We performed cross-validation by batch (Supplementary Fig. 25) and it can be observed that the batch-effects-corrected data did not improve translation

performance, suggesting that previous methods still have some underlying limitations and the challenge in analyses for unseen batches and cell types remains urgent.

We further evaluated the effectiveness of performing batch correction before data augmentation on a simulated dataset, which contained higher batch effects compared to the original dataset. Based on the original CL dataset, we applied the advanced scDesign3[18] method to simulate RNA profiles and generated an artificial RNA dataset containing 3000 cells. The CL dataset consists of 549 cells from 4 different batches, with some batches lacking part of cell types (Columns #1 and #3 in Supplementary Fig. 26a). We maintained the proportions of different cell types and the variations between cell types during generation, and further amplified the batch effects. For ATAC profiles, although scDesign3 could simulate ATAC profiles with differentially accessible peaks[18], it failed to tackle the original ATAC profiles with 157,358 peaks. Therefore, we performed the newer ATAC simulation method, simCAS[19], to generate the artificial ATAC profiles. Similar to the simulation of RNA, we generated a 3000-cell artificial ATAC dataset with increased batch effects and preserved the proportional discrepancy of cell types and batches. As shown in Columns #2 and #4 in Supplementary Fig. 26a, the simulated RNA and ATAC profiles significantly magnified the differences between batches compared to the original dataset (Columns #1 and #3 in Supplementary Fig. 26a). This simulated unpaired dataset enabled us to better assess the impact of incorporating batch correction step before data augmentation, in the scenarios with large batch effects.

We conducted a five-fold cross-validation on this simulated unpaired dataset and evaluated the translation performance of scButterfly-T and scButterfly-T-with-batch-correction. As described in our first revision, the batch correction step was performed by scGen[16] and

pyComBat[17], respectively for RNA and ATAC profiles. As shown in Supplementary Fig. 26b, scButterfly-T-with-batch-correction still performed worse compared to scButterfly-T. We also visualized the translated profiles from scButterfly-T and scButterfly-T-with-batch-correction. Although the RNA to ATAC translated profiles of scButterfly-T still contained some batch effects (Column #2 in Supplementary Fig. 26c), the RNA to ATAC translated profiles of scButterfly-T-with-batch-correction were difficult to distinguish cell type HCT-116 and PDX2 (Column #4 in Supplementary Fig. 26c), indicating that batch correction may lead to some biological heterogeneity loss, resulting in a decrease in translation performance. These results demonstrate that even for the data with large batch effects, the existing batch correction methods may still not be suitable for use in the cross-modality translation of scButterfly.

Supplementary Text 2: Discussion of biological insights for evaluating the translation performance from both RNA to ATAC and ATAC to RNA with cell clustering

We discussed the biological insights for the utilization of clustering metrics and for evaluating both RNA to ATAC and ATAC to RNA translations, respectively. For the biological insights of mainly focusing on clustering metrics for evaluation, we demonstrate that clustering metrics offer three advantages. Firstly, the clustering metrics have been widely used in evaluating various tasks, such as batch alignment and cell type annotation[5, 20-24], indicating their ability to reflect the model's performance in multiple tasks. Secondly, evaluating the clustering metrics often provides insights into the ability of prediction to preserve cell heterogeneity. As shown in Supplementary Fig. 27, translated profiles with higher clustering metrics tend to exhibit more distinct visualizations, implying that the translated profiles are more adept at preserving cell heterogeneity. Thirdly, in addition to clustering metrics, correlation coefficients, such as Pearson and Spearman correlation coefficients, are widely used indicators for assessing translation effectiveness. Compared to correlation coefficients, clustering metrics are less susceptible to noise and sparsity[25] present in the single-cell data[26]. Moreover, it has been shown that correlation coefficients are non-robust to outliers[27] and inaccurate when applied to high-dimensional data[28]. In brief, evaluating with the clustering metrics could accurately measure the translation performance of the model and is suitable for reflecting the ability of the model for multiple downstream analyses.

For the biological insights of simultaneously evaluating RNA to ATAC and ATAC to RNA translations, we emphasize that, although ATAC to RNA translation often performs worse than RNA to ATAC translation, both of them provide valuable biological insights. Firstly, RNA and

ATAC can capture distinct types of cellular information and provide multi-perspective insights into cell states. While RNA data often has higher quality and lower noise compared to ATAC data, offering comprehensive gene expression profiles, it fails to capture the chromatin regulatory landscape that governs transcription in each cell type[29]. Secondly, for single-cell multi-omics profiles, the information from RNA and ATAC data should be complementary to each other rather than substitute. For example, in the MDS dataset, using the original ATAC data, we can distinguish Basal, Spinous, and Infundibulum cells in t-Distributed Stochastic Neighbor Embedding (t-SNE) visualization (marked with red boxes) that cannot be distinguished using the original RNA data (Row #6 in Supplementary Fig. 27). Conversely, using the original RNA data, we could differentiate Dermal Fibroblast, Dermal Papilla, Dermal Sheath, Endothelial, and Melanocyte cells, whereas the original ATAC data mixed them together (marked with blue boxes). This result indicated that RNA and ATAC data usually possess unique information respectively and could be complementary. Thus, we should fully exploit this information for multi-modal data analysis, rather than discarding one of them. Thirdly, both RNA to ATAC and ATAC to RNA translations have practical applications. With the use of the scButterfly model trained on multi-modal data, massive single-modality RNA or ATAC data accumulated in repositories[30-32] could be translated into different modalities and be utilized for modality-specific downstream analysis. For example, through ATAC to RNA translation, we can conduct differential expression analysis on single-modality ATAC data, identifying the differentially expressed genes (DEGs) and performing gene ontology enrichment analysis[33, 34] (Section “scButterfly effectively translates data of novel contexts and reveals biological insights”). Besides, by employing advanced RNA annotation methods to

translated profiles, we could accurately annotate the unlabeled ATAC data, which is challenging for directly performing annotation on the ATAC data (Section “scButterfly facilitates integrative analysis, data enhancement, and cell type annotation”). Through RNA to ATAC translation, we can perform ATAC-specific analyses such as single-nucleotide polymorphisms analysis[35] and TF regulatory networks inferring[36], for single-modality RNA data (Section “scButterfly effectively translates data of novel contexts and reveals biological insights”), helping understand the mechanisms of interaction between transcription factors. Additionally, as demonstrated in the integrative analysis section (Section “scButterfly facilitates integrative analysis, data enhancement, and cell type annotation”), both RNA to ATAC and ATAC to RNA translations enable us to complement single-modality RNA or ATAC data for further integrative analysis, yielding more comprehensive results than single-modal analyses. Fourthly, RNA to ATAC and ATAC to RNA translations can also be used together when dealing with low-quality paired data. Experimental results of data enhancement exhibited that, the quality of the data improved after model translation, and the translated profiles could be better utilized in downstream analyses, such as integrative analyses (Section “scButterfly facilitates integrative analysis, data enhancement, and cell type annotation”). In summary, we identified that both RNA to ATAC and ATAC to RNA can complementarily capture diverse information in the original data, and can be utilized separately or jointly for multiple downstream analyses and applications, providing versatile biological insights.

Supplementary Text 3: Discussion of the performance difference between the RNA to ATAC translation and the ATAC to RNA translation

It is evident that RNA to ATAC translation generally exhibits superior clustering performance compared to ATAC to RNA translation. We attributed this difference in clustering performance primarily to the quality of the input data. We addressed this inference with the following two points. Firstly, RNA data often possess higher quality compared to ATAC data. Recent studies have demonstrated that ATAC data typically manifest higher dimensionality and sparsity, leading to increased noise compared to RNA[22, 37-39]. As depicted in Columns #1 and #3 in Supplementary Fig. 27, the original RNA data consistently achieves better clustering performance than the original ATAC data. Secondly, we clarify that the qualities of RNA to ATAC and ATAC to RNA translations are strongly influenced by the qualities of original RNA and ATAC data, respectively. The results in Supplementary Fig. 27 illustrate a strong association between the clustering metrics of the RNA to ATAC translation and original RNA data. We also observed this association between the clustering metrics of the ATAC to RNA translation and original ATAC data. Particularly in the MB and MK datasets, the quality difference between original RNA data and noisy original ATAC data led to a significant disparity in translation performance (Rows #2 and #5 in Supplementary Fig. 27). Furthermore, we conducted five-fold cross-validation on seven datasets including BMMC, MB, CL, MCC, MK, MDS, and PBMC. We evaluated the clustering metrics of the AMI, ARI, HOM, and NMI of the original RNA data, the original ATAC data, RNA to ATAC translation, and ATAC to RNA translation from scButterfly-B, respectively. For each clustering metric, we calculated the Pearson correlation coefficients between the metric values of the original data and that of

translated profiles among seven datasets. Supplementary Table 2 illustrated that the clustering performance of RNA to ATAC translation had a stronger correlation with the clustering metrics of original RNA data, compared to the metrics of the original ATAC data. For the ATAC to RNA translation, we also observed a similar stronger correlation with the metrics of original ATAC data, compared to the metrics of original RNA data. These results indicated that the cell clustering metrics of the translated profiles from scButterfly may have a strong association with the clustering metrics of input data. Taking the above two points into consideration, we inferred that the difference in clustering performance between the two methods primarily stems from the variance in input data quality.

Supplementary Text 4: Discussion of the additional noise introduced by data augmentation

For the data augmentation with cell-type labels, scButterfly-T generate synthetic samples by randomly pair two omics of the cells in the same cell types (Methods). Some additional noise may be introduced during this augmentation step. However, the level of heterogeneity within one cell type is usually smaller than the heterogeneity between different cell types. We visualized the seven paired datasets (BMMC, MB, CL, MCC, MK, MDS, and PBMC) with t-SNE. As shown in Supplementary Fig. 27, cells of the same type in the original data generally tend to form tightly clustered groups, while cells of different types are more likely to be loosely distributed in the visualizations. Therefore, we believe that these noises may not have a significant impact on the learning for the cell type-specific characteristics of scButterfly.

Secondly, in addition to scButterfly-T, we also proposed scButterfly-C (**C**luster) with data augmentation based on cluster labels, which may have the potential ability to address this concern. The scButterfly-C variant incorporates data augmentation by randomly pairing two omics of the cells within the same Leiden[40] clusters. Furthermore, we have employed a high resolution of 3 to further increase the number of clusters, thereby reducing the number of cells in each cluster, enhancing the similarity among cells within the same clusters, and consequently reducing the noise introduced by data augmentation. Therefore, scButterfly-C has the potential ability to balance the number of cells in each cluster compared to scButterfly-T and thereby reducing the differences of cells in each cluster.

Thirdly, to further reduce the additional noise introduced in data augmentation, we separately performed clustering on cells of each cell type and then conducted data

augmentation by randomly pairing two omics of the cells in the same cluster, resulting in a variant of scButterfly named scButterfly-TC (**T**ype **C**luster). Specifically, we partitioned all training samples by cell-type labels and trained a MultiVI[41] model with default settings for each cell type to obtain joint cell embeddings based on RNA and ATAC profiles. We then performed Leiden clustering to obtain refined clusters for each cell type. Finally, scButterfly-TC utilized these refined clustering labels to perform data augmentation, thus avoiding introducing too much additional noise when pairing two omics of the cells in the same cell types (scButterfly-T). We conducted five-fold cross-validations by cell on the BMMC, MB, CL, MCC, MK, MDS, and PBMC datasets to evaluate the performance of scButterfly-TC, the basic scButterfly model (scButterfly-B) and the other two variants (scButterfly-T and scButterfly-C). As shown in Supplementary Fig. 28, scButterfly-TC performed similarly to scButterfly-T in the translation from ATAC to RNA. However, scButterfly-TC performed slightly worse than scButterfly-T for the translation from RNA to ATAC, indicating that decreasing the additional noise may not lead to an improvement in performance. Considering that scButterfly-T is simpler and more user-friendly compared to scButterfly-TC, we still recommend that users perform cross-modality translation with scButterfly-T. Even so, we also provide the scButterfly-TC as an optional variant. Users could choose to use scButterfly-TC according to the situation of various datasets, especially for the datasets only with coarse labels.

Supplementary Text 5: Discussion of the impact of the number of augmented data on the performance

We discussed the impact of the number of augmented data on the model's performance. Illustrated with the five-fold cross-validation on the MCC dataset as an example, we evaluated the performance of applying two kinds of augmentation strategies on scButterfly-B with different counts of augmented data. We augmented the dataset to w times as large as the original dataset, and chose the settings of w ranging from 1.5 to 4.5 with a step of 0.5. For non-integer times of augmentation, we kept one copy of the original data, randomly shuffled the ATAC profiles for $\lfloor w-1 \rfloor$ times and paired with unshuffled RNA profiles, and for the decimal part $w-\lfloor w \rfloor$ (e.g., 0.5), we randomly selected the corresponding proportion $(w-\lfloor w \rfloor) \times 100\%$ (e.g., 50%) of the augmented samples. We presented the experimental results in Supplementary Fig. 29, marking our default setting (augmented the dataset to three times as the original dataset) as yellow. As w increased, the model's performance initially improved because more diverse samples were added to the training process. However, when w exceeded 3, the model's performance did not improve or even decline, possibly because the proportion of the original data was constantly diluted. In conclusion, we identified that augmenting the dataset to three times as large as the original sample size is a suitable and robust choice.

Supplementary Text 6: Statement of anti-noise capacity for scButterfly

Given that single-cell data typically exhibits high-level noise and extreme sparsity, both the anti-noise capacity and the robustness to data sparsity are essential for a computational method. To mimic protocols that generate sparser data, we took the MCC dataset as an example and randomly dropped out the non-zero entries in the profiles to be translated to zero with a probability equal to the dropout rate, which was set to range from 10% to 90%. We compared the performance of scButterfly-B and baseline methods to better test the effectiveness of the basic scButterfly framework without data augmentation. As shown in Supplementary Fig. 30, although JAMIE also achieved comparable performance on the translation from ATAC to RNA, scButterfly-B showcased remarkable performance at different levels of data sparsity and achieved much more stable performance, underscoring the superior robustness of scButterfly to data corruption.

Supplementary Text 7: Statement of robustness to the hyperparameters for scButterfly

scButterfly demonstrates the satisfactory robustness to the multiple hyperparameters, including the training epochs, patience of early stop and the loss weights. To be specific, we conducted the sensitivity analyses of the training epochs and early stop patience to give more details about convergence. As described in Section "The training procedure of scButterfly", we separately pretrained the encoders and decoders for different modalities with 100 epochs and integratively trained the model for 200 epochs, with 50 epochs patience of early-stop. We denoted the pretraining epochs for RNA and ATAC, the epochs for integrative training, and the patience as $w_{\text{pre-r}}$, $w_{\text{pre-a}}$, w_{integ}, w_{patience}, respectively. We generated 40 different hyperparameter settings by changing one parameter at a time. Specifically, we selected the range of $w_{\text{pre-r}}$ and $w_{\text{pre-a}}$ from 50 to 150 with step of 10, the range of w_{integ} from 100 to 300 with a step of 20, and the range of w_{patience} from 0 to 100 with a step of 10, based on the default settings of $(w_{\text{pre-r}}, w_{\text{pre-a}}, w_{\text{integ}}, w_{\text{patience}}) = (100, 100, 200, 50)$. We conducted the five-fold cross-validation on the MCC dataset to evaluate the translation performance of scButterfly-B with different hyperparameter settings. The results are shown in Supplementary Fig. 31, with the hyperparameter denoted like $(w_{\text{pre-r}}, w_{\text{pre-a}}, w_{\text{integ}}, w_{\text{patience}})$ and the default settings marked with yellow. scButterfly exhibited relative robustness to the epochs of pretraining and integrative training, implying that under different parameter settings about the training epochs, scButterfly had consistently excellent performance (Columns #1, #2, and #3 in Supplementary Fig. 31). It is worth noting that the model performance was bad when the patience parameter was set below 30, while the performance tended to be stable when the patience gradually increased to 50 (Column #4 in

Supplementary Fig. 31). This is intuitive and may be due to the fact that, when the patience is small, the model is more likely to stop training when it can't improve in a small number of epochs, resulting in insufficient training of the model. Therefore, we think the default setting of patience is reasonable.

We further conducted sensitivity analyses of four hyperparameters in loss calculation, including w_{r}, w_{a}, the numerator of w_{ELBO} denoted as w_{e} ($w_{\mathrm{ELBO}} = \frac{w_{\mathrm{e}}}{\mathrm{Input\ dimensions}}$), and w_{dis}. Here, w_{r} and w_{a} are the weights of reconstruction/translation loss for ATAC and RNA respectively. w_{ELBO} is the weight of ELBO loss and w_{dis} is the weight of discriminator loss. According to the default settings $(w_{\mathrm{r}}, w_{\mathrm{a}}, w_{\mathrm{e}}, w_{\mathrm{dis}}) = (1, 2, 20, 1)$, we selected the range of w_{r} from 0.5 to 1.5 with a step of 0.1, the range of w_{a} from 1.5 to 2.5 with a step of 0.1, the range of w_{e} from 15 to 25 with a step of 1, and the range of w_{dis} from 0.5 to 1.5 with a step of 0.1. By changing one parameter at a time, we generated a total of 40 different hyperparameter settings. Take the five-fold cross-validation on the MCC dataset as an example, we evaluated the translation performance of the scButterfly-B with these hyperparameter settings based on clustering metrics. We denoted the settings of hyperparameters with the format $(w_{\mathrm{r}}, w_{\mathrm{a}}, w_{\mathrm{e}}, w_{\mathrm{dis}})$, marked the default settings with yellow. As shown in Supplementary Figs. 32, 33, with the variation of different hyperparameters settings, the clustering metrics remained stable, demonstrating that scButterfly is relatively robust to these hyperparameters and achieved consistent performance.

Supplementary Text 8: Statement and evaluation of the numerical accuracy for scButterfly

To evaluate the cross-modality translation performance of scButterfly form the perspective of numerical accuracy, we additionally evaluated the translated profiles by various correlation metrics. For the RNA data, we computed the average per-cell Pearson correlation coefficients and the average per-cell Spearman correlation coefficients, which are widely recognized indicators used to assess the performance of cross-modality translation in previous studies[42, 43]. To be specific, for each cell, we separately calculated the Pearson correlation coefficient and Spearman correlation coefficient between the original profiles and the translated profiles. We utilized the average of the correlation coefficients of all cells as the metric to evaluate the translation performance of the scButterfly and other baseline methods. However, the correlation coefficients appear to be sensitive to high noise and sparsity data[25]. Moreover, they are non-robust to outliers[27] and inaccurate for high-dimensional data[28].

For the ATAC data, given its binary feature and extreme sparsity, we serve the translation task as the binary classification problem. We considered utilizing the auROC (the area under the ROC curve) as a measure of translation performance, which was also widely mentioned in previous studies about cross-modality translation[42, 44]. We calculated the auROC of the translated profiles for each cell and took the average of auROCs of all cells as the metrics. Additionally, deriving from the auPRC (area under the precision-recall curve), we have further considered auPRCnorm for each cell and served the average auPRCnorm of each cell as the metric to evaluate models' performance on ATAC data, as suggested in recent research[44]. auPRCnorm is normalized to correct for the skew in auPRC measurement since ATAC data is

usually extremely sparse. The auPRCnorm for each cell could be calculated as follows:

$$\text{auPRCnorm} = \frac{(\text{AUPR} - \text{PP})}{(1 - \text{PP})}, \text{PP} = \frac{\#(\text{peaks expressed for cell})}{\#\text{peaks}}$$

We performed five-fold cross-validation on the seven paired datasets (BMMC, MB, CL, MCC, MK, MDS, and PBMC) and evaluated the cross-modality translation performance based on the aforementioned four metrics. As shown in Supplementary Fig. 34, scButterfly outperformed other baseline methods in terms of Spearman correlation coefficients and auROC. For the Pearson correlation coefficients and auPRCnorm, although BABEL also performed comparably, scButterfly showcased competitive performance compared to other baseline methods. Overall, we identified that scButterfly also demonstrates promising translation performance in terms of numerical accuracy.

Supplementary Text 9: Discussion of the batch correction step before generate pseudo-paired samples for unpaired data training

For the unpaired data training, we directly paired the data from different batches and different omics. We have considered whether to take batch effects into account when designing the scButterfly framework for unpaired data. However, we ultimately abandoned this consideration based on the following reasons:

Firstly, although batch effects can be indeed observed in downstream analysis, we believe that batch effects have a limited impact on our data augmentation strategy. The inter-batch heterogeneities are often smaller compared to the inter-cell-type heterogeneities, especially in the datasets with batch effects attributed to media storage or patients[45]. Taking the UP_MPMC dataset as an example, we visualized the original RNA and ATAC profiles of the cells in the first fold of the five-fold cross-validation with t-SNE. As shown in Columns #1 and #3 in Supplementary Fig. 35a, batch effects could cause poor mixing or even separation of cells within a part of cell types, especially for the original RNA data. However, most of the cells of the same cell type tend to cluster together in the t-SNE visualizations. These results indicate that the variants from batch effects are usually smaller than the variants from the cell type.

Secondly, we observed that scButterfly for unpaired data translation was generally less affected by batch effects to some extent. In Section "scButterfly can be generalized to unpaired data training and perturbational analysis" of the manuscript, we designed a method similar to the scButterfly-T variant for translating the unpaired data, which matches cells of the same type to generate artificial paired training samples. For other baseline methods, we performed the same matching to make cross-modality translations. It is worth noting that we did not require

the matched samples to come from the same batch, since in the unpaired datasets, batches of one modality data often have no overlap with that of another modality. Taking the UP_MPMC dataset as an illustration again, the RNA data contains 4 batches, while the ATAC data contains different 9 batches as shown in Columns #1 and #3 in Supplementary Fig. 35a. Therefore, the scButterfly model was trained to translate samples from different batches, which has given the model the potential ability to be not significantly affected by batch effects. The experiment results in Supplementary Fig. 35a also support this point. In the original ATAC data (Column #1 in Supplementary Fig. 35a), some samples of Batch #3 exhibit variations compared to other samples (marked with red boxes), while in translated profiles from ATAC to RNA (Column #2 in Supplementary Fig. 35a), all batches are well mixed, indicating that scButterfly could be insignificantly impacted by the batch effects. For the more challenging original RNA data (Column #3 in Supplementary Fig. 35a), where batch effects are more pronounced, RNA to ATAC translation (Column #4 in Supplementary Fig. 35a) also mixes samples from different batches, implying the potential ability of the model to confront batch effects.

Thirdly, the existing batch correction methods may have some limitations[4, 5]: most existing methods are likely to simultaneously lose some biological heterogeneity when correcting the batch effects, presenting the trade-off between the preservation of biological variation and the mixing of batches. Moreover, most batch correction methods perform dimensionality reduction on data and output low-dimensional embeddings instead of the count matrixes, resulting in the inability to use the original dimensionality data for downstream analysis. To further characterize the impact of batch correction, we added a batch correction step before matching the cells with the same cell type. As suggested in recent research[5], scGen[16] and pyComBat[17]

could achieve the best overall performance among the batch correction methods which could output the corrected original-dimensionality count matrices for RNA and ATAC, respectively. We chose these two state-of-the-art batch correction methods to respectively correct the batch effects in the RNA and ATAC data before data augmentation. As shown in Supplementary Fig. 35b, the combination of scGen and pyComBat (scButterfly-T-with-batch-correction) led to a decrease in translation performance, revealing that the batch correction before data augmentation may not be suitable for unpaired data translation.

Supplementary Text 10: Discussion of distinguish the foreground and background signals in ADT data

The background signal is a kind of noise that widely appears in many recently introduced droplet-based single-cell assays[15], including CITE-seq[46], Perturb-seq[47], scCAT-seq[48], SNARE-seq[49], SHARE-seq[50], and 10x Multiome. For ADT data, the background signal appears to be a major component of noise since ADT data usually contains non-sparse positive counts, posing a challenging problem[51]. Recent research could effectively remove background noise by accurately estimating the background signal via the level of "ambient" ADT counts in empty droplets. However, methods without using strike-in control cells still mainly use a two-component mixture model to separately estimate the foreground and background signals[52-54].

To evaluate the impact of distinguishing the foreground and background signals in ADT data, we developed a variant of scButterfly-B namely scButterfly-B-ADT-BG, based on the two-component Gaussian mixture model similar to totalVI[54]. To be specific, we substituted the translator T in the scButterfly-B model with another translator T^{BG}, which considered background signals (Supplementary Fig. 36). T^{BG} in latent space facilitated the translation between different modalities and the end-to-end mapping within each of the individual modalities. Illustrated with the ADT-to-ADT mapping and the ADT-to-RNA translation as an example, we first mapped the input $\mathrm{En}_{\mathrm{a}}(\mathbf{X}_{\mathrm{a}})$ from the ADT encoder into a 128-dimensional mean vector $\mathbf{X}^{\mathrm{a}}_{\mathrm{mean}}$ and log-variance vector $\mathbf{X}^{\mathrm{a}}_{\mathrm{var}}$ with two blocks of fully connected layer and LeakyReLU, respectively. Here, $\mathbf{X}_{\mathrm{a}}$ represented the ADT input of the scButterfly model and En_{a} was the ADT encoder. Then, the translator T^{BG} obtained the latent embedding by sampling from the multivariate Gaussian distribution as follows:

$$\mathbf{X}_{\text{embed}} = \mathbf{X}_{\text{mean}}^{\text{a}} + e^{\frac{\mathbf{X}_{\text{var}}^{\text{a}}}{2}} \times \mathbf{epseps}{\sim}\mathcal{N}(\mathbf{0},\ \mathbf{I}). \quad (1)$$

For the ADT to RNA translation, we ultimately generated a translated RNA embedding $\mathrm{T}_{\mathrm{a}\to\mathrm{r}}^{\mathrm{BG}}\left(\mathrm{En}_{\mathrm{a}}(\mathbf{X}_{\mathrm{a}})\right)$ with one block of a fully connected layer and LeakyReLU. For the ADT to ADT mapping, we generated a mapped ADT embedding $\mathrm{T}_{\mathrm{a}\to\mathrm{a}}^{\mathrm{BG}}\left(\mathrm{En}_{\mathrm{a}}(\mathbf{X}_{\mathrm{a}})\right)$ with one background correction block from $\mathbf{X}_{\text{embed}}$. In the background correction block, we first got two matrices $\boldsymbol{\pi}$ and $\boldsymbol{\alpha}$ from $\mathbf{X}_{\text{embed}}$ with two blocks of the fully connected layer with activations of Sigmoid and $\mathrm{Exp} + 1$, respectively. $\boldsymbol{\pi}$ could be interpreted as the probability that any cell-feature pair activated due to the background alone, while $\boldsymbol{\alpha}$ denoted the scale factor for the foreground compared to the background for any cell-feature pair. We assumed that the background embeddings $\boldsymbol{\beta}$ follow a multivariate Gaussian distribution with a mean of $\boldsymbol{\beta}_{\text{mean}}$ and log-variance $\boldsymbol{\beta}_{\text{var}}$, where $\boldsymbol{\beta}_{\text{mean}}$ and $\boldsymbol{\beta}_{\text{var}}$ are two learnable vectors. Then we could model the corrected latent embedding $\mathbf{X}_{\text{embed}}^{\text{corrected}}$ as a two-component Gaussian mixture model as follows:

$$\begin{cases} \mathbf{v} \sim \mathrm{Bernoulli}(\boldsymbol{\pi}) \\ \boldsymbol{\beta} \sim \mathcal{N}(\boldsymbol{\beta}_{\text{mean}}, \boldsymbol{\beta}_{\text{var}}) \\ \mathbf{X}_{\text{embed}}^{\text{corrected}} = \mathbf{v}\boldsymbol{\beta} + (1 - \mathbf{v})\boldsymbol{\alpha}\boldsymbol{\beta} \end{cases}. \quad (2)$$

At last, we generated a mapped ADT embedding $\mathrm{T}_{\mathrm{a}\to\mathrm{a}}^{\mathrm{BG}}\left(\mathrm{En}_{\mathrm{a}}(\mathbf{X}_{\mathrm{a}})\right)$ with one fully connected layer and LeakyReLU from $\mathbf{X}_{\text{embed}}^{\text{corrected}}$, and used it as the input of the ADT decoder. Analogously, we used another two blocks of fully connected layer and LeakyReLU to map the input $\mathrm{En}_{\mathrm{r}}(\mathbf{X}_{\mathrm{r}})$ from the RNA encoder into a 128-dimensional mean vector $\mathbf{X}_{\text{mean}}^{\text{r}}$ and log-variance vector $\mathbf{X}_{\text{var}}^{\text{r}}$, respectively. For the RNA to RNA mapping, we generated the $\mathrm{T}_{\mathrm{r}\to\mathrm{r}}^{\mathrm{BG}}\left(\mathrm{En}_{\mathrm{r}}(\mathbf{X}_{\mathrm{r}})\right)$ using the same fully connected block of ADT to RNA translation. For the RNA to ADT, we used the same background correction block as ADT to ADT mapping to obtain

$\mathrm{T}_{\mathrm{r}\to\mathrm{a}}^{\mathrm{BG}}\left(\mathrm{En}_{\mathrm{r}}(\mathbf{X}_{\mathrm{r}})\right)$. When performing translation for testing data, we used the formula $\mathbf{X}_{\mathrm{embed}}^{\mathrm{corrected}} = (1-\mathbf{v})\boldsymbol{\alpha}\boldsymbol{\beta}$ to generate the corrected embedding $\mathbf{X}_{\mathrm{embed}}^{\mathrm{corrected}}$, to remove the influence of background.

To evaluate the performance of scButterfly-B-ADT-BG, we performed cross-validation by batch on the CITE_BM and CITE_BMMC datasets. We also considered the influence of background signals of ADT data on the scButterfly-C and scButterfly-T variants, which applied the data augmentation strategies based on the cluster labels and cell types respectively, resulting in scButterfly-C-ADT-BG and scButterfly-T-ADT-BG. We illustrated the final benchmarking results in Supplementary Fig. 37. Taking the CITE_BM dataset as an example (Supplementary Fig. 37a), scButterfly-B-ADT-BG performed slightly better than scButterfly-B, mainly on the adjusted Rand index (ARI) and Pearson correlation coefficient for RNA to ADT translation. We also observed similar improvements of scButterfly-C-ADT-BG and scButterfly-T-ADT-BG, compared to scButterfly-C and scButterfly-T, respectively. However, the strategy of considering background signals of ADT data will not lead to an obvious improvement of scButterfly on every dataset. For example, scButterfly-B-ADT-BG had no significant advantages compared to scButterfly-B on the CITE_BMMC dataset (Supplementary Fig. 37b). The same situation was also observed in the comparisons between scButterfly-C-ADT-BG and scButterfly-C, as well as scButterfly-T-ADT-BG and scButterfly-T. One-sided paired Wilcoxon signed-rank tests showed that distinguishing the background and foreground signal could only provide a limited advantage on the clustering metrics for the translation from RNA to ADT on these two datasets (Supplementary Fig. 37c). The translation performance of scButterfly considering background signals did not have a significant

advantage over that without considering background under the significance level 0.1, which indicated that the consideration of background signals is an optional but not necessary choice for cross-modality translation.

Supplementary Text 11: Comparation between the augmented samples by different scButterfly variants and the experimental paired samples

We compared the differences between the artificial samples generated by randomly pairing and experimentally paired original data. Illustrating with the SNARE-seq[49] profiled MCC dataset as an example, we obtained the joint cell embeddings of augmented and experimental multi-omics profiles via MultiVI[41] and visualized the embeddings with t-SNE[55]. As shown in Supplementary Fig. 38, the samples generated by randomly pairing (Column #2 in Supplementary Fig. 38) do not exhibit significant discrepancies compared to the experimentally paired original samples (Column #3 in Supplementary Fig. 38) in t-SNE visualizations. Furthermore, we observed that the augmented data can better distinguish different cell types in the visualizations compared to the original data, especially for scButterfly-T and scButetrfly-TC. For example, as shown in Supplementary Fig. 38, the augmented dataset could distinguish the InN, InP, InS, and InV (marked with red boxes), while the original dataset mixed them in the visualizations. Additionally, we found that there are difference between the dataset augmented with different strategies. Specifically, scButterfly-C augments data by randomly pairing the profiles of different modalities within the same cluster, resulting in the samples seeming to be tightly distributed into many clusters in the visualizations. scButterfly-T augments data by randomly pairing the profiles of different modalities of the same cell type, causing the samples of the same cell type to cluster together in the visualizations, while the samples of different cell types loosely distribute. scButterfly-TC augments data with the refined clusters within each cell type, leading to some cell types with more cells further distinguished into smaller parts in the visualizations, which aligns with

expectations. In conclusion, we observed that the generated samples do not exhibit significant differences from the experimental samples in visualizations and the patterns of the augmented datasets are related to the strategies of data augmentation.

Supplementary Text 12: Discussion of data augmentation with the anchors detected by CCA-based method

We incorporated the canonical correlation analysis (CCA)-based method, namely Seurat[10], to detect anchors between two modalities and utilized them for data augmentation, resulting in a new variant of scButterfly named scButterfly-A (**A**nchor). For scButterfly-A, we first treated the RNA and ATAC training sets as the reference and query datasets, respectively, and obtained the CCA embeddings and the anchor pairs between the two modalities via Seurat. Subsequently, for the RNA profile of each cell, we identified the nearest RNA anchor under the Euclidean distance on the RNA CCA embeddings. We then paired this RNA profile with the ATAC profile of the ATAC anchor associated with the nearest RNA anchor to generate artificial samples. Since a single RNA anchor may exist in multiple anchor pairs with different ATAC anchors, we only selected the anchor pairs with the highest shared neighbor overlap score for data augmentation. After the above steps, we obtained a generated dataset of the same size as the original training set. Similarly, for the ATAC profile of each cell, we found the nearest ATAC anchor and paired it with the RNA profile of the corresponding RNA anchor with the highest score, resulting in a same number of artificial samples as the original training set. By merging these generated samples with the original data, we finally obtained an augmented dataset three times the size of the original data, which was used for model training.

To investigate the influence of the cell type-specific anchors, we further proposed the variant scButterfly-TA (**T**ype **A**nchor). We first performed Seurat for the multi-omics profiles of cells of each cell type to obtain cell type-specific CCA embeddings and anchors. Subsequently, for each cell type, similar to scButterfly-A, we identified the nearest RNA anchor for the RNA

profiles of each cell and paired the RNA profiles with the ATAC profile of the highest-scoring ATAC anchor corresponding to the nearest RNA anchor. We also paired each ATAC profile with the RNA profile of the highest-scoring RNA anchor related to the nearest ATAC anchor. These artificial samples were mixed with the original training samples, resulting in a training dataset that is three times as large as the original dataset for model training.

To evaluate the effectiveness of these two variants, we conducted five-fold cross-validation on the MCC dataset to compare the performance of scButterfly-A, and scButterfly-TA with the basic scButterfly model (scButterfly-B) and the other two variants (scButterfly-C and scButterfly-T). As shown in Supplementary Fig. 39, scButterfly-A struggled to achieve satisfactory cross-modality translation performance. The poor performance of scButterfly-A may be attributed to the anchors detected by Seurat not accurately linking the profiles of the same cell type. For example, for the cells in the first test fold, only 13.080% anchor pairs (203/1552) contain the RNA and ATAC anchors with the same cell type. These augmented training data may mislead the model and harm the cross-modality translation performance. This result suggests that directly using the detected anchors for data augmentation may not be suitable. For scButterfly-TA, it did not show a significant advantage compared to scButterfly-T (p-value=0.965) under one-side Wilcoxon signed-rank test. Considering that scButterfly-T is simpler and more user-friendly compared to scButterfly-TA, we still recommend that users perform cross-modality translation with scButterfly-T. We hope the reviewer can agree with us that incorporating the CCA-detected anchors may not lead to significant improvement in the translation performance.

Supplementary Text 13: Discussion of the reusing for stimulated cells in perturbation-response prediction with scButterfly

In Section "Single-cell perturbation-response prediction with scButterfly", we applied the optimal transport to match stimulated cells with control cells to generate paired training samples. We computed an optimal transport matrix for each cell type and matched each control cell with the highest-weighted stimulated cell, even if that stimulated cell had already been assigned to another control cell. To address the concern about reusing for stimulated cells, we would like to first clarify that scButterfly may reuse the same cell multiple times, but this occurs solely when the count of the control cell is significantly larger than the count of the stimulated cell. For example, we counted the amount of reusing for each cell type on the PT_PBMC dataset. As shown in Supplementary Fig. 40a, the results illustrate that a large number of reusing for stimulated cells only occurs in cell types of CD14+Mono and Dendritic, due to the over-numbering of control cells compared to stimulated cells. Besides, we further considered an alternative matching approach without reusing stimulated cells. Specifically, for a cell type k, we initially computed the optimal transport matrix $\boldsymbol{\gamma}_k$ as introduced in Section "Single-cell perturbation-response prediction with scButterfly". Subsequently, we formulated the problem as an assignment problem and selected an assignment method that maximizes the total weight. Mathematically, the problem can be expressed as:

$$\mathbf{A}_k = \underset{\mathbf{A}}{\operatorname{argmax}} < \mathbf{A}, \boldsymbol{\gamma}_k >_{\mathrm{F}}$$
$$s.t. \begin{cases} \sum_{j=1}^{n_k^{\mathrm{sti}}} \mathbf{A}[i][j] = 1, \ \ \forall i \\ \sum_{i=1}^{n_k^{\mathrm{ctr}}} \mathbf{A}[i][j] = 1, \ \ \forall j \\ \mathbf{A}[i][j] \in \{\mathbf{0}, \mathbf{1}\}, \ \ \forall i, j \end{cases} \tag{3}$$

where $\mathbf{A}_k$ represents the best assignment for control and stimulated data of type k and $< \mathbf{A}, \boldsymbol{\gamma}_k >_{\mathrm{F}}$ is the Frobenius inner product. Finally, we paired the profile of control cell i with the profile of stimulated cell j if $\mathbf{A}_k[i][j]$ equals to 1, and generated the paired sample for the training of scButterfly, denoted as scButterfly-T-no-repeat.

We evaluated the performance of scButterfly-T-no-repeat for the challenging out-of-sample prediction on the PT_PBMC dataset. As shown in Supplementary Fig. 40b, there was no significant difference between scButterfly-T-no-repeat and scButterfly-T, suggesting that reusing had no negative impact on the performance of scButterfly on the datasets used in our experiment.

Supplementary Text 14: Discussion of impact of different embedding dimensions on translation performance in CITE-seq data analyses

we discussed the impact of different embedding dimensions on translation performance in CITE-seq data analyses. Firstly, ADT data often have very low dimensions, such as the CITE_BM dataset, which only contains 25 antibodies. However, scButterfly needs to map the RNA data and ADT data into the embedding spaces with the same dimensions to realize cross-modality translations. Since RNA data in CITE-seq usually contains dimensions much higher than ADT data (for example, the CITE_BM dataset contains 17,009 genes but only 25 antibodies), simultaneously projecting both RNA and ADT data to the same dimensions lower than the original ADT data may lead to a massive loss of information for RNA data. Therefore, we chose a trade-off dimension 128 between the high dimension of RNA and the low dimension of ADT data. Secondly, as suggested in recent research[56], the principle of choosing a proper embedding dimension should be small enough to be efficient and large enough to be effective. Therefore, we further investigated the translation performance using different embedding dimensions. As an example for illustration, we performed five-fold cross-validation on the CITE_BM dataset using scButterfly-B with embedding dimensions of 8, 16, 32, 64, 128, 256, and 512. As shown in Supplementary Fig. 41a, the translation performance improved as the embedding dimensions increased and reached a relatively stable level at 128 dimensions, especially on the scores of the adjusted mutual information (AMI), homogeneity (HOM), and normalized mutual information (NMI). We also conducted one-sided paired Wilcoxon signed-rank tests between the adjacent choices of embedding dimensions (Supplementary Fig. 41b), including 8 vs 16, 16 vs 32, 32 vs 64, 64 vs 128, 128 vs 256, and 256 vs 512. The *p*-values

indicate that, at the significance level of 0.05, the translation performance significantly increases with the increase of dimensions below 128. However, embedding dimensions greater than 128 no longer provide significant improvements, which implies that using 128 as the embedding dimension provides a balance between efficiency and effectiveness.

Supplementary Text 15: Details of evaluation metrics

For the quantitative evaluation of the translated profiles for preserving cell heterogeneity, we assessed the clustering results with four metrics: adjusted Rand index (ARI), adjusted mutual information (AMI), normalized mutual information (NMI), and homogeneity (HOM). Assuming that there are translated profiles consisting of N cells with cell types $\mathbf{T} = \{T_1, \dots\dots, T_n\}$, alongside cluster labels $\mathbf{P} = \{P_1, \dots\dots, P_m\}$ obtained with the Leiden[40] algorithm (Methods). a_i and b_j separately stand for the number of cells with labels T_i and P_j, while n_{ij} is the cell count for the overlaps of T_i and P_j. ARI takes chance agreement into consideration and is adjusted based on Rand index (RI), with the following formula:

$$ARI = \frac{\sum_{i,j}\binom{n_{ij}}{2} - \left[\sum_i \binom{a_i}{2} \sum_j \binom{b_j}{2}\right] / \binom{N}{2}}{\frac{1}{2}\left[\sum_i \binom{a_i}{2} + \sum_j \binom{b_j}{2}\right] - \left[\sum_i \binom{a_i}{2} \sum_j \binom{b_j}{2}\right] / \binom{N}{2}}. \quad (4)$$

NMI is a normalized variant of mutual information (MI), which could be represented as:

$$\mathrm{NMI} = \frac{\mathrm{MI}(\mathbf{P}, \mathbf{T})}{\sqrt{\mathrm{H}(\mathbf{P})\mathrm{H}(\mathbf{T})}}, \quad (5)$$

where $\mathrm{MI}(\cdot)$ is the mutual information and $\mathrm{H}(\cdot)$ represents the entropy. AMI further considers the chance agreement and is written as:

$$\mathrm{AMI} = \frac{\mathrm{MI}(\mathbf{P}, \mathbf{T}) - \mathrm{E}[\mathrm{MI}(\mathbf{P}, \mathbf{T})]}{\frac{1}{2}\big(\mathrm{H}(\mathbf{P}) + \mathrm{H}(\mathbf{T})\big) - \mathrm{E}[\mathrm{MI}(\mathbf{P}, \mathbf{T})]}. \quad (6)$$

HOM provides a reference for the purity of cell types within each cluster, calculated as follows:

$$\mathrm{HOM} = 1 - \frac{\mathrm{H}(\mathbf{T}|\mathbf{P})}{\mathrm{H}(\mathbf{T})}, \quad (7)$$

where $\mathrm{H}(\mathbf{X}|\mathbf{Y})$ means the conditioned entropy of $\mathbf{X}$ under the condition that $\mathbf{Y}$ is given.

For the evaluation of cell type annotation, we evaluated the performance with four metrics: Accuracy (Acc), Cohen's kappa value (Kappa), F1-macro, and F1-weighted. Assuming that there are N cells with cell types denoted as $\mathbf{T} = \{T_1, \dots\dots, T_n\}$, a_k and b_k separately represent the counts of cells with ground-truth cell-type label T_k and annotated label T_k. Denote the count of cells corrected-classified as t, Acc directly reflects the classification accuracy as follows:

$$\mathrm{Acc} = \frac{t}{N}. \tag{8}$$

Kappa further considers the chance agreement with the following formula:

$$\mathrm{Kappa} = \frac{\mathrm{Acc} - p_\mathrm{e}}{1 - p_\mathrm{e}}, \tag{9}$$

where $p_\mathrm{e} = \frac{1}{N^2}\sum_{k=1}^{n} a_k b_k$ is the hypothetical probability of chance variable. F1-macro and F1-weighted are derived from F1-score:

$$\mathrm{F1} = 2 \times \frac{\mathrm{precision} \times \mathrm{recall}}{\mathrm{precision} + \mathrm{recall}}, \tag{10}$$

where $\mathrm{precision}$ is the number of samples correctly labeled with T_k divided by b_k, while recall is the number of samples correctly labeled with T_k divided by a_k. Then we have the following F1-macro and F1-weighted:

$$\begin{gathered} \mathrm{F1} - \mathrm{macro} = \frac{1}{n}\sum_{k=1}^{n} \mathrm{F1}(k) \\ \mathrm{F1} - \mathrm{weighted} = \frac{1}{N}\sum_{k=1}^{n} a_k \mathrm{F1}(k), \end{gathered} \tag{11}$$

where $\mathrm{F1}(k)$ represents the F1-score when the label T_k is seen as a positive label.

For the evaluation of single-cell perturbation-response prediction, we mainly focused on the number of common differentially expressed genes (DEGs) of the top 100 real DEGs versus the

top 100 predicted DEGs, and the squared Pearson correlation (R^2) for mean gene expression of the top 100 real DEGs. We first performed the one-sided Wilcoxon rank-sum test in Scanpy[57] to separately identify the real DEGs between the control data and the real stimulated data, and the predicted DEGs between the control data and the predicted stimulated data. Then we counted the number of common DEGs of the top 100 real DEGs versus the top 100 predicted DEGs as a reference for the capability of preserving the biological variance. Further, we calculated the R^2 to assess the numerical consistency between the real perturbation responses and the predicted responses. We randomly sampled 80% of the test data with replacement 100 times, and for each time, we computed the mean expression of top 100 real DEGs for predicted and real stimulated data. R^2 was calculated as the mean of squared Pearson correlation between the real and predicted mean expression vector among these 100 times.

For the evaluation of the translation between transcriptome and proteome profiles, we investigated the average Pearson and Spearman correlation coefficients for each cell. Pearson and Spearman correlation coefficients evaluate the performance of translation from a numerical accuracy standpoint, given by the following formulas:

$$\text{Pearson index} = \frac{1}{N}\sum_{i=1}^{N}\text{Pearson}\left(\mathbf{X}_{\mathrm{a}}[i], \mathbf{X}_{\mathrm{r}\to\mathrm{a}}^{\mathrm{pred}}[i]\right)$$

$$\text{Spearman index} = \frac{1}{N}\sum_{i=1}^{N}\text{Spearman}\left(\mathbf{X}_{\mathrm{a}}[i], \mathbf{X}_{\mathrm{r}\to\mathrm{a}}^{\mathrm{pred}}[i]\right), \quad (12)$$

where $\mathbf{X}_{\mathrm{a}}[i]$ and $\mathbf{X}_{\mathrm{r}\to\mathrm{a}}^{\mathrm{pred}}[i]$ represent the real and predicted protein expression level of cell i, respectively, and N represents the total count for test cells.

Supplementary Figures

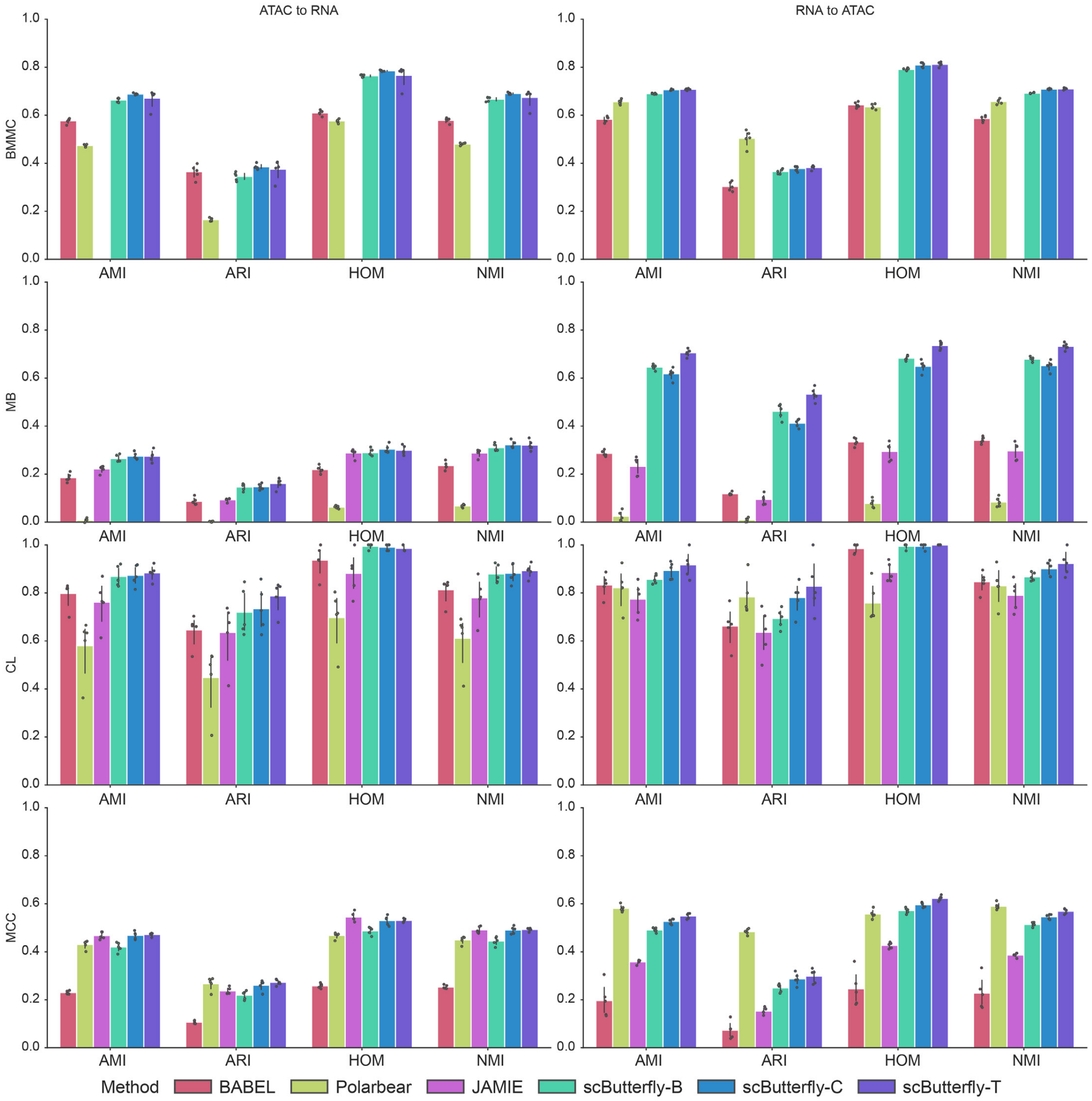

Supplementary Figure 1. Quantitative evaluation of the translation performance between chromatin and transcriptome profiles in five-fold cross-validation by cell on the four datasets (BMMC, MB, CL, and MCC) ($n = 5$ cross-validations for each dataset), via cell clustering with metrics of AMI, ARI, HOM and NMI. The height of each bar denotes the median value of each metric and the error bars show 95% confidence interval. Note that JAMIE encountered GPU memory errors on the BMMC dataset.

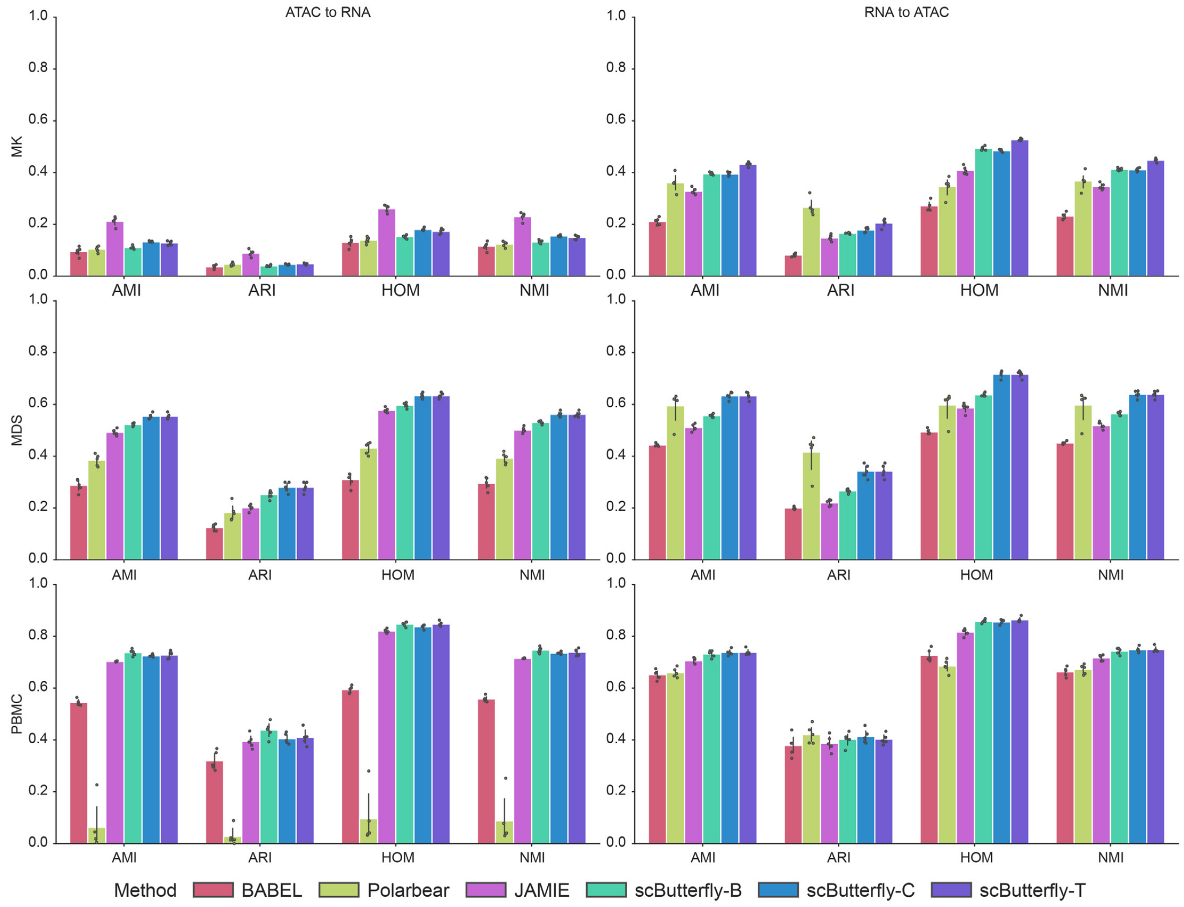

Supplementary Figure 2. Quantitative evaluation of the translation performance between chromatin and transcriptome profiles in five-fold cross-validation by cell on the three datasets (MK, MDS, and PBMC) (n = 5 cross-validations for each dataset), via cell clustering with metrics of AMI, ARI, HOM and NMI. The height of each bar denotes the median value of each metric and the error bars show 95% confidence interval.

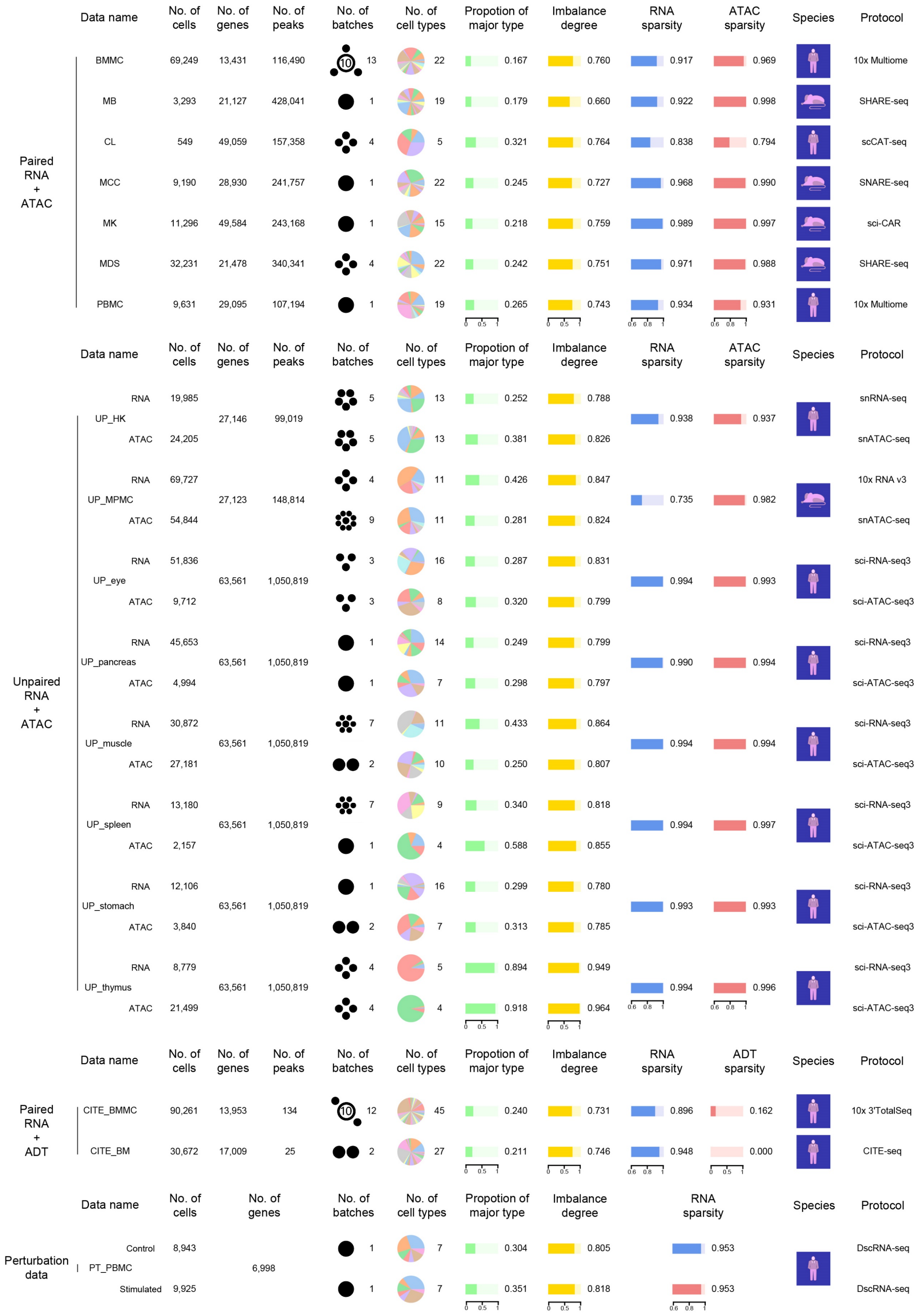

Supplementary Figure 3. A summary of the datasets for evaluating the performance of scButterfly model, including the detailed information about the number of cells, features, batches and cell types, as well as the imbalance of cell types, sparsity, protocol and species.

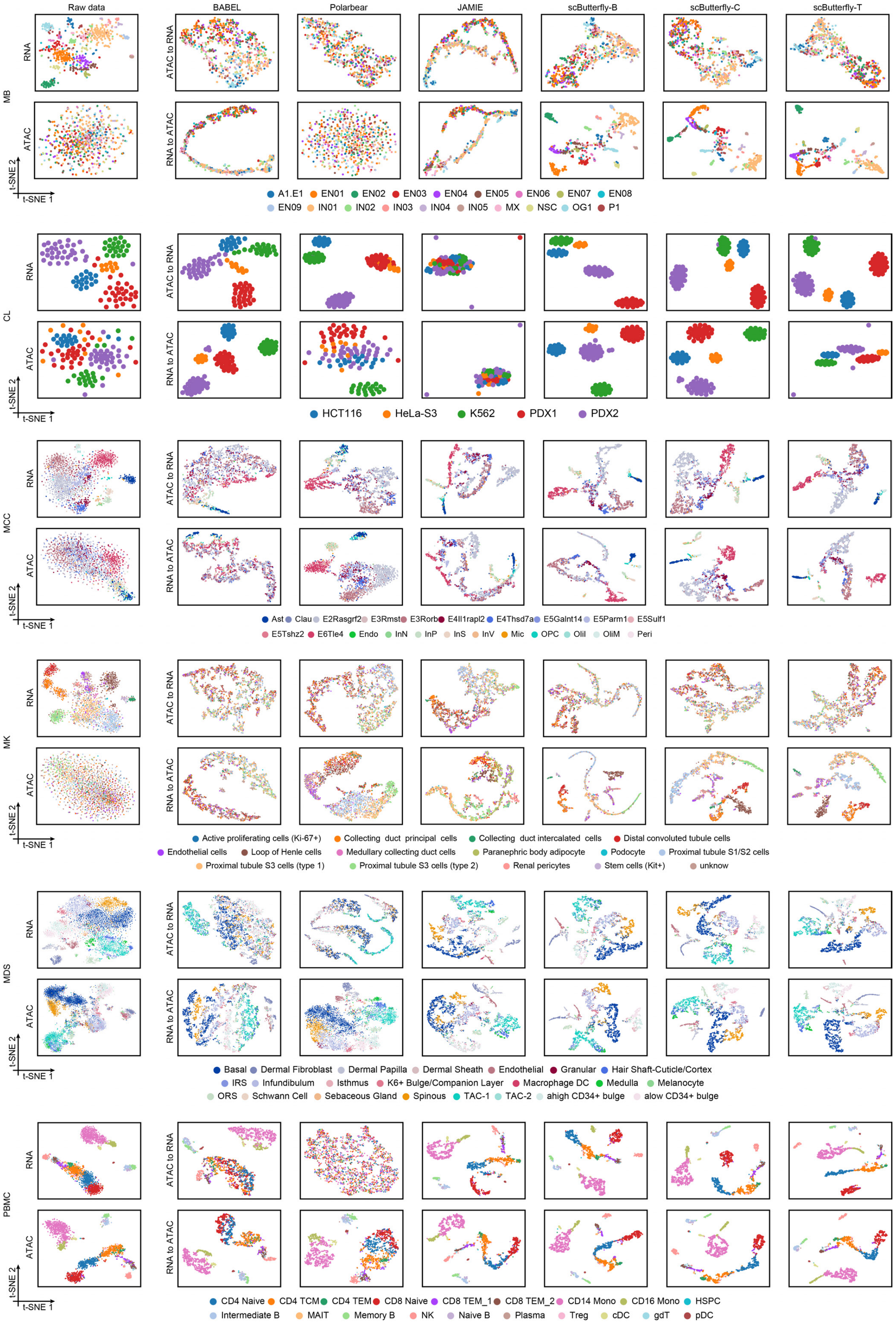

Supplementary Figure 4. t-SNE visualization of cells in the first test fold of five-fold cross-validation by cell on the six datasets (MB, CL, MCC, MK, MDS, and PBMC), using the raw profiles and the profiles translated by different methods.

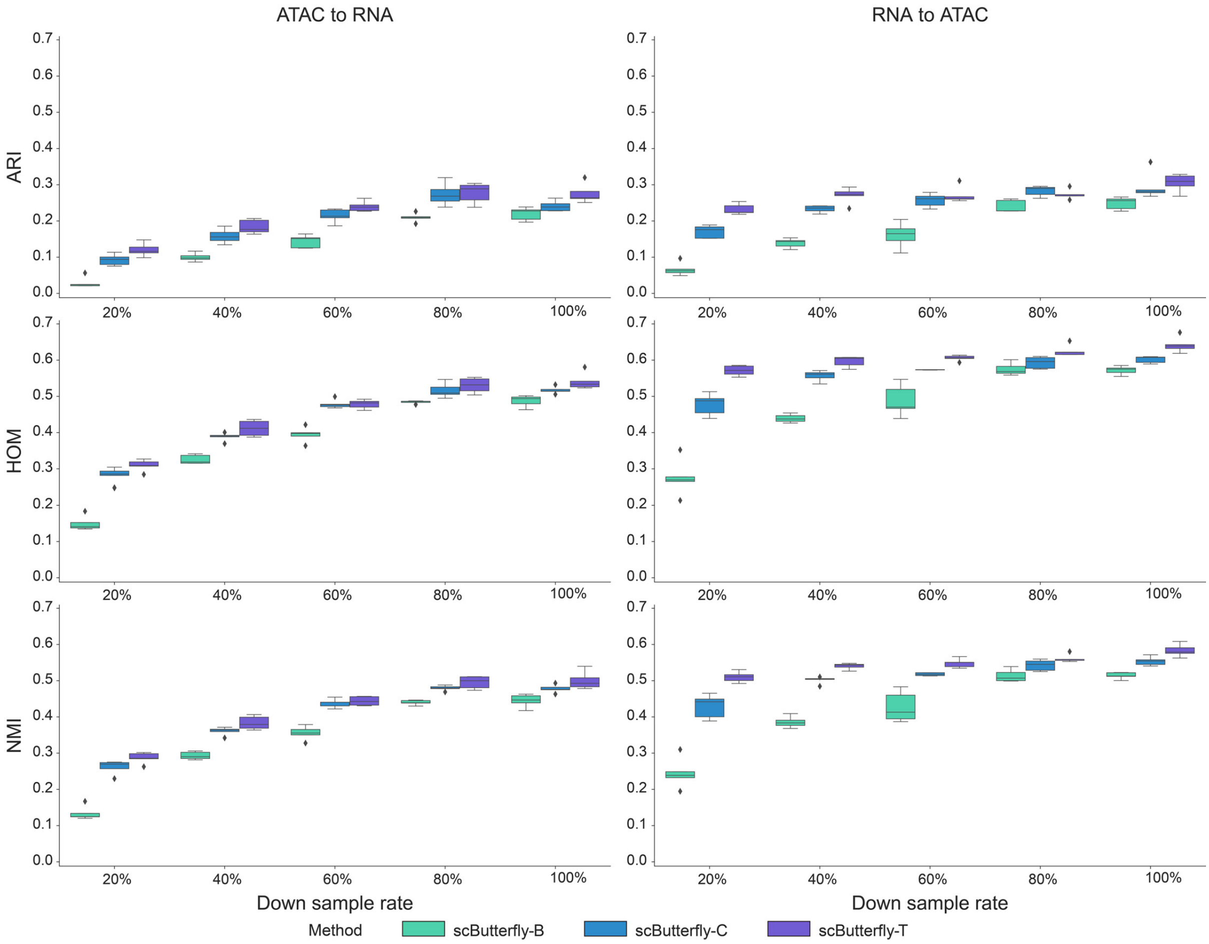

Supplementary Figure 5. Evaluation of the cross-modality translation performance for data with different rate of random down sampled, including five-fold cross-validation on the MCC dataset (*n* = 5 cross-validations on 9190 cells), via cell clustering with metrics of ARI, HOM and NMI. In the boxplots, the center lines, box limits, whiskers and notches denote the median, upper and lower quartiles, 1.5× interquartile range and 95% confidence interval calculated using a Gaussian-based asymptotic approximation, respectively.

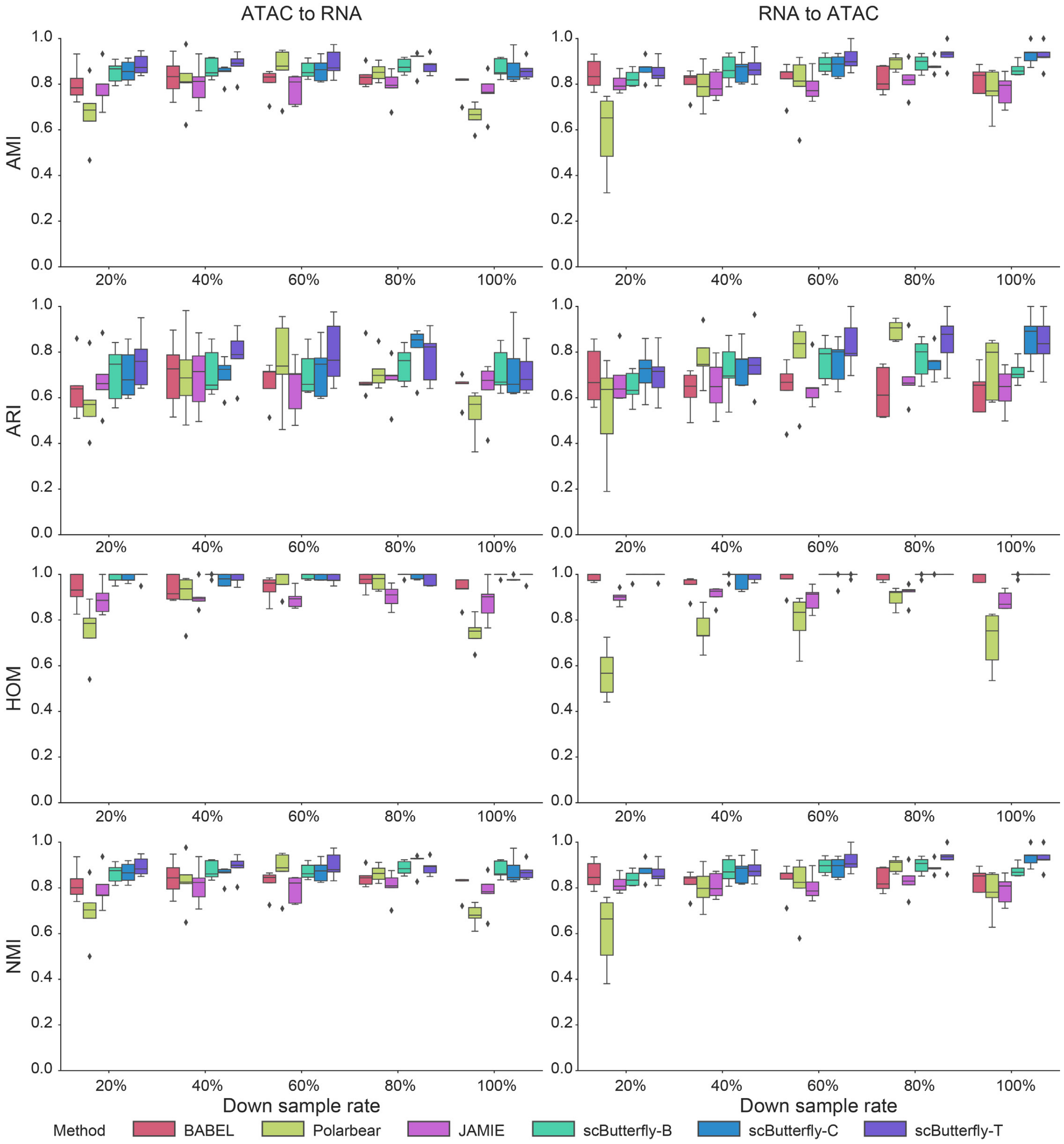

Supplementary Figure 6. Evaluation of the cross-modality translation performance for data with different rate of random down sampled features, including five-fold cross-validation by cell experiments in the CL dataset (n = 5 cross-validations on 549 cells), via cell clustering with metrics of AMI, ARI, HOM and NMI. In the boxplots, the center lines, box limits, whiskers and notches denote the median, upper and lower quartiles, 1.5× interquartile range and 95% confidence interval calculated using a Gaussian-based asymptotic approximation, respectively.

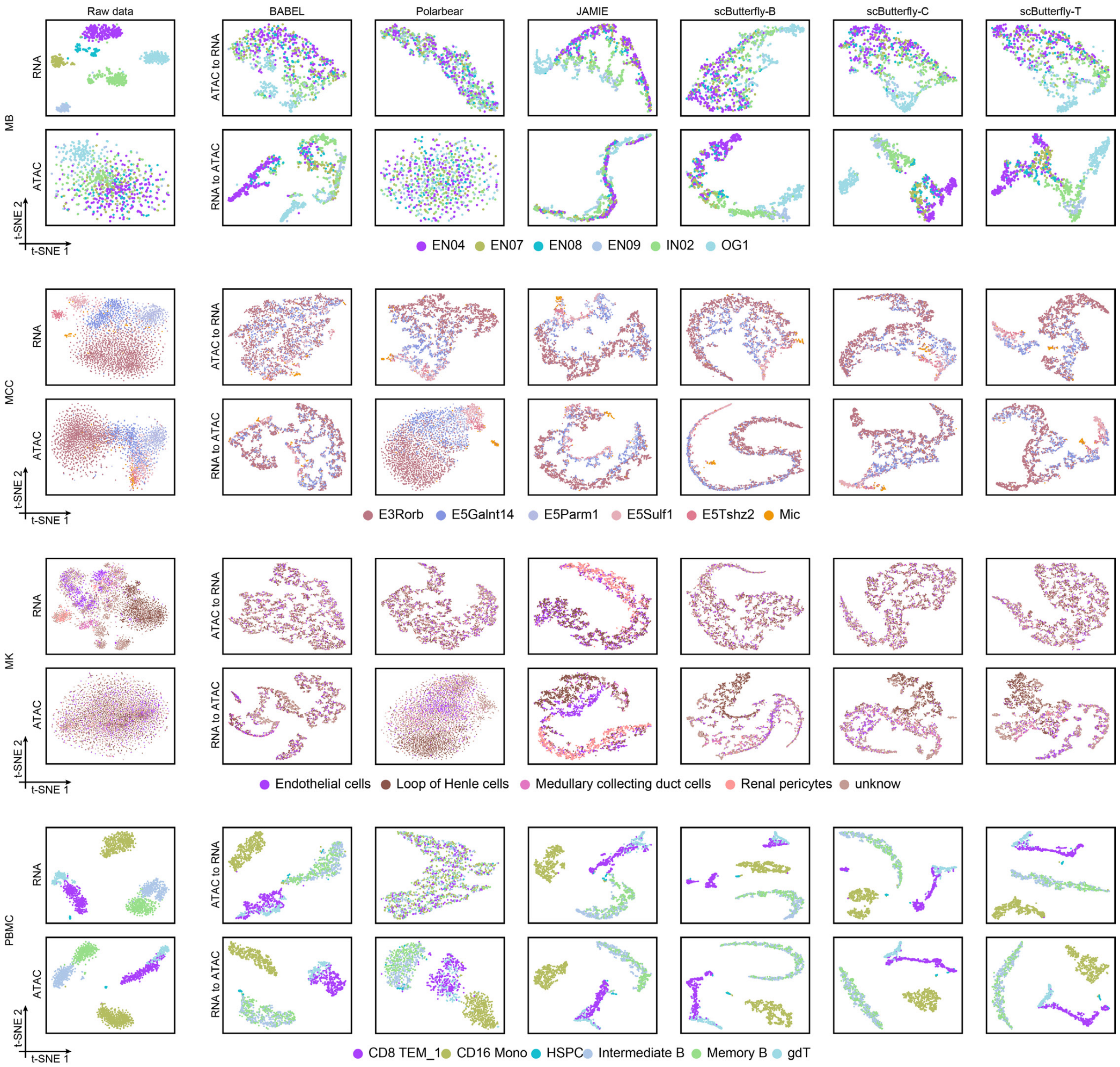

Supplementary Figure 7. t-SNE visualization of cells in the first test fold of three-fold cross-validation by cell type on the four datasets (MB, MCC, MK, and PBMC), using the raw profiles and the profiles translated by different methods.

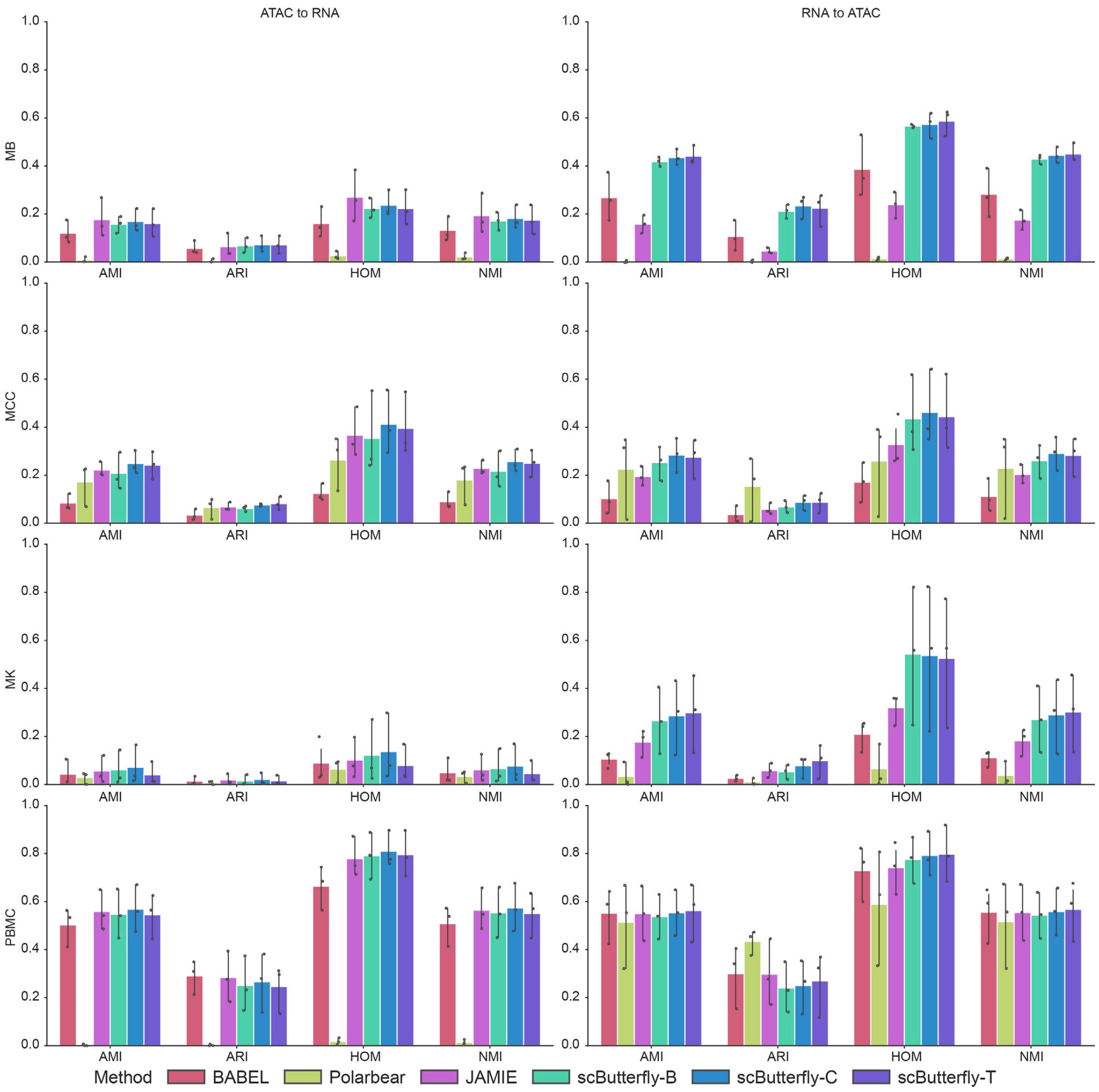

Supplementary Figure 8. Quantitative evaluation of the translation performance between chromatin and transcriptome profiles in three-fold cross-validation by cell type on the four datasets (MB, MCC, MK, and PBMC) (*n* = 3 cross-validations for each dataset), via cell clustering with metrics of AMI, ARI, HOM and NMI. The height of each bar denotes the median value of each metric and the error bars show 95% confidence interval.

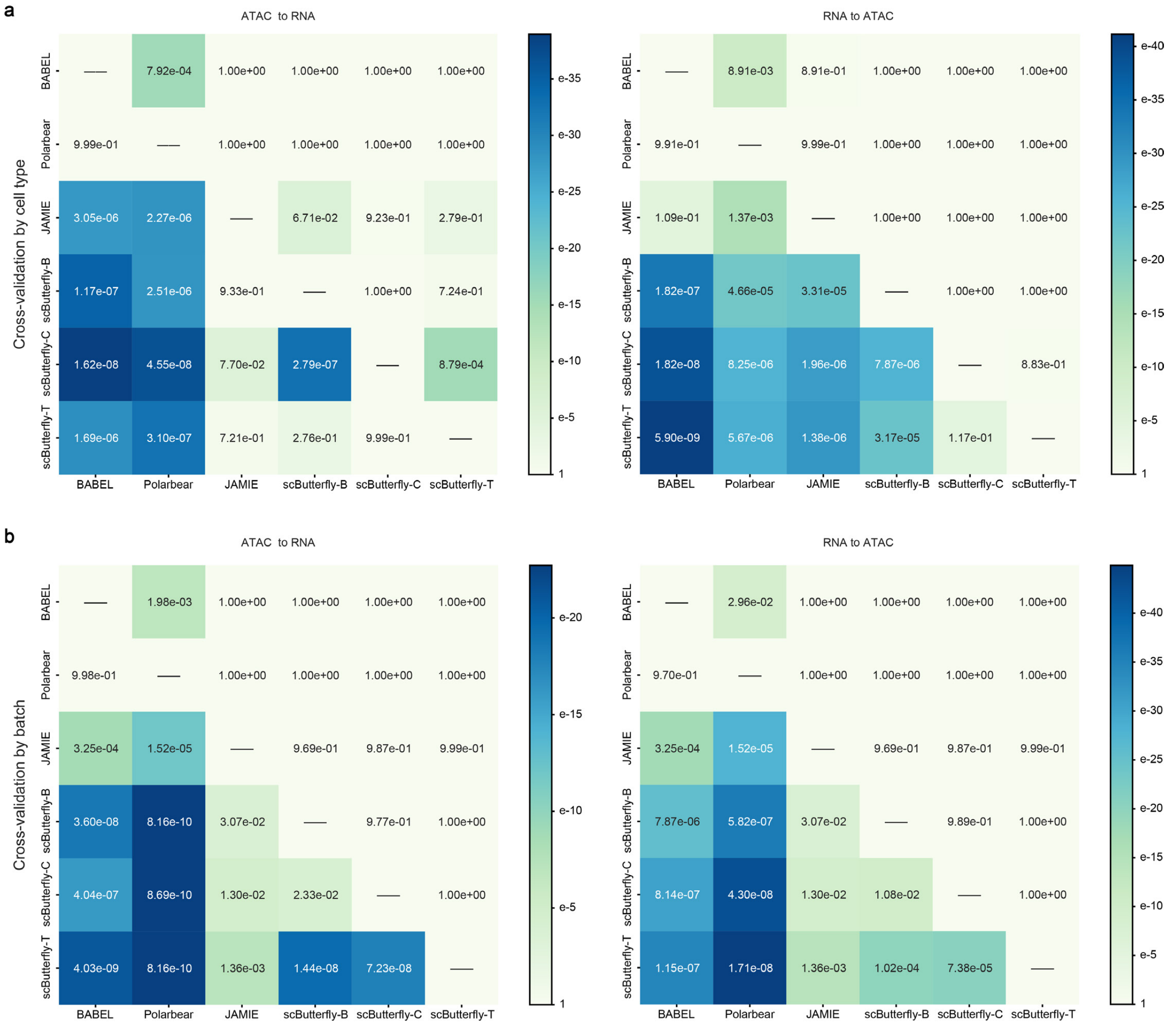

Supplementary Figure 9. a,**b**, Heatmap of *p*-values of one-sided paired Wilcoxon signed-rank tests for three-fold cross-validation by cell type (n = 48 evaluations on four datasets via four metrics) (**a**) and four-fold cross-validation by batch (n = 48 evaluations on three datasets via four metrics) (**b**). Each value in the heatmap indicates the significance of the advantage of a method (row) over another method (column). Note that for the tests associated with JAMIE for four-fold cross-validation by batch, we only considered the CL and MDS dataset, since JAMIE encountered GPU memory errors on the BMMC dataset.

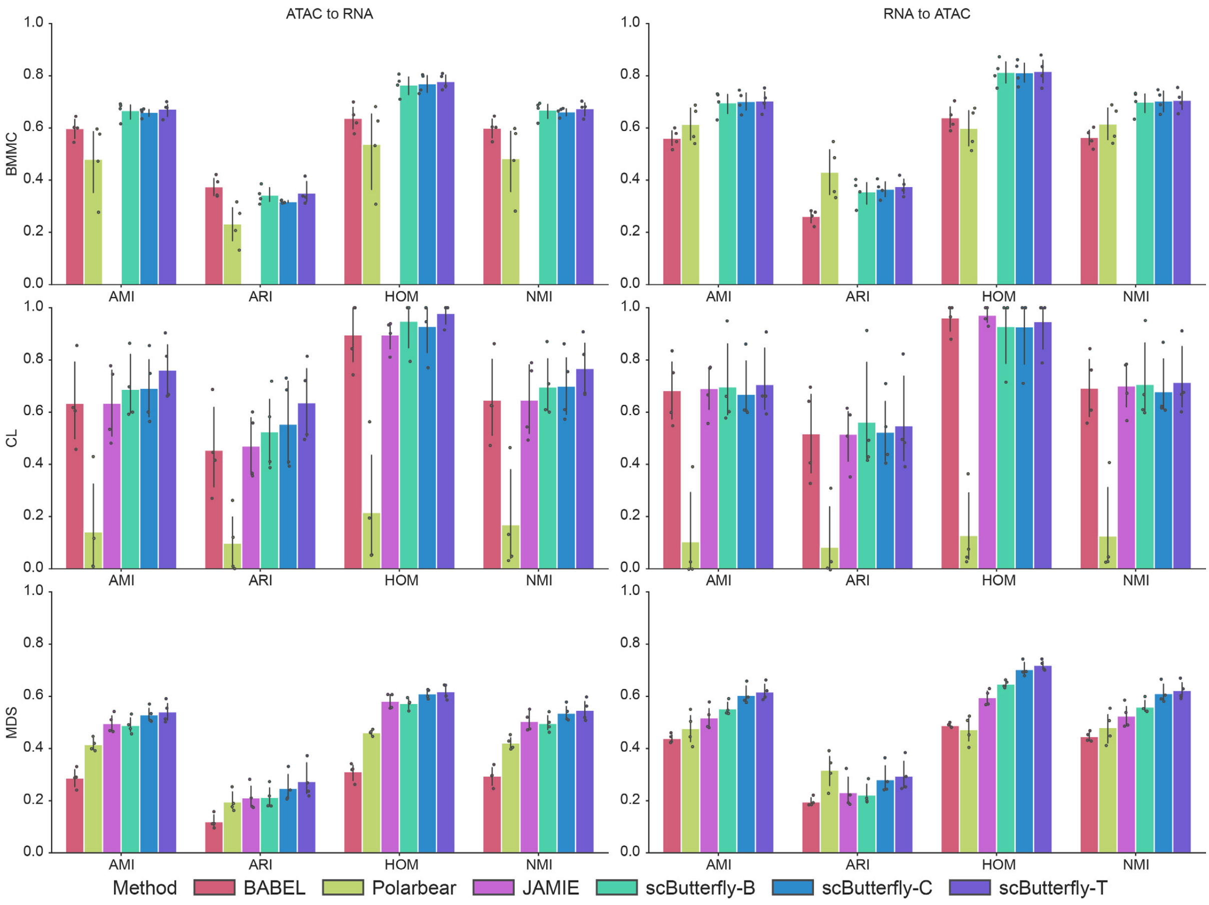

Supplementary Figure 10. Quantitative evaluation of the translation performance between chromatin and transcriptome profiles in four-fold cross-validation by batch on the three datasets (BMMC, CL, and MDS) (n = 4 cross-validations for each dataset), via cell clustering with metrics of AMI, ARI, HOM and NMI. The height of each bar denotes the median value of each metric and the error bars show 95% confidence interval. Note that JAMIE encountered GPU memory errors on the BMMC dataset.

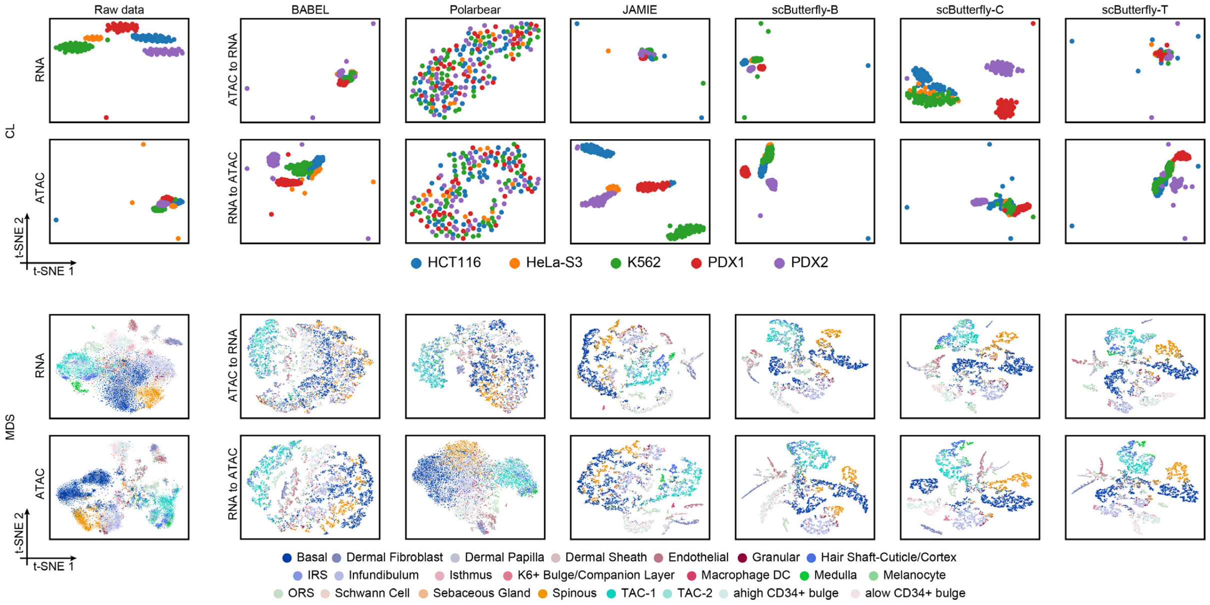

Supplementary Figure 11. t-SNE visualization of cells in the first test fold of four-fold cross-validation by batch on the CL and MDS datasets, using the raw profiles and the profiles translated by different methods.

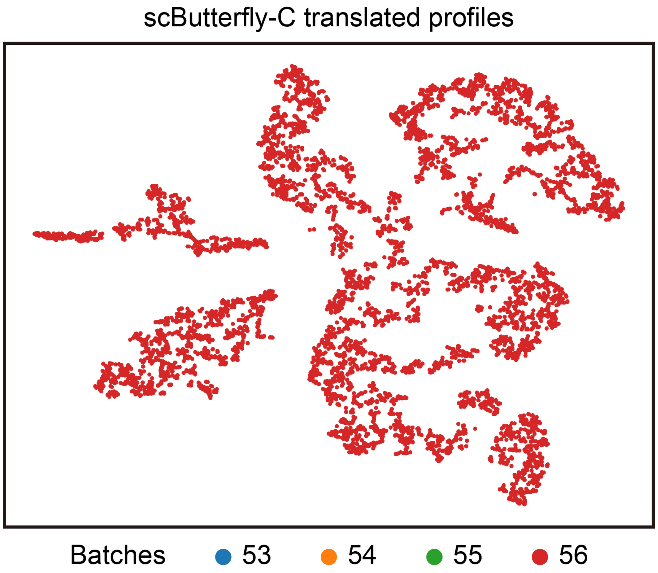

Supplementary Figure 12. t-SNE visualization of cells in the first test fold of cross-validation by batch on the MDS dataset, using the scButterfly-C translated profiles, colored by batches.

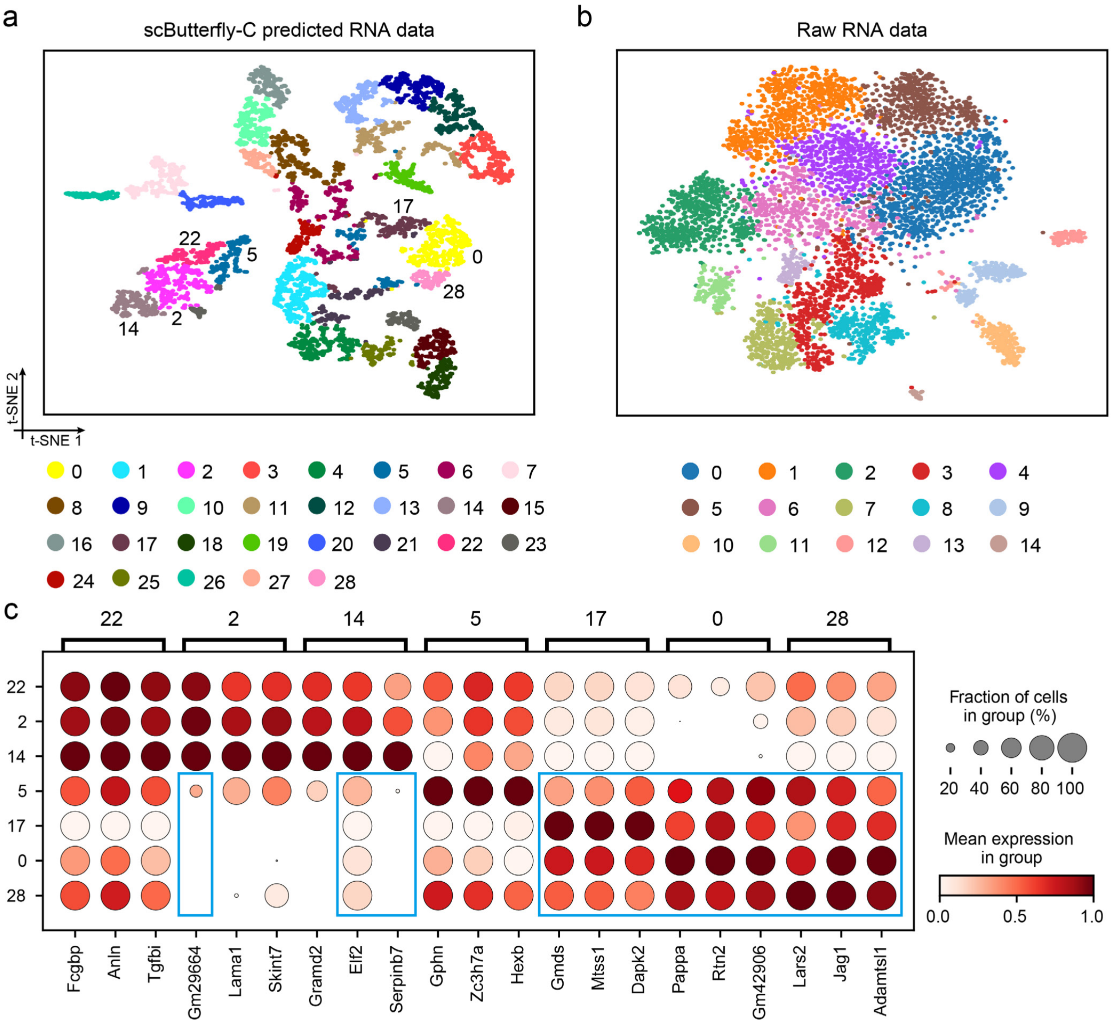

Supplementary Figure 13. a, **b**, t-SNE visualizations of cells in the first test fold of cross-validation by batch on the MDS dataset, using the scButterfly-C translated profiles, colored by Leiden cluster labels (**a**) and two identified subgroups of basal cells (**b**). **c**, Cell-protein dotplots of the scButterfly-C translated profiles for the cells in the first test fold of cross-validation by batch on the MDS dataset, with the top 3 DEGs selected for each cluster. The size of each dot represents the fraction of cells expressing the genes for each cell type, while the color of each dot represents the average expression level of the genes within the cell type. The average expression values are standardized within the range of 0 to 1.

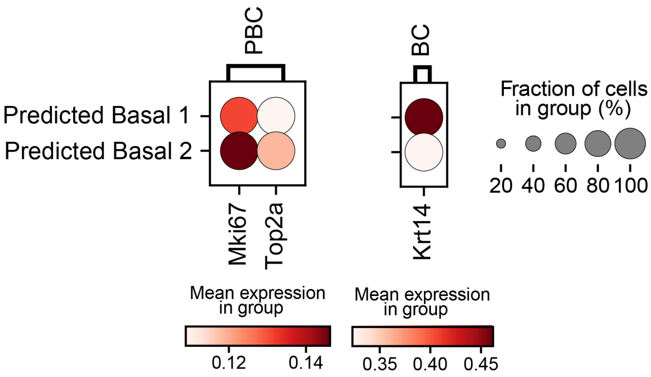

Supplementary Figure 14. Average expression of the PBC and BC markers of identified two subgroups of basal cells in the scButterfly-C translated profiles. The size of each dot represents the fraction of cells expressing the genes for each cell type, while the color of each dot represents the average expression level of the genes within the cell type.

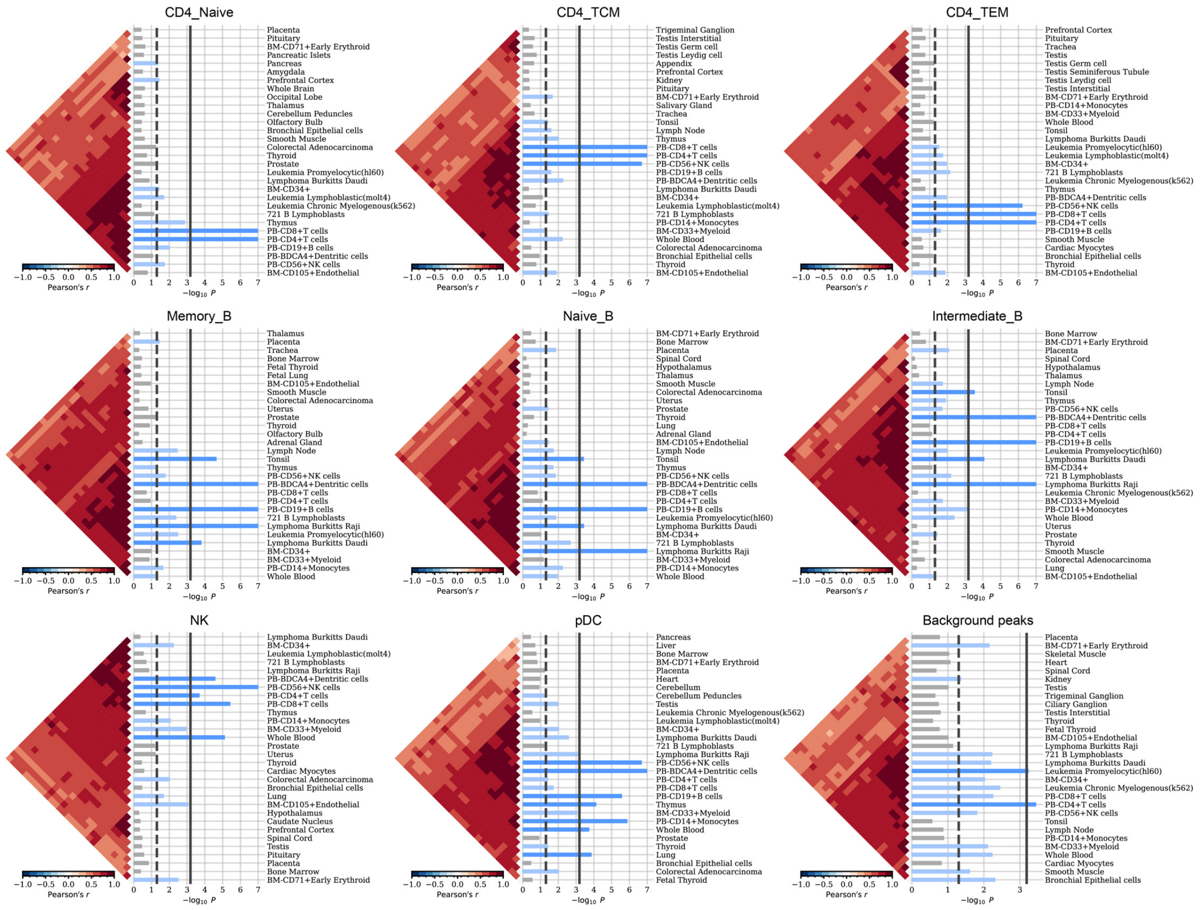

Supplementary Figure 15. SNPsea analysis for the identified cell type-specific peaks for the scButterfly-C translated profiles. The top 30 significantly enriched tissues in SNPsea analysis on OC-specific peaks identified by EpiAnno and the background peaks. The vertical dashed and solid lines denote the one-sided P-value cutoff at the 0.05 level (unadjusted and with Bonferroni correction, respectively), testing the significance of whether all implicated genes, in aggregate, are enriched for specificity to an annotation. The heatmaps show Pearson correlation coefficients for pairs of expression profiles ordered by hierarchical clustering with unweighted pair-group method with arithmetic means (UPGMA).

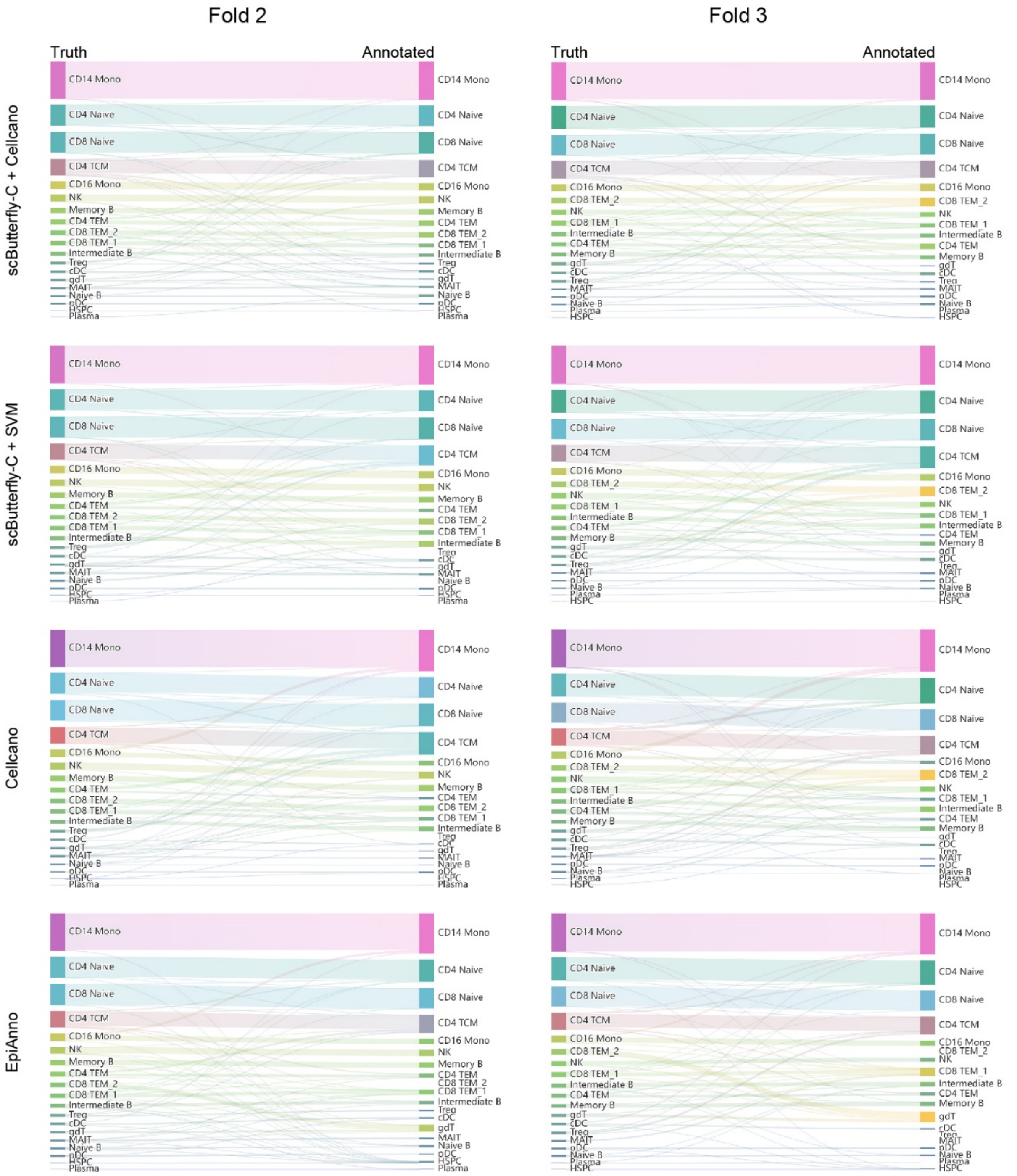

Supplementary Figure 16. Illustration of the cell type annotation results of the well-tailored methods and the combinations of scButterfly-C with different classifiers for scATAC-seq data of the folds 2, 3 of five-fold cross-validation by cell on the PBMC dataset.

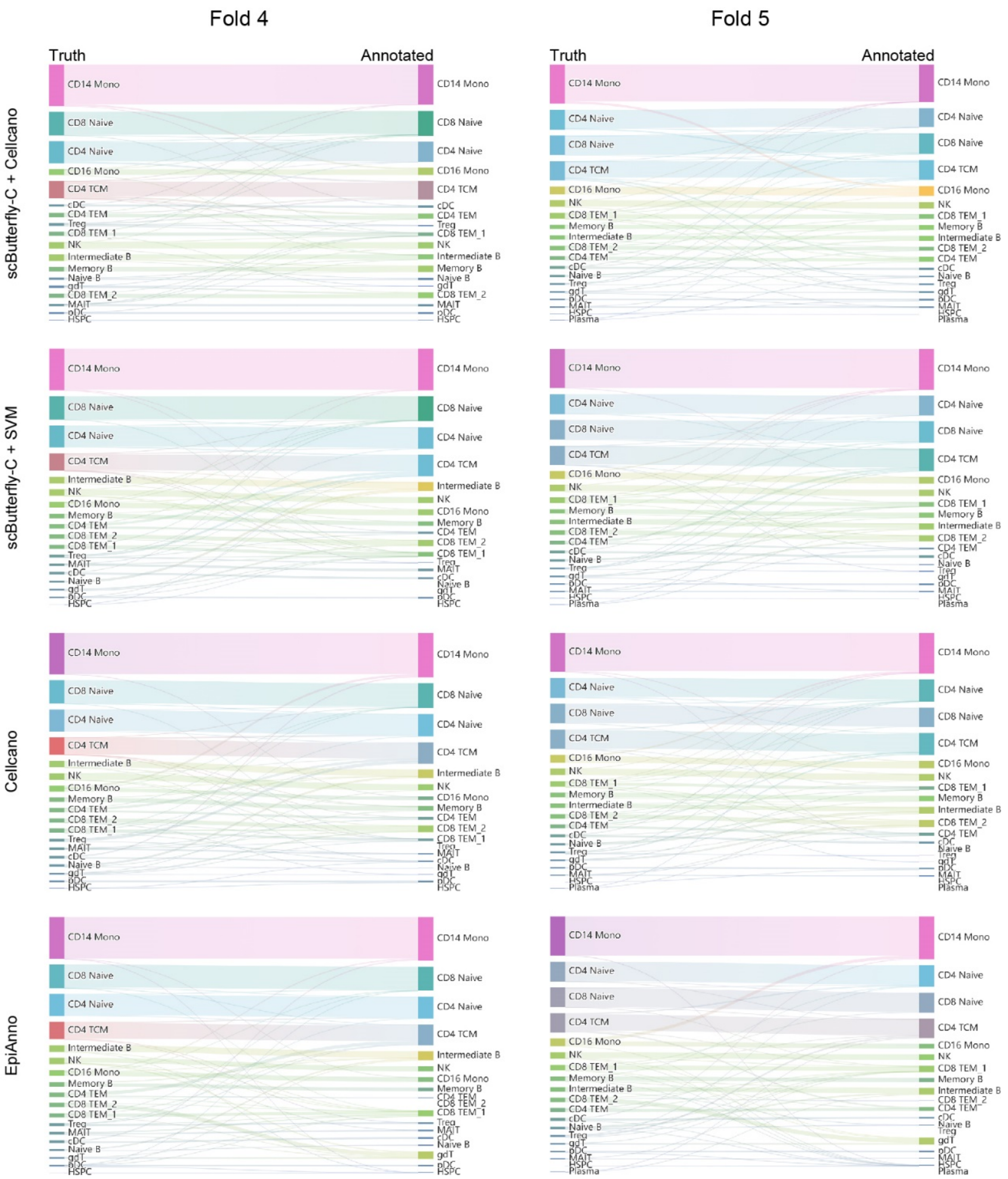

Supplementary Figure 17. Illustration of the cell type annotation results of the well-tailored methods and the combinations of scButterfly-C with different classifiers for scATAC-seq data of the folds 4, 5 of five-fold cross-validation by cell on the PBMC dataset.

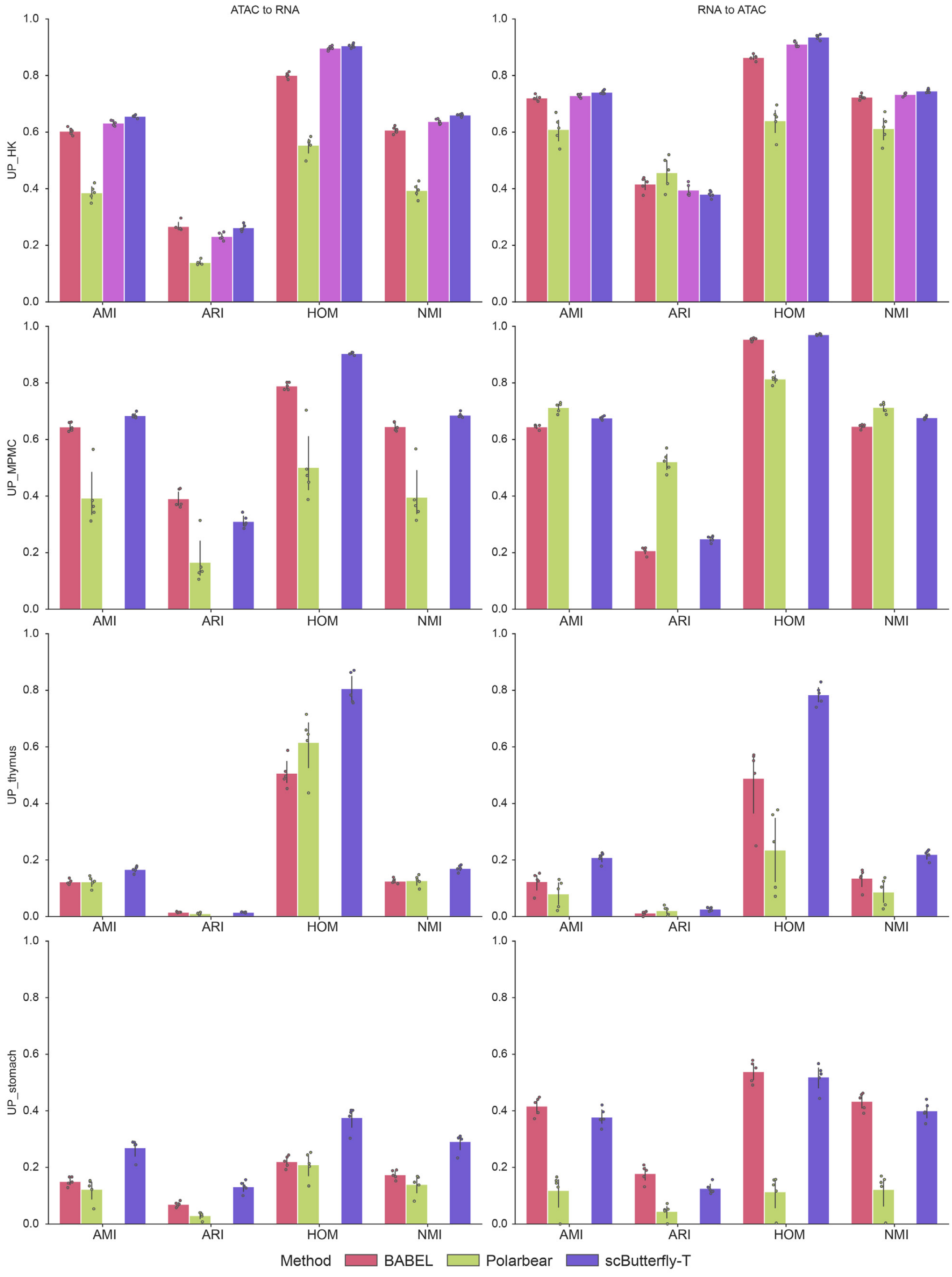

Supplementary Figure 18. Quantitative evaluation of the cross-modality translation performance for unpaired data in five-fold cross-validation by cell on the four datasets (UP_HK, UP_MPMC, UP_thymus, and UP_stomach) (n = 5 cross-validations for each dataset), via cell clustering with metrics of AMI, ARI, HOM and NMI. The height of each bar denotes the median value of each metric and the error bars show 95% confidence interval. Note
735 that JAMIE encountered GPU memory errors on all the datasets except the UP_HK dataset.

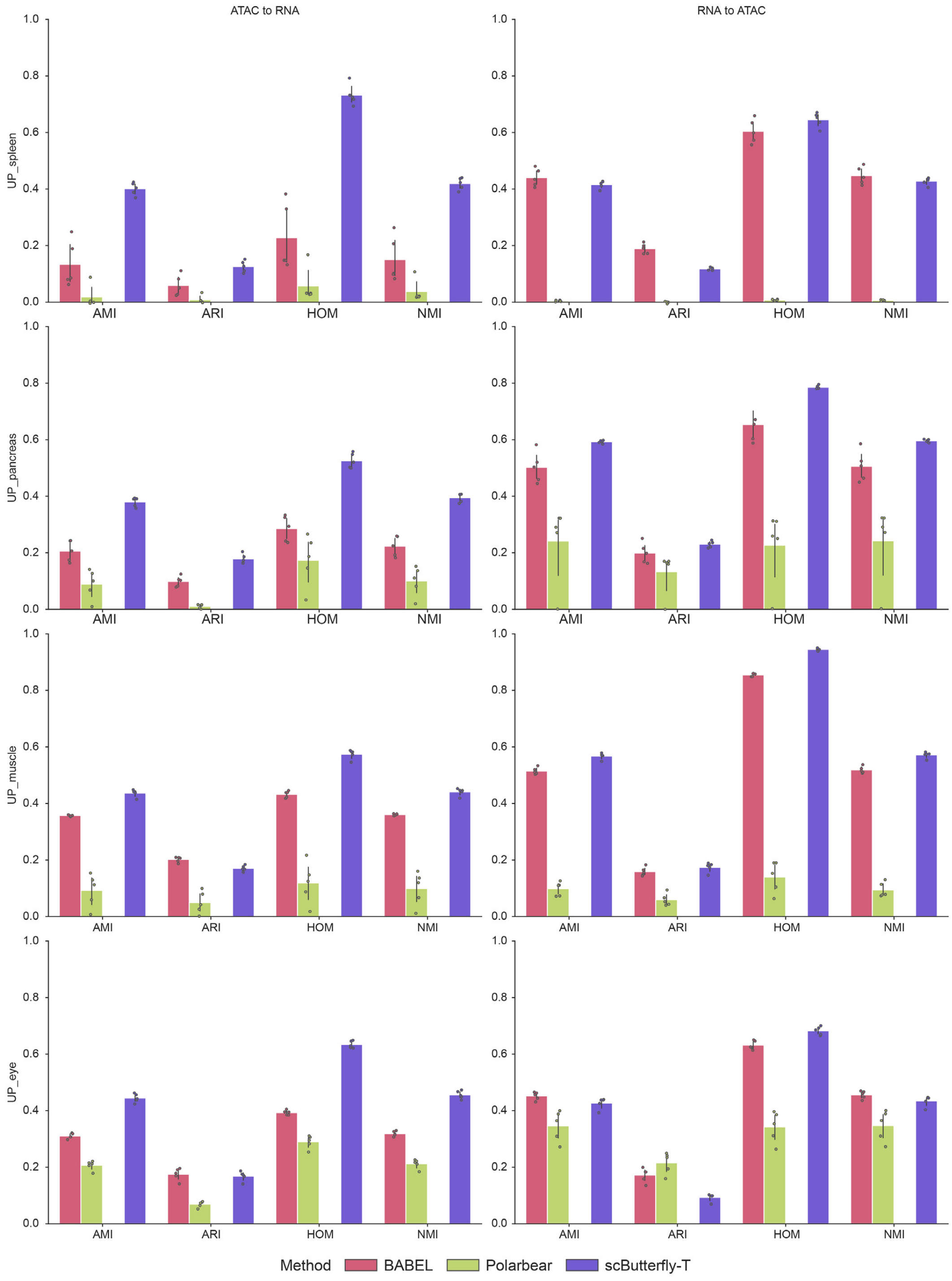

Supplementary Figure 19. Quantitative evaluation of the cross-modality translation performance for unpaired data in five-fold cross-validation by cell on the four datasets (UP_spleen, UP_pancreas, UP_muscle, and UP_eye) (n = 5 cross-validations for each dataset), via cell clustering with metrics of AMI, ARI, HOM and NMI. The height of each bar denotes the median value of each metric and the error bars show 95% confidence interval. Note that
736 JAMIE encountered GPU memory errors on all the datasets.

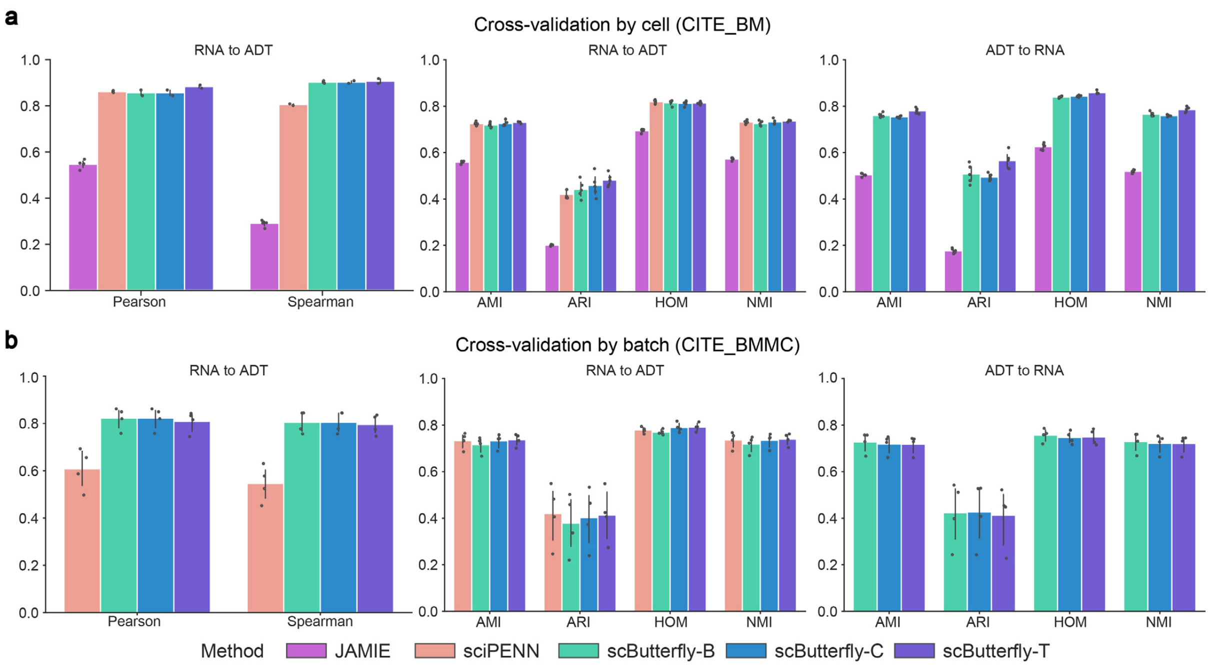

Supplementary Figure 20. a, **b**, Quantitative evaluation of the translation performance between transcriptome and proteome profiles in five-fold cross-validation by cell on the CITE_BM dataset (*n* = 5 cross-validations on 30672 cells) (**a**) and four-fold cross-validation by batch on the CITE_BMMC dataset (*n* = 4 cross-validations on 90261 cells) (**b**), via cell clustering with metrics of AMI, ARI, HOM and NMI. The height of each bar denotes the median value of each metric and the error bars show 95% confidence interval. Note that JAMIE encountered GPU memory errors on the CITE_BMMC dataset.

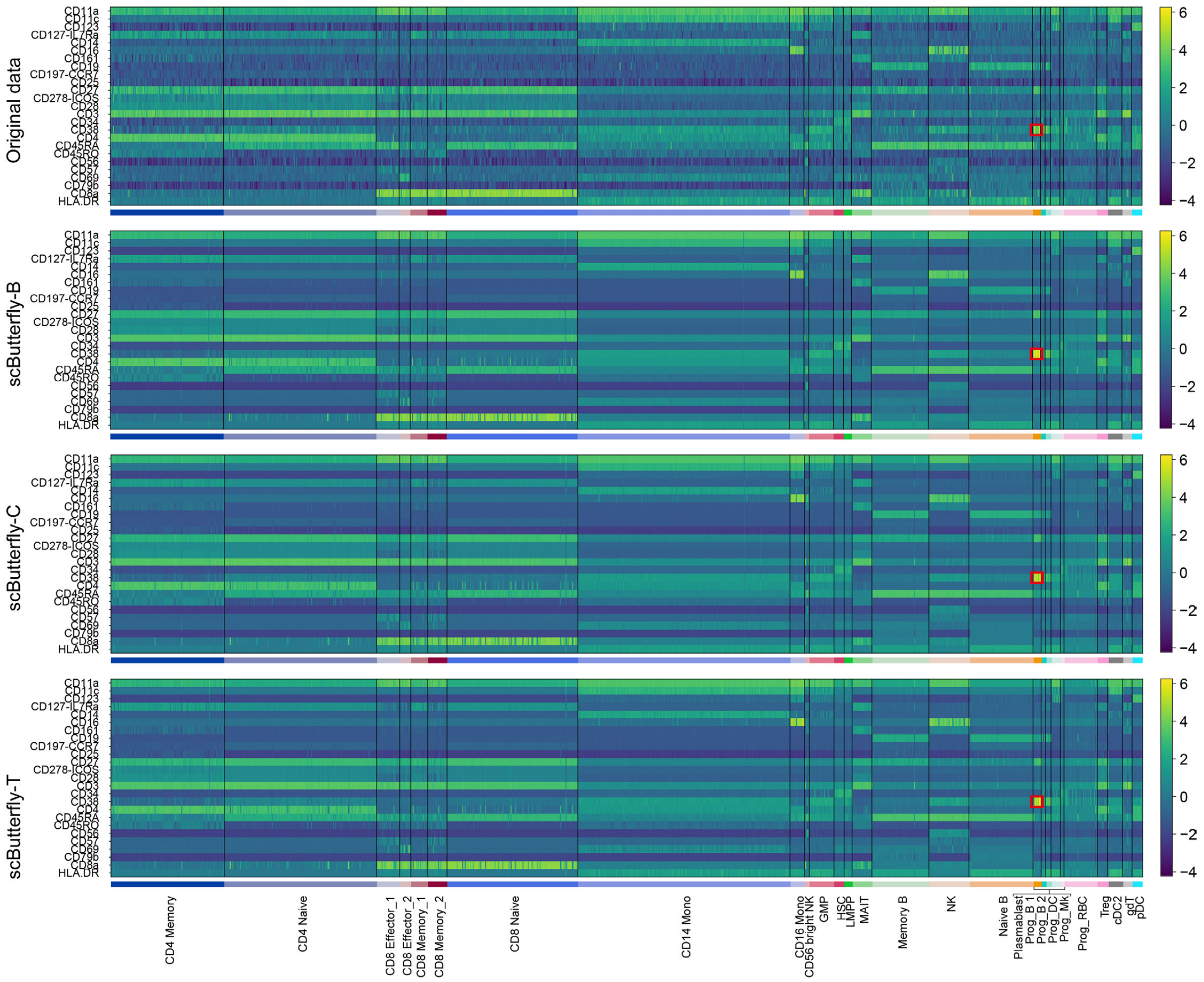

Supplementary Figure 21. Cell-protein heatmaps of the original data and the translation from scButterfly, for the cells in the first fold of five-fold cross-validation by cell on the CITE_BM dataset.

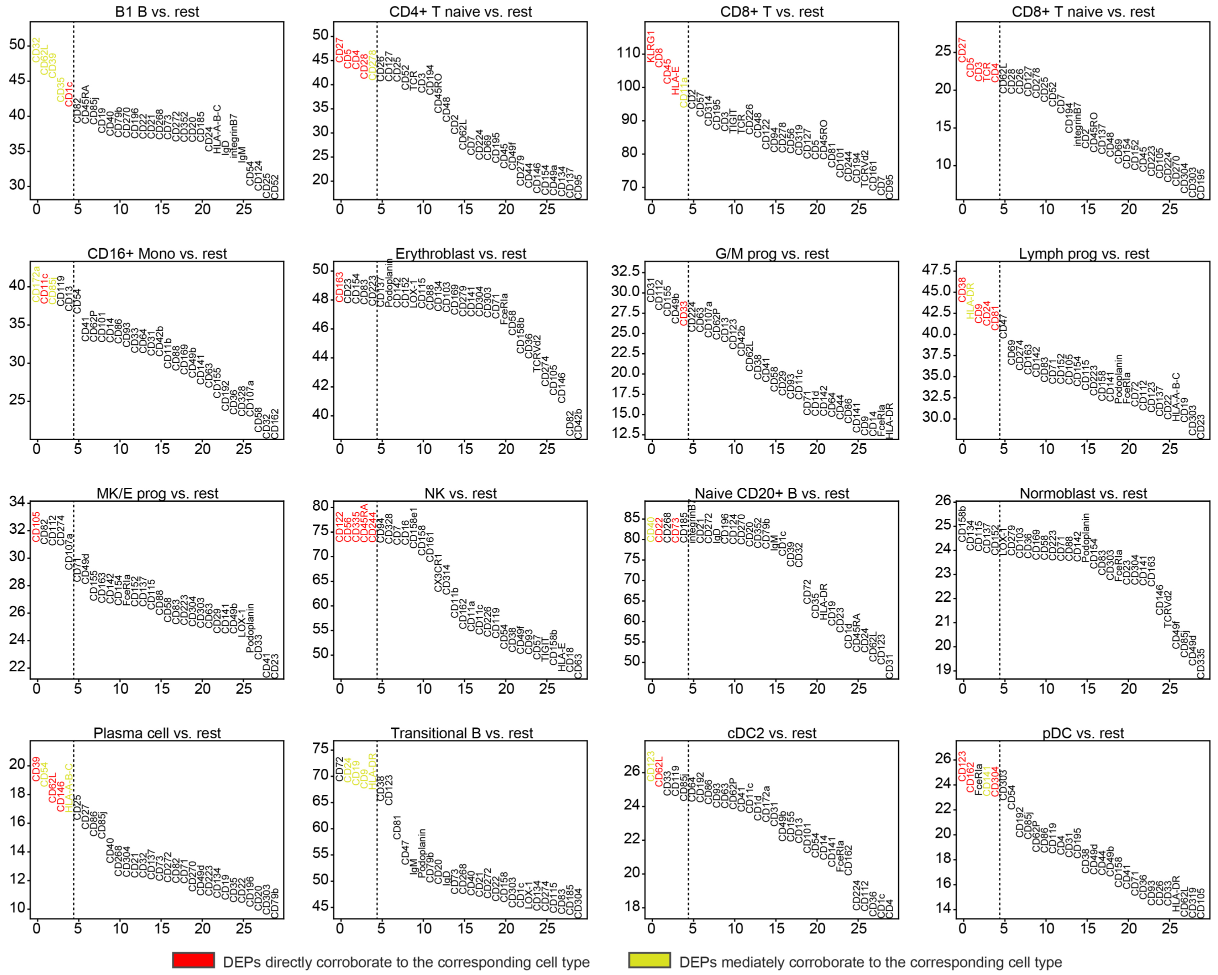

Supplementary Figure 22. Differentially expressed proteins (DEPs) of different cell types in the proteome profiles that were consecutively translated from epigenome to transcriptome and then to proteome based on the BMMC and CITE_BMMC datasets. The DEPs were obtained via one-sided Wilcoxon rank-sum tests.

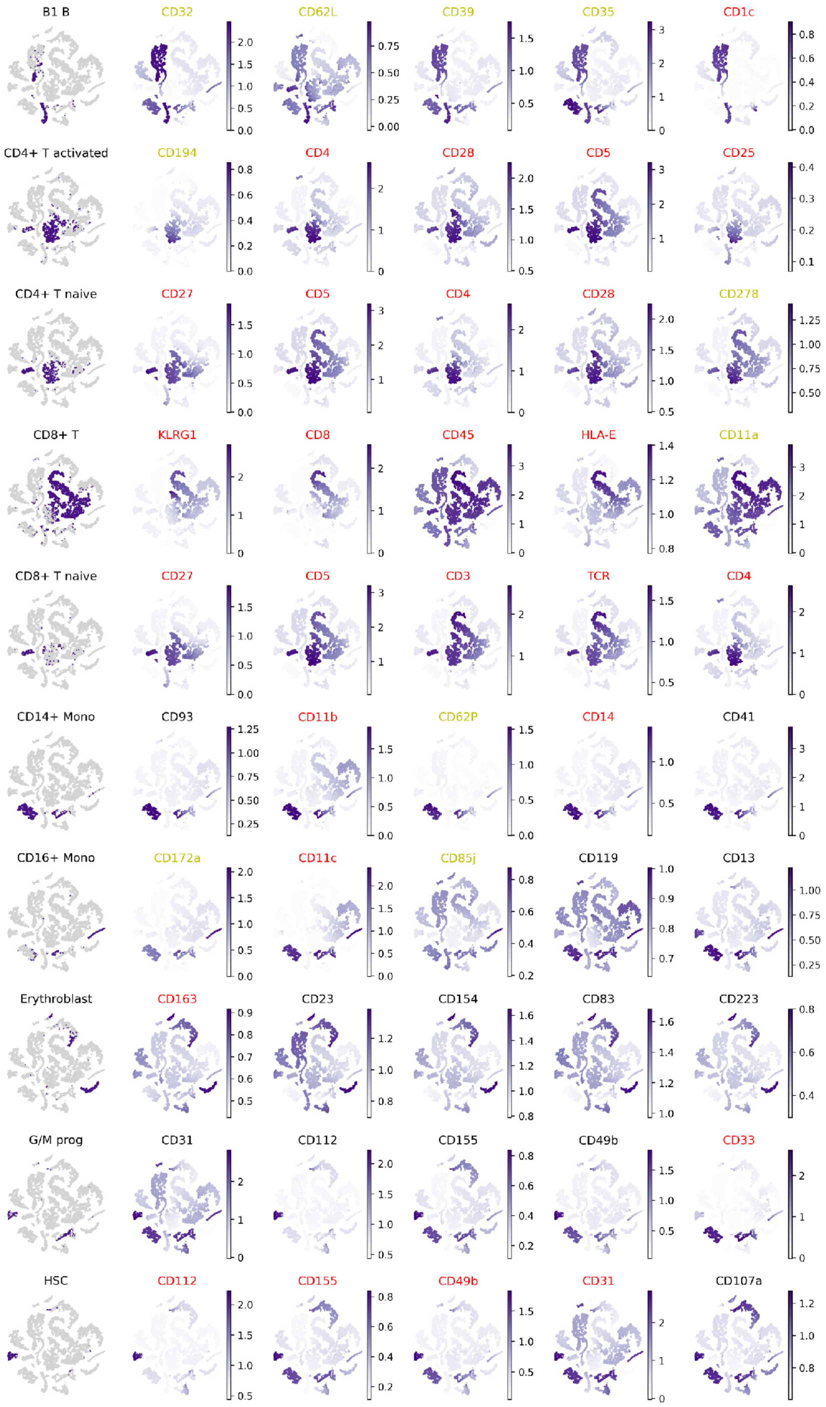

Supplementary Figure 23. t-SNE visualization of cells in the first test fold (with batches independent with the batches for training) of BMMC dataset based on the proteome profiles consecutively translated by scButterfly-C. The distribution of each cell type (Column #1) and the scButterfly-C-predicted expression levels of the top five differentially expressed proteins (DEPs) of each cell type (Columns #2-6) are projected onto the t-SNE visualization. The proteins among the top five DEPs directly and mediately corroborated to the corresponding cell type are marked with red and yellow, respectively.

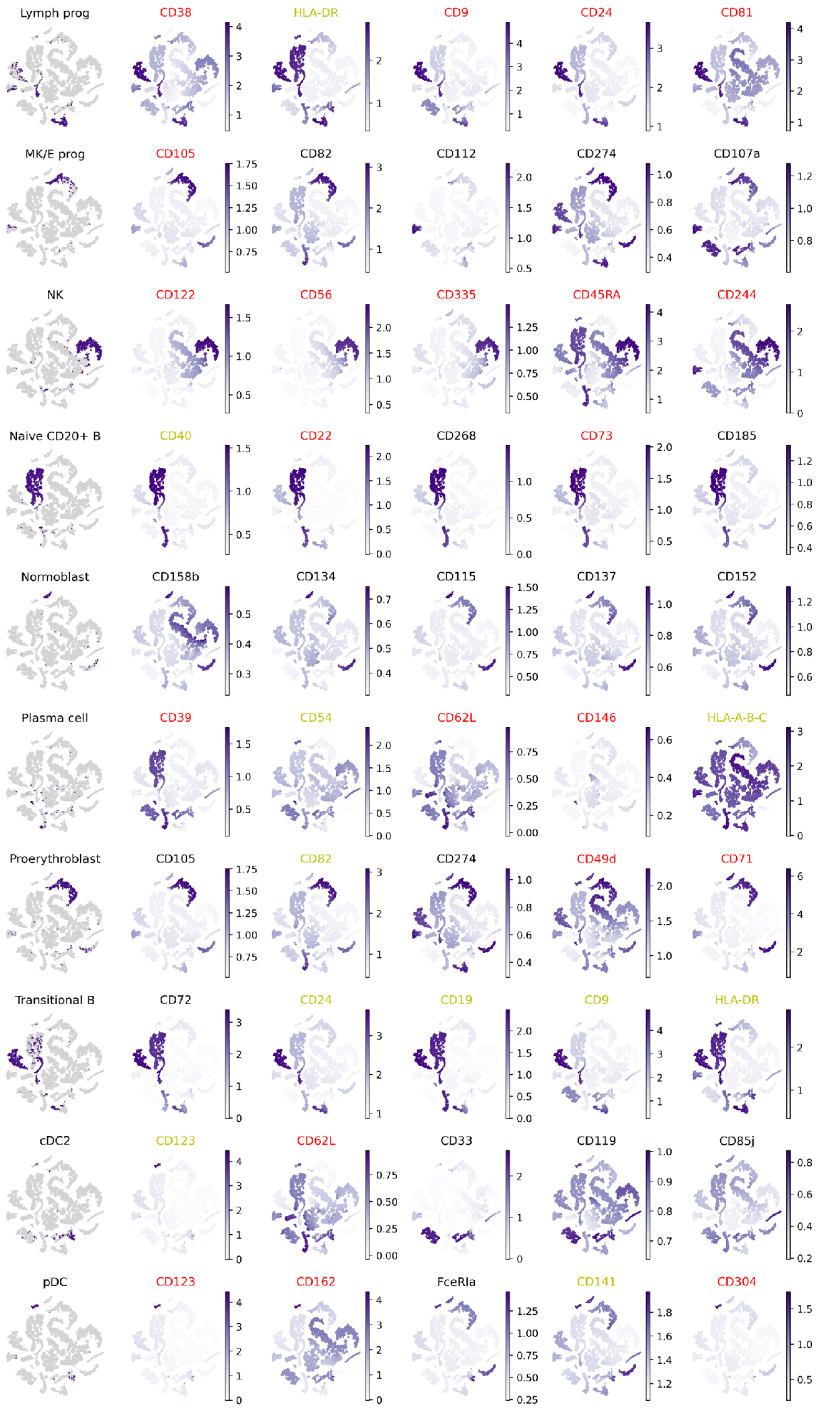

Supplementary Figure 24. t-SNE visualization of cells in the first test fold (with batches independent with the batches for training) of BMMC dataset based on the proteome profiles consecutively translated by scButterfly-C. The distribution of each cell type (Column #1) and the scButterfly-C-predicted expression levels of the top five differentially expressed proteins (DEPs) of each cell type (Columns #2-6) are projected onto the t-SNE visualization. The proteins among the top five DEPs directly and mediately corroborated to the corresponding cell type are marked with red and yellow, respectively.

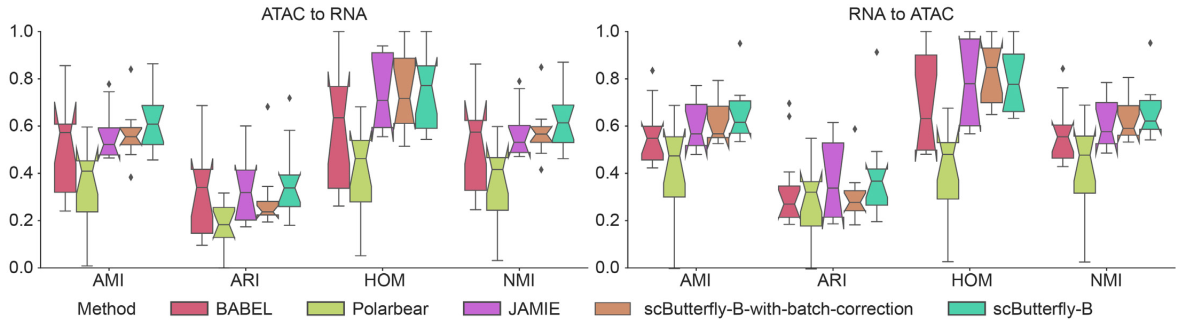

Supplementary Figure 25. Evaluation of the cross-modality translation performance for paired data, including four-fold cross-validation by batch experiments in three multi-batch datasets of BMMC, CL, and MDS (n = 12 cross-validations on three datasets), via cell clustering with metrics of AMI, ARI, HOM and NMI. Note that JAMIE encountered GPU memory errors on the BMMC dataset. In the boxplots, the center lines, box limits, whiskers and notches denote the median, upper and lower quartiles, 1.5× interquartile range and 95% confidence interval calculated using a Gaussian-based asymptotic approximation, respectively.

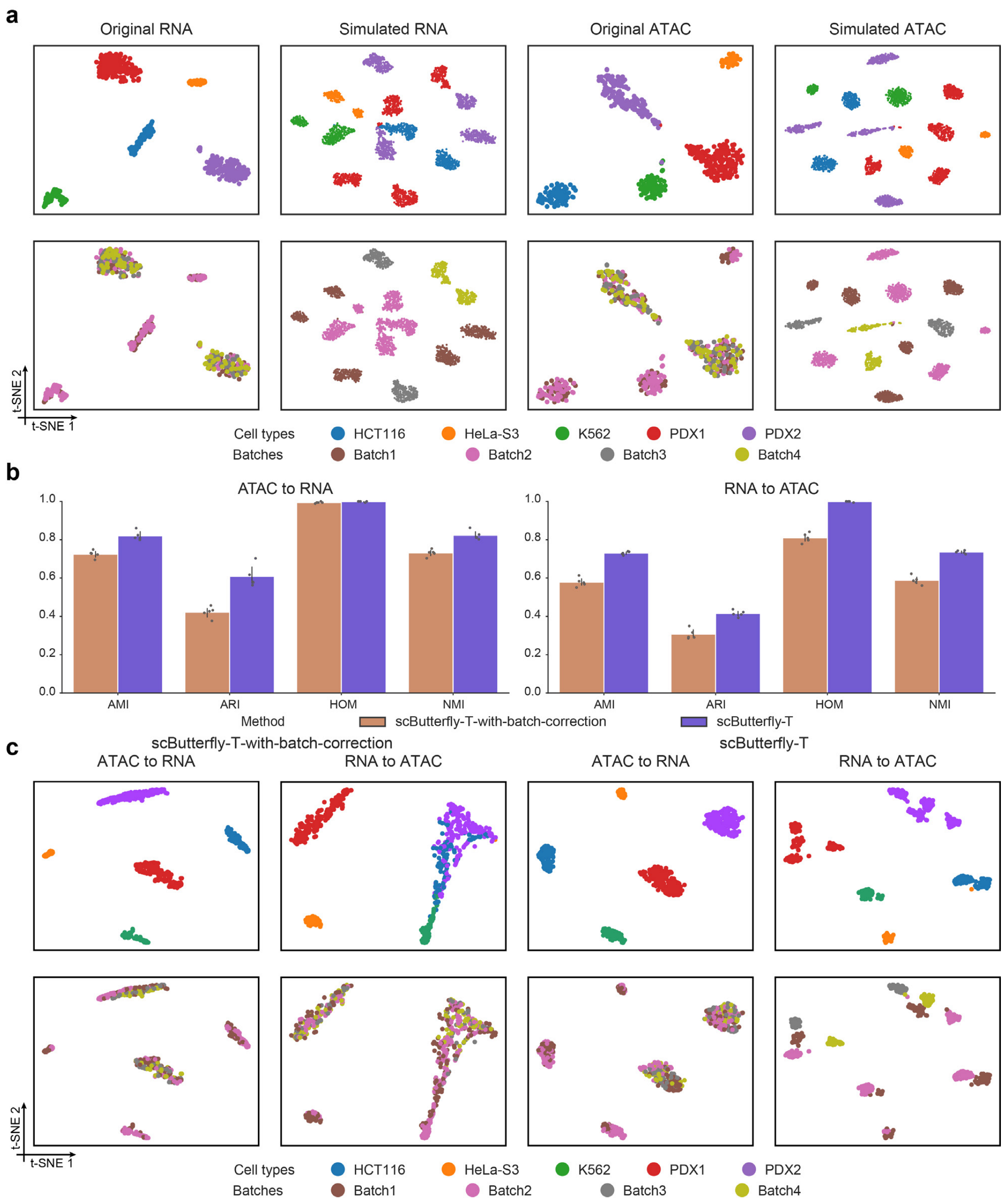

Supplementary Figure 26. a, t-SNE visualizations of cells in the CL dataset, using the original profiles and the profiles simulated by scDesign3 and simCAS, respectively for RNA and ATAC, colored by the cell types and batches. **b**, Evaluation of the cross-modality translation performance in five-fold cross-validation on the simulated CL dataset (n = 5 cross-validations on 3000 simulated cells), via cell clustering with metrics of AMI, ARI, HOM and NMI. The height of each bar denotes the median value of each metric and the error bars show 95% confidence interval. **c**, t-SNE visualizations of cells in the simulated CL dataset, using the profiles translated by scButterfly-T and scButterfly-T-with-batch-correction, respectively for RNA and ATAC, colored by cell types and batches.

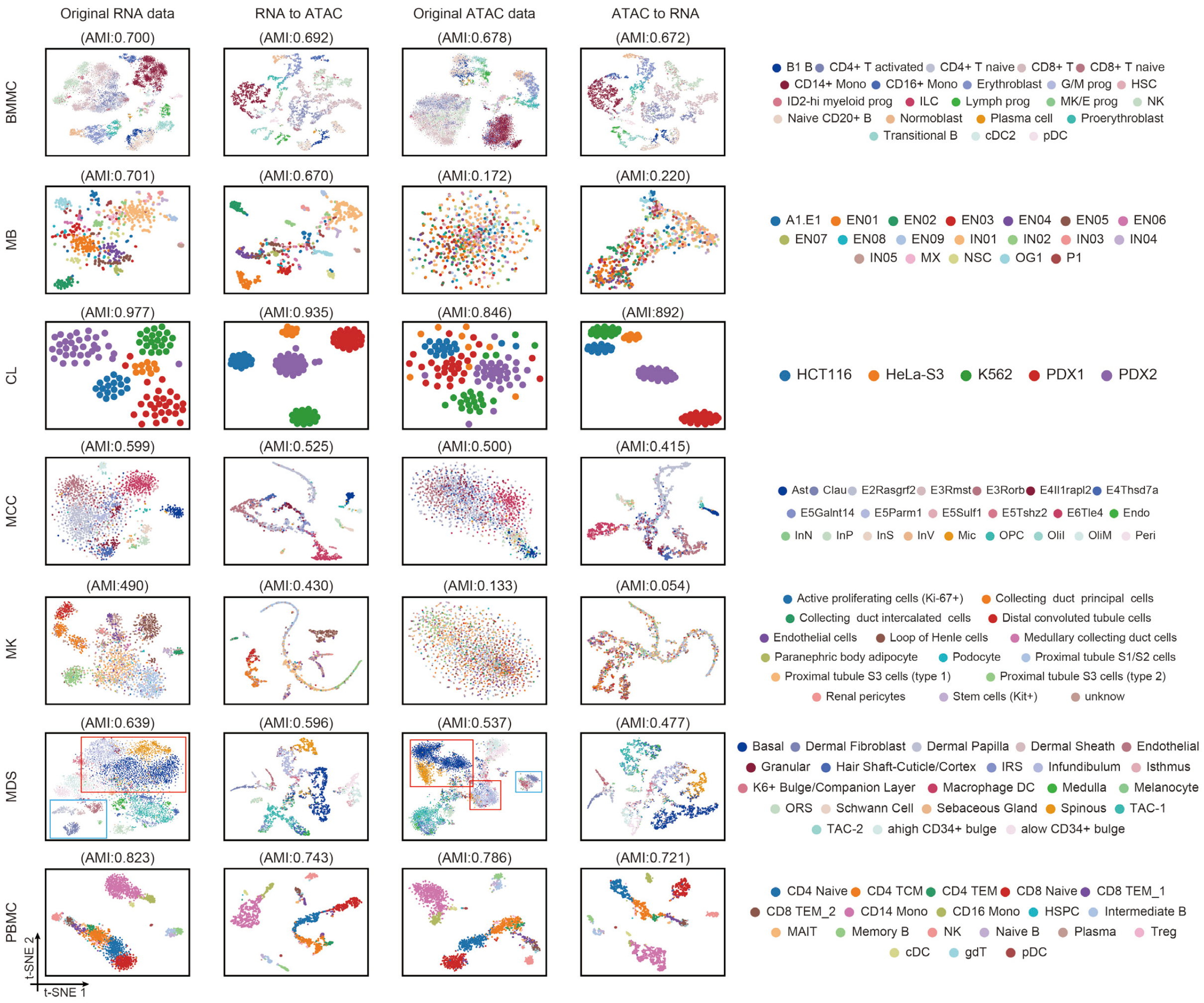

Supplementary Figure 27. t-SNE visualizations of cells in the first test fold of five-fold cross-validation on the seven datasets (BMMC, MB, CL, MCC, MK, MDS, and PBMC), using the original profiles and the profiles translated by scButterfly-B, with AMI clustering performance marked upon the visualization.

748

749

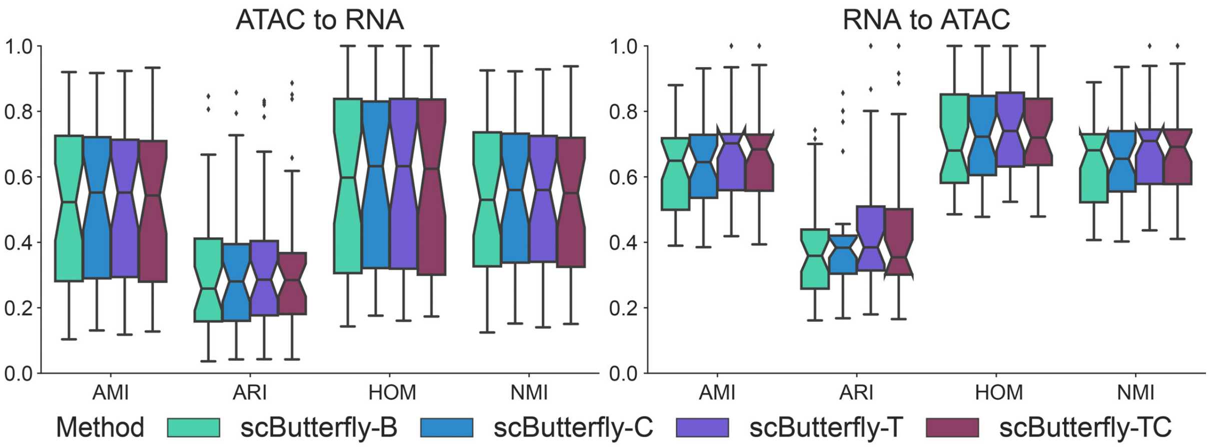

Supplementary Figure 28. Quantitative evaluation of the translated profiles for preserving cell heterogeneity. We evaluated the cross-validation performance on the seven datasets (BMMC, MB, CL, MCC, MK, MDS, and PBMC) (n = 35 cross-validations on seven datasets), via cell clustering with metrics of AMI, ARI, HOM, and NMI. In the boxplots, the center lines, box limits, whiskers and notches denote the median, upper and lower quartiles, 1.5× interquartile range and 95% confidence interval calculated using a Gaussian-based asymptotic approximation, respectively.

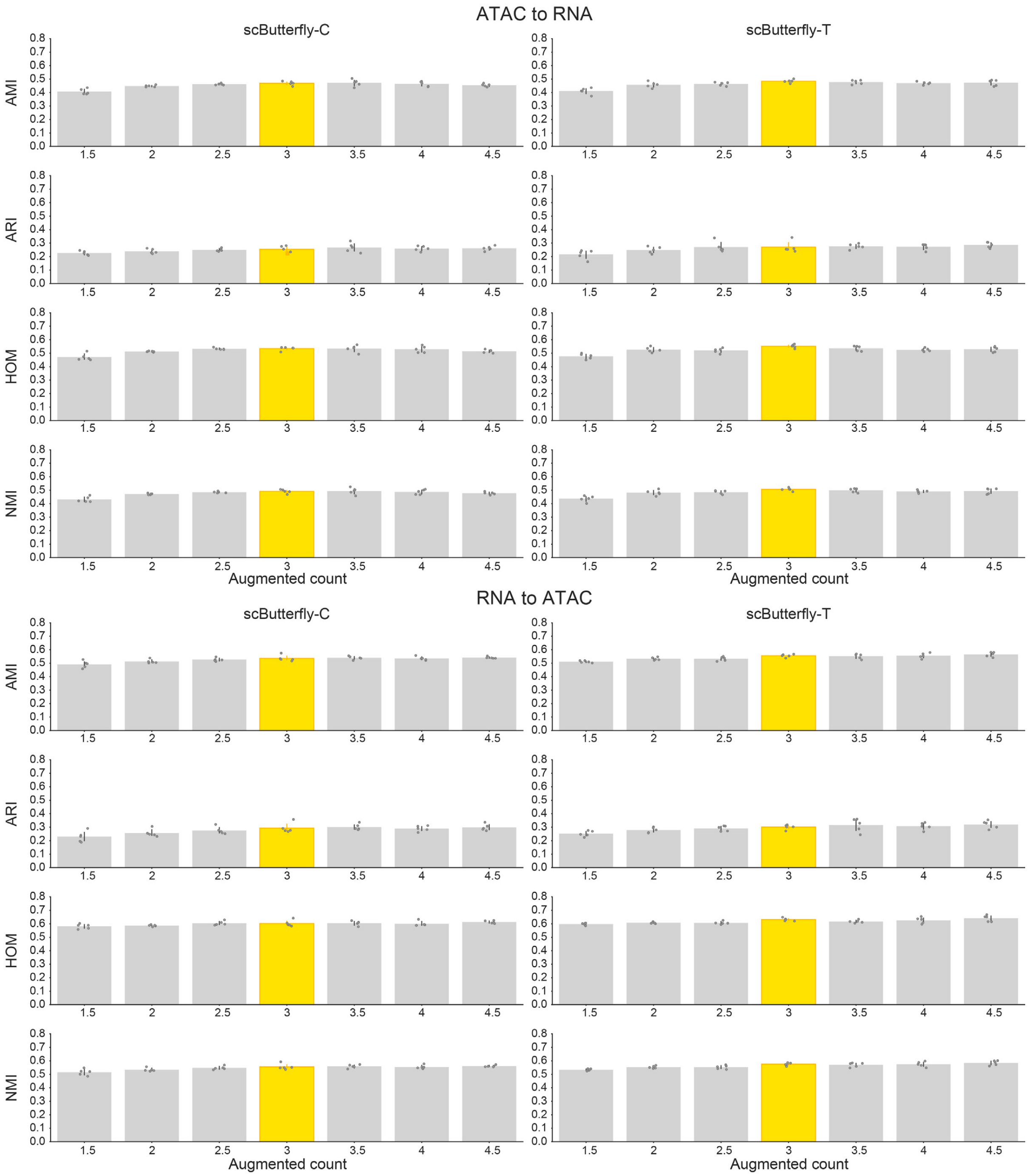

Supplementary Figure 29. Evaluation of translation performance for scButterfly-C and scButterfly-T with different count of augmented data with five-fold cross-validation by cell on the MCC dataset (*n* = 5 cross-validations on 9190 cells). The bars marked with yellow denote the default setting of scButterfly-C and scButterfly-T. The height of each bar denotes the median value of each metric and the error bars show 95% confidence interval.

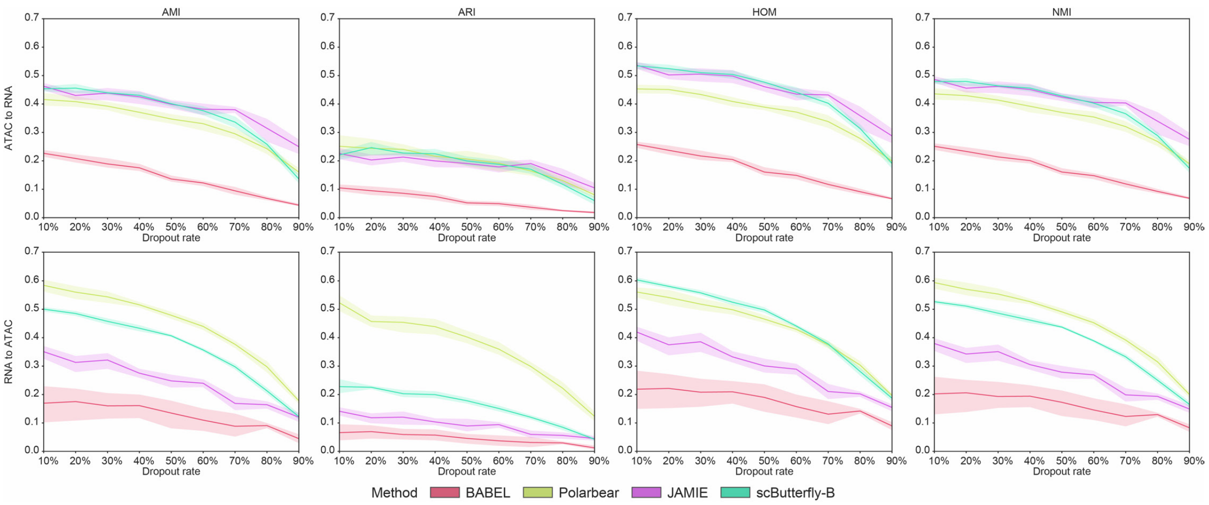

Supplementary Figure 30. Clustering performance using various methods on the MCC dataset at different dropout rates evaluated by AMI, ARI, HOM, and NMI. Each point on the lines denotes the median performance in five-fold cross-validation at a specific dropout rate (*n* = 5 cross-validations for each dropout rate), with error bands implying the 95% confidence

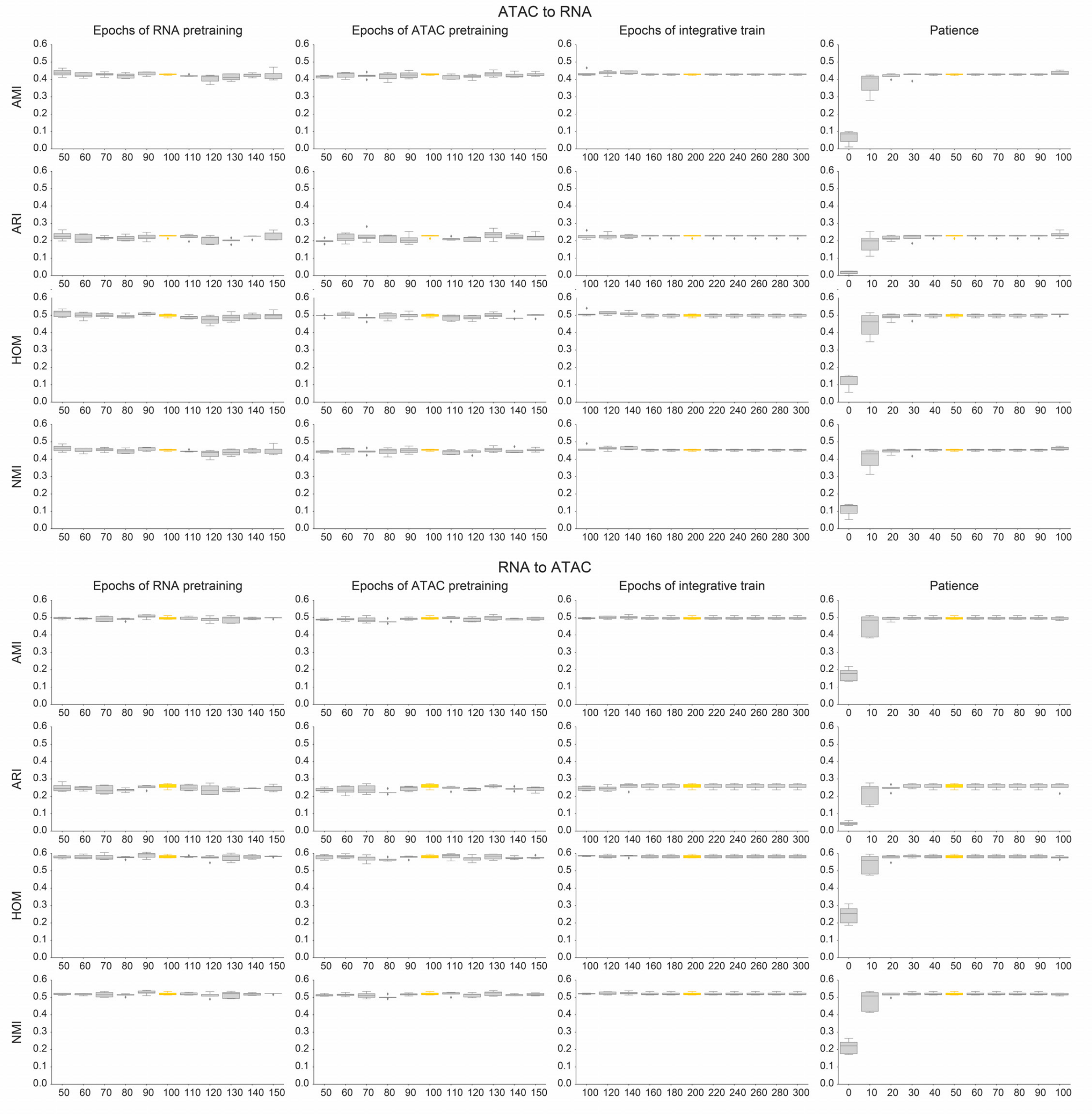

Supplementary Figure 31. Evaluation of translation performance for scButterfly-B under different training epochs and early-stop settings with five-fold cross-validation on the MCC dataset (n = 5 cross-validations on 9190 cells). The boxplots marked with yellow denote the default hyperparameter setting of scButterfly-B. In the boxplots, the center lines, box limits and whiskers denote the median, upper and lower quartiles, and 1.5× interquartile range, respectively.

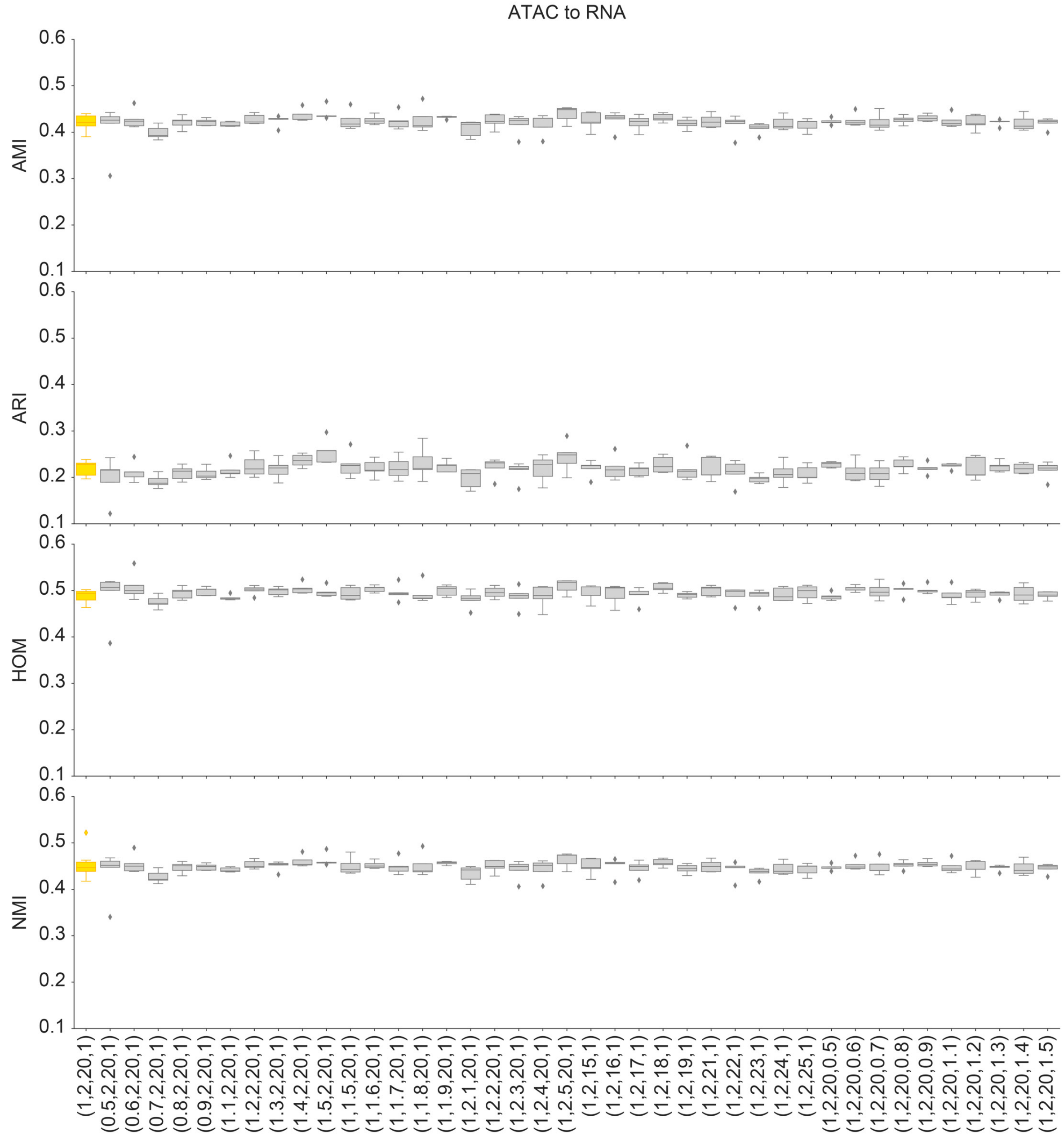

Supplementary Figure 32. Performance of scButterfly-B with different hyperparameters on the MCC dataset (n = 5 cross-validations on 9190 cells). The settings of hyperparameter are denoted as (w_r, w_a, w_e, w_{dis}) and we evaluated the performance of scButterfly-B under 41 different hyperparameter settings. The boxplots marked with yellow denote the default hyperparameter setting of scButterfly-B. In the boxplots, the center lines, box limits and whiskers denote the median, upper and lower quartiles, and 1.5× interquartile range, respectively.

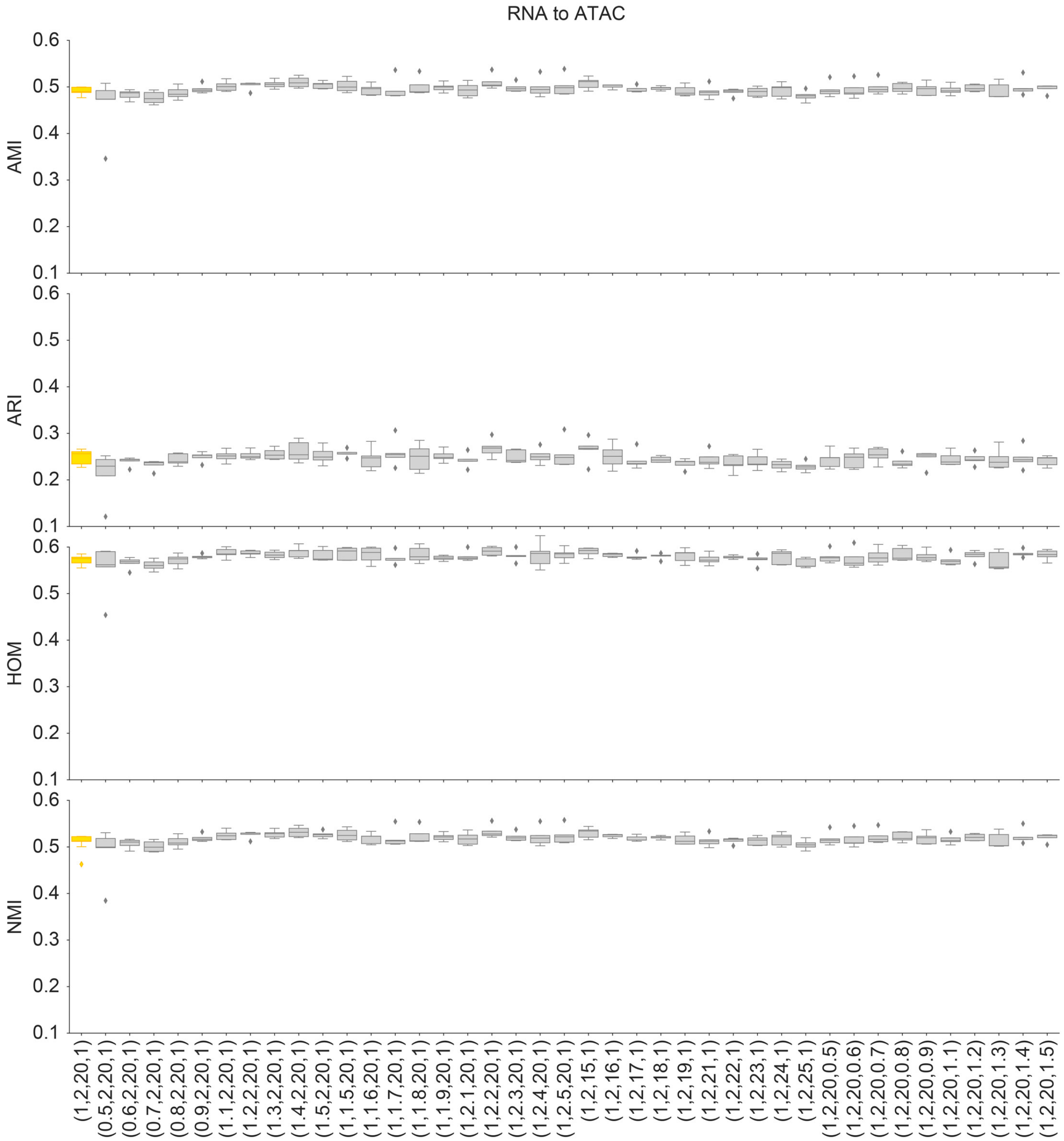

Supplementary Figure 33. Performance of scButterfly-B with different hyperparameters on the MCC dataset (*n* = 5 cross-validations on 9190 cells). The settings of hyperparameter are denoted as (w_r, w_a, w_e, w_{dis}) and we evaluated the performance of scButterfly-B under 41 different hyperparameter settings. The boxplots marked with yellow denote the default hyperparameter setting of scButterfly-B. In the boxplots, the center lines, box limits and whiskers denote the median, upper and lower quartiles, and 1.5× interquartile range, respectively.

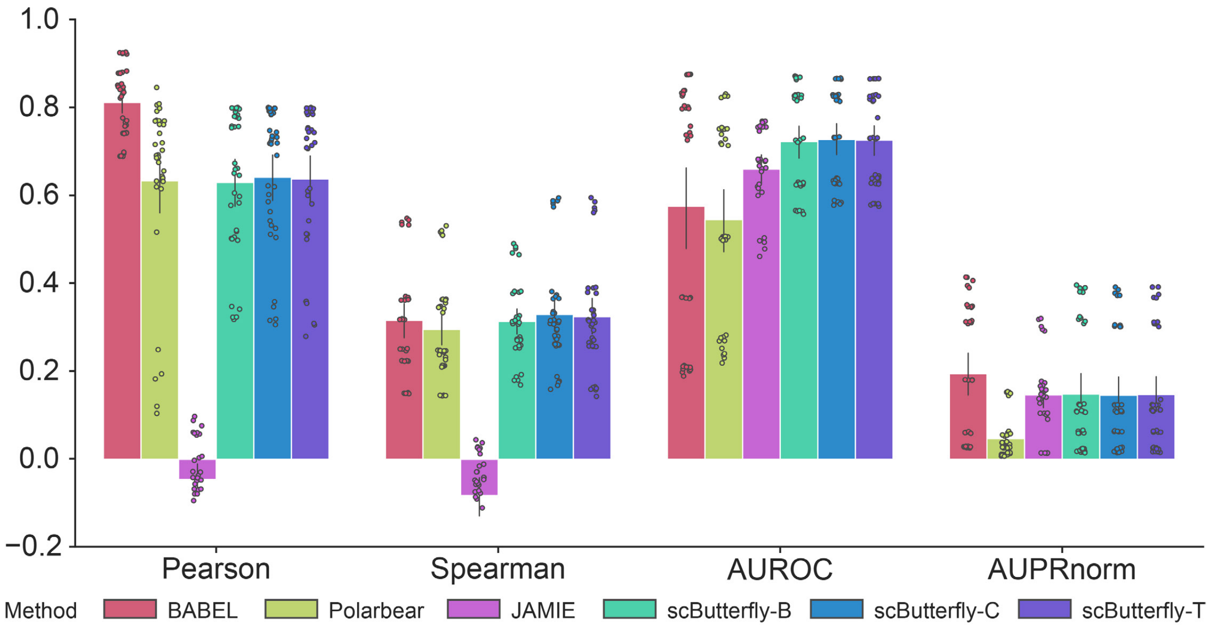

Supplementary Figure 34. Quantitative evaluation of the translated profiles for numerical accuracy in five-fold cross-validation by cell on the BMMC, MB, CL, MCC, MK, MDS and PBMC datasets (n = 35 cross-validations on seven datasets), via Pearson and Spearman correlation for RNA data and AUROC and AUPRnorm for ATAC data. The height of each bar denotes the median value of each metric and the error bars show 95% confidence interval. Note that the bars of JAMIE only contain 30 data points from six datasets since JAMIE encountered GPU memory errors on the BMMC dataset.

762

763

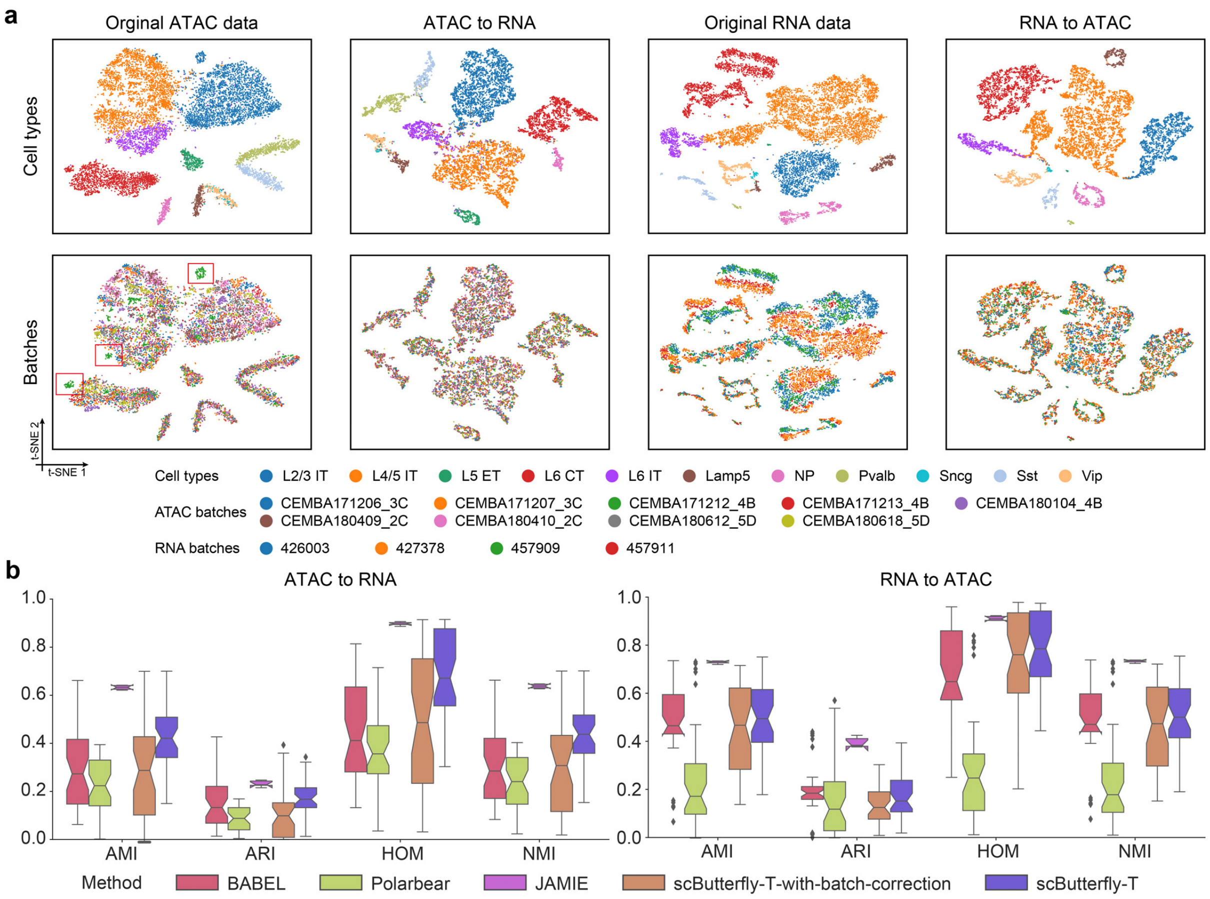

Supplementary Figure 35. a, t-SNE visualizations of cells in the first test fold of five-fold cross-validation by cell on the UP_MPMC dataset, using the original profiles and the profiles translated by scButterfly-T, colored by the cell types and batches. **b**, Evaluation of the cross-modality translation performance for unpaired data, including five-fold cross-validation on eight unpaired datasets of UP_HK, UP_MPMC, UP_eye, UP_muscle, UP_pancreas, UP_spleen, UP_stomach, and UP_thymus (n = 40 cross-validations on eight datasets), via cell clustering with metrics of AMI, ARI, HOM and NMI. Note that JAMIE encountered GPU memory errors on all the datasets except the UP_HK dataset. In the boxplots, the center lines, box limits, whiskers and notches denote the median, upper and lower quartiles, 1.5× interquartile range and 95% confidence interval calculated using a Gaussian-based asymptotic

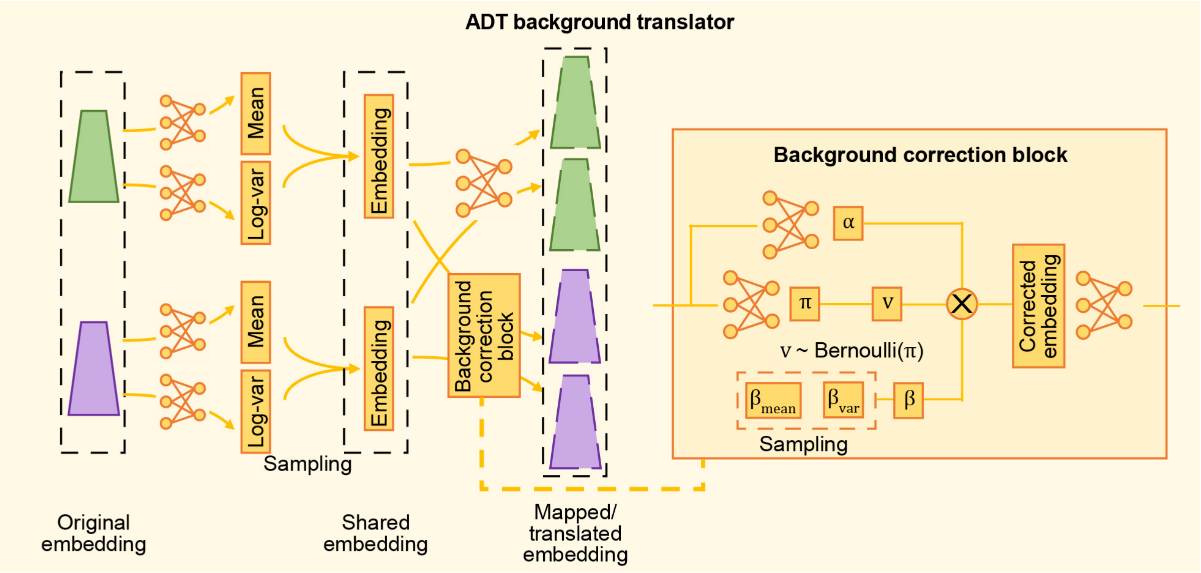

Supplementary Figure 36. Overview of scButterfly translator considered background signals in ADT data. ADT background translator contains a background correction block, modelling
766 the corrected embedding as a two-components mixture Gaussian distribution.

767

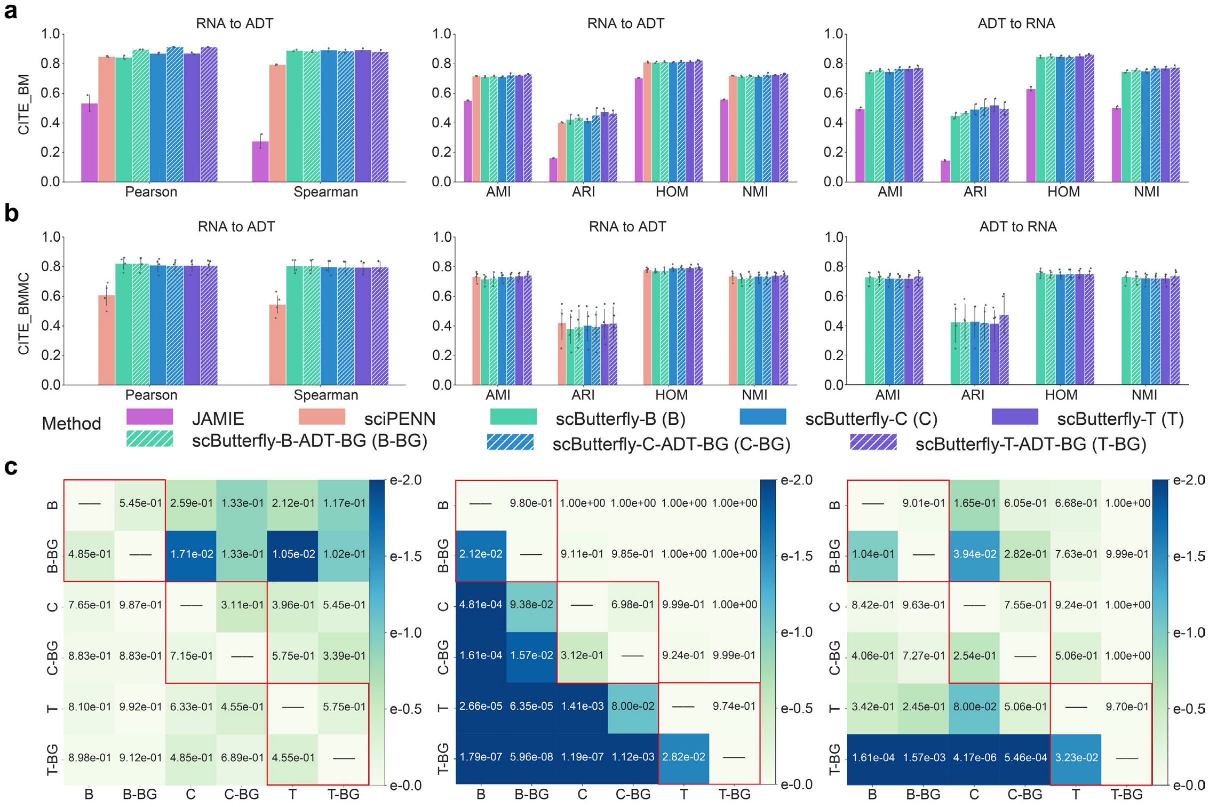

Supplementary Figure 37. a, **b**, Quantitative evaluation of the translation performance between transcriptome and proteome profiles in two-fold cross-validation by batch on the CITE_BM dataset (n = 2 cross-validations on 30672 cells) (**a**) and four-fold cross-validation by batch on the CITE_BMMC dataset (n = 4 cross-validations on 90261 cells) (**b**), via cell clustering with metrics of AMI, ARI, HOM, NMI and correlation with metrics of Pearson and Spearman correlation coefficients. The height of each bar denotes the median value of each metric and the error bars show 95% confidence interval. The scButterfly models with consideration about background are marked with white diagonal lines. Note that JAMIE encountered GPU memory errors on the CITE_BMMC dataset. **c**, Heatmap of p-values of one-sided paired Wilcoxon signed-rank tests (n = 24 evaluations on two datasets with four metrics). Each value in the heatmap indicates the significance of the advantage of a method (row) over another method (column). Red boxes emphasize the directly comparison between the variant considering background signals with its corresponding scButterfly model.

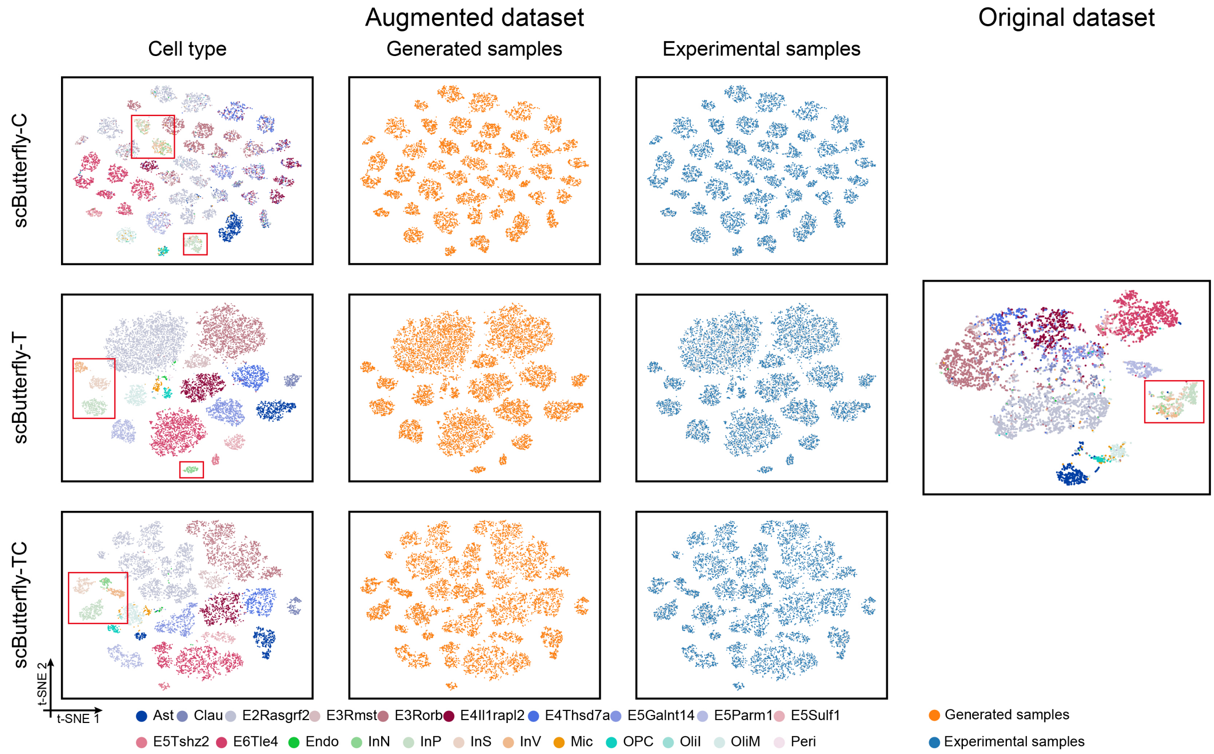

Supplementary Figure 38. Visualization of cells in the original dataset and the augmented datasets. t-SNE visualizations of cells in the first training fold of five-fold cross-validation on the MCC dataset, using the original dataset and the datasets augmented by scButterfly-C, scButterfly-T, and scButterfly-TC, respectively. In the Columns #2 and #3, the generated artificial samples and the experimental original samples are colored with yellow and blue, respectively. Note that the cell types of cells generated by scButterfly-C are labeled as consistent with the cell types of RNA data in the pairs, since scButterfly-C may pair the profiles of the cells of different cell types.

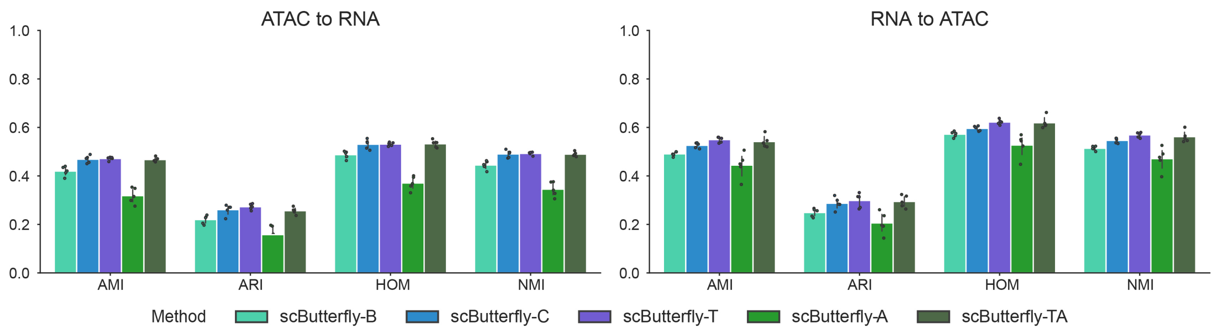

Supplementary Figure 39. Quantitative evaluation of the translated profiles for preserving cell heterogeneity. We evaluated the cross-validation performance on the MCC datasets via cell clustering with metrics of AMI, ARI, HOM, and NMI (n = 5 cross-validations on 9190 cells). The height of each bar denotes the median value of each metric and the error bars show 95% confidence interval.

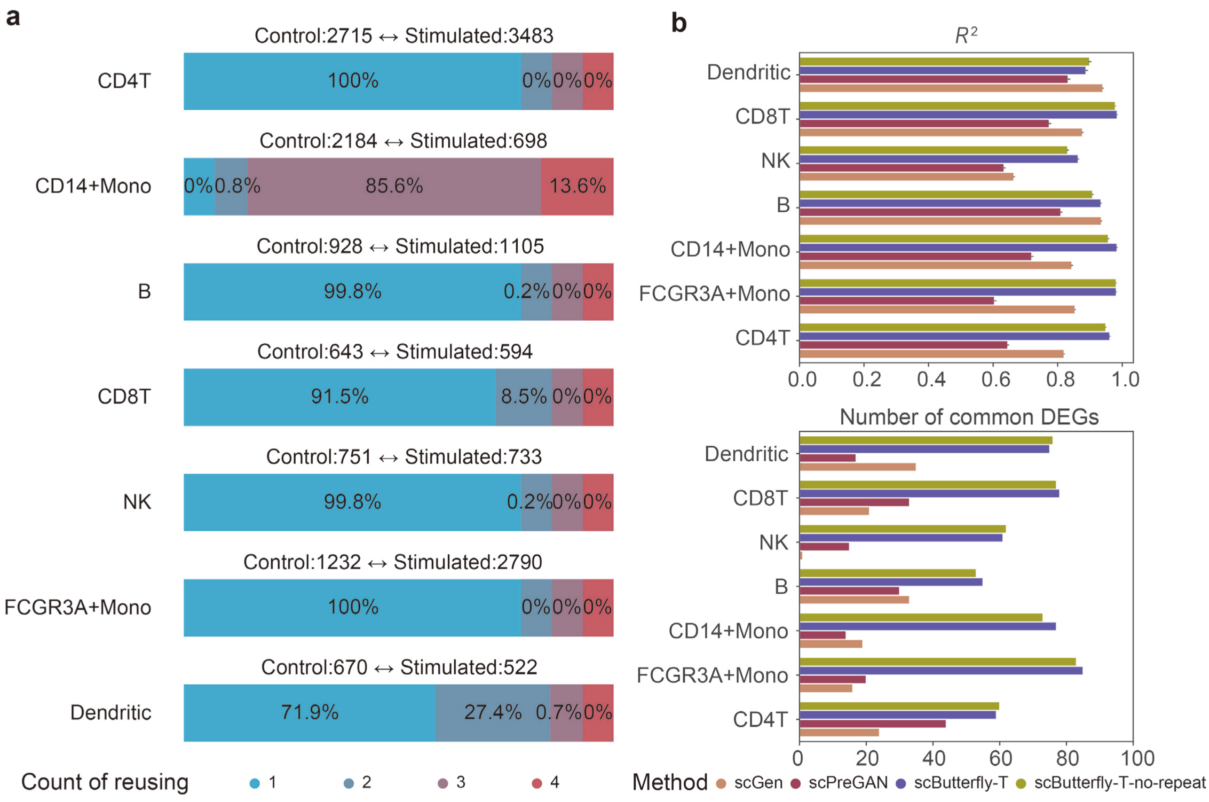

Supplementary Figure 40. a, Proportion of reusing for stimulated cells in sample generating in perturbation-response prediction, for each cell type on the PT_PBMC dataset. The counts of control cells and stimulated cells in original data are marked upon each bar. **b**, Performance of single-cell perturbation-response prediction on the PT_PBMC dataset, evaluated by the number of common DEGs of the top 100 (real) DEGs between the control data and the real stimulated data versus the top 100 (predicted) DEGs between the control data and the predicted stimulated data and the R^2 values for mean gene expression of the top 100 (real) DEGs between predicted and real stimulated cells for each cell type. Each bar contains 100 data points of R^2 values estimated using random subsampling at 80% The center values and error bars denote the mean and standard deviation, respectively.

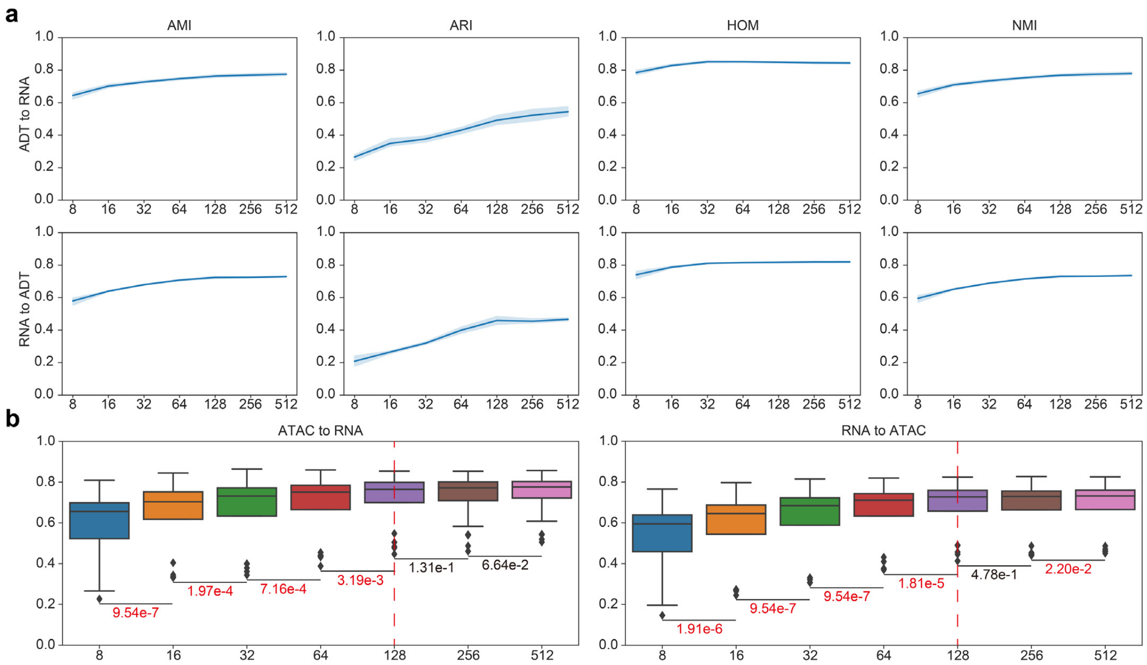

Supplementary Figure 41. **a**, Clustering performance on the CITE_BM dataset with different embedding dimensions evaluated by AMI, ARI, HOM and NMI (n = 5 cross-validations on 30672 cells). Each point on the lines denotes the median performance in five-fold cross-validation with different embedding dimensions, with error bands implying the 95% confidence interval. **b**, Overall clustering performance on the CITE_BM dataset with different embedding dimensions (n = 20 evaluations for five-fold cross-validation with four metrics). In the boxplots, the center lines, box limits and whiskers denote the median, upper and lower quartiles, and 1.5× interquartile range, respectively. The p-values of one-sided paired Wilcoxon signed-rank tests are shown under the boxes with the values under 0.05 marked with red, indicating the significance of the advantage of model with high embedding dimensions over that with low dimensions.

Supplementary Tables

Supplementary Table 1. The top five differentially expressed proteins (DEPs) of different cell types in the proteome profiles that were consecutively translated from epigenome to transcriptome and then to proteome based on the BMMC and CITE_BMMC datasets. The DEPs were obtained via one-sided Wilcoxon rank-sum tests and the proteins among the top five DEPs directly and mediately corroborated to the corresponding cell type are marked with dark grey and light grey, respectively.

Cell type	Top five differentially expressed proteins				
B1 B	CD32[58]	CD62L[59]	CD39[60]	CD35[61]	CD1c[62]
CD4+ T activated	CD194[63]	CD4	CD28[64]	CD5[65]	CD25[66]
CD4+ T naive	CD27[67]	CD5[68]	CD4	CD28[69]	CD278[70]
CD8+ T	KLRG1[71]	CD8	CD45[72]	HLA-E[73]	CD11a[74]
CD8+ T Naive	CD27[75]	CD5[76]	CD3[77]	TCR[76]	CD4[77]
CD14+ Mono	CD93	CD11b[78]	CD62P[79]	CD14	CD41
CD16+ Mono	CD172a[80]	CD11c[81]	CD85j[82]	CD119	CD13
Erythroblast	CD163[83]	CD23	CD154	CD83	CD223
G/M prog	CD31	CD112	CD155	CD49b	CD33[84]
HSC	CD112[85]	CD155[86]	CD49b[87]	CD31[88]	CD107a
Lymph prog	CD38[89]	HLA DR[90]	CD9[91]	CD24[92]	CD81[93]
MK/E prog	CD105[94]	CD82	CD112	CD274	CD107a
NK	CD122[95]	CD56[96]	CD335[97]	CD45RA[98]	CD244[99]
Naive CD20+ B	CD40[100]	CD22[101]	CD268	CD73[102]	CD185
Normoblast	CD158b	CD134	CD115	CD137	CD152
Plasma cell	CD39[103]	CD54[104]	CD62L[105]	CD146[106]	HLA ABC[107]
Proerythroblast	CD105	CD82[108]	CD274	CD49d[109]	CD71[109]
Transitional B	CD72	CD24[110]	CD19[111]	CD9[112]	HLA DR[113]
cDC2	CD123[114]	CD62L[115]	CD33	CD119	CD85j
pDC	CD123[116]	CD162[117]	FceRla	CD141[118]	CD304[119]

Supplementary Table 2. Pearson correlation coefficients between the clustering performance of original profiles and translated profiles. The Pearson correlation coefficients between the clustering performance of original profiles and translated profiles was calculated based on five-fold cross-validation on seven datasets (BMMC, MB, CL, MCC, MK, MDS, and PBMC) based on different clustering metrics. The highest value for each row is marked with bold.

Clustering metrics		Original RNA	Original ATAC
AMI	RNA to ATAC	**0.973**	0.754
	ATAC to RNA	0.844	**0.985**
ARI	RNA to ATAC	**0.880**	0.540
	ATAC to RNA	0.849	**0.882**
HOM	RNA to ATAC	**0.976**	0.725
	ATAC to RNA	0.851	**0.981**
NMI	RNA to ATAC	**0.970**	0.860
	ATAC to RNA	0.786	**0.989**

Supplementary References

1. Lu, J. et al. Learning under Concept Drift: A Review. *IEEE Transactions on Knowledge and Data Engineering* **31**, 2346-2363 (2019).
2. G. D, Y., Nair, N.G., Satpathy, P. & Christopher, J. Covariate Shift: A Review and Analysis on Classifiers. 2019 Global Conference for Advancement in Technology (GCAT) 1-6 (2019).
3. Biswas, A. & Mukherjee, S. Ensuring Fairness under Prior Probability Shifts. Proceedings of the 2021 AAAI/ACM Conference on AI, Ethics, and Society 414–424 (Association for Computing Machinery, Virtual Event, USA; 2021).
4. Tran, H.T.N. et al. A benchmark of batch-effect correction methods for single-cell RNA sequencing data. *Genome Biol.* **21**, 12 (2020).
5. Luecken, M.D. et al. Benchmarking atlas-level data integration in single-cell genomics. *Nat. Methods* **19**, 41-50 (2022).
6. Heumos, L. et al. Best practices for single-cell analysis across modalities. *Nat Rev Genet* **24**, 550-572 (2023).
7. Heryanto, Y.D., Zhang, Y.Z. & Imoto, S. Predicting cell types with supervised contrastive learning on cells and their types. *Sci. Rep.* **14**, 430 (2024).
8. Wang, S. et al. Leveraging the Cell Ontology to classify unseen cell types. *Nat. Commun.* **12**, 5556 (2021).
9. Xiong, Y.X., Wang, M.G., Chen, L. & Zhang, X.F. Cell-type annotation with accurate unseen cell-type identification using multiple references. *PLoS Comput. Biol.* **19**, e1011261 (2023).
10. Stuart, T. et al. Comprehensive Integration of Single-Cell Data. *Cell* **177**, 1888-1902.e1821 (2019).
11. Liu, J. et al. Jointly defining cell types from multiple single-cell datasets using LIGER. *Nat. Protoc.* **15**, 3632-3662 (2020).
12. Xu, C. et al. Probabilistic harmonization and annotation of single-cell transcriptomics data with deep generative models. *Mol. Syst. Biol.* **17**, e9620 (2021).
13. Lopez, R., Regier, J., Cole, M.B., Jordan, M.I. & Yosef, N. Deep generative modeling for single-cell transcriptomics. *Nat. Methods* **15**, 1053-1058 (2018).
14. Korsunsky, I. et al. Fast, sensitive and accurate integration of single-cell data with Harmony. *Nat. Methods* **16**, 1289-1296 (2019).
15. Fleming, S.J. et al. Unsupervised removal of systematic background noise from droplet-based single-cell experiments using CellBender. *Nat. Methods* **20**, 1323-1335 (2023).
16. Lotfollahi, M., Wolf, F.A. & Theis, F.J. scGen predicts single-cell perturbation responses. *Nat. Methods* **16**, 715-721 (2019).
17. Behdenna, A. et al. pyComBat, a Python tool for batch effects correction in high-throughput molecular data using empirical Bayes methods. *BMC Bioinformatics* **24**, 459 (2023).
18. Song, D. et al. scDesign3 generates realistic in silico data for multimodal single-cell and spatial omics. *Nat. Biotechnol.* **42**, 247-252 (2024).
19. Li, C., Chen, X., Chen, S., Jiang, R. & Zhang, X. simCAS: an embedding-based method for simulating single-cell chromatin accessibility sequencing data. *Bioinformatics* **39**, btad453 (2023).
20. Shengquan, C., Boheng, Z., Xiaoyang, C., Xuegong, Z. & Rui, J. stPlus: a reference-based method for the accurate enhancement of spatial transcriptomics. *Bioinformatics* **37**, i299-i307 (2021).
21. Yu, X., Xu, X., Zhang, J. & Li, X. Batch alignment of single-cell transcriptomics data using deep metric learning. *Nat. Commun.* **14**, 960 (2023).
22. Chen, H. et al. Assessment of computational methods for the analysis of single-cell ATAC-seq data.

Genome Biol. **20**, 241 (2019).
23. Chen, S. et al. RA3 is a reference-guided approach for epigenetic characterization of single cells. *Nat. Commun.* **12**, 2177 (2021).
24. Li, D., Ding, J. & Bar-Joseph, Z. Unsupervised cell functional annotation for single-cell RNA-seq. *Genome Res.* **32**, 1765-1775 (2022).
25. Baak, M., Koopman, R., Snoek, H. & Klous, S. A new correlation coefficient between categorical, ordinal and interval variables with Pearson characteristics. *Computational Statistics & Data Analysis* **152**, 107043 (2020).
26. Lähnemann, D. et al. Eleven grand challenges in single-cell data science. *Genome Biol.* **21**, 31 (2020).
27. Niven, E.B. & Deutsch, C.V. Calculating a robust correlation coefficient and quantifying its uncertainty. *Computers & Geosciences* **40**, 1-9 (2012).
28. Raymaekers, J. & Rousseeuw, P.J. Fast Robust Correlation for High-Dimensional Data. *Technometrics* **63**, 184-198 (2021).
29. Cusanovich, D.A. et al. A Single-Cell Atlas of In Vivo Mammalian Chromatin Accessibility. *Cell* **174**, 1309-1324.e1318 (2018).
30. Zhang, K. et al. A single-cell atlas of chromatin accessibility in the human genome. *Cell* **184**, 5985-6001.e5919 (2021).
31. Jones, R.C. et al. The Tabula Sapiens: A multiple-organ, single-cell transcriptomic atlas of humans. *Science* **376** (2022).
32. Elmentaite, R., Domínguez Conde, C., Yang, L. & Teichmann, S.A. Single-cell atlases: shared and tissue-specific cell types across human organs. *Nat. Rev. Genet.* **23**, 395-410 (2022).
33. Ashburner, M. et al. Gene ontology: tool for the unification of biology. The Gene Ontology Consortium. *Nat. Genet.* **25**, 25-29 (2000).
34. Gene Ontology, C. The Gene Ontology resource: enriching a GOld mine. *Nucleic Acids Res.* **49**, D325-D334 (2021).
35. Slowikowski, K., Hu, X. & Raychaudhuri, S. SNPsea: an algorithm to identify cell types, tissues and pathways affected by risk loci. *Bioinformatics* **30**, 2496-2497 (2014).
36. Li, H. et al. Inferring transcription factor regulatory networks from single-cell ATAC-seq data based on graph neural networks. *Nat. Mach. Intell.* **4**, 389-400 (2022).
37. Persad, S. et al. SEACells infers transcriptional and epigenomic cellular states from single-cell genomics data. *Nat. Biotechnol.* **41**, 1746-1757 (2023).
38. Fang, R. et al. Comprehensive analysis of single cell ATAC-seq data with SnapATAC. *Nat. Commun.* **12**, 1337 (2021).
39. Zhang, W., Jiang, R., Chen, S. & Wang, Y. scIBD: a self-supervised iterative-optimizing model for boosting the detection of heterotypic doublets in single-cell chromatin accessibility data. *Genome Biol.* **24**, 225 (2023).
40. Traag, V.A., Waltman, L. & van Eck, N.J. From Louvain to Leiden: guaranteeing well-connected communities. *Sci. Rep.* **9** (2019).
41. Ashuach, T. et al. MultiVI: deep generative model for the integration of multimodal data. *Nat. Methods* **20**, 1222-1231 (2023).
42. Wu, K.E., Yost, K.E., Chang, H.Y. & Zou, J. BABEL enables cross-modality translation between multiomic profiles at single-cell resolution. *Proc. Natl. Acad. Sci. U. S. A.* **118** (2021).
43. Cohen Kalafut, N., Huang, X. & Wang, D. Joint variational autoencoders for multimodal imputation and embedding. *Nat. Mach. Intell.* **5**, 631-642 (2023).

44. Zhang, R., Meng-Papaxanthos, L., Vert, J.-P. & Noble, W.S. Semi-supervised Single-Cell Cross-modality Translation Using Polarbear. Research in Computational Molecular Biology. (ed. I. Pe'er) 20-35 (Springer International Publishing, Cham; 2022).

45. Lütge, A. et al. CellMixS: quantifying and visualizing batch effects in single-cell RNA-seq data. *Life Sci Alliance* **4** (2021).

46. Stoeckius, M. et al. Simultaneous epitope and transcriptome measurement in single cells. *Nat. Methods* **14**, 865-868 (2017).

47. Dixit, A. et al. Perturb-Seq: Dissecting Molecular Circuits with Scalable Single-Cell RNA Profiling of Pooled Genetic Screens. *Cell* **167**, 1853-1866.e1817 (2016).

48. Liu, L. et al. Deconvolution of single-cell multi-omics layers reveals regulatory heterogeneity. *Nat. Commun.* **10** (2019).

49. Chen, S., Lake, B.B. & Zhang, K. High-throughput sequencing of the transcriptome and chromatin accessibility in the same cell. *Nat. Biotechnol.* **37**, 1452-1457 (2019).

50. Ma, S. et al. Chromatin Potential Identified by Shared Single-Cell Profiling of RNA and Chromatin. *Cell* **183**, 1103-1116.e1120 (2020).

51. Mulè, M.P., Martins, A.J. & Tsang, J.S. Normalizing and denoising protein expression data from droplet-based single cell profiling. *Nat. Commun.* **13**, 2099 (2022).

52. Govek, K.W. et al. Single-cell transcriptomic analysis of mIHC images via antigen mapping. *Sci. Adv.* **7** (2021).

53. Trong, T.N. et al. Semisupervised Generative Autoencoder for Single-Cell Data. *J. Comput. Biol.* **27**, 1190-1203 (2020).

54. Gayoso, A. et al. Joint probabilistic modeling of single-cell multi-omic data with totalVI. *Nat. Methods* **18**, 272-282 (2021).

55. Van Der Maaten, L. & Hinton, G. Visualizing data using t-SNE. *J. Mach. Learn. Res.* **9**, 2579-2625 (2008).

56. Gu, W., Tandon, A., Ahn, Y.Y. & Radicchi, F. Principled approach to the selection of the embedding dimension of networks. *Nat. Commun.* **12**, 3772 (2021).

57. Wolf, F.A., Angerer, P. & Theis, F.J. SCANPY: Large-scale single-cell gene expression data analysis. *Genome Biol.* **19** (2018).

58. Zhu, Z., Li, R., Li, H., Zhou, T. & Davis, R.S. FCRL5 exerts binary and compartment-specific influence on innate-like B-cell receptor signaling. *Proc. Natl. Acad. Sci. U. S. A.* **110**, E1282-E1290 (2013).

59. Hart, G.T., Wang, X., Hogquist, K.A. & Jameson, S.C. Kruppel-like factor 2 (KLF2) regulates B-cell reactivity, subset differentiation, and trafficking molecule expression. *Proc. Natl. Acad. Sci. U. S. A.* **108**, 716-721 (2011).

60. Kaku, H., Cheng, K.F., Al-Abed, Y. & Rothstein, T.L. A novel mechanism of B cell-mediated immune suppression through CD73 expression and adenosine production. *J. Immunol.* **193**, 5904-5913 (2014).

61. Erdei, A. et al. New aspects in the regulation of human B cell functions by complement receptors CR1, CR2, CR3 and CR4. *Immunol. Lett.* **237**, 42-57 (2021).

62. Xu, Y. et al. Human B1 Cells are the Main Blood Group A-Specific B Cells That Have a Moderate Correlation With Anti-A Antibody Titer. *Ann Lab Med* **40**, 48-56 (2020).

63. Sugiyama, D. et al. Anti-CCR4 mAb selectively depletes effector-Type FoxP3+CD4+ regulatory T cells, evoking antitumor immune responses in humans. *Proc. Natl. Acad. Sci. U. S. A.* **110**, 17945-17950 (2013).

64. Linterman, M.A. et al. CD28 expression is required after T cell priming for helper T cell responses and protective immunity to infection. *eLife* **3**, 1-21 (2014).

65. Matson, C.A. et al. CD5 dynamically calibrates basal NF-κB signaling in T cells during thymic development and peripheral activation. *Proc. Natl. Acad. Sci. U. S. A.* **117**, 14342-14353 (2020).
66. Kuniyasu, Y. et al. Naturally anergic and suppressive CD25+CD4+ T cells as a functionally and phenotypically distinct immunoregulatory T cell subpopulation. *Int. Immunol.* **12**, 1145-1155 (2000).
67. Withers, D.R. & Marriott, C.L. in Encyclopedia of Cell Biology, Vol. 3 770-775 (2016).
68. Freitas, C.M.T., Hamblin, G.J., Raymond, C.M. & Weber, K.S. Naïve helper T cells with high CD5 expression have increased calcium signaling. *PLoS ONE* **12** (2017).
69. Gigliotti, C.L. et al. Specific transcriptional programs differentiate ICOS from CD28 costimulatory signaling in human Naïve CD4+ T cells. *Front. Immunol.* **13** (2022).
70. Ariga, H. et al. Instruction of naive CD4+ T-cell fate to T-bet expression and T helper 1 development: roles of T-cell receptor-mediated signals. *Immunology* **122**, 210-221 (2007).
71. Herndler-Brandstetter, D. et al. KLRG1+ Effector CD8+ T Cells Lose KLRG1, Differentiate into All Memory T Cell Lineages, and Convey Enhanced Protective Immunity. *Immunity* **48**, 716-729.e718 (2018).
72. Cho, J.H. et al. CD45-mediated control of TCR tuning in naïve and memory CD8+ T cells. *Nat. Commun.* **7** (2016).
73. Hansen, S.G. et al. Broadly targeted CD8(+) T cell responses restricted by major histocompatibility complex E. *Science* **351**, 714-720 (2016).
74. Beyer, M. et al. The beta2 integrin CD11c distinguishes a subset of cytotoxic pulmonary T cells with potent antiviral effects in vitro and in vivo. *Respir. Res.* **6** (2005).
75. Derhovanessian, E. et al. Lower proportion of naive peripheral CD8+ T cells and an unopposed pro-inflammatory response to human Cytomegalovirus proteins in vitro are associated with longer survival in very elderly people. *Age* **35**, 1387-1399 (2013).
76. Ju, Y.J. et al. Self-reactivity controls functional diversity of naive CD8+ T cells by co-opting tonic type I interferon. *Nat. Commun.* **12** (2021).
77. Kivisakk, P. et al. Human cerebrospinal fluid central memory CD4+ T cells: evidence for trafficking through choroid plexus and meninges via P-selectin. *Proc. Natl. Acad. Sci. U. S. A.* **100**, 8389-8394 (2003).
78. Sander, J. et al. Cellular Differentiation of Human Monocytes Is Regulated by Time-Dependent Interleukin-4 Signaling and the Transcriptional Regulator NCOR2. *Immunity* **47**, 1051-1066 e1012 (2017).
79. Han, P. et al. Platelet P-selectin initiates cross-presentation and dendritic cell differentiation in blood monocytes. *Sci. Adv.* **6** (2020).
80. Corripio-Miyar, Y. et al. Phenotypic and functional analysis of monocyte populations in cattle peripheral blood identifies a subset with high endocytic and allogeneic T-cell stimulatory capacity. *Vet. Res.* **46** (2015).
81. Sugimoto, C. et al. Differentiation Kinetics of Blood Monocytes and Dendritic Cells in Macaques: Insights to Understanding Human Myeloid Cell Development. *J. Immunol.* **195**, 1774-1781 (2015).
82. Merlo, A. et al. Inhibitory receptors CD85j, LAIR-1, and CD152 down-regulate immunoglobulin and cytokine production by human B lymphocytes. *Clin. Diagn. Lab. Immunol.* **12**, 705-712 (2005).
83. Fabriek, B.O. et al. The macrophage CD163 surface glycoprotein is an erythroblast adhesion receptor. *Blood* **109**, 5223-5229 (2007).
84. Hahn, G., Jores, R. & Mocarski, E.S. Cytomegalovirus remains latent in a common precursor of dendritic and myeloid cells. *Proc. Natl. Acad. Sci. U. S. A.* **95**, 3937-3942 (1998).

85. Kaufmann, K.B. et al. A latent subset of human hematopoietic stem cells resists regenerative stress to preserve stemness. *Nat. Immunol.* **22**, 723-734 (2021).
86. Freistadt, M., Eberle, K.E., Huang, W. & Schwarzenberger, P. CD34+ hematopoietic stem cells support entry and replication of poliovirus: A potential new gene introduction route. *Cancer Gene Ther.* **20**, 201-207 (2013).
87. Somuncular, E. et al. CD49b identifies functionally and epigenetically distinct subsets of lineage-biased hematopoietic stem cells. *Stem Cell Rep.* **17**, 1546-1560 (2022).
88. Kim, S.W. et al. Human peripheral blood-derived CD31+ cells have robust angiogenic and vasculogenic properties and are effective for treating ischemic vascular disease. *J. Am. Coll. Cardiol.* **56**, 593-607 (2010).
89. Robin, C., Bennaceur-Griscelli, A., Louache, F., Vainchenker, W. & Coulombel, L. Identification of human T-lymphoid progenitor cells in CD34+CD38(low) and CD34+CD38+ subsets of human cord blood and bone marrow cells using NOD- SCID fetal thymus organ cultures. *Br. J. Haematol.* **104**, 809-819 (1999).
90. Boegel, S. et al. HLA and proteasome expression body map. *BMC Med. Genomics* **11** (2018).
91. Milburn, J.V. et al. Expression of CD9 on porcine lymphocytes and its relation to T cell differentiation and cytokine production. *Dev. Comp. Immunol.* **121**, 104080 (2021).
92. Israel, E. et al. Expression of CD24 on CD19- CD79a+ early B-cell progenitors in human bone marrow. *Cell. Immunol.* **236**, 171-178 (2005).
93. Luo, R.F. et al. CD81 protein is expressed at high levels in normal germinal center B cells and in subtypes of human lymphomas. *Hum. Pathol.* **41**, 271-280 (2010).
94. Mori, Y., Chen, J.Y., Pluvinage, J.V., Seita, J. & Weissman, I.L. Prospective isolation of human erythroid lineage-committed progenitors. *Proc. Natl. Acad. Sci. U. S. A.* **112**, 9638-9643 (2015).
95. Wu, Y., Tian, Z. & Wei, H. Developmental and functional control of natural killer cells by cytokines. *Front. Immunol.* **8** (2017).
96. Poli, A. et al. CD56bright natural killer (NK) cells: An important NK cell subset. *Immunology* **126**, 458-465 (2009).
97. Freud, A.G. et al. Expression of the activating receptor, NKp46 (CD335), in human natural killer and T-cell neoplasia. *Am. J. Clin. Pathol.* **140**, 853-866 (2013).
98. Krzywinska, E. et al. CD45 isoform profile identifies natural killer (NK) subsets with differential activity. *PLoS ONE* **11** (2016).
99. Agresta, L., Hoebe, K.H.N. & Janssen, E.M. The Emerging Role of CD244 Signaling in Immune Cells of the Tumor Microenvironment. *Front. Immunol.* **9**, 2809 (2018).
100. Marken, J., Muralidharan, S. & Giltiay, N.V. Anti-CD40 antibody KPL-404 inhibits T cell-mediated activation of B cells from healthy donors and autoimmune patients. *Arthritis Res. Ther.* **23** (2021).
101. Dörner, T., Shock, A., Goldenberg, D.M. & Lipsky, P.E. The mechanistic impact of CD22 engagement with epratuzumab on B cell function: Implications for the treatment of systemic lupus erythematosus. *Autoimmun. Rev.* **14**, 1079-1086 (2015).
102. Schena, F. et al. Dependence of immunoglobulin class switch recombination in B cells on vesicular release of ATP and CD73 ectonucleotidase activity. *Cell Reports* **3**, 1824-1831 (2013).
103. Dang, V.D. et al. CD39 and CD326 Are Bona Fide Markers of Murine and Human Plasma Cells and Identify a Bone Marrow Specific Plasma Cell Subpopulation in Lupus. *Front. Immunol.* **13** (2022).
104. Pojero, F. et al. Utility of CD54, CD229, and CD319 for the identification of plasma cells in patients with clonal plasma cell diseases. *Cytometry Part B - Clinical Cytometry* **90**, 91-100 (2016).

105. Medina, F., Segundo, C., Campos-Caro, A., González-García, I. & Brieva, J.A. The heterogeneity shown by human plasma cells from tonsil, blood, and bone marrow reveals graded stages of increasing maturity, but local profiles of adhesion molecule expression. *Blood* **99**, 2154-2161 (2002).

106. Liao, J. et al. Plasma Soluble CD146 as a Potential Diagnostic Marker of Acute Rejection in Kidney Transplantation. *Front. Med.* **7** (2020).

107. Yi, Q., Dabadghao, S., Österborg, A., Bergenbrant, S. & Holm, G. Myeloma bone marrow plasma cells: Evidence for their capacity as antigen-presenting cells. *Blood* **90**, 1960-1967 (1997).

108. Spring, F.A. et al. Tetraspanins CD81 and CD82 Facilitate α4β1-Mediated Adhesion of Human Erythroblasts to Vascular Cell Adhesion Molecule-1. *PLoS ONE* **8** (2013).

109. Cao, Y.A. et al. Heme oxygenase-1 deletion affects stress erythropoiesis. *PLoS ONE* **6**, e20634 (2011).

110. Simon, Q. et al. In-depth characterization of CD24highCD38high transitional human B cells reveals different regulatory profiles. *J. Allergy Clin. Immunol.* **137**, 1577-1584.e1510 (2016).

111. Cencioni, M.T., Ali, R., Nicholas, R. & Muraro, P.A. Defective CD19+CD24hiCD38hi transitional B-cell function in patients with relapsing-remitting MS. *Mult. Scler. Int.* **27**, 1187-1197 (2021).

112. Ai, X. et al. Expression of KAI1/CD82 and MRP-1/CD9 in transitional cell carcinoma of bladder. *Journal of Huazhong University of Science and Technology - Medical Science* **27**, 79-82 (2007).

113. Shabir, S. et al. Transitional B lymphocytes are associated with protection from kidney allograft rejection: a prospective study. *Am. J. Transplant.* **15**, 1384-1391 (2015).

114. Hasan, A. et al. Fatal COVID-19 is Associated with Reduced HLA-DR, CD123 or CD11c Expression on Circulating Dendritic Cells. *J. Inflamm. Res.* **15**, 5665-5675 (2022).

115. Tatsumi, N., Codrington, A.L., El-Fenej, J., Phondge, V. & Kumamoto, Y. Effective CD4 T cell priming requires repertoire scanning by CD301b(+) migratory cDC2 cells upon lymph node entry. *Sci. Immunol.* **6**, eabg0336 (2021).

116. Kutzner, H., Kerl, H., Pfaltz, M.C. & Kempf, W. CD123-positive plasmacytoid dendritic cells in primary cutaneous marginal zone b-cell lymphoma: Diagnostic and pathogenetic implications. *Am. J. Surg. Pathol.* **33**, 1307-1313 (2009).

117. Schuster, P. et al. Co-ordinated regulation of plasmacytoid dendritic cell surface receptors upon stimulation with herpes simplex virus type 1. *Immunology* **129**, 234-247 (2010).

118. Shih, T., Daip, J., Singh, S. & Fitzgerald-Bocarsly, P. ID: 221: “Super-pDCs”: A novel highly potent, CD141+ human blood plasmacytoid dendritic cell subtype. *Cytokine* **76**, 105 (2015).

119. Röck, J. et al. CD303 (BDCA-2) signals in plasmacytoid dendritic cells via a BCR-like signalosome involving Syk, Slp65 and PLCγ2. *Eur. J. Immunol.* **37**, 3564-3575 (2007).
