# Supplementary material for: scButterfly: a versatile single-cell cross-modality translation method via dual-aligned variational autoencoders
Source: Nat Commun. 2024 Apr 6;15:2973. doi: 10.1038/s41467-024-47418-x (PMC10998864; doi:10.1038/s41467-024-47418-x)
Supplement: Supplementary file 2 — Peer Review File [file 41467_2024_47418_MOESM2_ESM.pdf]

nature portfolio

Peer Review File

scBeutterfly: a versatile single-cell cross-modality translation method via dual-aligned variational autoencoders

Open Access This file is licensed under a Creative Commons Attribution 4.0 International License, which permits use, sharing, adaptation, distribution and reproduction in any medium or format, as long as you give appropriate credit to the original author(s) and the source, provide a link to the Creative Commons license, and indicate if changes were made. In the cases where the authors are anonymous, such as is the case for the reports of anonymous peer reviewers, author attribution should be to 'Anonymous Referee' followed by a clear attribution to the source work. The images or other third party material in this file are included in the article's Creative Commons license, unless indicated otherwise in a credit line to the material. If material is not included in the article's Creative Commons license and your intended use is not permitted by statutory regulation or exceeds the permitted use, you will need to obtain permission directly from the copyright holder. To view a copy of this license, visit http://creativecommons.org/licenses/by/4.0/.

Please note that Reviewer #1 co-reviewed this manuscript with one of the reviewers who provided the listed reports as part of the Nature Communications initiative to facilitate training in peer review and appropriate recognition for co-reviewers.

Reviewer #2 (Remarks to the Author):

In this manuscript, the authors proposed scButterfly, a single-cell cross-modality translation method based on variational autoencoders with a data augmentation approach. The authors demonstrate that scButterfly can outperform the competing methods in cross-modality translation for both paired and unpaired single-cell data, as well as for mRNA + ATAC and mRNA + ADT single-cell data. The authors also show scButterfly's ability in perturbation-response prediction. However, many technological problems should be explained and addressed before considering this work for publication.

Major:
In data argumentation (and also some other analyses), the authors randomly pair two omics of the cells in the same cell types. I wonder if we also bring some noise by using this approach because there is also a high heterogeneity within a cell type. The authors should explain if this is a potential problem for the proposed algorithm.

In the section "unpaired data training with scButterfly", the authors directly paired the data from different batches and different omics. I wonder if we need to consider the batch effect between two batches before pairing them. The authors should explain why there is no need to consider the batch effect here or add the batch correction step.

In the CITE-seq analyses, did the authors consider the background signal of the ADT data (referring to TotalVI)? If the background signal was not considered, please add an experiment to show that it is unnecessary to distinguish the foreground and background signals. Besides, some ADT data have very low dimensions, such as 10, but the model projects them into 128. The authors may consider using a more flexible model structure for ADT data.

How did the authors set the hyperparameters, such as setting w_r=1, w_a=2, w_elbo=20/I_r (w_elbo=20/I_r + 20/I_a in total loss) and w_dis=1. Is the model sensitive to these parameters? The authors should provide some sensitivity analyses.

The authors show many clustering results on the RNA to ATAC data and ATAC to RNA data. In most cases, the RNA to ATAC data has a better performance than the ATAC to RNA data. The authors should explain this. Besides, are there any biological insights in doing the cell clustering from both RNA to ATAC and ATAC to RNA? I think we just need to find the best way to do this task.

I believe the proposed model is not only for cell type annotation. Can we also do any ATAC-specific analyses, such as TF-target gene regulatory network inference, from the output of the model? That would be more meaningful and provide more biological insights.

Minor:
In the introduction, we authors provide some reasons/motivations for developing the model. However, it seems that some motivations are very weak, and some problems have been addressed by one or more previous methods. Specifically:

"Second, an effective data augmentation scheme is in demand to better tackle the limited number of cells measured by multi-omics protocols and uncover cell heterogeneity in highly noisy data of complex tissues.". This is an optional, not necessary, function in the model. So, this reason/motivation is very weak. It would be better if authors could show the necessity of doing this approach and provide some evidence.

"Third, there is a prevalent challenging scenario where the single-modality data to be translated has inter-sample variations (e.g., batch effects) and even different biological contexts (e.g., novel cell types) compared to the data used for training". These problems, such as batch correction, have been addressed, to some extent, by many previous methods. So, this reason/motivation is

also very weak. It is advisable to highlight potential issues with previous methods concerning batch correction.

“Fifth, extensive applications of cross-modality translators have not been fully investigated, such as integrative analysis of single-modality data with its predicted profiles of other modalities, enhancement of single-cell multi-omics data, cell type annotation of challenging modalities such as scATAC-seq data, and consecutive translations among multiple modalities.” Many previous methods can do one or more of these tasks. Besides, the authors mentioned “cell type annotation of challenging modalities such as scATAC-seq data”. This is confusing because scATAC-seq is not good data for cell type annotations. After integrating mRNA and ATAC data, we can directly use mRNA to identify cell types. Of course, the authors can do these to demonstrate the model performance, but I wonder if it provides any biological insights.

In the equation of L_D, the authors stated that “randomly assigning target labels 0 and 1 to the values following uniform distribution.” What is the “values” mentioned here? How to connect this sentence to the equation? The authors should make these contents more understandable.

In the section “Single-cell perturbation-response prediction with scButterfly”. What if multiple control cells pair with the same stimulated cell? Did the authors reuse the same cell multiple times? The authors should provide these details in the method section.

The authors pretrained the model for 100 epochs and integrative trained the model for 200 epochs with early stops. What are the criteria for the early stops? When will the model converge (in pretraining and integrative training)? The authors should provide more details.

In Fig 1, there are two windows of the translator: a brief window in the butterfly and a detailed window aside. However, the first one has two outputs and the second one has four outputs. This is confusing. I recommend that the authors ensure consistency or provide an explanation in the legend.

Reviewer #3 (Remarks to the Author):

For a technical perspective, there is no difference in the method between this paper and the other paper (published on https://proceedings.neurips.cc/paper_files/paper/2022/hash/662b1774ba8845fc1fa3d1fc0177ceeb-Abstract-Conference.html), although the proposed scbutterfly is applied to multiple datasets.

1. What distinguishes the proposed scButterfly from the paper titled 'Cross-Linked Unified Embedding for Cross-Modality Representation Learning'? It appears that both methods are grounded in variational autoencoders, and the translation is accomplished through cross-domain autoencoders within the latent/semantic feature space.
2. The incorporation of multi-omics data poses a challenge due to its high dimensionality and technical variability. How can you ascertain scButterfly's ability to handle high-dimensional multi-omics data? It is recommended to validate the model using varying numbers of features.
3. Regarding the data augmentation method, it is advisable to analyze its effectiveness. For instance, using a single multi-omics single-cell dataset, the author should demonstrate and compare the model's performance with and without data augmentation. Furthermore, it's essential to specify how many augmented pairs/data points were generated and if there is any impact on model performance with varying numbers of augmented data.
4. On line 783, it is stated that NVIDIA supports TensorFlow 1.13 for RTX 6000. However, NVIDIA provides an NGC docker with pre-compiled TensorFlow 1.15 for use on newer GPUs.

Reviewer #4 (Remarks to the Author):

The author has introduced a framework named scButterfly, with a primary focus on cross-modality

translation, particularly between transcriptome and chromatin accessibility data. The scButterfly framework comprises 7 key components: 2 encoders, 2 decoders, 1 translator, and 2 discriminators. To facilitate cross-omics translation, the framework aligned omic-specific latent spaces using the translator, in contrast to previous methods that projected them into a single shared latent space. Moreover, the author has developed two model variants scButterfly-T and scButterfly-C aimed at expanding sample size, enhancing data quality, and enabling the translation of unpaired data. To evaluate the model's performance, the author conducted a series of downstream experiments, including cell clustering on paired and unpaired data, cross-modality translation involving novel cell types and batches, data enhancement using the predicted profiles, perturbation response prediction, and consecutive translation.

The following are my comments.

1. In line 254, the author identified two subgroups of basal cells based on the predicted profiles. However, it remains unclear how these two subgroups were identified. In supplementary Figure 10a, cells within cluster 5 are divided into two parts, yet they have been labeled as subgroup2 rather than subgroup1. Is there any special reason or rationale behind this labeling choice?

2. In line 264, it remains unclear what biological features distinguish these two subgroups. The enriched pathways in both subgroups are related to basal cells, which is expected as they are all basal cells. Are the enriched pathways in two subgroups related to different functions, or different developmental stages of basal cells? Please provide additional information to better explain the distinctions between these two subgroups.

3. Additionally, the author should color the tSNE by batches in Fig3d. The two subgroups may potentially be attributed to different batch effects rather than biological variation.

4. When evaluating batch effects removal on the multi-batches dataset, the author should also report numeric evaluation metrics such as k-nearest-neighbor batch-effect test (kBET) and Graph Connectivity (GC).

5. For the translation on paired datasets, the author should also report correlations for RNA and ATAC data.

6. Line 224, Fig.2b should be Fig.3b

7. In Fig. 2a, 3c, it’s hard to find cell types mentioned in the main text. Please highlight.

8. Since the ADT data dimension is low, it would be beneficial to visualize the translated ADT data pattern (such as heatmap) and compare it with the true data pattern.

To the reviewers,

We want to further thank the four reviewers for their time reviewing and their constructive comments on our manuscript entitled “scButterfy: a versatile single-cell cross-modality translation method via dual-aligned variational autoencoders”. We have thoroughly revised and enhanced our manuscript based on their comments. In addition to the minor changes, we have made the following six major modifications in our revised manuscript, with the modifications highlighted in red. These changes are summarized in Figs. 1-5, Supplementary Texts 1-12, Supplementary Figs. 3-14, 16, 18, 21-33, Supplementary Table 2, as well as the main text and Methods section.

1) We conducted a comprehensive sensitivity analysis on scButterfly, including the four weights of the loss function calculation, feature counts, pretraining and integrative training epochs, and early-stop patience. scButterfly achieved stable and satisfactory translation performance across various hyperparameter settings, implying its powerful robustness to these hyperparameters (Supplementary Text 7, Supplementary Figs. 26, 27).

2) We conducted a series of detailed analysis of the effectiveness of the data augmentation strategy, showcased the powerful capability of data augmentation in tackling the issues with limited samples (Fig. 2d), and discussed the impact of augmented sample counts on translation performance (Supplementary Text 5, Supplementary Fig. 24). Additionally, we designed a variant of scButterfly, namely scButterfly-TC, to further reduce the additional noise introduced by data augmentation (Supplementary Text 4, Supplementary Fig. 23).

3) We provided more experimental details on the RNA-specific Gene Ontology (GO) analysis and more information about the biological distinctions between the identified subgroups. Additionally, we further included ATAC-specific analysis, including single-nucleotide polymorphisms (SNPs) enrichment analysis and TF regulatory network inference, highlighting the powerful potential of scButterfly for revealing biological insights (Supplementary Figs. 12-14).

4) We discussed the ability of scButterfly to perform cross-modality translation on multi-batch datasets, and further analyzed the impact of adding batch effects correction step before data augmentation. The results demonstrated that scButterfly already holds the potential of not being significantly affected by batch effects (Fig. 3, Supplementary Texts 1, 9, Supplementary Figs. 8-10, 21, 29).

5) For the CITE-seq data analyses, we added the consideration for distinguishing the foreground and background signals in the ADT data and designed a variant of scButterfly-B based on the two-component Gaussian mixture model, named scButterfly-B-ADT-BG, as an optional variant for users (Supplementary Text 10, Supplementary Figs. 30, 31). Moreover, we discussed the impact of different embedding dimensions on the performance of translation between the RNA and ADT data and demonstrated that the default setting provides a balance between efficiency and effectiveness (Supplementary Text 12, Supplementary Fig. 33).

6) In the introduction, we reorganized our description to ensure rigorous formulations of motivations. Besides, we also refined our figures to improve readability and double-checked the description for multiple parts to avoid misunderstandings.

Next, we will clarify the comments raised by the reviewers point-by-point, with the reviewers' points in black and our responses in blue.

REVIEWER #1

Please note that Reviewer #1 co-reviewed this manuscript with one of the reviewers who provided the listed reports as part of the Nature Communications initiative to facilitate training in peer review and appropriate recognition for co-reviewers.

RESPONSE

We sincerely appreciate the reviewer for co-reviewing our manuscript. We have carefully considered all the comments raised by the reviewers point-by-point, and made revisions in the corresponding section of the manuscript.

REVIEWER #2

In this manuscript, the authors proposed scButterfly, a single-cell cross-modality translation method based on variational autoencoders with a data augmentation approach. The authors demonstrate that scButterfly can outperform the competing methods in cross-modality translation for both paired and unpaired single-cell data, as well as for mRNA + ATAC and mRNA + ADT single-cell data. The authors also show scButterfly's ability in perturbation-response prediction. However, many technological problems should be explained and addressed before considering this work for publication.

RESPONSE

We express our sincere gratitude to the reviewer for the constructive comments which helped us to improve the manuscript. We have carefully considered and answered every comment raised by the reviewer and made revisions to the manuscript correspondingly.

COMMENT 1

In data argumentation (and also some other analyses), the authors randomly pair two omics of the cells in the same cell types. I wonder if we also bring some noise by using this approach because there is also a high heterogeneity within a cell type. The authors should explain if this is a potential problem for the proposed algorithm.

RESPONSE TO COMMENT 1

We appreciate the reviewer for this thoughtful comment. Firstly, we deeply agree with the reviewer that randomly pairing two omics of the cells in the same cell type may bring some additional noise. However, the level of heterogeneity within one cell type is usually smaller than the heterogeneity between different cell types. We visualized the seven paired datasets (BMMC, MB, CL, MCC, MK, MDS, and PBMC) with t-distributed stochastic neighbor embedding[1] (t-SNE). As shown in Fig. R1, cells of the same type in the original data generally tend to form tightly clustered groups, while cells of different types are more likely to be loosely distributed in the visualizations. Therefore, we believe that these noises may not have a significant impact on the learning for the cell type-specific characteristics of scButterfly.

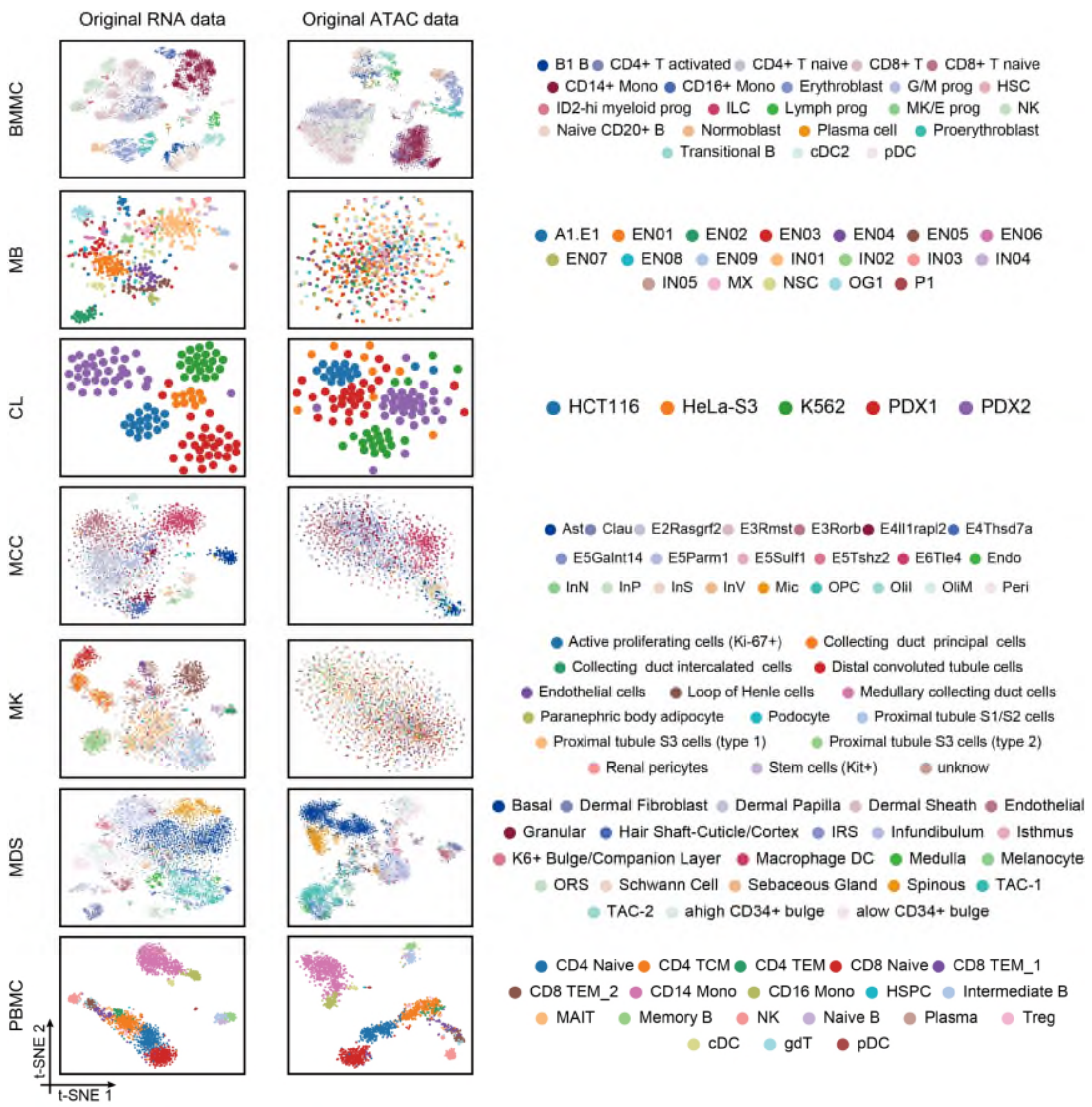

Fig. R1 | Visualization of the cells in multiple datasets. t-SNE visualizations of cells in the first test fold of five-fold cross-validation on the seven datasets (BMMC, MB, CL, MCC, MK, MDS, and PBMC), using the original RNA profiles and ATAC profiles.

Secondly, the data augmentation strategy mentioned by the reviewer is based on the cell-type labels, namely scButterfly-T (**T**ype) in the manuscript. As the reviewer pointed out, data augmentation based on cell-type labels may lead to additional noise in some types with a large number of cells. Thus, in addition to scButterfly-T, we also proposed scButterfly-C (**C**luster) with data augmentation based on cluster labels, which may have the potential ability to address this concern. The scButterfly-C variant incorporates data augmentation by randomly pairing two omics of the cells within the same Leiden[2] clusters. Furthermore, we have employed a high resolution of 3 to further increase the number of clusters, thereby reducing the number of cells in each cluster, enhancing the similarity among cells within the same clusters, and consequently

reducing the noise introduced by data augmentation. Therefore, scButterfly-C has the potential ability to balance the number of cells in each cluster compared to scButterfly-T and thereby reducing the differences of cells in each cluster.

Thirdly, to further reduce the additional noise introduced in data augmentation, we separately performed clustering on cells of each cell type and then conducted data augmentation by randomly pairing two omics of the cells in the same cluster, resulting in a variant of scButterfly named scButterfly-TC (**T**ype **C**luster). Specifically, we partitioned all training samples by cell-type labels and trained a MultiVI[3] model with default settings for each cell type to obtain joint cell embeddings based on RNA and ATAC profiles. We then performed Leiden clustering to obtain refined clusters for each cell type. Finally, scButterfly-TC utilized these refined clustering labels to perform data augmentation, thus avoiding introducing too much additional noise when pairing two omics of the cells in the same cell types (scButterfly-T). We conducted five-fold cross-validations by cell on the BMMC, MB, CL, MCC, MK, MDS, and PBMC datasets to evaluate the performance of scButterfly-TC, the basic scButterfly model (scButterfly-B) and the other two variants（scButterfly-T and scButterfly-C）. As shown in Fig. R2, scButterfly-TC performed similarly to scButterfly-T in the translation from ATAC to RNA. However, scButterfly-TC performed slightly worse than scButterfly-T for the translation from RNA to ATAC, indicating that decreasing the additional noise may not lead to an improvement in performance. Considering that scButterfly-T is simpler and more user-friendly compared to scButterfly-TC, we still recommend that users perform cross-modality translation with scButterfly-T. Even so, we also provide the

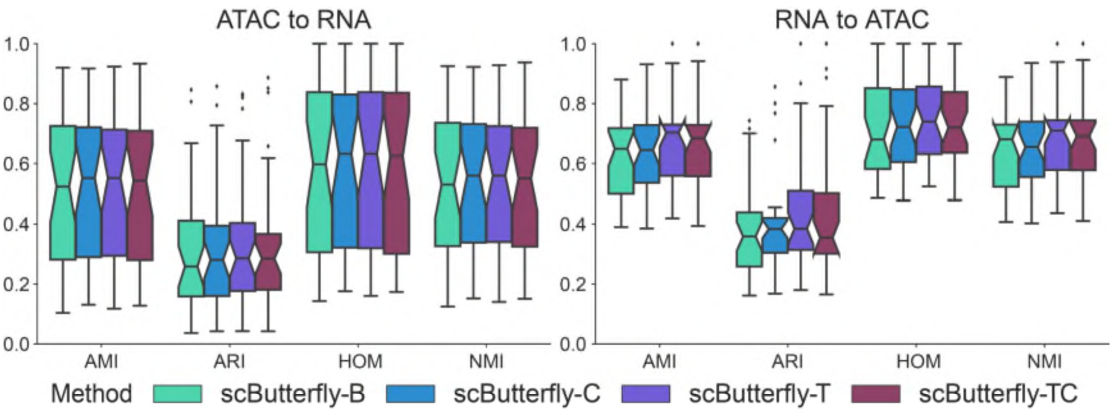

Fig. R2 | Cross-modality translation performance of scButterfly-TC. Quantitative evaluation of the translated profiles for preserving cell heterogeneity. We evaluated the cross-validation performance on the seven datasets (BMMC, MB, CL, MCC, MK, MDS, and PBMC) via cell clustering with metrics of AMI, ARI, HOM, and NMI. In the boxplots, the center lines, box limits, whiskers and notches denote the median, upper and lower quartiles, 1.5× interquartile range and 95% confidence interval calculated using a Gaussian-based asymptotic approximation, respectively.

scButterfly-TC as an optional variant. Users could choose to use scButterfly-TC according to the situation of various datasets, especially for the datasets only with coarse labels.

In summary, we first clarify that although pairing two modalities of the cells in the same cell types may introduce some additional noise, such noise can be deemed negligible compared to the variations between different cell types. Furthermore, scButterfly does not necessarily use cell-type labels to match two omics, since the scButterfly-C model uses clustering labels instead, which holds the potential to avoid being affected by high heterogeneity within the same type. Moreover, the performance of scButterfly-TC shows that the noise caused by randomly matching cells from the same cell types may not lead to a degradation in translation performance. We have added these discussions in Section "scButterfly enables cross-modality translation while preserving cell heterogeneity" and Supplementary Text 4, and provided scButterfly-TC for users in scenarios with coarse cell-type labels.

COMMENT 2

In the section "unpaired data training with scButterfly", the authors directly paired the data from different batches and different omics. I wonder if we need to consider the batch effect between two batches before pairing them. The authors should explain why there is no need to consider the batch effect here or add the batch correction step.

RESPONSE TO COMMENT 2

We appreciate the reviewer for this insightful comment. We strongly agree with the reviewer that considering the batch effects holds immense significance. We have considered whether to take batch effects into account when designing the scButterfly framework for unpaired data. However, we ultimately abandoned this consideration based on the following reasons:

Firstly, we deeply agree that the batch effects could lead to systematic variations and additional noise in high-throughput sequencing. However, although batch effects can be indeed observed in downstream analysis, we believe that batch effects have a limited impact on our data augmentation strategy. The inter-batch heterogeneities are often smaller compared to the inter-cell-type heterogeneities, especially in the datasets with batch effects attributed to media storage or patients[4]. Taking the UP_MPMC

dataset as an example, we visualized the original RNA and ATAC profiles of the cells in the first fold of the five-fold cross-validation with t-SNE. As shown in Columns #1 and #3 in Fig. R3a, batch effects could cause poor mixing or even separation of cells within a part of cell types, especially for the original RNA data. However, most of the cells of the same cell type tend to cluster together in the t-SNE visualizations. These results indicate that the variants from batch effects are usually smaller than the variants from the cell type.

Secondly, we observed that scButterfly for unpaired data translation was generally less affected by batch effects to some extent. In Section "scButterfly can be generalized to unpaired data training and perturbational analysis" of the manuscript, we designed a

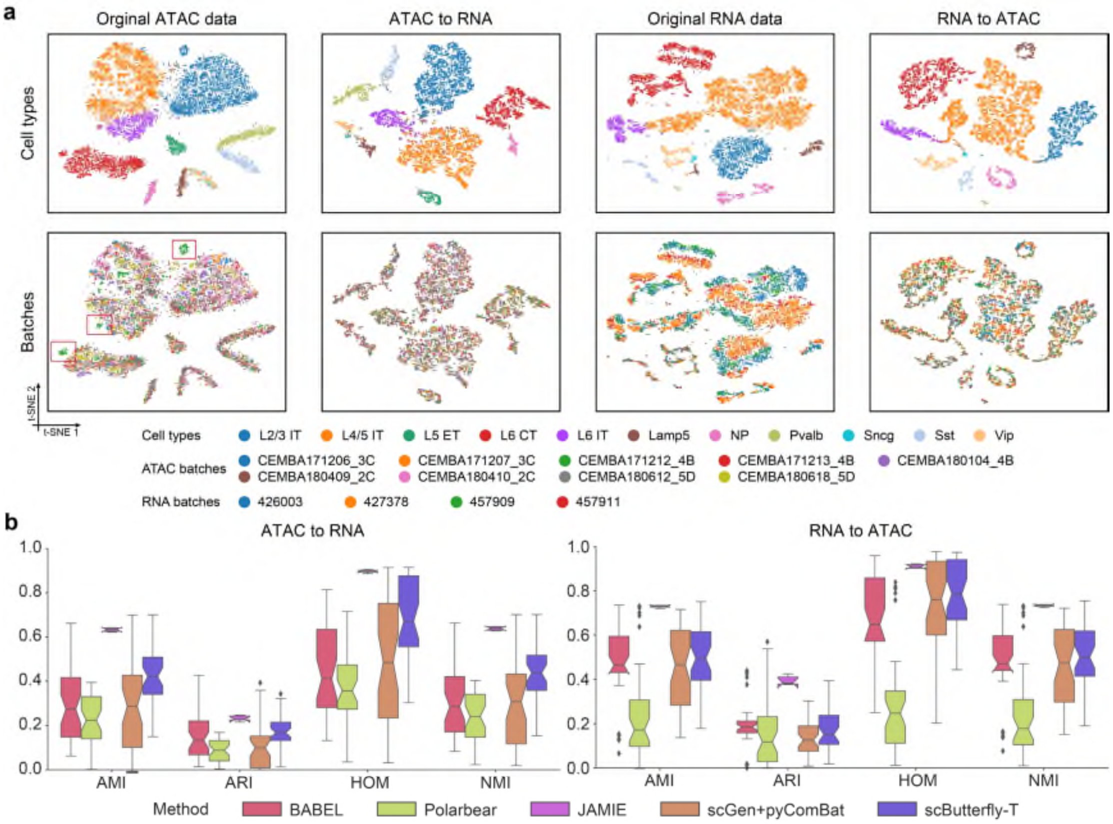

Fig. R3 | Potential ability to be less affected by batch effects for scButterfly and the limitations of batch correction methods. a, t-SNE visualizations of cells in the first test fold of five-fold cross-validation by cell on the UP_MPMC dataset, using the original profiles and the profiles translated by scButterfly-T, colored by the cell types and batches. **b**, Evaluation of the cross-modality translation performance for unpaired data, including five-fold cross-validation on eight unpaired datasets of UP_HK, UP_MPMC, UP_eye, UP_muscle, UP_pancreas, UP_spleen, UP_stomach, and UP_thymus, via cell clustering with metrics of AMI, ARI, HOM and NMI. Note that JAMIE encountered GPU memory errors on all the datasets except the UP_HK dataset. In the boxplots, the center lines, box limits, whiskers and notches denote the median, upper and lower quartiles, 1.5× interquartile range and 95% confidence interval calculated using a Gaussian-based asymptotic approximation, respectively.

method similar to the scButterfly-T variant for translating the unpaired data, which matches cells of the same type to generate artificial paired training samples. For other baseline methods, we performed the same matching to make cross-modality translations. It is worth noting that we did not require the matched samples to come from the same batch, since in the unpaired datasets, batches of one modality data often have no overlap with that of another modality. Taking the UP_MPMC dataset as an illustration again, the RNA data contains 4 batches, while the ATAC data contains different 9 batches as shown in Columns #1 and #3 in Fig. R3a. Therefore, the scButterfly model was trained to translate samples from different batches, which has given the model the potential ability to be not significantly affected by batch effects. The experiment results in Fig. R3a also support this point. In the original ATAC data (Column #1 in Fig. R3a), some samples of Batch #3 exhibit variations compared to other samples (marked with red boxes), while in translated profiles from ATAC to RNA (Column #2 in Fig. R3a), all batches are well mixed, indicating that scButterfly could be insignificantly impacted by the batch effects. For the more challenging original RNA data (Column #3 in Fig. R3a), where batch effects are more pronounced, RNA to ATAC translation (Column #4 in Fig. R3a) also mixes samples from different batches, implying the potential ability of the model to confront batch effects.

Thirdly, the existing batch correction methods may have some limitations[5, 6]: most existing methods are likely to simultaneously lose some biological heterogeneity when correcting the batch effects, presenting the trade-off between the preservation of biological variation and the mixing of batches. Moreover, most batch correction methods perform dimensionality reduction on data and output low-dimensional embeddings instead of the count matrixes, resulting in the inability to use the original dimensionality data for downstream analysis. To further characterize the impact of batch correction, we added a batch correction step before matching the cells with the same cell type. As suggested in [5], scGen[7] and pyComBat[8] could achieve the best overall performance among the batch correction methods which could output the corrected original-dimensionality count matrices for RNA and ATAC, respectively. We chose these two state-of-the-art batch correction methods to respectively correct the batch effects in the RNA and ATAC data before data augmentation. As shown in Fig. R3b, the combination of scGen and pyComBat led to a decrease in translation performance, revealing that the batch correction before data augmentation may not be suitable for unpaired data translation.

In conclusion, we observed that the variants from batch effects are usually smaller than the variants from the cell type and have a limited impact on data augmentation. Besides, we highlighted that the scButterfly model for unpaired data translation enables itself to be less affected by the batch effects. Additionally, we supposed that there may still be some limitations for current batch correction methods, and introducing the batch correction step might lead to over-correction. We would like to express our gratitude to the reviewer again for the constructive comment, which helped us to rethink the rationality of batch correction in the unpaired data translation. We have added the discussion about the batch effects in the translation for the unpaired data in Section "scButterfly can be generalized to unpaired data training and perturbational analysis" and Supplementary Text 9.

COMMENT 3

In the CITE-seq analyses, did the authors consider the background signal of the ADT data (referring to TotalVI)? If the background signal was not considered, please add an experiment to show that it is unnecessary to distinguish the foreground and background signals. Besides, some ADT data have very low dimensions, such as 10, but the model projects them into 128. The authors may consider using a more flexible model structure for ADT data.

RESPONSE TO COMMENT 3

We appreciate the reviewer for this constructive comment, helping us rethink and improve the model structure for ADT data. In response to this comment, we first developed a variant namely scButterfly-B-ADT-BG based on scButterfly-B, which incorporated the step for distinguishing the foreground and background signals in ADT data. We conducted cross-validation on the CITE_BMMC and CITE_BM datasets to validate the performance of scButterfly-B-ADT-BG. Additionally, we discussed the impact of the embedding dimensions on the translation performance in CITE-seq data analyses. The detailed explanations in terms of these two points are illustrated in the subsequent paragraphs.

For the first point, we deeply value the insightful commentary provided by the reviewer and sincerely apologize for not considering distinguishing the foreground and background signals in ADT data. The background signal is a kind of noise that widely appears in many recently introduced droplet-based single-cell assays[9], including CITE-seq[10], Perturb-seq[11], scCAT-seq[12], SNARE-seq[13], SHARE-seq[14], and 10x Multiome.

For ADT data, the background signal appears to be a major component of noise since ADT data usually contains non-sparse positive counts, posing a challenging problem[15]. Recent research could effectively remove background noise by accurately estimating the background signal via the level of "ambient" ADT counts in empty droplets. However, methods without using strike-in control cells still mainly use a two-component mixture model to separately estimate the foreground and background signals[16-18].

Inspired by the reviewer's comment, we developed a variant of scButterfly-B namely scButterfly-B-ADT-BG, based on the two-component Gaussian mixture model similar to totalVI[18]. To be specific, we substituted the translator T in the scButterfly-B model with another translator T^{BG}, which considered background signals (Fig. R4). T^{BG} in latent space facilitated the translation between different modalities and the end-to-end mapping within each of the individual modalities. Illustrated with the ADT-to-ADT mapping and the ADT-to-RNA translation as an example, we first mapped the input $En_a(\mathbf{X}_a)$ from the ADT encoder into a 128-dimensional mean vector $\mathbf{X}^a_{mean}$ and log-variance vector $\mathbf{X}^a_{var}$ with two blocks of fully connected layer and LeakyReLU, respectively. Here, $\mathbf{X}_a$ represented the ADT input of the scButterfly model and En_a was the ADT encoder. Then, the translator T^{BG} obtained the latent embedding by sampling from the multivariate Gaussian distribution as follows:

$$\mathbf{X}_{embed} = \mathbf{X}^a_{mean} + \mathrm{e}^{\frac{\mathbf{X}^a_{var}}{2}} \times \mathbf{eps} \qquad \mathbf{eps} \sim \mathcal{N}(\mathbf{0}, \mathbf{I}).$$

For the ADT to RNA translation, we ultimately generated a translated RNA embedding $T^{BG}_{a\to r}(En_a(\mathbf{X}_a))$ with one block of a fully connected layer and LeakyReLU. For the ADT to ADT mapping, we generated a mapped ADT embedding $T^{BG}_{a\to a}(En_a(\mathbf{X}_a))$ with one background correction block from $\mathbf{X}_{embed}$. In the background correction block, we first got two matrices $\boldsymbol{\pi}$ and $\boldsymbol{\alpha}$ from $\mathbf{X}_{embed}$ with two blocks of the fully connected layer with activations of Sigmoid and $\mathrm{Exp} + 1$, respectively. $\boldsymbol{\pi}$ could be interpreted as the probability that any cell-feature pair activated due to the background alone, while $\boldsymbol{\alpha}$ denoted the scale factor for the foreground compared to the background for any cell-feature pair. We assumed that the background embeddings $\boldsymbol{\beta}$ follow a multivariate Gaussian distribution with a mean of $\boldsymbol{\beta}_{mean}$ and log-variance $\boldsymbol{\beta}_{var}$, where $\boldsymbol{\beta}_{mean}$ and $\boldsymbol{\beta}_{var}$ are two learnable vectors. Then we could model the corrected latent embedding $\mathbf{X}^{corrected}_{embed}$ as a two-component Gaussian mixture model as follows:

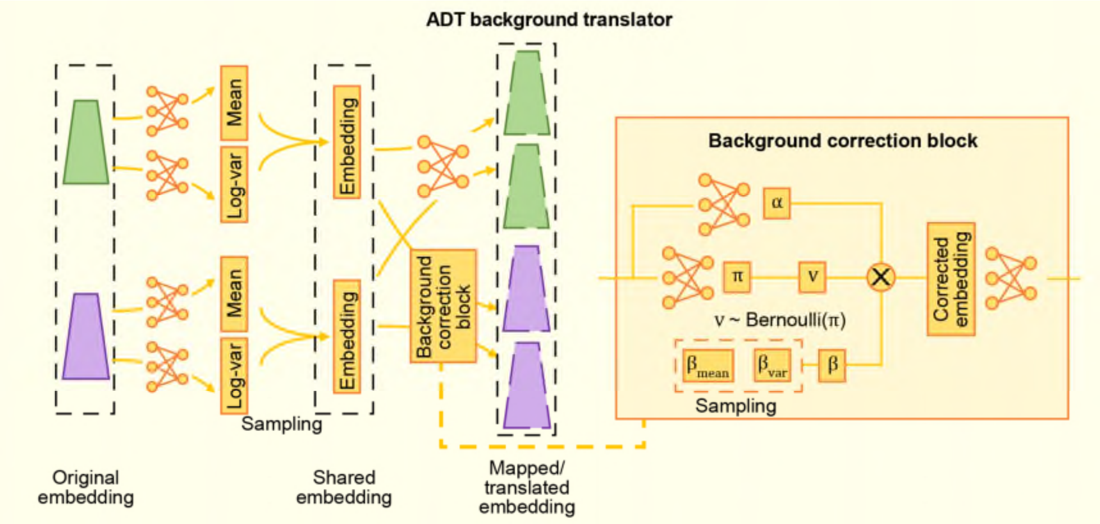

Fig. R4 | Overview of scButterfly translator considered background signals in ADT data. ADT background translator contains a background correction block, modelling the corrected embedding as a two-components mixture Gaussian distribution.

$$\begin{cases} \mathbf{v} \sim Bernoulli(\boldsymbol{\pi}) \\ \boldsymbol{\beta} \sim \mathcal{N}(\boldsymbol{\beta}_{mean}, \boldsymbol{\beta}_{var}) \\ \mathbf{X}_{embed}^{corrected} = \mathbf{v}\boldsymbol{\beta} + (1-\mathbf{v})\boldsymbol{\alpha}\boldsymbol{\beta} \end{cases}.$$

At last, we generated a mapped ADT embedding $T_{a\to a}^{BG}\left(En_a(\mathbf{X}_a)\right)$ with one fully connected layer and LeakyReLU from $\mathbf{X}_{embed}^{corrected}$, and used it as the input of the ADT decoder. Analogously, we used another two blocks of fully connected layer and LeakyReLU to map the input $En_r(\mathbf{X}_r)$ from the RNA encoder into a 128-dimensional mean vector $\mathbf{X}_{mean}^{r}$ and log-variance vector $\mathbf{X}_{var}^{r}$, respectively. For the RNA to RNA mapping, we generated the $T_{r\to r}^{BG}\left(En_r(\mathbf{X}_r)\right)$ using the same fully connected block of ADT to RNA translation. For the RNA to ADT, we used the same background correction block as ADT to ADT mapping to obtain $T_{r\to a}^{BG}\left(En_r(\mathbf{X}_r)\right)$. When performing translation for testing data, we used the formula $\mathbf{X}_{embed}^{corrected} = (1-\mathbf{v})\boldsymbol{\alpha}\boldsymbol{\beta}$ to generate the corrected embedding $\mathbf{X}_{embed}^{corrected}$, to remove the influence of background.

To evaluate the performance of scButterfly-B-ADT-BG, we performed cross-validation by batch on the CITE_BM and CITE_BMMC datasets. We also considered the influence of background signals of ADT data on the scButterfly-C and scButterfly-T variants, which applied the data augmentation strategies based on the cluster labels and cell types respectively, resulting in scButterfly-C-ADT-BG and scButterfly-T-ADT-BG. We illustrated the final benchmarking results in Fig. R5. Taking the CITE_BM dataset as an example (Fig. R5a), scButterfly-B-ADT-BG performed

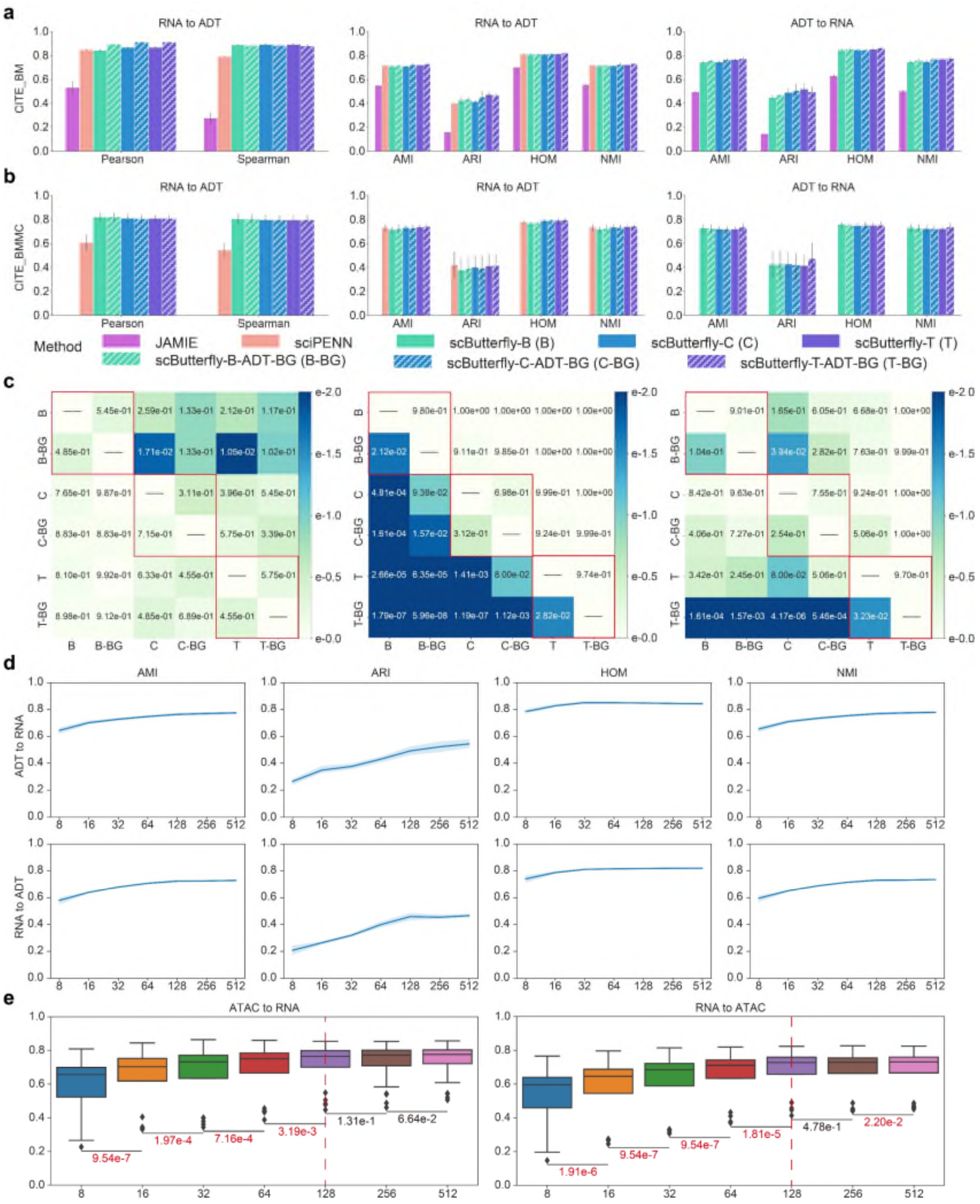

Fig. R5 | Performance of scButterfly when considering the background signals in ADT data and changing the embedding dimensions. a, **b**, Quantitative evaluation of the translation performance between transcriptome and proteome profiles in five-fold cross-validation by cell on the CITE_BMMC dataset (**a**) and two-fold cross-validation by batch on the CITE_BM dataset (**b**), via cell clustering with metrics of AMI, ARI, HOM, NMI and correlation with metrics of Pearson and Spearman correlation coefficients. The height of each bar denotes the median value of each metric and the error bars show 95% confidence interval. The scButterfly models with consideration about background are marked with white diagonal lines. Note that JAMIE encountered GPU memory errors on the CITE_BMMC dataset. **c**, Heatmap of p-values of one-sided paired Wilcoxon signed-rank tests. Each value in the heatmap indicates the significance of the advantage of a method (row) over another method (column). Red boxes emphasize the directly comparison between the

variant considering background signals with its corresponding scButterfly model. **d**, Clustering performance on the CITE_BM dataset with different embedding dimensions evaluated by AMI, ARI, HOM and NMI. Each point on the lines denotes the median performance in five-fold cross-validation with different embedding dimensions, with error bands implying the 95% confidence interval. **e**, Overall clustering performance on the CITE_BM dataset with different embedding dimensions. In the boxplots, the center lines, box limits and whiskers denote the median, upper and lower quartiles, and 1.5× interquartile range, respectively. The *p*-values of one-sided paired Wilcoxon signed-rank tests are shown under the boxes with the values under 0.05 marked with red, indicating the significance of the advantage of model with high embedding dimensions over that with low dimensions.

slightly better than scButterfly-B, mainly on the adjusted Rand index (ARI) and Pearson correlation coefficient for RNA to ADT translation. We also observed similar improvements of scButterfly-C-ADT-BG and scButterfly-T-ADT-BG, compared to scButterfly-C and scButterfly-T, respectively. However, the strategy of considering background signals of ADT data will not lead to an obvious improvement of scButterfly on every dataset. For example, scButterfly-B-ADT-BG had no significant advantages compared to scButterfly-B on the CITE_BMMC dataset (Fig. R5b). The same situation was also observed in the comparisons between scButterfly-C-ADT-BG and scButterfly-C, as well as scButterfly-T-ADT-BG and scButterfly-T. One-sided paired Wilcoxon signed-rank tests showed that distinguishing the background and foreground signal could only provide a limited advantage on the clustering metrics for the translation from RNA to ADT on these two datasets (Fig. R5c). The translation performance of scButterfly considering background signals did not have a significant advantage over that without considering background under the significance level 0.1, which indicated that the consideration of background signals is an optional but not necessary choice for cross-modality translation.

For the second point, we discussed the impact of different embedding dimensions on translation performance in CITE-seq data analyses. Firstly, indeed as the reviewer pointed out, ADT data often have very low dimensions, such as the CITE_BM dataset, which only contains 25 antibodies. However, scButterfly needs to map the RNA data and ADT data into the embedding spaces with the same dimensions to realize cross-modality translations. Since RNA data in CITE-seq usually contains dimensions much higher than ADT data (for example, the CITE_BM dataset contains 17,009 genes but only 25 antibodies), simultaneously projecting both RNA and ADT data to the same dimensions lower than the original ADT data may lead to a massive loss of information for RNA data. Therefore, we chose a trade-off dimension 128 between the high

dimension of RNA and the low dimension of ADT data. Secondly, as suggested in recent research[19], the principle of choosing a proper embedding dimension should be small enough to be efficient and large enough to be effective. Therefore, we further investigated the translation performance using different embedding dimensions. As an example for illustration, we performed five-fold cross-validation on the CITE_BM dataset using scButterfly-B with embedding dimensions of 8, 16, 32, 64, 128, 256, and 512. As shown in Fig. R5d, the translation performance improved as the embedding dimensions increased and reached a relatively stable level at 128 dimensions, especially on the scores of the adjusted mutual information (AMI), homogeneity (HOM), and normalized mutual information (NMI). We also conducted one-sided paired Wilcoxon signed-rank tests between the adjacent choices of embedding dimensions (Fig. R5e), including 8 vs 16, 16 vs 32, 32 vs 64, 64 vs 128, 128 vs 256, and 256 vs 512. The *p*-values indicate that, at the significance level of 0.05, the translation performance significantly increases with the increase of dimensions below 128. However, embedding dimensions greater than 128 no longer provide significant improvements, which implies that using 128 as the embedding dimension provides a balance between efficiency and effectiveness.

In conclusion, we noticed that considering the background signals could only provide limited improvements, thus, distinguishing the foreground and background signals in the ADT data is not necessary. For the choice of embedding dimensions, we demonstrated that 128 is a robust embedding dimension. We have added the discussion about distinguishing the foreground and background signals and the impact of different embedding dimensions into the supplementary file (Supplementary Texts 10 and 12). Additionally, we provided scButterfly-B-ADT-BG, scButterfly-C-ADT-BG, scButterfly-T-ADT-BG as optional choices for users and once again deeply valued the insightful commentary provided by the reviewer about the modelling of ADT data.

COMMENT 4

How did the authors set the hyperparameters, such as setting $w_r = 1$, $w_a = 2$, $w_{ELBO} = 20/I_r$ ($w_{ELBO} = 20/I_r + 20/I_a$ in total loss) and $w_{dis} = 1$. Is the model sensitive to these parameters? The authors should provide some sensitivity analyses.

RESPONSE TO COMMENT 4

We appreciate the reviewer for this insightful comment. We first clarify that we chose the setting of hyperparameters empirically based on multiple datasets. For the hyperparameters in loss calculation, we used four parameters, including w_r, w_a, the numerator of w_{ELBO} denoted as w_e ($w_{ELBO} = \frac{w_e}{Input\ dimensions}$), and w_{dis}. w_r and w_a are the weights of reconstruction/translation loss for ATAC and RNA respectively. w_{ELBO} is the weight of ELBO loss and w_{dis} is the weight of discriminator loss. Taking the weights of loss calculation as an example, we set the w_{ELBO} with the value adaptive to the dimensions of the input data to avoid the posterior collapse following [20]. As demonstrated in the manuscript, scButterfly with these hyperparameters consistently outperformed baseline methods in various experiments on various datasets, indicating the effectiveness and robustness of scButterfly with these hyperparameters settings in practice.

Following the reviewer's suggestion, we further conducted sensitivity analysis of four hyperparameters in loss calculation. According to the default settings $(w_r, w_a, w_e, w_{dis}) = (1, 2, 20, 1)$, we selected the range of w_r from 0.5 to 1.5 with a step of 0.1, the range of w_a from 1.5 to 2.5 with a step of 0.1, the range of w_e from 15 to 25 with a step of 1, and the range of w_{dis} from 0.5 to 1.5 with a step of 0.1. By changing one parameter at a time, we generated a total of 40 different hyperparameter settings. Take the five-fold cross-validation on the MCC dataset as an example, we evaluated the translation performance of the scButterfly-B with these hyperparameter settings. We denoted the settings of hyperparameters with the format (w_r, w_a, w_e, w_{dis}), marked the default settings with yellow. As shown in Fig. R6, with the variation of different hyperparameters settings, the clustering metrics remained stable, demonstrating that scButterfly is relatively robust to these hyperparameters and achieved consistent performance. Furthermore, scButterfly also showed robustness to the hyperparameters for training epochs and early-stop patience, with more detailed results provided in Response to Comment #13 of Reviewer #2.

In summary, the results of sensitivity analysis highlighted that scButterfly has robustness to different hyperparameter settings, such as the weights of loss calculation (w_r, w_a, w_e, w_{dis}), within a certain range. The discussions about the sensitive analysis for the hyperparameters have been added to the Section "scButterfly enables cross-modality translation while preserving cell heterogeneity" in the revised manuscript.

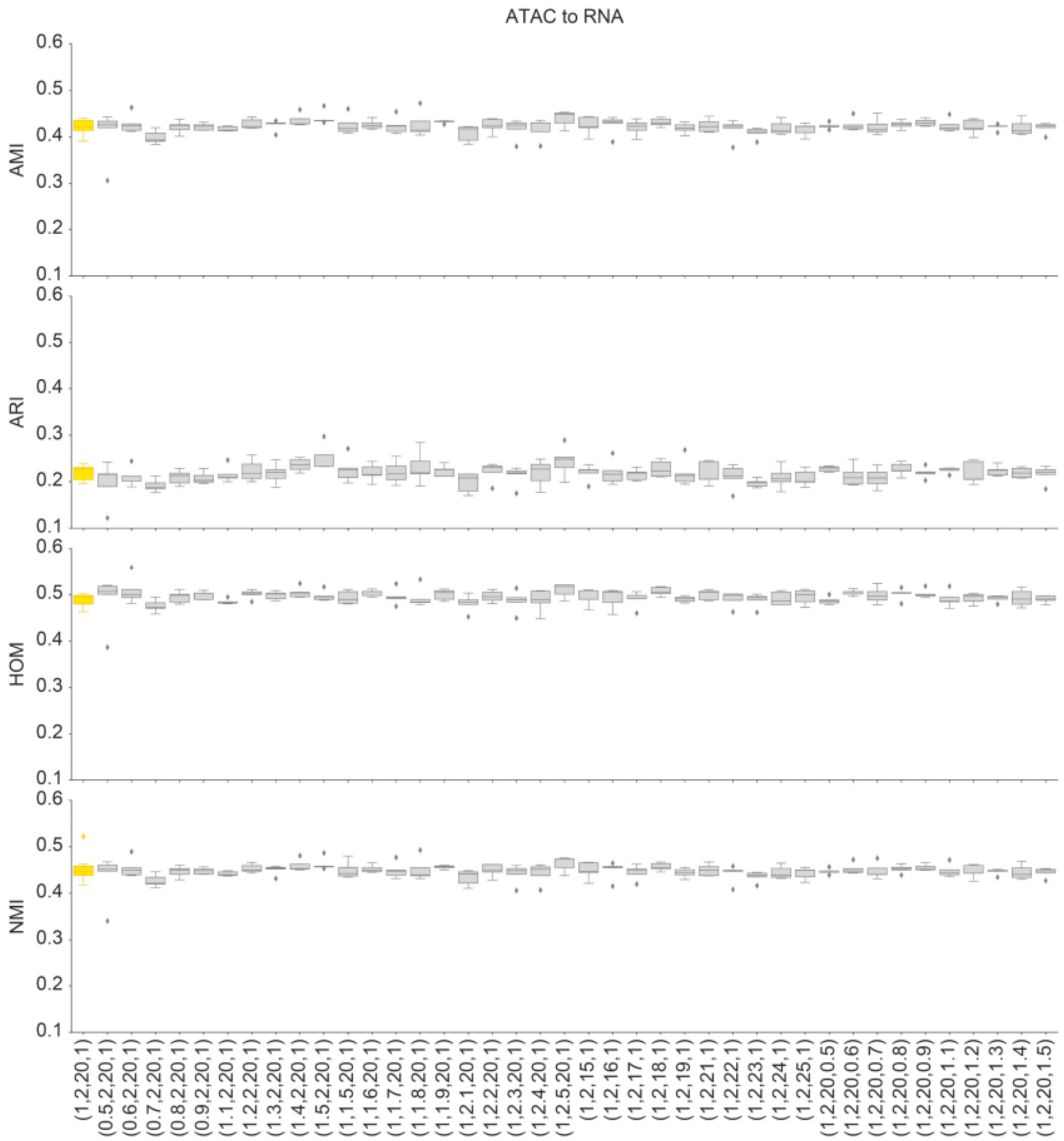

Figure R6 | Performance of scButterfly-B with different hyperparameters on the MCC dataset. The settings of hyperparameter are denoted as (w_r, w_a, w_e, w_{dis}) and we evaluated the performance of scButterfly-B under 41 different hyperparameter settings. The boxplots marked with yellow denote the default hyperparameter setting of scButterfly-B. In the boxplots, the center lines, box limits and whiskers denote the median, upper and lower quartiles, and 1.5× interquartile range, respectively.

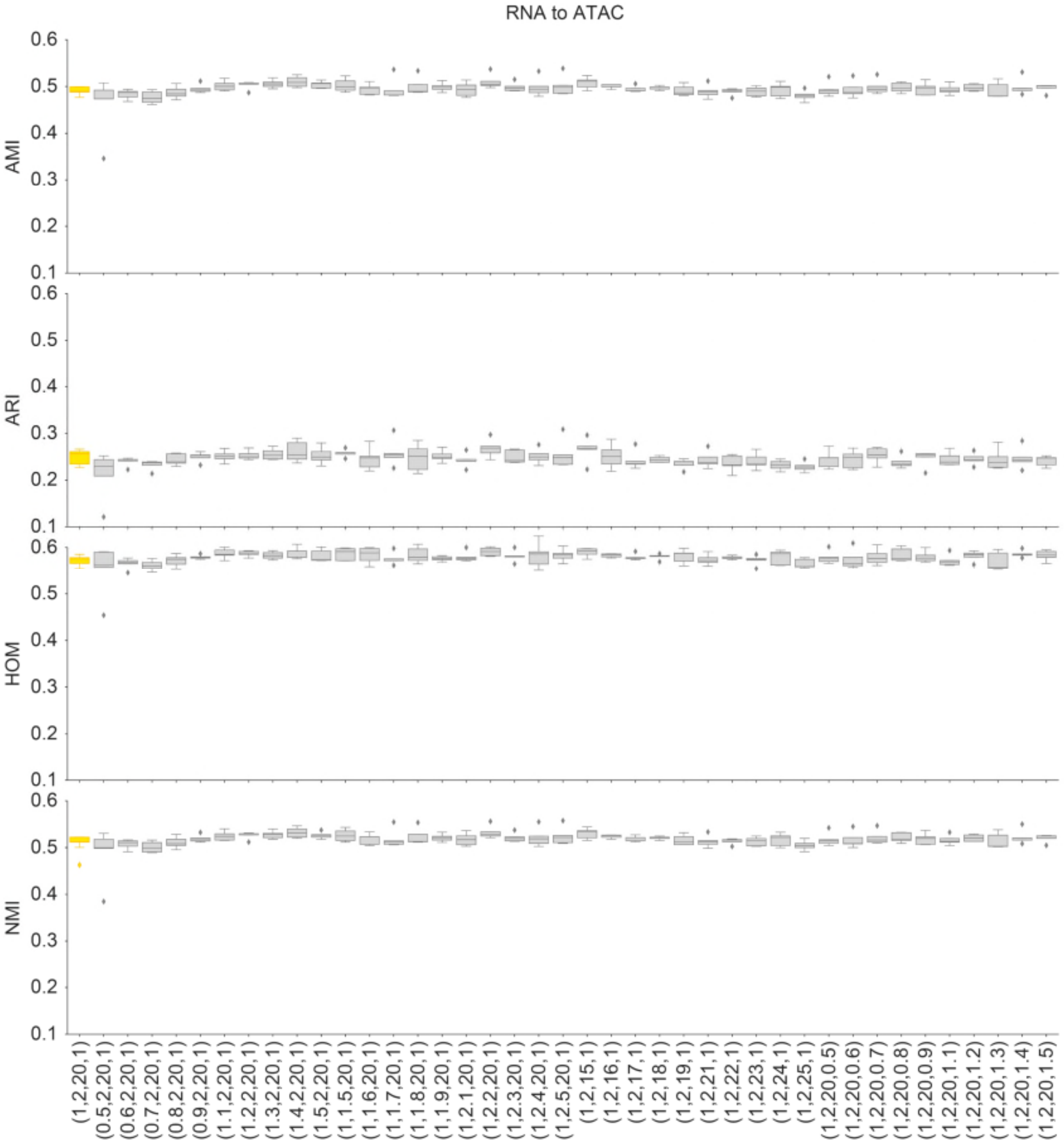

Figure R6 (continue) | Performance of scButterfly-B with different hyperparameters on the MCC dataset. The settings of hyperparameter are denoted as (w_r, w_a, w_e, w_{dis}) and we evaluated the performance of scButterfly-B under 41 different hyperparameter settings. The boxplots marked with yellow denote the default hyperparameter setting of scButterfly-B. In the boxplots, the center lines, box limits and whiskers denote the median, upper and lower quartiles, and 1.5× interquartile range, respectively.

COMMENT 5

The authors show many clustering results on the RNA to ATAC data and ATAC to RNA data. In most cases, the RNA to ATAC data has a better performance than the ATAC to RNA data. The authors should explain this. Besides, are there any biological insights in doing the cell

clustering from both RNA to ATAC and ATAC to RNA? I think we just need to find the best way to do this task.

RESPONSE TO COMMENT 5

We appreciate the reviewer for this insightful comment. As a response to this comment, we first explained that the performance difference between the RNA to ATAC translation and the ATAC to RNA translation primarily arises from the variations between the qualities of RNA and ATAC data. Moreover, we believe that clustering is an appropriate approach to evaluate the performance of translation, and clustering based on RNA to ATAC and ATAC to RNA translated profiles will provide biological insights from different perspectives. The detailed explanations in terms of the two main points are illustrated in the subsequent paragraphs.

For the first point, in agreement with the reviewer's comment, it is evident that RNA to ATAC translation generally exhibits superior clustering performance compared to ATAC to RNA translation. We attributed this difference in clustering performance primarily to the quality of the input data. We addressed this inference with the following two points. Firstly, RNA data often possess higher quality compared to ATAC data. Recent studies have demonstrated that ATAC data typically manifest higher dimensionality and sparsity, leading to increased noise compared to RNA[21-24]. As depicted in Columns #1 and #3 in Fig. R7, the original RNA data consistently achieves better clustering performance than the original ATAC data. Secondly, we clarify that the qualities of RNA to ATAC and ATAC to RNA translations are strongly influenced by the qualities of original RNA and ATAC data, respectively. The results in Fig. R7 illustrate a strong association between the clustering metrics of the RNA to ATAC translation and original RNA data. We also observed this association between the clustering metrics of the ATAC to RNA translation and original ATAC data. Particularly in the MB and MK datasets, the quality difference between original RNA data and noisy original ATAC data led to a significant disparity in translation performance (Rows #2 and #5 in Fig. R7). Furthermore, we conducted five-fold cross-validation on seven datasets including BMMC, MB, CL, MCC, MK, MDS, and PBMC. We evaluated the clustering metrics of the AMI, ARI, HOM, and NMI of the original RNA data, the original ATAC data, RNA to ATAC translation, and ATAC to RNA translation from scButterfly-B, respectively. For each clustering metric, we calculated the Pearson correlation coefficients between the metric values of the original data and that of translated profiles among seven datasets. Table R1 illustrated that the clustering

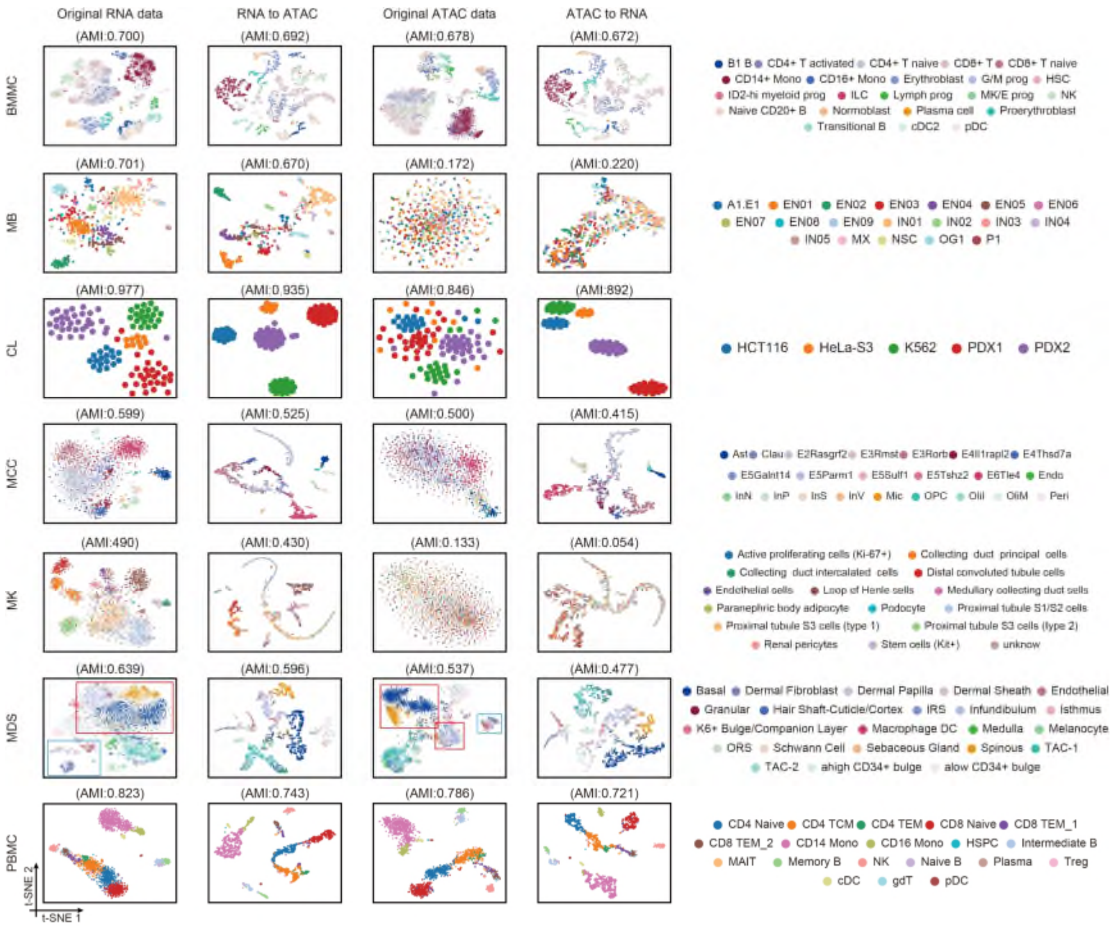

Figure R7 | Visualization results and clustering performance of original profiles and translated profiles on multiple datasets. t-SNE visualizations of cells in the first test fold of five-fold cross-validation on the seven datasets (BMMC, MB, CL, MCC, MK, MDS, and PBMC), using the original profiles and the profiles translated by scButterfly-B, with AMI clustering performance marked upon the visualization.

performance of RNA to ATAC translation had a stronger correlation with the clustering metrics of original RNA data, compared to the metrics of the original ATAC data. For the ATAC to RNA translation, we also observed a similar stronger correlation with the metrics of original ATAC data, compared to the metrics of original RNA data. These results indicated that the cell clustering metrics of the translated profiles from scButterfly may have a strong association with the clustering metrics of input data. Taking the above two points into consideration, we inferred that the difference in clustering performance between the two methods primarily stems from the variance in input data quality.

For the second point, we discussed the biological insights for the utilization of clustering metrics and for evaluating both RNA to ATAC and ATAC to RNA

Table R1 | Pearson correlation coefficients between the clustering performance of original profiles and translated profiles. The Pearson correlation coefficients between the clustering performance of original profiles and translated profiles was calculated based on five-fold cross-validation on seven datasets (BMMC, MB, CL, MCC, MK, MDS, and PBMC) based on different clustering metrics. The highest value for each row is marked with bold.

Clustering metrics		Original RNA	Original ATAC
AMI	RNA to ATAC	**0.973**	0.754
	ATAC to RNA	0.844	**0.985**
ARI	RNA to ATAC	**0.880**	0.540
	ATAC to RNA	0.849	**0.882**
HOM	RNA to ATAC	**0.976**	0.725
	ATAC to RNA	0.851	**0.981**
NMI	RNA to ATAC	**0.970**	0.860
	ATAC to RNA	0.786	**0.989**

translations, respectively. For the biological insights of mainly focusing on clustering metrics for evaluation, we demonstrate that clustering metrics offer three advantages. Firstly, the clustering metrics have been widely used in evaluating various tasks, such as batch alignment and cell type annotation[5, 24-28], indicating their ability to reflect the model's performance in multiple tasks. Secondly, evaluating the clustering metrics often provides insights into the ability of prediction to preserve cell heterogeneity. As shown in Fig. R7, translated profiles with higher clustering metrics tend to exhibit more distinct visualizations, implying that the translated profiles are more adept at preserving cell heterogeneity. Thirdly, in addition to clustering metrics, correlation coefficients, such as Pearson and Spearman correlation coefficients, are widely used indicators for assessing translation effectiveness. Compared to correlation coefficients, clustering metrics are less susceptible to noise and sparsity[29] present in the single-cell data[30]. Moreover, it has been shown that correlation coefficients are non-robust to outliers[31] and inaccurate when applied to high-dimensional data[32]. In brief, evaluating with the clustering metrics could accurately measure the translation performance of the model and is suitable for reflecting the ability of the model for multiple downstream analyses.

In response to simultaneously evaluating RNA to ATAC and ATAC to RNA translations, we emphasize that, although ATAC to RNA translation often performs worse than RNA to ATAC translation, both of them provide valuable biological insights. Firstly, RNA and ATAC can capture distinct types of cellular information and provide multi-perspective insights into cell states. While RNA data often has higher quality and lower noise compared to ATAC data, offering comprehensive gene expression profiles, it fails to capture the chromatin regulatory landscape that governs transcription in each cell type[33]. Secondly, for single-cell multi-omics profiles, the information from RNA and ATAC data should be complementary to each other rather than substitute. For example, in the MDS dataset, using the original ATAC data, we can distinguish Basal, Spinous, and Infundibulum cells in t-SNE visualization (marked with red boxes) that cannot be distinguished using the original RNA data (Row #6 in Fig. R7). Conversely, using the original RNA data, we could differentiate Dermal Fibroblast, Dermal Papilla, Dermal Sheath, Endothelial, and Melanocyte cells, whereas the original ATAC data mixed them together (marked with blue boxes). This result indicated that RNA and ATAC data usually possess unique information respectively and could be complementary. Thus, we should fully exploit this information for multi-modal data analysis, rather than discarding one of them. Thirdly, both RNA to ATAC and ATAC to RNA translations have practical applications. With the use of the scButterfly model trained on multi-modal data, massive single-modality RNA or ATAC data accumulated in repositories[34-36] could be translated into different modalities and be utilized for modality-specific downstream analysis. For example, through ATAC to RNA translation, we can conduct differential expression analysis on single-modality ATAC data, identifying the differentially expressed genes (DEGs) and performing gene ontology enrichment analysis[37, 38] (Section "scButterfly effectively translates data of novel contexts and reveals biological insights"). Besides, by employing advanced RNA annotation methods to translated profiles, we could accurately annotate the unlabeled ATAC data, which is challenging for directly performing annotation on the ATAC data (Section "scButterfly facilitates integrative analysis, data enhancement, and cell type annotation"). Through RNA to ATAC translation, we can perform ATAC-specific analyses such as single-nucleotide polymorphisms analysis[39] and TF regulatory networks inferring[40], for single-modality RNA data (Response to Comment #6 of Reviewer #2), helping understand the mechanisms of interaction between transcription factors. Additionally, as demonstrated in the integrative analysis section (Section "scButterfly facilitates integrative analysis,

data enhancement, and cell type annotation"), both RNA to ATAC and ATAC to RNA translations enable us to complement single-modality RNA or ATAC data for further integrative analysis, yielding more comprehensive results than single-modal analyses. Fourthly, RNA to ATAC and ATAC to RNA translations can also be used together when dealing with low-quality paired data. Experimental results of data enhancement exhibited that, the quality of the data improved after model translation, and the translated profiles could be better utilized in downstream analyses, such as integrative analyses (Section "scButterfly facilitates integrative analysis, data enhancement, and cell type annotation"). In summary, we identified that both RNA to ATAC and ATAC to RNA can complementarily capture diverse information in the original data, and can be utilized separately or jointly for multiple downstream analyses and applications, providing versatile biological insights.

Overall, we explain the differences in the performance of RNA to ATAC and ATAC to RNA primarily stem from the quality differences in RNA and ATAC data. Moreover, we further provide more detailed motivation and richer biological insights by using the clustering metrics for evaluating both RNA to ATAC and ATAC to RNA. Finally, we would like to again express our gratitude to the reviewer for your constructive suggestion, helping us further contemplate these issues. We have added these discussions in the Supplementary Texts 2 and 3.

COMMENT 6

I believe the proposed model is not only for cell type annotation. Can we also do any ATAC-specific analyses, such as TF-target gene regulatory network inference, from the output of the model? That would be more meaningful and provide more biological insights.

RESPONSE TO COMMENT 6

We appreciate the reviewer for this enlightening comment and further added more ATAC-specific analyses for the translated profiles. Firstly, we can identify cell type-specific peaks and provide cell type-specific enrichment based on the scButterfly translated ATAC profiles. Specifically, we used scButterfly-C to obtain the RNA to ATAC translated profiles for the cells in the first test fold of five-fold cross-validation on the PBMC dataset. We utilized EpiScanpy[41] on the ATAC translated profiles to identify the top 1000 differentially accessible peaks (DAPs) of each cell type and calculated 1000 peaks with the lowest accessibility among all cell types as the

background peaks. Subsequently, we performed single-nucleotide polymorphisms (SNPs) enrichment analysis using SNPsea[39] for the cell type-specific DAPs and background peaks to obtain tissues explicitly affected by these peaks, respectively. We illustrated the top 30 significantly enriched tissues for each cell type in Fig. R8, where the horizontal dashed and solid lines denote *p*-value cutoffs at the significant level of 0.05 (unadjusted and with Bonferroni correction, respectively), and the heatmaps show Pearson correlation coefficients for pairs of expression profiles ordered by hierarchical clustering with unweighted pair-group method with arithmetic means (UPGMA). The identified cell type-specific DAPs from the scButterfly translated ATAC profiles showed significant affection in their corresponding types (Fig. R8). For example, the DAPs for all three subgroups of CD4+T cells in the original data (CD4_Naive, CD4_TCM, and CD4_TEM) demonstrated a significant relation with PB-CD4+T cells.

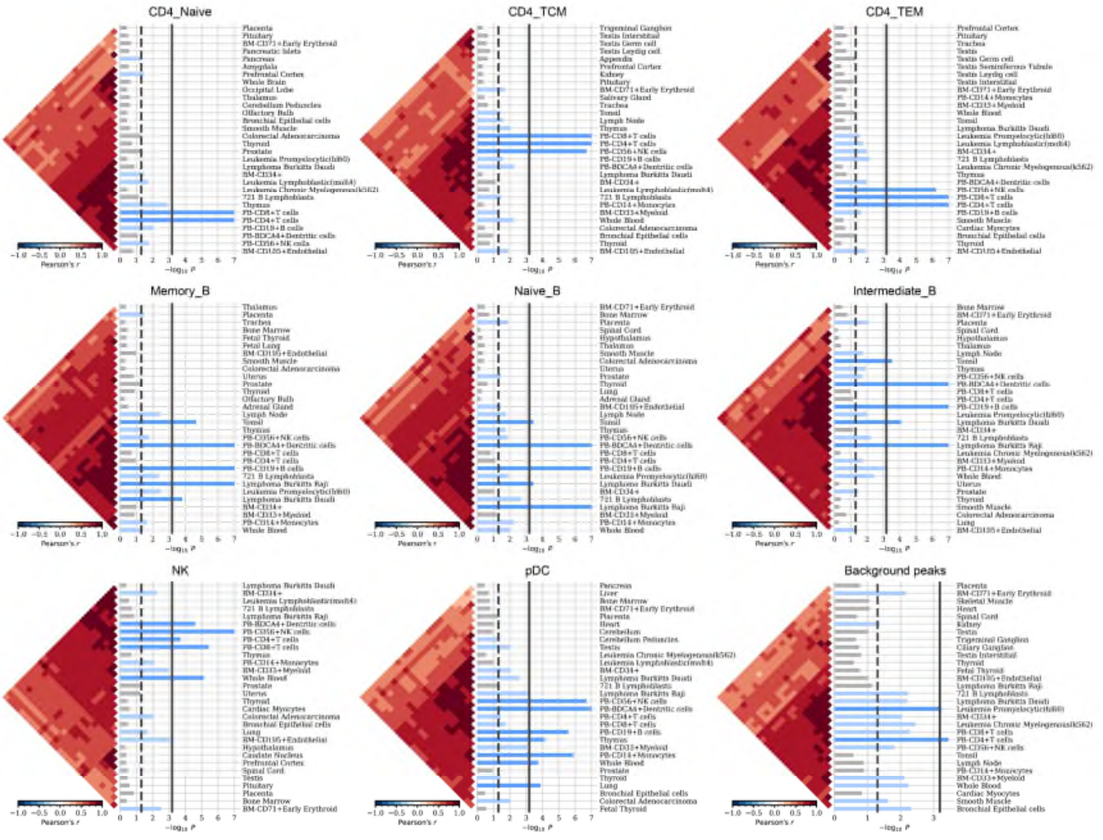

Figure R8 | SNPsea analysis for the identified cell type-specific peaks for the scButterfly-C translated profiles. The top 30 significantly enriched tissues in SNPsea analysis on cell type-specific peaks identified by EpiScanpy and the background peaks. The vertical dashed and solid lines denote the one-sided *p*-value cutoff at the significant level of 0.05 (unadjusted and with Bonferroni correction, respectively), testing the significance of whether all implicated genes, in aggregate, are enriched for specificity to an annotation. The heatmaps show Pearson correlation coefficients for pairs of expression profiles ordered by hierarchical clustering with UPGMA.

Besides, the DAPs of three different subtypes of B cells in the original data (Memory_B, Naive_B, and Intermediate_B) also exhibited a high correlation with PB-CD19+B cells. Moreover, the DAPs for nature killer (NK) cells and plasmacytoid dendritic (pDC) cells were also related to PB-CD56+NK cells and PB-BDCA+Dentritic cells respectively. However, compared to the cell type-specific DAPs, the background peaks exhibited less significant enrichment, indicating that scButterfly-C translated ATAC profiles could be effectively used for identifying the cell type-specific peaks and providing cellular heterogeneity insights in related tissues.

To further demonstrate the ability of scButterfly to reveal cell type-specific transcription factors (TFs), we performed TF regulatory networks inference for the scButterfly translated profiles with DeepTFni[40]. Specifically, for each cell type, we first obtained the TF-TF adjacency matrix for the translated profiles from scButterfly-C with DeepTFni. Then, to identify the cell type-specific TFs, we first enriched the top 30 TFs with the highest degree in the TF-TF adjacency matrix as the candidates. From these candidates, we identified the differentially expressed TFs that were only enriched in the specific type but not enriched in other types, indicating their cell type specificity. We observed that retinoic acid receptor *RARγ* was enriched as the cell type-specific TFs for CD8_Naive, which has been suggested as a potential regulatory role in Naive CD8+ T cells[42]. Additionally, we found *FOXP3* was enriched in CD4_Naive, which is consistent with its known association with this cell type[43]. Furthermore, we observed the enrichment of *MEF2D* in CD14_Mono cells, which has been shown to interact synergistically with *SP1* to activate *CD14* promoter[44]. Besides, we also observed this synergistic relationship between *MEF2D* and *SP1* in the TF-TF adjacency matrix of CD14_Mono. Although *SP1* was not identified as differentially expressed TF in CD14_Mono cells, it is worth noting that *SP1* was still highly enriched in CD14_Mono cells (ranked 4/30 for the candidates with the highest degree in TF-TF adjacency matrix of CD14_Mono). These results suggest that the translated profiles from scButterfly can be used for TF-TF network inference and provide biological insights.

In conclusion, we demonstrated that scButterfly RNA to ATAC translation can be applied to various ATAC-specific analyses. We sincerely appreciate the valuable comment provided by the reviewer, which helped us improve our manuscript. We have added the aforementioned ATAC-specific analyses to the Section "scButterfly effectively translates data of novel contexts and reveals biological insights".

COMMENT 7

In the introduction, we authors provide some reasons/motivations for developing the model. However, it seems that some motivations are very weak, and some problems have been addressed by one or more previous methods. Specifically:

RESPONSE TO COMMENT 7

We deeply appreciate the reviewer for this constructive comment. We have provided detailed descriptions of the motivations for developing the model in Response to Comments #8, #9, and #10 of Reviewer #2 and reorganized the motivations in our revised manuscript.

COMMENT 8

“Second, an effective data augmentation scheme is in demand to better tackle the limited number of cells measured by multi-omics protocols and uncover cell heterogeneity in highly noisy data of complex tissues.”. This is an optional, not necessary, function in the model. So, this reason/motivation is very weak. It would be better if authors could show the necessity of doing this approach and provide some evidence.

RESPONSE TO COMMENT 8

We express our gratitude for the reviewer's valuable insights, which helped us refine the description of our motivation. Indeed, as the reviewer pointed out, we found that data augmentation was not necessary for all methods, as some methods did not show improvement when using additional synthetic samples. However, we believe that data augmentation remains an effective approach for scButterfly since data augmentation could bring significant improvements to the translation performance across multiple datasets, although scButterfly has already outperformed other baseline methods. Moreover, we observed that data augmentation performs particularly effectively in datasets with limited numbers of cells. In the following paragraphs, we will provide detailed discussions.

Firstly, we apologize for the weak description of our motivation in the original manuscript. As the reviewer mentioned, the data augmentation strategy should be considered as an optional instead of a necessary function, because we found that it does not consistently benefit all methods. Specifically, we applied the data augmentation

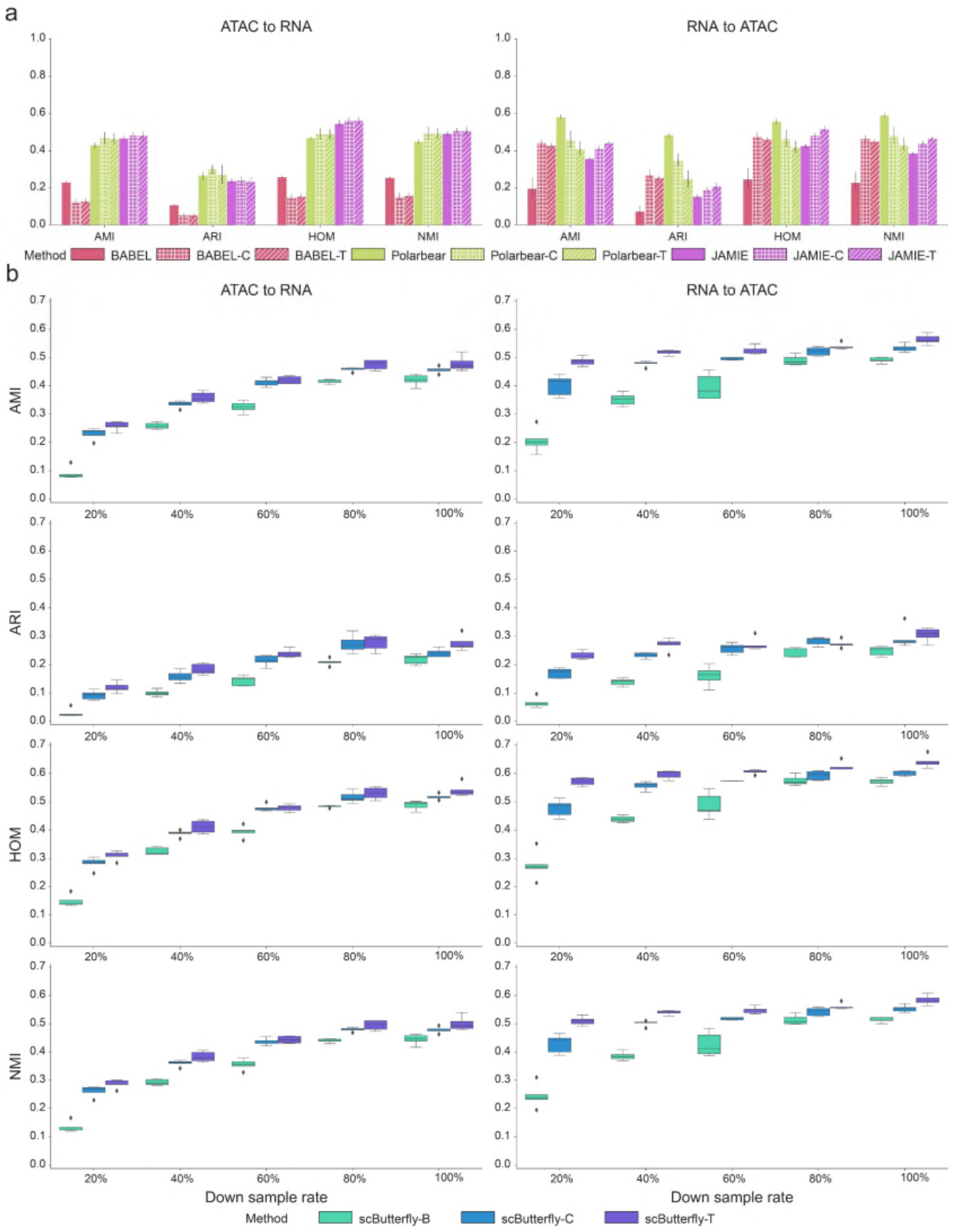

Figure R9 | Performance of data augmentation strategy on different methods and the scenario with limited number of cells. a, Quantitative evaluation of the translation performance of different methods with data augmentation strategy, with five-fold cross-validation on the MCC dataset, via cell clustering with metrics of AMI, ARI, HOM and NMI. The height of each bar denotes the median value of each metric and the error bars show 95% confidence interval. **b**, Evaluation of the cross-modality translation performance for data with different rate of random down sampled, including five-fold cross-validation on the MCC dataset, via cell clustering with metrics of AMI, ARI, HOM and NMI. In the boxplots, the center lines, box limits, whiskers and notches denote the median, upper and lower quartiles, 1.5× interquartile range and 95% confidence interval calculated using a Gaussian-based asymptotic approximation, respectively.

methods used in scButterfly-C and scButterfly-T variants to other baseline methods, including BABEL[45], Polarbear[46], and JAMIE[47], and conducted the five-fold cross-validation on the MCC dataset. The experimental results demonstrate that, for JAMIE, the data augmentation scheme indeed led to simultaneous improvements in both RNA to ATAC and ATAC to RNA translations (Fig. R9a). Nevertheless, data augmentation adversely affected the model's ability to learn effectively in the case of Polarbear for the RNA to ATAC translation (Fig. R9a). When considering BABEL, it is evident that data augmentation resulted in a decline in ATAC to RNA translation performance, but conversely, there was a significant improvement in RNA to ATAC translation (Fig. R9a). Therefore, data augmentation strategies do not always consistently result in robust developments for all methods. Thus, we have revised the description of our motivation.

However, we must clarify that data augmentation remains an effective strategy for the scButterfly model. Firstly, data augmentation strategies based on cluster labels or cell-type labels improved the translation performance of scButterfly-B. As we have introduced in Section “scButterfly enables cross-modality translation while preserving cell heterogeneity”, we conducted five-fold cross-validation on seven datasets (BMMC, MB, CL, MCC, MK, MDS, and PBMC). As shown in Fig. 2b, scButterfly-B achieved better performance than the baseline methods in both directions of cross-modality translation and efficiently characterized cellular heterogeneity. Besides, we also noticed that scButterfly-C yielded significantly better performance than scButterfly-B, while scButterfly-T further promoted the performance (Fig. 2c). These results indicated that the data augmentation strategies are necessary for scButterfly.

Additionally, we found that data augmentation shows strong potential in tackling the issue that multi-omics data usually contains limited samples. To illustrate this point, we randomly selected 20%, 40%, 60%, 80%, and 100% of the cells in the MCC dataset and evaluated the translation performance of scButterfly-B, scButterfly-C, and scButterfly-T, by conducting five-fold cross-validation on this down-sampled dataset. As shown in Fig. R9b, the data augmentation strategies were particularly effective when the number of cells was extremely low, suggesting that data augmentation strategies could competently handle multi-omics data with limited cell numbers and uncover cell heterogeneity.

We would like to express our gratitude once again to the reviewer for the insightful comment. According to the suggestion from the reviewer, we have modified the

description of the motivation in the manuscript to make it more accurate and added the discussions about the data augmentation strategies under the scenarios with limited cells in Section "scButterfly enables cross-modality translation while preserving cell heterogeneity".

COMMENT 9

"Third, there is a prevalent challenging scenario where the single-modality data to be translated has inter-sample variations (e.g., batch effects) and even different biological contexts (e.g., novel cell types) compared to the data used for training". These problems, such as batch correction, have been addressed, to some extent, by many previous methods. So, this reason/motivation is also very weak. It is advisable to highlight potential issues with previous methods concerning batch correction.

RESPONSE TO COMMENT 9

We appreciate the meticulous comment from the reviewer and apologize for any ambiguity description of the motivation. We would like to clarify that the scenario here we referring to is when the samples to be translated contain entirely unseen batches or cell types, which are not available on the training datasets. Although some previous cross-modality translation methods have already considered the issues of translating on unseen batches and cell types, they still struggled to achieve satisfactory performance, implying this problem is still not well resolved. Besides, several limitations of existing batch correction methods, such as over-correcting and difficulty in maintaining the dimensions of original data, may lead to potential issues.

We fully agree with the reviewer's comments that issues such as batch correction have been addressed in many previous studies. The issues of unseen batches or unseen cell types in prediction tasks are essentially the result of differences in the distribution of training and testing samples, which has been widely investigated in machine learning as the problem of dataset shift[48-50]. The dataset shift in biological datasets has also been extensively studied, especially in the context of batch correction[5, 6, 51] and analysis with unseen cell types[52-54]. In the case of cross-modality translation problems, only a few methods including BABEL and Polarbear have taken into account the translation for cells from different batches and cell types, which better aligns with real-world scenarios. However, we found that these issues haven't yet been fully resolved and remain to be challenging. Specifically, we conducted four-fold cross-validation by batch on the three

multi-batch datasets (BMMC, CL, and MDS) and three-fold cross-validation by cell type on the other four datasets (MB, MCC, MK, and PBMC). As shown in Fig. 3ab, both BABEL and Polarbear struggled to maintain reasonable performance when dealing with unseen batches or unseen cell types. Contrarily, scButterfly model outperformed BABEL and Polarbear (Fig. 3ab and Supplementary Figs. 6-10), indicating the advantage of scButterfly in the cross-modality translation scenario with unseen batches or unseen cell types. This result demonstrated that existing methods have not adequately addressed this problem of translating on datasets with dataset shift.

In addition, it is an optional scheme to perform batch correction before training, enabling cross-modality translation on unseen batches. However, we believe that there are some other potential issues with the existing batch correction methods[5, 6]. As we discussed in Response to Comment #2 of Reviewer #2, these issues have been raised in many batch correction studies and could be summarized as follows: (1) There is a trade-off between batch effects correction and conservative biological variations and stronger removal of batch effects often leads to a higher degree elimination of bio-conservation. Keeping the balance between the preservation of biological heterogeneity and the correction of batch effect is still an open problem[5]. (2) Most state-of-the-art batch correction methods often perform dimension reduction, and cannot output corrected data with the original dimension. These issues result in the combination of batch effect correction methods and scButterfly being unable to achieve better performance. We utilized existing batch correction methods to perform batch correction on the training and testing datasets and trained scButterfly on the corrected data.

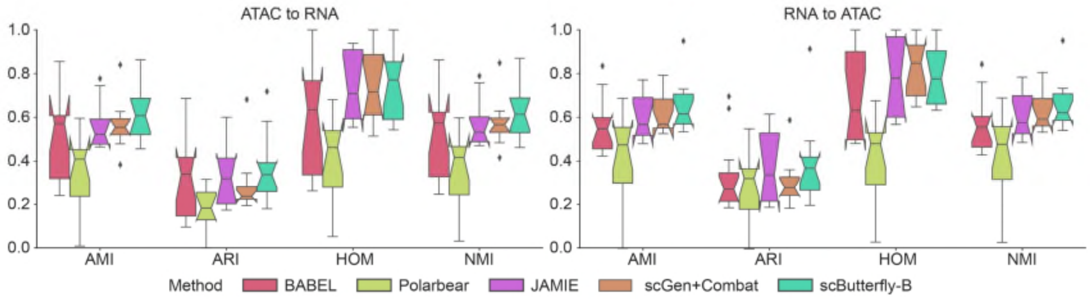

Figure R10 | Performance of cross-modality translation for the batch corrected dataset. Evaluation of the cross-modality translation performance for paired data, including four-fold cross-validation by batch experiments in three multi-batch datasets of BMMC, CL, and MDS, via cell clustering with metrics of AMI, ARI, HOM and NMI. Note that JAMIE encountered GPU memory errors on the BMMC dataset. In the boxplots, the center lines, box limits, whiskers and notches denote the median, upper and lower quartiles, 1.5× interquartile range and 95% confidence interval calculated using a Gaussian-based asymptotic approximation, respectively.

Specifically, we performed batch correction using scGen and pyComBat separately for RNA and ATAC data on the BMMC, CL, and MDS datasets and trained the basic scButterfly model for translation. As suggested in recent research[5], scGen[7] and pyComBat[8] could separately achieve the best overall performance among the batch effect correction methods which could output the corrected original dimension count matrix. We performed cross-validation by batch (Fig. R10) and it can be observed that the batch-effects-corrected data did not improve translation performance, suggesting that previous methods still have some underlying limitations and the challenge in analyses for unseen batches and cell types remains urgent.

Overall, translation for unseen batches and cell types is still a problem not well addressed. While many studies have provided solutions, there still exist potential problems or limitations. Following the reviewer's comment, we reorganized our description of motivation. We would like to express our gratitude to the reviewer for this valuable suggestion again and apologize for the weak description of the motivation.

MINOR COMMENT 10

"Fifth, extensive applications of cross-modality translators have not been fully investigated, such as integrative analysis of single-modality data with its predicted profiles of other modalities, enhancement of single-cell multi-omics data, cell type annotation of challenging modalities such as scATAC-seq data, and consecutive translations among multiple modalities." Many previous methods can do one or more of these tasks. Besides, the authors mentioned "cell type annotation of challenging modalities such as scATAC-seq data". This is confusing because scATAC-seq is not good data for cell type annotations. After integrating mRNA and ATAC data, we can directly use mRNA to identify cell types. Of course, the authors can do these to demonstrate the model performance, but I wonder if it provides any biological insights.

RESPONSE TO MINOR COMMENT 10

We appreciate the thoughtful comment from the reviewer and apologize for any confusion in the description of the motivation. We fully agree with the reviewer's viewpoint that many previous methods could address one or more of the tasks, including integrative analysis[55-57], data enhancement[58, 59], and consecutive cross-modal translation[45]. However, our intention was to highlight the application of cross-modality translation has not been comprehensively and systematically evaluated yet, rather than

suggesting that the existing methods cannot solve individual tasks. For example, BABEL[45] can perform consecutive translations and sciPENN[60] can perform label transfer. However, to the best of our knowledge, none of these methods have carried out a comprehensive and systematic evaluation of multiple applications. In summary, we believe that there is still a need for further exploration of how to fully leverage the results of cross-modal translation for multiple applications.

Regarding the use of ATAC data for cell type annotation, firstly, we apologize that the lack of description of the usage context in the original manuscript led to misunderstandings. We aimed to simulate a scenario that utilizes unlabeled multi-omics data along with labeled RNA-only data to annotate unlabeled ATAC-only data. In other words, in a general practical scenario, the ATAC data to be annotated is single modality data without corresponding RNA data. Our experiments have demonstrated that utilizing scButterfly translated profiles could achieve more accurate annotation results compared to state-of-the-art ATAC annotation methods EpiAnno[61] and Cellcano[62] (Fig. 4e), showcasing the powerful potential of scButterfly's cross-modality translation.

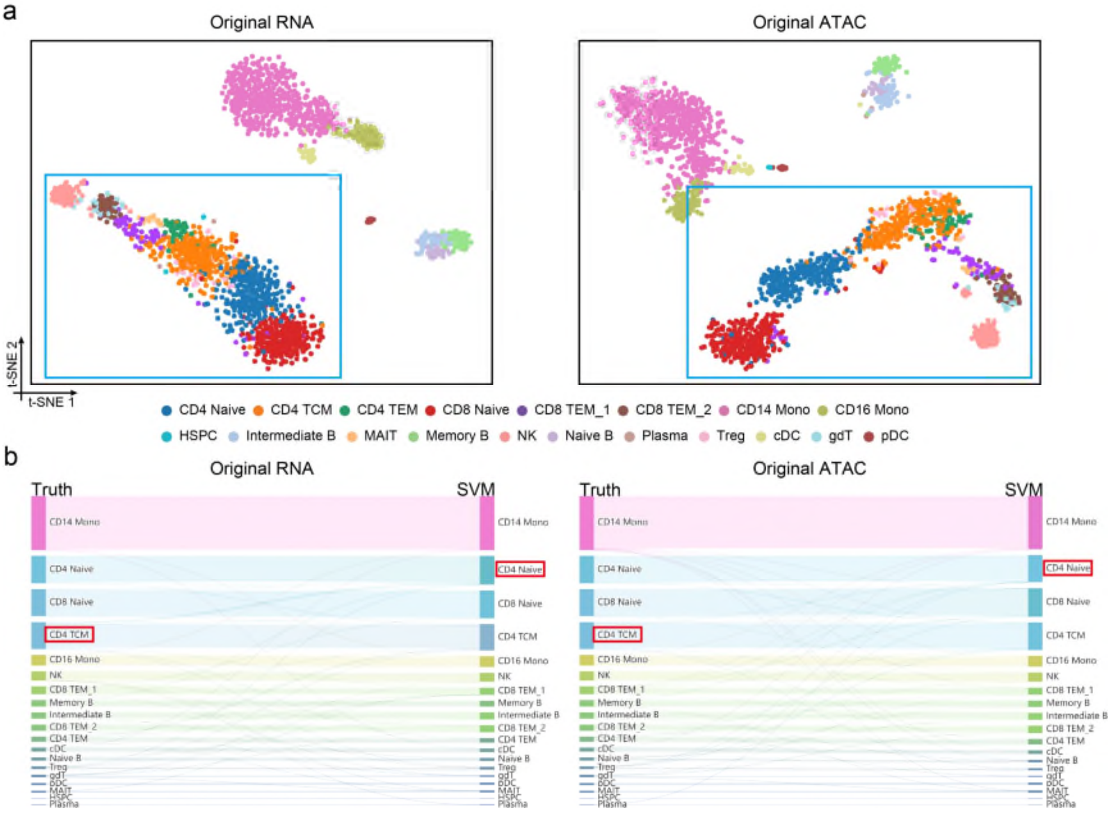

Figure R11 | Visualization and annotation performance of SVM for the original RNA and ATAC data. a, t-SNE visualizations of cells in the first test fold of five-fold cross-validation on the PBMC dataset using the original RNA and ATAC profiles. **b**, Illustration of the cell type annotation results for RNA and ATAC data of the first fold in PBMC dataset by SVM classifiers.

Secondly, we fully acknowledge the reviewer's suggestion that cell-type annotations based on RNA data are relatively more straightforward, accurate, and widely used. However, cell-type annotations based on ATAC data could also provide biological insights. We would like to clarify that even though ATAC data may not be ideal for cell type annotation purposes, for single modality ATAC data, cell type annotation is necessary for performing comprehensive analyses specific to chromatin accessibility. Existing studies have already proposed numerous approaches to reveal novel mechanistic and evolutionary insights about the functional landscape of the genome[33, 34, 63-68], making accurate annotations of ATAC data even more essential. By cross-modality translation, ATAC data could be translated into RNA data, which usually contains more annotated samples and a richer cellular atlas. Thirdly, we believe that RNA and ATAC data contain different information on cellular heterogeneity. While RNA data is better suited for annotation, it cannot capture the chromatin landscape of regulatory transcription and only provides gene expression information for identified cell types, making it difficult to reveal the upstream gene regulatory mechanisms[33]. Besides, the annotated labels from RNA and ATAC data are usually complementary to each other. For example, Fig. R11a demonstrated that on the original ATAC data, we could accurately distinguish cell types CD4_Naive and CD8_Naive, whereas they couldn’t be completely separated on the paired RNA data. This also implies that ATAC data provide new biological insights for annotation. We further trained support vector machines (SVM) classifiers both for the original RNA and ATAC data on the PBMC dataset, since SVM is a widely used baseline cell type annotation method of RNA data[69-71]. The experimental results (Fig. R11b) indicate that the ATAC-trained SVM classifier could accurately annotate CD4_TCM cells, whereas the RNA-trained SVM classifier mis-annotated a small number of CD4_TCM cells as CD4_naive cells, demonstrating that ATAC data can provide biological insights different from RNA data, and thus, annotation for both modalities is meaningful.

In summary, we would like to once again express our appreciation for the reviewer’s comment, which helped us improve our manuscript. We also apologize for any confusion in the motivation section of our manuscript. We have revised the writing of the motivation and provided a more detailed description of the usage scenario for ATAC annotation in Section “scButterfly facilitates integrative analysis, data enhancement, and cell type annotation”.

MINOR COMMENT 11

In the equation of L_D, the authors stated that "randomly assigning target labels 0 and 1 to the values following uniform distribution." What is the "values" mentioned here? How to connect this sentence to the equation? The authors should make these contents more understandable.

RESPONSE TO MINOR COMMENT 11

We appreciate the reviewer for this insightful comment and apologize for any confusion caused by our description. Here the "values" refer to the random numbers sampled from U[0, 0.2] and U[0.8, 1], respectively for the cases where the target labels are 0 and 1. In the calculation of L_D we utilize the soft labels technique to improve the robustness of the discriminator, as suggested in [72]. Specifically, before calculating the L_D, we first sample the smoothed positive label $l_{pos} \sim U[0.8,1]$ and negative label $l_{neg} \sim U[0,0.2]$, respectively for the labels 1 and 0. Subsequently, we derive the loss for the discriminator L_{Dis} as follow:

$$L_{Dis} = L_D\left(Dis_a\left(T_{r\to a}(En_r(\mathbf{X}_r))\right), l_{neg}\right) + L_D\left(Dis_a(En_a(\mathbf{X}_a)), l_{pos}\right)$$

$$+L_D\left(Dis_r\left(T_{a\to r}(En_a(\mathbf{X}_a))\right), l_{neg}\right) + L_D\left(Dis_r(En_r(\mathbf{X}_r)), l_{pos}\right)$$

Following the suggestion of the reviewer, we rewrote the explanation about the soft labels technique and the equation of discriminator loss in Section "The training procedure of scButterfly". We once again extend our sincere gratitude to the reviewer and apologize for the confusing description in the original manuscript.

COMMENT 12

In the section "Single-cell perturbation-response prediction with scButterfly". What if multiple control cells pair with the same stimulated cell? Did the authors reuse the same cell multiple times? The authors should provide these details in the method section.

RESPONSE TO COMMENT 12

We appreciate the reviewer for this insightful comment and apologize for the missing details about this part. In Section "Single-cell perturbation-response prediction with scButterfly", we applied the optimal transport to match stimulated cells with control cells to generate paired training samples. We computed an optimal transport matrix for each cell type and matched each control cell with the highest-weighted stimulated cell,

even if that stimulated cell had already been assigned to another control cell. To address the concern about reusing for stimulated cells, we would like to first clarify that scButterfly may reuse the same cell multiple times, but this occurs solely when the count of the control cell is significantly larger than the count of the stimulated cell. For example, we counted the amount of reusing for each cell type on the PT_PBMC dataset. As shown in Fig. R12a, the results illustrate that a large number of reusing for stimulated cells only occurs in cell types of CD14+Mono and Dendritic, due to the over-numbering of control cells compared to stimulated cells. Besides, we further considered an alternative matching approach without reusing stimulated cells. Specifically, for a cell type k, we initially computed the optimal transport matrix $\boldsymbol{\gamma}_k$ as introduced in Section "Single-cell perturbation-response prediction with scButterfly". Subsequently, we formulated the problem as an assignment problem and selected an assignment method that maximizes the total weight. Mathematically, the problem can be expressed as:

$$\mathbf{A_k} = \underset{\mathbf{A}}{\operatorname{argmax}} < \mathbf{A}, \boldsymbol{\gamma}_k >_F$$

$$s.t. \begin{cases} \sum_{j=1}^{n_k^{sti}} \mathbf{A}[i][j] = 1, \ \ \forall i \\ \sum_{i=1}^{n_k^{ctr}} \mathbf{A}[i][j] = 1, \ \ \forall j \\ \mathbf{A}[i][j] \in \{\mathbf{0}, \mathbf{1}\}, \ \ \forall i, j \end{cases}$$

where $\mathbf{A}_k$ represents the best assignment for control and stimulated data of type k and $< \mathbf{A}, \boldsymbol{\gamma}_k >_F$ is the Frobenius inner product. Finally, we paired the profile of control cell i with the profile of stimulated cell j if $\mathbf{A}_k[i][j]$ equals to 1, and generated the paired sample for the training of scButterfly, denoted as scButterfly-T-no-repeat.

We evaluated the performance of scButterfly-T-no-repeat for the challenging out-of-sample prediction on the PT_PBMC dataset. As shown in Fig. R12b, there was no significant difference between scButterfly-T-no-repeat and scButterfly-T, suggesting that reusing had no negative impact on the performance of scButterfly on the datasets used in our experiment.

We once again appreciate the valuable comment of the reviewer. We have added detailed information about generating paired training samples perturbation-response

prediction in Section "Single-cell perturbation-response prediction with scButterfly" and provided scButterfly-T-no-repeat as a variant of scButterfly for users.

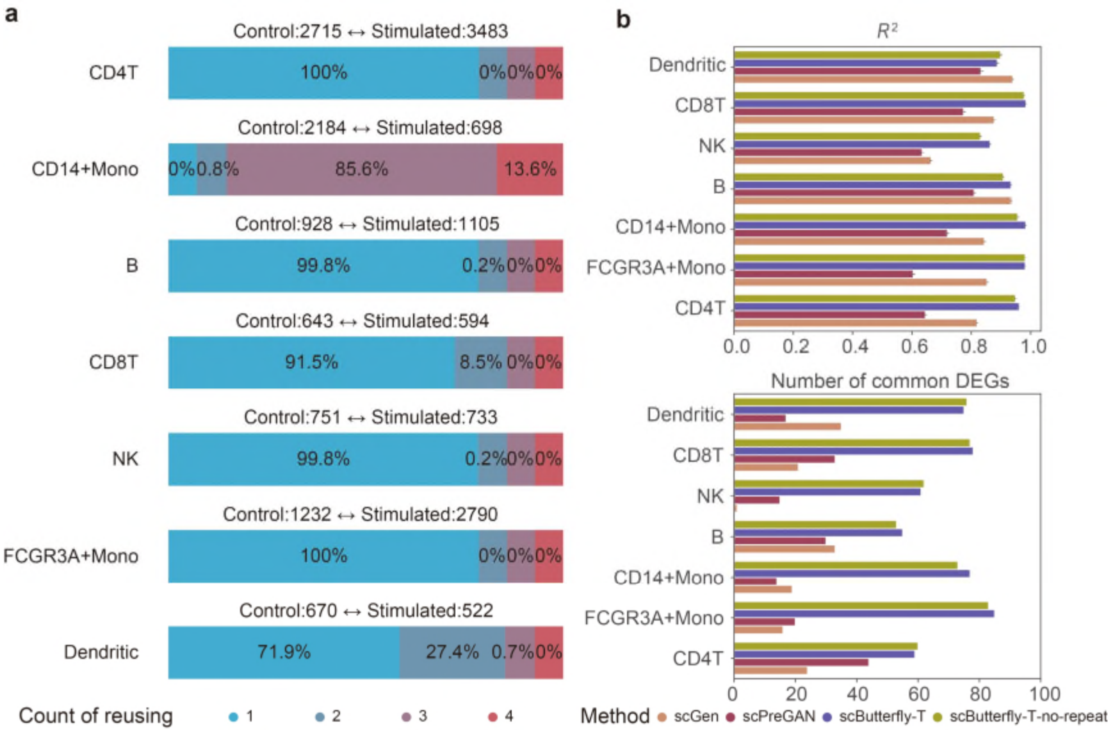

Figure R12 | Count of reusing in sample generating in perturbation-response prediction and the performance of scButterfly-T-no-repeat. a, Proportion of reusing for stimulated cells in sample generating in perturbation-response prediction, for each cell type on the PT_PBMC dataset. The counts of control cells and stimulated cells in original data are marked upon each bar. **b**, Performance of single-cell perturbation-response prediction on the PT_PBMC dataset, evaluated by the number of common DEGs of the top 100 (real) DEGs between the control data and the real stimulated data versus the top 100 (predicted) DEGs between the control data and the predicted stimulated data and the R2 values for mean gene expression of the top 100 (real) DEGs between predicted and real stimulated cells for each cell type. Center values show the mean of R2 values estimated using 100 times of random subsampling at 80% and error bars depict standard deviation.

COMMENT 13

The authors pretrained the model for 100 epochs and integrative trained the model for 200 epochs with early stops. What are the criteria for the early stops? When will the model converge (in pretraining and integrative training)? The authors should provide more details.

RESPONSE TO COMMENT 13

We appreciate the reviewer's comment and apologize for not explaining the details of convergence and the criteria for the early-stop in the original manuscript. We employ

the early-stop with a patience of 50 for both the pretraining phase and the integrative training phase. Specifically, for each epoch, we calculate and record the average reconstruction loss among all mini-batches, as described in Section “The training procedure of scButterfly”. If there is no decrease in the average reconstruction loss in the continuous 50 epochs, then we terminate the current training process prematurely and revert the model to the recorded model before 50 epochs, which achieves the lowest historical reconstruction loss.

Fully respecting the reviewer’s opinion, we further conducted the sensitivity analyses of the training epochs and early-stop patience to give more details about convergence. As described in Section “The training procedure of scButterfly”, we separately pretrained the encoders and decoders for different modalities with 100 epochs and integratively trained the model for 200 epochs, with 50 epochs patience of early-stop. We denoted the pretraining epochs for RNA and ATAC, the epochs for integrative training, and the patience as w_{pre-r}, w_{pre-a}, w_{integ}, $w_{patience}$, respectively. We generated 40 different hyperparameter settings by changing one parameter at a time. Specifically, we selected the range of w_{pre-r} and w_{pre-a} from 50 to 150 with step of 10, the range of w_{integ} from 100 to 300 with a step of 20, and the range of $w_{patience}$ from 0 to 100 with a step of 10, based on the default settings of $(w_{pre-r}, w_{pre-a}, w_{integ}, w_{patience}) = (100, 100, 200, 50)$. We conducted the five-fold cross-validation on the MCC dataset to evaluate the translation performance of scButterfly-B with different hyperparameter settings. The results are shown in Fig. R13, with the hyperparameter denoted like $(w_{pre-r}, w_{pre-a}, w_{integ}, w_{patience})$ and the default settings marked with yellow. scButterfly exhibited relative robustness to the epochs of pretraining and integrative training, implying that under different parameter settings about the training epochs, scButterfly had consistently excellent performance (Columns #1, #2, and #3 in Fig. R13). It is worth noting that the model performance was bad when the patience parameter was set below 30, while the performance tended to be stable when the patience gradually increased to 50 (Column #4 in Fig. R13). This is intuitive and may be due to the fact that, when the patience is small, the model is more likely to stop training when it can’t improve in a small number of epochs, resulting in insufficient training of the model. Therefore, we think the default setting of patience is reasonable.

In summary, we believe that the model could achieve satisfactory convergence with the default settings about the epochs and early-stop patience. Moreover, the scButterfly model is insensitive to the three parameters of training epochs

$w_{pre-r}, w_{pre-a}, w_{integ}$, as well as the patience of early-stop $w_{patience}$ within the range from 30 to 100. We would like to express our gratitude to the reviewer again and apologize for the lack of details about the early-stop. We have added more comprehensive information about the early-stops and sensitive analyses in Section "scButterfly enables cross-modality translation while preserving cell heterogeneity".

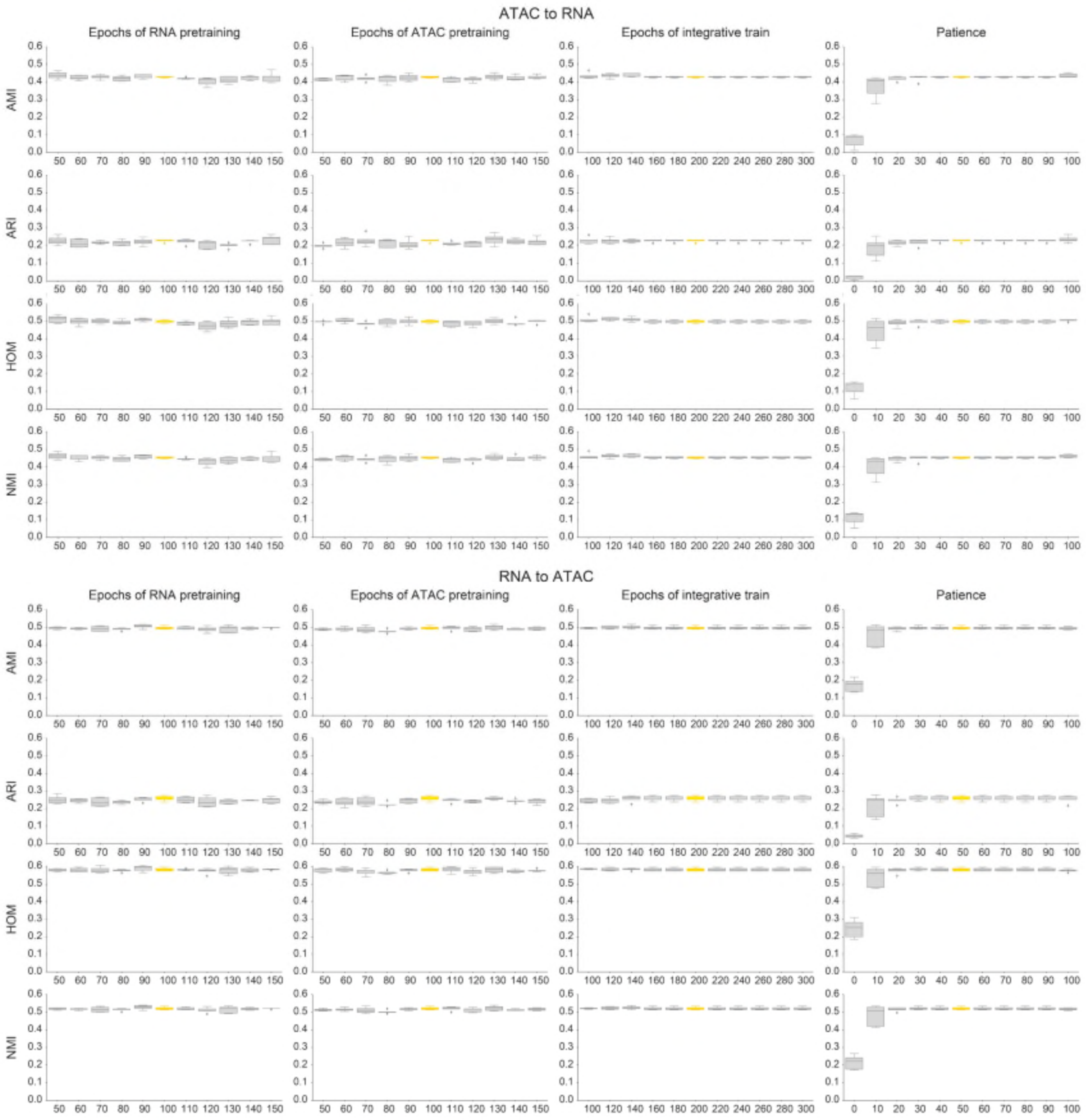

Figure R13 | Performance of scButterfly with different epochs and patience on the MCC dataset. Evaluation of translation performance for scButterfly-B under different training epochs and early-stop settings with five-fold cross-validation on the MCC dataset. The boxplots marked with yellow denote the default hyperparameter setting of scButterfly-B. In the boxplots, the center lines, box limits and whiskers denote the median, upper and lower quartiles, and 1.5× interquartile range, respectively.

Minor comment 14

7) In Fig 1, there are two windows of the translator: a brief window in the butterfly and a detailed window aside. However, the first one has two outputs and the second one has four outputs. This is confusing. I recommend that the authors ensure consistency or provide an explanation in the legend.

Response to minor comment 14

We appreciate the reviewer's keen insight and apologize for the confusion in Fig. 1b. Here the two outputs in the brief window separately refer to RNA to ATAC translated embedding and ATAC to RNA translated embedding, while the four outputs in the detailed window refer to the RNA to RNA mapped embedding, ATAC to RNA translated embedding, RNA to ATAC translated embedding and ATAC to ATAC mapped embedding, respectively. As stated in Section "The basic model of scButterfly", the translator T takes the original RNA embedding $En_r(\mathbf{X}_r)$ and following generates the reconstructed RNA embedding $T_{r\to r}\left(En_r(\mathbf{X}_r)\right)$ as well as the translated ATAC embedding $T_{r\to a}\left(En_r(\mathbf{X}_r)\right)$. Similarly, it also takes the original ATAC embedding $En_a(\mathbf{X}_a)$ and then produces the reconstructed ATAC embedding $T_{a\to a}\left(En_a(\mathbf{X}_a)\right)$ along with the translated RNA embedding $T_{a\to r}\left(En_a(\mathbf{X}_a)\right)$. In the detailed window of the translator shown in Fig. 1b, we depicted these four outputs from top to bottom, separately representing the reconstructed ATAC embedding $T_{a\to a}\left(En_a(\mathbf{X}_a)\right)$, the translated ATAC embedding $T_{r\to a}\left(En_r(\mathbf{X}_r)\right)$, the reconstructed RNA embedding $T_{a\to r}\left(En_a(\mathbf{X}_a)\right)$, and translated RNA embedding $T_{r\to r}\left(En_r(\mathbf{X}_r)\right)$. In the revised manuscript, we have made changes to the translator in the brief window of scButterfly in Fig. 1b to ensure consistency and avoid any further confusion.

REVIEWER #3

For a technical perspective, there is no difference in the method between this paper and the other paper (published on https://proceedings.neurips.cc/paper_files/paper/2022/hash/662b1774ba8845fc1fa3d1fc0177ceeb-Abstract-Conference.html), although the proposed scbutterfly is applied to multiple datasets.

RESPONSE

We deeply appreciate the reviewer for this knowledgeable comment. Although scButterfly and cross-linked unified embedding[73] (CLUE) are both VAE-based models, scButterfly differs from CLUE in terms of application scenarios, basic assumptions, model structure, implementation details, and model extensions. We have provided a detailed description of the differences between the two methods in Response to Comment #1 of Reviewer #3.

COMMENT 1

What distinguishes the proposed scButterfly from the paper titled 'Cross-Linked Unified Embedding for Cross-Modality Representation Learning'? It appears that both methods are grounded in variational autoencoders, and the translation is accomplished through cross-domain autoencoders within the latent/semantic feature space.

RESPONSE TO COMMENT 1

We deeply appreciate this insightful comment from the reviewer. We would like to first acknowledge that both scButterfly and CLUE are based on a variational autoencoder (VAE) framework. VAE framework is widely used in relevant studies, especially in the models for single-cell multi-omics data. For instance, multiple methods designed for analyzing single-cell multi-omics data are based on the VAE framework, such as totalVI[18], SCALEX[74], GLUE[75], and scVI[76]. All of these methods are modified based on the VAE framework, enabling various models to be better applied to different scenarios, implying that VAE is a powerful and flexible framework for handling multi-omics data. For the problem of cross-modality translation, many methods are also grounded in variational autoencoders and accomplish the translation within the latent feature space: BABEL combines two autoencoders to embed different modality profiles and makes cross-modal inferences by simultaneously minimizing two reconstruction

losses and two cross-prediction losses[45]. Polarbear achieves translation by connecting the pre-trained frozen encoder for one modality with the decoder for another modality, using a fully-connected layer[46]. JAMIE develops joint VAEs for multi-modal imputation and embedding using cross-modal correspondence[47]. Therefore, we also chose the VAE framework as the foundation and accomplished the translation within the latent feature space.

Furthermore, we concluded that there are five main differences between scButterfly and CLUE, including (1) the main task of the model, (2) the basic assumptions for latent space, (3) the model structure such as the translator and encoders, (4) the loss function, and (5) the extensions of the model. We provided a detailed explanation for each point as follows:

Firstly, it is important to clarify that although scButterfly and CLUE are both designed for single-cell multi-omics data, their main tasks are different. Following the classification of multi-omics tasks defined in the NeurIPS 2021 Multimodal Single-cell Data Integration Competition[77], CLUE primarily focuses on "modality matching" and "joint embedding", whereas scButterfly is mainly responsible for "modality prediction". "Modality matching" is to identify the mutual correspondences between single modality data, while "joint embedding" aims to map multimodal data into a unified latent space. On the other hand, "modality prediction" requires methods to utilize profiles from one modality to predict the profiles in another modality. Specifically, the "modality matching" and "joint embedding" tasks allow methods to perform dimension reduction on the data, whereas the "modality prediction" task needs to predict the profiles of one modality with the profiles of another modality. In brief, although both scButterfly and CLUE are based on the VAE framework, they are designed to handle different tasks.

Secondly, scButterfly and CLUE have different underlying assumptions about latent spaces. Although the two models both consider the modality-specific information in the shared latent spaces, their assumptions regarding these spaces are different from each other. To be specific, let the $\mathbf{x}_k^c \in \mathbb{R}^{m_k}$ denote the sample from cell c with m_k features of the modality $k \in \{1, \dots, K\}$, and the $\mathbf{z}^c$ and $\mathbf{z}_k^c$ separately indicate the variables in the shared latent space and the modality k specific latent space. CLUE assumes that the variable $\mathbf{z}^c$ of the shared latent space is simply a combination of modality-specific space variables $\mathbf{z}^c = (\mathbf{z}_1^c, \dots, \mathbf{z}_K^c)$. While in scButterfly, we model the modality-specific latent space variables $\mathbf{z}_k^c$ as a generative distribution $p(\mathbf{z}_k^c|\mathbf{z}^c)$

from the shared latent space variable $\mathbf{z}^c$, which allows for better balancing of modality-specific and modality-shared information (Fig. R13).

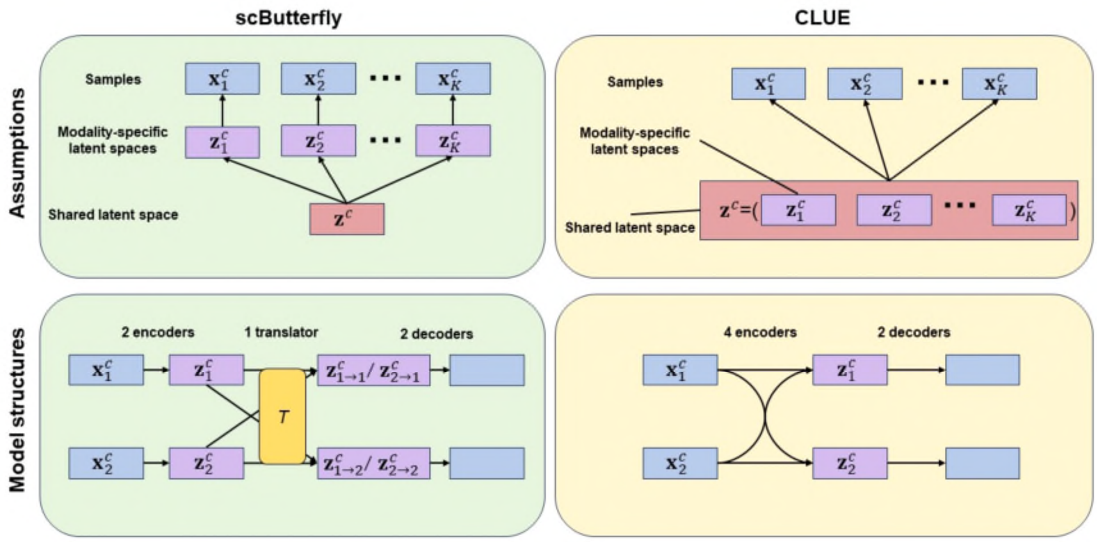

Figure R13 | Differences between scButterfly and CLUE for the assumptions of the embedding spaces and model structures. a, CLUE assumes the shared latent space is a combination of modality-specific latent spaces, while scButterfly models the modality-specific latent spaces as a generative model shared latent space. **b**, CLUE uses multiple encoders for cross-modality mapping, while scButterfly utilizes the translator to translated between the modality-specific latent spaces.

Thirdly, scButterfly and CLUE could be distinguished in terms of their model structures. In CLUE, the input $\mathbf{x}_i^c$ of the modality i is directly encoded to the modality j specific latent space variable $\mathbf{z}_{i\to j}^c$ with an encoder $En_{i\to j}$. For scButterfly, the input $\mathbf{x}_i^c$ of the modality i is first encoded to the modality i specific latent space with an encoder En_i. Then a translator $T_{i\to j}$ is used to convert $\mathbf{z}_i^c = En_i(\mathbf{x}_i^c)$ to the modality j specific latent space variable $\mathbf{z}_{i\to j}^c = T_{i\to j}\big(En_i(\mathbf{x}_i^c)\big)$ (Fig. R13). Illustrate the mapping from RNA to ATAC as an example, in the CLUE, the original RNA data $\mathbf{x}_r^c$ is encoded using the RNA to ATAC encoder to map it directly to the modality-specific latent space of ATAC $\mathbf{z}_{r\to a}^c$, which is then decoded using the ATAC decoder De_a. However, in the scButterfly, the RNA data is first encoded using the RNA encoder En_r to map it to the RNA-specific latent space $\mathbf{z}_r^c = En_r(\mathbf{x}_r^c)$, then the translator T is used to map it to the translated ATAC-specific latent space as $\mathbf{z}_{r\to a}^c = T_{r\to a}(\mathbf{z}_r^c)$ for decoding. Additionally, both scButterfly and CLUE employ discriminators. CLUE's discriminators aim to differentiate the modality-specific latent

spaces obtained from different modalities, e.g. $\mathbf{z}_{i\to k}^{c}$ and $\mathbf{z}_{j\to k}^{c}\,(i\neq j)$, while the discriminators of scButterfly focus primarily on distinguishing between original modality-specific latent space variables $\mathbf{z}_{k}^{c}$ and reconstructed modality-specific latent space variables $\mathbf{z}_{j\to k}^{c}=T_{j\to k}(\mathbf{z}_{j}^{c})$. Besides, scButterfly also employs various specialized model structures. For example, we prune the inter-chromosomal connections for the ATAC encoder and ATAC decoder, and adopt a masking strategy for the input data, which is not mentioned in the CLUE paper.

Fourthly, scButterfly and CLUE also differ in the design of the loss functions. scButterfly and CLUE both contain the reconstruction loss and the evidence lower bound (ELBO) for the VAE framework. However, CLUE also calculates the mean squared error (MSE) on the shared latent space to increase the similarity between the representations of shared latent space from different modalities. To be specific, CLUE obtains the shared latent space from modality k, denoted as $\mathbf{z}_{k\to}^{c}=(\mathbf{z}_{k\to 1}^{c},\ldots,\mathbf{z}_{k\to K}^{c})$. Then mean of these representations of shared latent space is calculated as $\overline{\mathbf{z}_{k\to}^{c}}=\frac{1}{K}\sum_{k=1}^{K}\mathbf{z}_{k\to}^{c}$. At last, the MSE between $\mathbf{z}_{k\to}^{c}$ and $\overline{\mathbf{z}_{k\to}^{c}}$ is calculated for each modality k and the sum of these loss is added to the loss function for CLUE training. However, scButterfly does not incorporate such restrictions on the modality-specific latent spaces obtained from different modalities.

Fifthly, we proposed two kinds of data augmentation strategies based on cluster labels (scButterfly-C) and cell-type labels (scButterfly-T), respectively. These methods can further enhance the translation performance and address the challenges arising from the limited number of training cells, compared to the basic scButterfly model (scButterfly-B). However, in the paper of CLUE, we did not find similar extensions for the model.

Furthermore, in the paper “Cross-Linked Unified Embedding for Cross-Modality Representation Learning”, the authors demonstrated the impressive performance of the CLUE model on the modality matching and joint embedding tasks but did not mention its applicability to cross-modality translation. After carefully examining the CLUE code, we found that the “scglue.models.scclue.SCCLUEModel.cross_predict” function could be used for cross-modality translation. We also found that the “scglue.models.configure_dataset” function required specifying the distribution for the decoder, however, we did not find any information about the principle for choosing

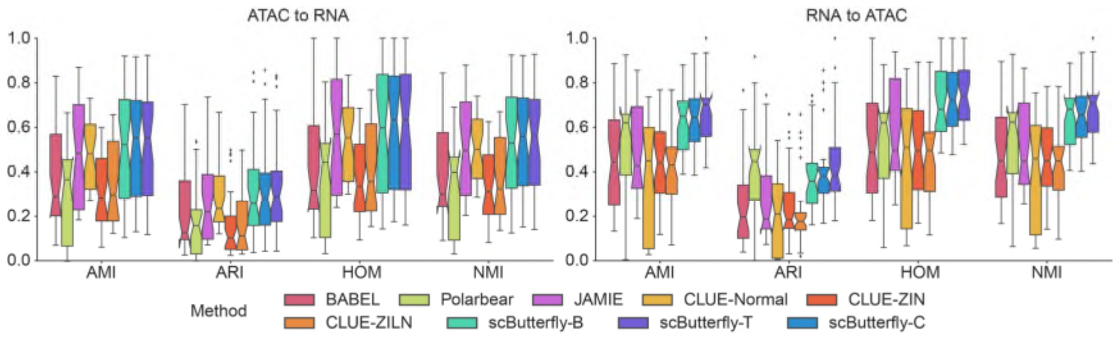

Figure R14 | Cross-modality translation performance on multiple datasets. Quantitative evaluation of the translated profiles for preserving cell heterogeneity in five-fold cross-validation by cell on the BMMC, MB, CL, MCC, MK, MDS and PBMC datasets, via cell clustering with metrics of AMI, ARI, HOM and NMI. In the boxplots, the center lines, box limits, whiskers and notches denote the median, upper and lower quartiles, 1.5× interquartile range and 95% confidence interval calculated using a Gaussian-based asymptotic approximation, respectively. Note that the boxes of JAMIE only contain 30 data points from six datasets since JAMIE encountered GPU memory errors on the BMMC dataset.

distribution for cross-modality translation with CLUE. Due to the lack of a tutorial, we attempted to use each of their provided five distributions including “Normal”, “ZIN”, “ZILN”, “NB” and “ZINB”. Among them, CLUE was able to run successfully with distributions of “Normal”, “ZIN” and “ZILN”, separately denoted as CLUE-Normal, CLUE-ZIN, and CLUE-ZILN. For the distribution of “NB”, we encountered the “RuntimeError: Cannot use NB prob model for cross prediction!”, while for the distribution of “ZINB”, we encountered the “KeyError: 'ZINB'”. We conducted five-fold cross-validation on seven paired datasets (BMMC, MB, CL, MCC, MK, MDS, and PBMC) to evaluate the performance of different methods, and the results illustrated that scButterfly-B (-C, -T) significantly outperformed the CLUE model with three different kinds of distribution (Fig. R14).

In conclusion, although scButterfly and CLUE share similarities in the use of the VAE framework and translation in the modality-specific latent spaces, they distinguish in the main task, the assumptions about the latent spaces, the model structure, the design of the loss functions, and the extensions of the models. We have added an introduction to CLUE in Section “introduction”.

COMMENT 2

The incorporation of multi-omics data poses a challenge due to its high dimensionality and technical variability. How can you ascertain scButterfly's ability to handle high-dimensional multi-omics data? It is recommended to validate the model using varying numbers of features.

RESPONSE TO COMMENT 2

We greatly appreciate the knowledgeable comment from the reviewer. Indeed, as pointed out by the reviewer, performing multi-omics analyses is usually challenging due to the varied assay types[56, 78] and high dimensionality[79, 80]. To address the issue of high dimensionality, we have made efforts from multiple aspects: (1) we performed feature selection through pre-processing to reduce the dimensionality of the features. For RNA data, we chose to retain the top 3000 highly variable genes (HVGs) for model training. For ATAC data, we filtered out the peaks that are accessible less than 0.5% of cells, as commonly employed in previous research[27, 61, 81-83]. (2) We designed the models based on the autoencoder framework to map the high-dimensional features to a lower-dimensional embedding space, which has been widely applied to solve high-dimensional issues in previous multi-omics researches[45-47, 60, 84, 85]. By mapping the high-dimensional data to a lower-dimensional space, we could achieve cross-modality translation between the modality-specific embedding space and then recover the dimensions with the decoder. (3) For the high-dimensional modality such as ATAC data, inspired by the insight that most chromatin accessibility interactions occur at an intra-chromosomal level[86], we pruned the inter-chromosomal connections in the first layer of the encoder and the last layer of decoder to simplify the model.

To explore the performance of scButterfly on high-dimensional multi-omics data, we have evaluated the model on seven datasets with different dimensions, protocols, species, and tissues (Supplementary Fig. 2). Specifically, we collected seven paired RNA and ATAC datasets. The MB and MDS datasets have the highest dimensions of ATAC profiles among these seven datasets, containing SHARE-seq[14] profiles with high dimensions ATAC data with 428,041 and 340,341 peaks from the adult mouse brain and dorsal skin respectively. The CL and MK datasets are separately composed of the multiple cancer cell lines profiles from scCAT-seq[12] and adult mouse kidney profiles from sci-CAR[87], both with the highest dimensions of RNA profiles among these seven datasets with about 49,000 genes. We also incorporated the BMMC dataset with large counts of bone marrow mononuclear samples with 10x-Multiome[77], the adult mouse cerebral cortices dataset MCC[13], and the peripheral blood mononuclear dataset PBMC, which are widely used in previous multi-omics studies[45, 46, 75, 88]. Furthermore, we also

collected eight unpaired datasets[89-92] to evaluate the performance of scButterfly for diagonal analysis, with the dimensions of RNA data ranging from 27,123 to 63,561 and the dimensions of ATAC data ranging from 99,019 to 1,050,819. In the original manuscript, we have demonstrated the advantage of scButterfly over other baseline

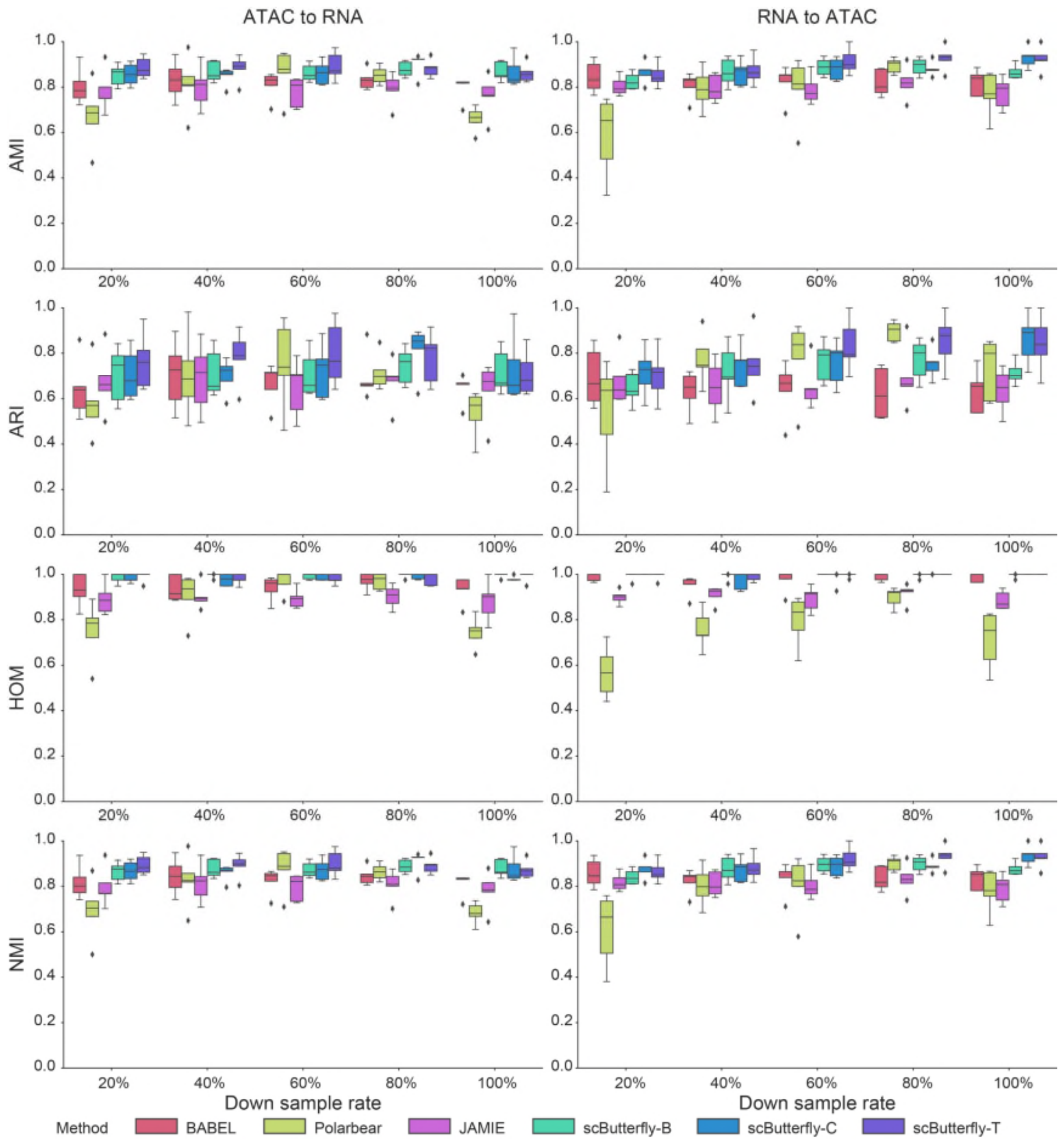

Figure R15 | Performance of different methods under the scenarios with varying numbers of features. Evaluation of the cross-modality translation performance for data with different rate of random down sampled features, including five-fold cross-validation by cell experiments in the CL dataset, via cell clustering with metrics of AMI, ARI, HOM and NMI. In the boxplots, the center lines, box limits, whiskers and notches denote the median, upper and lower quartiles, 1.5× interquartile range and 95% confidence interval calculated using a Gaussian-based asymptotic approximation, respectively.

methods on these datasets (Figs. 2bc and 4f), implying the superior performance of cross-modality translation and the robustness to dimensions.

Following the reviewer's suggestion, we further down-sampled the feature of the CL dataset to evaluate the performance of scButterfly for datasets with different dimensions. We randomly selected 20%, 40%, 60%, 80%, and 100% of the features in the original data for both RNA and ATAC, and conducted five-fold cross-validation. As shown in Fig. R15, scButterfly-B outperformed other baseline methods in most settings with different dimensions. Two variants (scButterfly-C and scButterfly-T) of scButterfly-B further significantly improved the cross-modality translation performance, underscoring the superior capability of scButterfly in handling multi-omics data at various dimensions.

In conclusion, we demonstrated the powerful capabilities of scButterfly in handling high-dimensional data. We would like to express our gratitude for the reviewer's comment again, which helped us refine our manuscript. We have added the above discussions in Section “scButterfly enables cross-modality translation while preserving cell heterogeneity”.

COMMENT 3

Regarding the data augmentation method, it is advisable to analyze its effectiveness. For instance, using a single multi-omics single-cell dataset, the author should demonstrate and compare the model's performance with and without data augmentation. Furthermore, it's essential to specify how many augmented pairs/data points were generated and if there is any impact on model performance with varying numbers of augmented data.

RESPONSE TO COMMENT 3

We sincerely appreciate the insightful comment provided by the reviewer and apologize for the insufficient analyses of the data augmentation strategies and their effectiveness. In the subsequent paragraphs, we presented a comprehensive evaluation of the effectiveness of data augmentation strategies, including an assessment of the translation performance improvements achieved on multiple datasets, the adaptability of the strategies to unseen batches or cell types, and the particular efficacy when dealing with a limited number of multi-omics samples. Additionally, we investigated the impact of varying the number of augmented samples on the model's performance.

Regarding the effectiveness of data augmentation, we found that it can improve the cross-modality translation performance of scButterfly in various scenarios. Firstly, the data augmentation strategies improve the translation performance of scButterfly on multiple datasets. We conducted five-fold cross-validation on each of the seven datasets (BMMC, MB, CL, MCC, MK, MDS, and PBMC). We demonstrated the improvement of translation performance for scButterfly-C (-T) compared with the performance of scButterfly-B on the same fold in Fig. R16. Specifically, each point in violin plots represented the ratio of clustering metrics of scButterfly-C (-T) to that of scButterfly-B. On most datasets (except for the PBMC dataset and the RNA to ATAC translation on the MB dataset), the scButterfly-C variant outperformed the scButterfly-B model, with an average improvement of 5.340%. Meanwhile, scButterfly-T achieved further improvement in performance on almost all seven datasets, with an average improvement of 7.954%. We further provided a detailed one-side Wilcoxon signed-rank test result between scButterfly-B and two variants of scButterfly-C and scButterfly-T on these seven datasets (Fig. 2c), which indicated the significant improvement of the scButterfly model with data augmentation strategies.

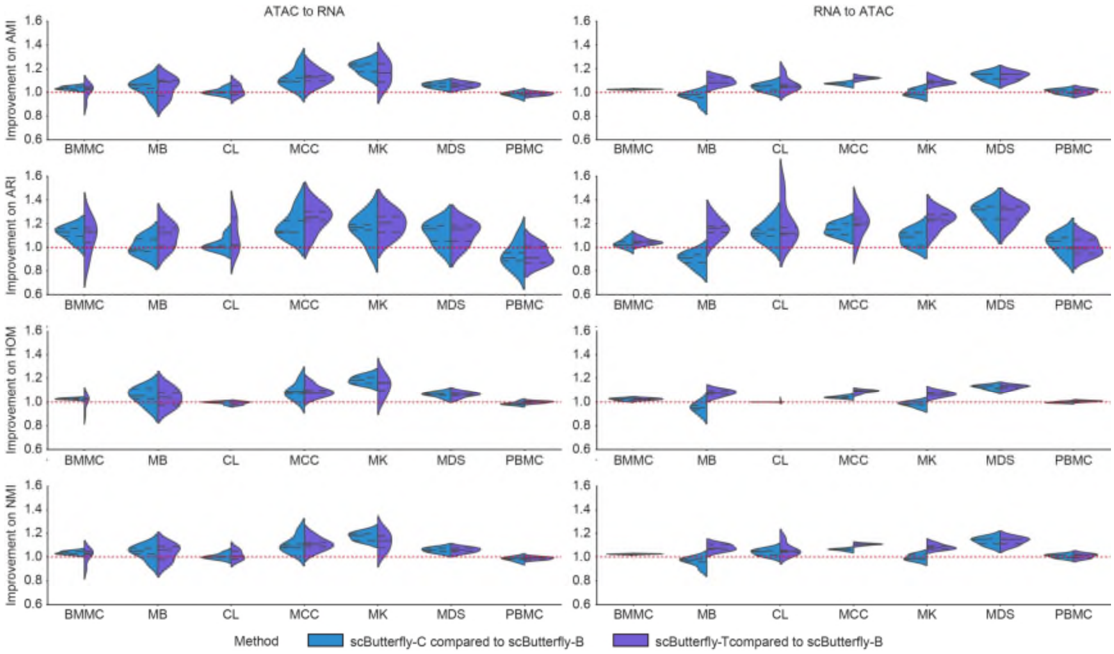

Figure R16 | Improvement of scButterfly with data augmentation based on clusters and cell types. We evaluated the performance improvement of scButterfly-C and scButterfly-T compared to scButterfly-B based on the cluster metrics AMI, ARI, HOM and NMI, on the seven datasets (BMMC, MB, CL, MCC, MK, MDS and PBMC). In the violinplots, the center lines and the upper and lower lines denote the median, upper and lower quartiles, respectively. The red dotted lines denote the position of 1, which means the equally performance compared with scButterfly-B.

Additionally, both two augmentation strategies improved the translation performance in more general scenarios, i.e. cross-modality translation for cells of novel batches or cell types. For the scenarios of translation on the unseen batches, we tested the translation performance of scButterfly-B (-C, -T) on three multi-batch datasets (BMMC, CL, and MDS). The experimental results are shown in Fig. 3b and Supplementary Fig. 9, and the p-values of the one-sided Wilcoxon signed-rank test are provided in the Supplementary Fig. 8b. These results demonstrated that scButterfly-C still significantly improves performance compared to scButterfly-B (p-value=8.41e-4) and scButterfly-T further enhances the ability of translation (p-value=2.52e-11). For the scenarios of translation on the unseen cell types, we evaluated the translation performance of scButterfly-B (-C, -T) on four datasets (MB, MCC, MK, and PBMC) and have presented the results in Fig. 3a and Supplementary Figs. 7, 8a. Both two strategies still demonstrated significant advantages compared to scButterfly-B (scButterfly-C: p-value=2.16e-11, scButterfly-T: p-value=2.77e-4), showcasing the capabilities of data augmentation.

Thirdly, the data augmentation strategies exhibit particular capabilities in handling datasets with multi-omics profiles of a limited count of cells. We conducted five-fold cross-validation on the down-sampled MCC dataset for the scButterfly-B model and two variants with data augmentation (more details in Response to Comment #8 of Reviewer #2). As shown in Fig. R9b, scButterfly-C and scButterfly-T showed a more pronounced improvement in performance compared to the scButterfly-B when the count of cells is low, suggesting that the data augmentation strategies can effectively improve the performance when there are limited cells in the training data.

For details about the data augmentation, we apologize for not clearly specifying the number of augmented pairs/data points in the original manuscript. As we introduced in Section "The data augmentation strategy of scButterfly", for scButterfly-C (-T), we shuffle the ATAC profiles within each cluster (cell type) twice to generate an artificial training dataset twice larger than the original dataset. We combine the artificial training dataset for each cluster (cell type) and mix them with the original dataset, resulting in a training dataset that is three times as large as the original dataset, for both the pretraining and integrative training phases.

We followed the reviewer's advice and discussed the impact of the number of augmented data on the model's performance. Illustrated with the five-fold cross-validation on the MCC dataset as an example, we evaluated the performance of

applying two kinds of augmentation strategies on scButterfly-B with different counts of augmented data. We augmented the dataset to w times as large as the original dataset, and chose the settings of w ranging from 1.5 to 4.5 with a step of 0.5. For non-integer times of augmentation, we kept one copy of the original data, randomly shuffled the ATAC profiles for $\lfloor w-1 \rfloor$ times and paired with unshuffled RNA profiles, and for the decimal part $w-\lfloor w \rfloor$ (e.g., 0.5), we randomly selected the corresponding

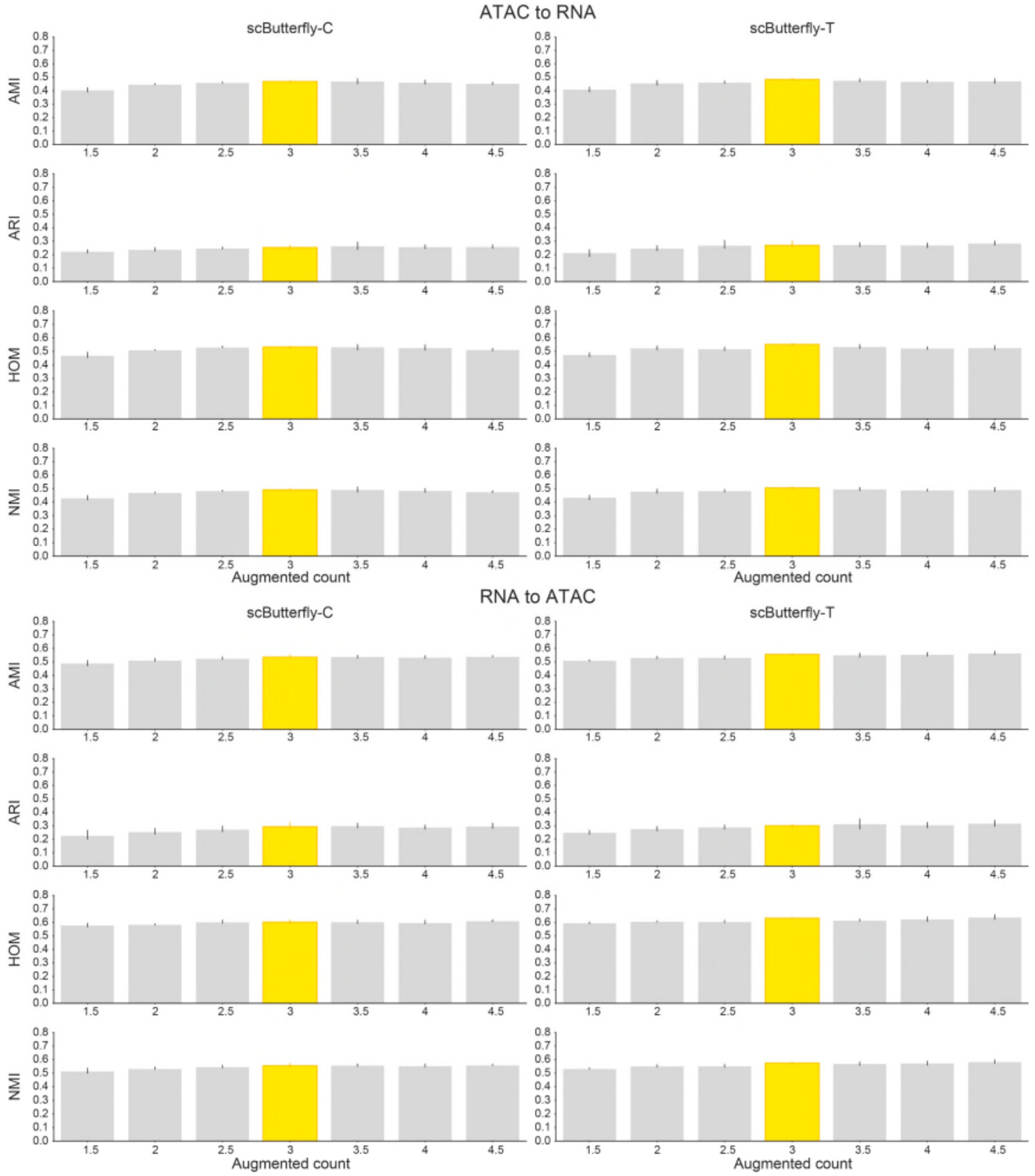

Figure R17 | Performance of scButterfly with different count of augmented data on the MCC dataset. Evaluation of translation performance for scButterfly-C and scButterfly-T with different count of augmented data with five-fold cross-validation by cell on the MCC dataset. The bars marked with yellow denote the default setting of scButterfly-C and scButterfly-T. The height of each bar denotes the median value of each metric and the error bars show 95% confidence interval.

proportion $(w - \lfloor w \rfloor) \times 100\%$ (e.g., 50%) of the augmented samples. We presented the experimental results in Fig. R15, marking our default setting (augmented the dataset to three times as the original dataset) as yellow. As w increased, the model's performance initially improved because more diverse samples were added to the training process. However, when w exceeded 3, the model's performance did not improve or even decline, possibly because the proportion of the original data was constantly diluted. In conclusion, we identified that augmenting the dataset to three times as large as the original sample size is a suitable and robust choice.

In conclusion, we would like to again express our gratitude to the reviewer for the insightful comment and apologize for any unclear descriptions in the manuscript. We have incorporated the aforementioned analysis into Section "scButterfly enables cross-modality translation while preserving cell heterogeneity" and double-checked the description in the revised version of the manuscript.

COMMENT 4

4) On line 783, it is stated that NVIDIA supports TensorFlow 1.13 for RTX 6000. However, NVIDIA provides an NGC docker with pre-compiled TensorFlow 1.15 for use on newer GPUs.

RESPONSE TO COMMENT 4

We greatly appreciate the knowledgeable comment from the reviewer and apologize for overlooking the availability of the NGC docker. In the original manuscript, we implemented Polarbear by converting the code to be compatible with TensorFlow v2.0 with the API "tf_upgrade_v2" provided by TensorFlow[93]. Strictly following the reviewer's suggestion, we updated the result for Polarbear with default settings as https://github.com/Noble-Lab/Polarbear implemented on the NGC docker with pre-compiled TensorFlow 1.15. We noticed that in the experiment of five-fold cross-validation by cell, the updated Polarbear showed significant improvement compared to the previous implementation, but still performed worse than scButterfly. Additionally, for the experiments of cross-validation by batch or cell type, the improvement of the updated Polarbear seemed to be more limited. Furthermore, we observed that the updated Polarbear performed worse on unpaired data compared to the implementation with TensorFlow v2.0. Overall, scButterfly still outperformed Polarbear in various scenarios, indicating that the implementation of Polarbear did not have any impact on our conclusions.

To ensure a fair comparison, we have updated all results related to Polarbear in the revised manuscript, supplementary, and responses to reviewers, with the results from the Polarbear ran on the NGC docker with pre-compiled TensorFlow 1.15.

REVIEWER #4

The author has introduced a framework named scButterfly, with a primary focus on cross-modality translation, particularly between transcriptome and chromatin accessibility data. The scButterfly framework comprises 7 key components: 2 encoders, 2 decoders, 1 translator, and 2 discriminators. To facilitate cross-omics translation, the framework aligned omic-specific latent spaces using the translator, in contrast to previous methods that projected them into a single shared latent space. Moreover, the author has developed two model variants scButterfly-T and scButterfly-C aimed at expanding sample size, enhancing data quality, and enabling the translation of unpaired data. To evaluate the model's performance, the author conducted a series of downstream experiments, including cell clustering on paired and unpaired data, cross-modality translation involving novel cell types and batches, data enhancement using the predicted profiles, perturbation response prediction, and consecutive translation.

The following are my comments.

RESPONSE

We deeply appreciate the reviewer for affirmation of our work and the enthusiastic assessment as well as thoughtful comments which helped us to improve the manuscript. We have carefully considered and addressed each comment, and have made revisions to the manuscript in the corresponding sections.

COMMENT 1

In line 254, the author identified two subgroups of basal cells based on the predicted profiles. However, it remains unclear how these two subgroups were identified. In supplementary Figure 10a, cells within cluster 5 are divided into two parts, yet they have been labeled as subgroup2 rather than subgroup1. Is there any special reason or rationale behind this labeling choice?

RESPONSE TO COMMENT 1

We appreciate the careful reading of the reviewer and apologize for the insufficient introduction which led to this confusion. Firstly, we clarify that our identification of two subgroups of basal cells was primarily based on the Leiden[2] clustering and t-

distributed stochastic neighbor embedding (t-SNE) visualization. Specifically, we obtained the ATAC to RNA translated profiles with scButterfly-C for the cells in the first test fold of cross-validation by batch on the MDS dataset. We then clustered all translated profiles using the Leiden clustering with the default resolution of 1. Seven clusters showed high overlapping with the original Basal cell population, including clusters #0, #2, #5, #14, #17, #22, and #28 (Fig. R18a). Based on the Leiden clustering and t-SNE visualization, we identified two subgroups of basal cells (Predicted Basal 1 and 2). We divided the cells in clusters #2, #14, and #22 into Predicted Basal 1 and the cells in clusters #0, #5, #17, and #28 into Predicted Basal 2 (Fig. R18b).

To further explain the rationale behind this identification, we visualized the cluster-specific gene expression patterns of the cells in these seven clusters. We next used the one-sided Wilcoxon rank-sum test in Scanpy[82] to find the top 3 differentially expressed genes (DEGs) for each cluster compared to the other six clusters. We visualized the gene expression patterns of these DEGs in the dotplot in Fig. R18c, columns represent differentially expressed genes, columns with the same square bracket represent DEGs belonging to a certain cluster, and rows represent different clusters. For each column, we standardized the average expression values within the range of 0 to 1 to ensure a clearer visualization. It can be observed that cluster #5 contains more similar patterns as clusters #0, #17, and #28 compared to #2, #14, and #22 (marked with blue boxes in Fig. R18c), especially for the DEGs of the clusters #0, #17 and #28 and *Gm29664* (DEG of cluster #2), *Elf2*, and *Serpinb7* (DEGs of cluster #14). These similar genes accounted for 12/21 of all DEGs of these seven clusters, so we considered it more appropriate to group cluster #5 with clusters #0, #17, and #28.

Additionally, we found that dividing cluster #5 into Predicted Basal 2 (with clusters #0, #17, and #28) could make the identified DEGs more significant. Specifically, we also attempted to divide cluster #5 into Predicted Basal 1 (with clusters #2, #14, and #22) and identified DEGs between Predicted Basal 1 and 2. As shown in Fig. R18d, the DEGs identified when cluster #5 was assigned to Predicted Basal 2 (left) exhibited a higher Z-scores compared to the DEGs identified when cluster #5 was assigned to Predicted Basal 1 (right). Therefore, we believed that assigning #5 to Predicted Basal 2 was more reasonable.

We sincerely appreciate the comment from the reviewer. We have included the aforementioned details in Section “scButterfly effectively translates data of novel

contexts and reveals biological insights” to further improve the readability of the manuscript.

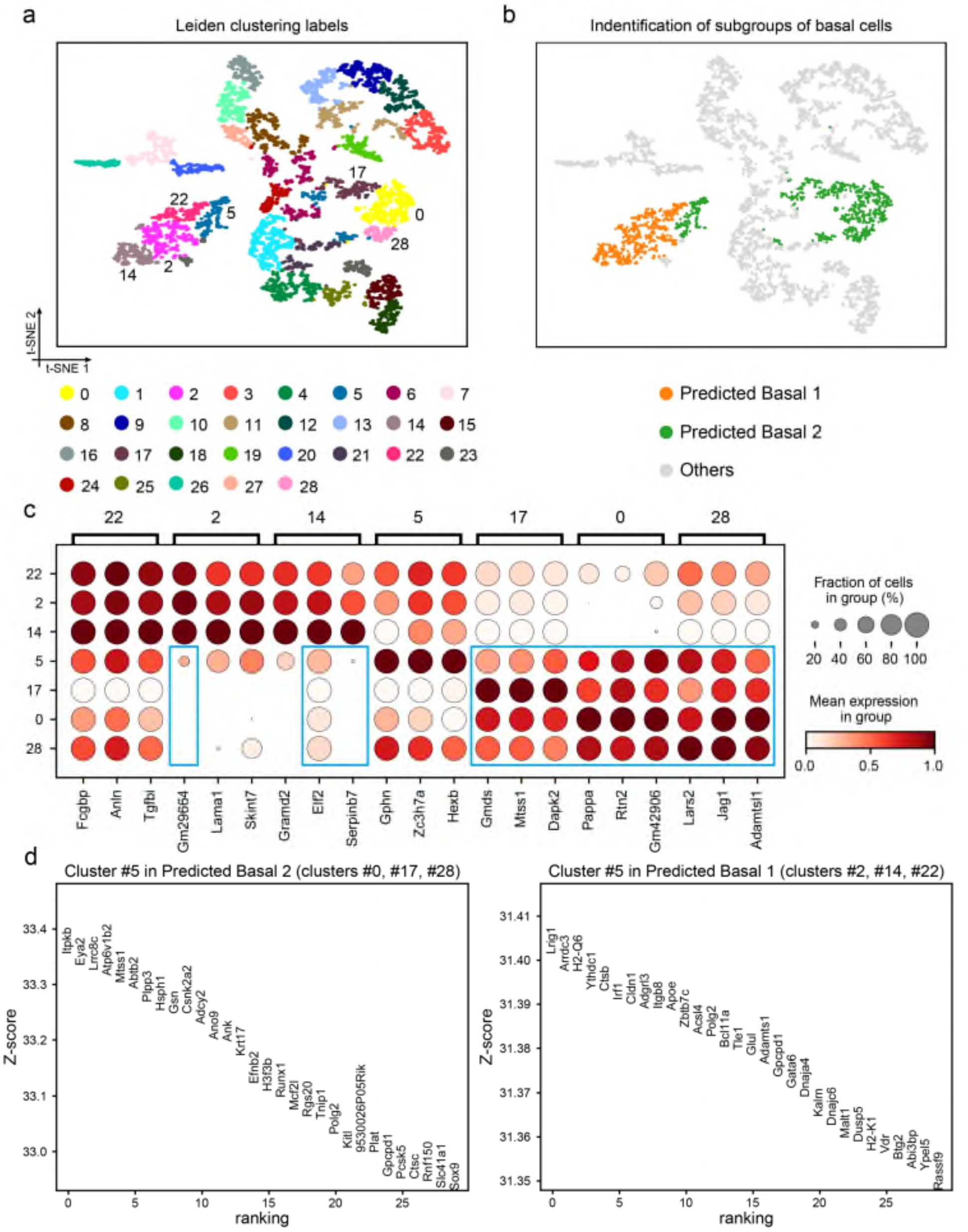

Figure R18 | Visualization and expression patterns of the translated profiles of basal cells. a, **b**, t-SNE visualizations of cells in the first test fold of cross-validation by batch on the MDS dataset, using the scButterfly-C translated profiles, colored by Leiden cluster labels (**a**) and two identified subgroups of basal cells (**b**). **c**, Cell-protein dotplots of the scButterfly-C translated profiles for the cells in the first test fold of cross-validation by batch on the MDS dataset, with the top 3 DEGs selected for each cluster. The size of each dot represents the fraction of cells expressing the genes for each cell type, while the color of each dot represents the average expression level of the genes within the cell type. The average expression values are standardized within the range of 0 to 1. **d**, DEGs between the Predicted Basal 1 and 2, when assigning the cluster #5 in Predicted Basal 2 (left) and Predicted Basal 1 (right), respectively. The DEGs were obtained via one-sided Wilcoxon rank-sum tests.

Comment 2

In line 264, it remains unclear what biological features distinguish these two subgroups. The enriched pathways in both subgroups are related to basal cells, which is expected as they are all basal cells. Are the enriched pathways in two subgroups related to different functions, or different developmental stages of basal cells? Please provide additional information to better explain the distinctions between these two subgroups.

Response to comment 2

Thank you for the meticulous review and this insightful comment. We have confirmed that the enriched pathways in two subgroups are related to different functions, and one of the subgroups may be a subtype of basal cells (BC), namely proliferative basal cells (PBC), which have higher levels of expression of cell cycle-related genes.

Firstly, in the original manuscript, we have provided the pathway enriched by the DEGs between the two subgroups of basal cells. Specifically, we first obtained the ATAC to RNA translated profiles of the cell in the first test fold of cross-validation by batch on the MDS dataset. As mentioned in Response to Comments #1 of Reviewer #4, we separated the basal cells into two subgroups based on the Leiden clustering and t-SNE visualization, namely Predicted Basal 1 and 2, respectively. We identified the top 40 DEGs between the two subgroups with a one-sided Wilcoxon rank-sum test using Scanpy[82]. We performed gene ontology (GO) enrichment[37, 38] analysis for these identified DESs and showed the enriched biological process pathways in Fig. R19a. As the reviewer pointed out, the GO-enriched pathways of the two subgroups are related to basal cells.

Secondly, we observed the DEGs of the translated profiles enriched the biological processes related to the cell cycle. As shown in Fig. R19a, the enrichment results indicated that the DEGs between two subgroups related to the cell cycle process, morphogenesis, and tissue development. We also observed similar findings in the description of the original research about the MDS dataset. As mentioned by [14], although the basal cells formed a single group by the original RNA data, the original ATAC data could identify a subset of actively proliferating basal cells strongly expressing cell cycle-related genes.

Thirdly, previous literature mentioned that one of the subgroups may be a subtype of basal cells (BC), namely proliferative basal cells (PBC), which usually highly express the genes associated with the positive regulation of the cell cycle[94]. To further determine whether the identified subgroups of cells are related to PBC, we evaluated the average expression of the markers for PBC and normal BC in the Predicted Basal 1 and 2. As introduced by previous studies[94, 95], the *Krt14* gene is highly expressed in normal BC while lowly expressed in PBC. Contrarily, for PBC, the *Mki67* and *Top2a* genes are expressed at higher levels compared to normal BC. In the scButterfly-C translated profiles (Fig. R19b), we observed that the PBC markers *Mki67* and *Top2a*

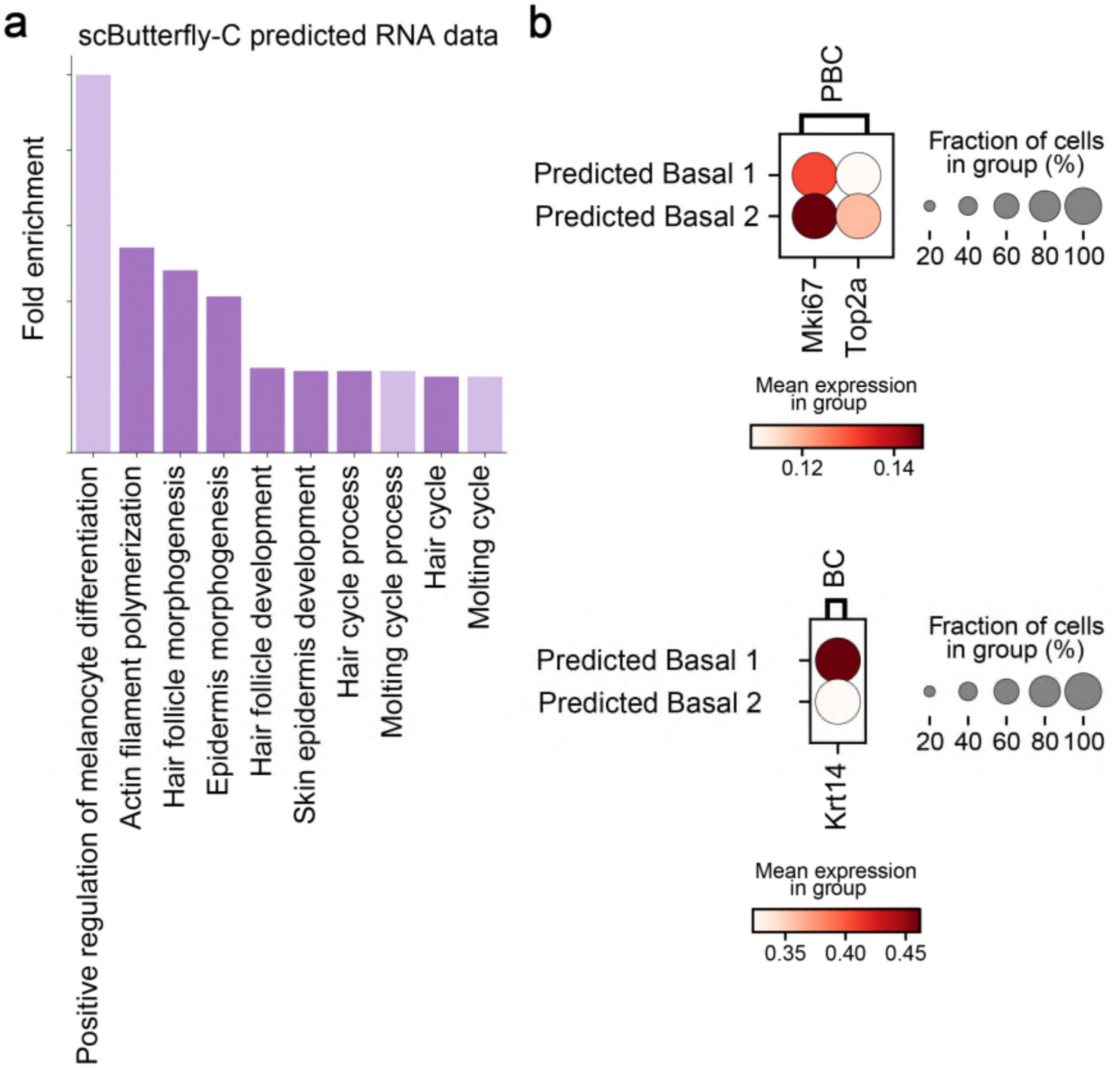

Figure R19 | Expression patterns and biology process terms enriched by the scButterfly-C translated profiles. a, Biology process terms enriched by the top 40 differentially expressed genes (DEGs) between the two subgroups identified based on the scButterfly-C translated RNA profiles, respectively. The height of each bar denotes the fold enrichment and the color indicates the correlation between the item and basal cells according to the literature (dark colors indicate high correlation, light colors indicate relatively weak correlation). **b**, Average expression of the PBC and BC markers of identified two subgroups of basal cells in the scButterfly-C translated profiles.

were highly expressed in Predicted Basal 2, while the BC marker *Krt14* was highly expressed in Predicted Basal 1. Previous study has also indicated that PBC exhibits significant differences from BC in the regulation of the cell cycle[94], which aligns with our enriched pathways identified through GO enrichment.

Overall, we believe that the Predicted Basal 1 and 2 may be related to the subset of non-proliferative basal cells and proliferative basal cells respectively. This result also demonstrated that scButterfly holds great potential in the identification of cell subtypes, as well as in providing functional insights into the identified cell subpopulations. We greatly appreciate the reviewer's suggestion to help us improve the manuscript. We have added the above discussions into Section "scButterfly effectively translates data of novel contexts and reveals biological insights".

COMMENT 3

Additionally, the author should color the tSNE by batches in Fig3d. The two subgroups may potentially be attributed to different batch effects rather than biological variation.

RESPONSE TO COMMENT 3

We appreciate the insightful comment from the reviewer and apologize for the confusion in the original manuscript. The t-SNE visualization in Fig. 3d is based on the

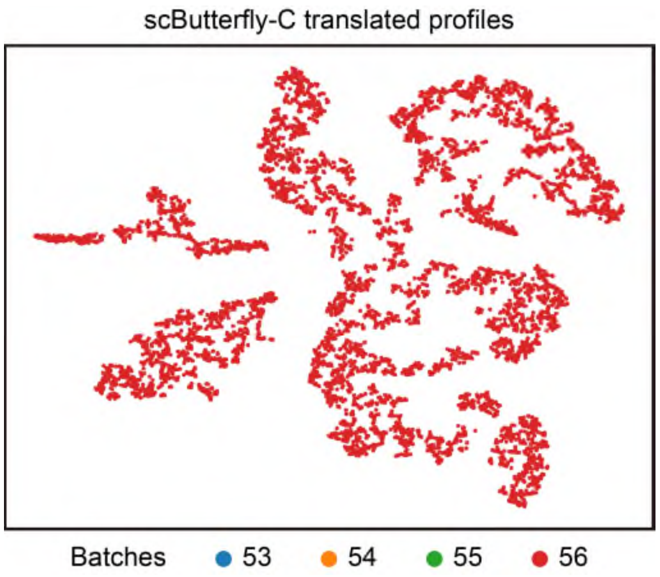

Figure R20 | Visualization of the translated profiles of basal cells. t-SNE visualization of cells in the first test fold of cross-validation by batch on the MDS dataset, using the scButterfly-C translated profiles, colored by batches.

cells in the first test fold of the four-fold cross-validation by batch on the MDS dataset, which means only one independent batch was used for testing and visualization. As shown in Fig. R20, all cells were from the same batch named "56". In addition, as we described in Response to Comments #1 and #2 of Reviewer #4, we identified that the Predicted Basal 1 and 2 may be separately related to the subset of proliferative basal cells and non-proliferative basal cells, further supporting that the two subgroups were not simply attributed to the batch effects. Furthermore, in the original study of the MDS dataset[14], the original ATAC data could also be used to identify a subset of actively proliferating basal cells strongly expressing cell cycle-related genes. Finally, we would like to thank the reviewer for their valuable suggestions again. We have added more details about this experiment in Section "scButterfly effectively translates data of novel contexts and reveals biological insights" to avoid any confusion.

COMMENT 4

When evaluating batch effects removal on the multi-batches dataset, the author should also report numeric evaluation metrics such as k-nearest-neighbor batch-effect test (kBET) and Graph Connectivity (GC).

RESPONSE TO COMMENT 4

We appreciate the valuable comments from the reviewer. We would like to first clarify that the experiments of the cross-validation by batch were not conducted to evaluate the model's ability of batch effects removal, but rather to evaluate the translation performance under a more general scenario. Specifically, for each dataset (BMMC, CL, and MDS), we divided it into four folds based on the batch labels. The BMMC dataset contains the samples from 10 donors from 4 sites, while the CL and MDS datasets separately include 4 batches (Supplementary Fig. 2). For each fold of cross-validation, we performed translation on the data from one batch (MDS and CL datasets) or site (BMMC dataset) while training the model on the remaining batches or sites to ensure that the training and test datasets included completely different batches or sites. In other words, through this cross-validation by batch, data from the same batch or site will not appear in both the training and testing sets simultaneously. Thus, we can evaluate the models' performance in a more general scenario with the cells from new batches to be translated. As described in Section "scButterfly effectively translates data of novel contexts and reveals biological insights", scButterfly outperformed other baseline

methods, indicating the advantages of scButterfly in this general cross-modality translation scenario. However, since all tested data came from the same batch or site, the results cannot demonstrate scButterfly's ability to remove batch effects. In brief, our goal of performing cross-validation by batch is to evaluate the translation performance in a challenging scenario rather than attempting to demonstrate the ability to correct batch effects of scButterfly.

Batch-effect correction is a widely studied problem, including the scenarios for both single-modality[6, 96] and multi-omics[5]. The task of batch correction involves removing the unwanted technical variation while conserving biological variance. However, simultaneously accurately translating between different modalities and removing the batch variation is a more challenging task. We hope the reviewer can agree that this task is beyond the scope of our study.

We would like to express our gratitude once again for the constructive comment from the reviewer. In our revised manuscript, we have included additional experimental details about the cross-validation by batch to avoid confusion.

COMMENT 5

For the translation on paired datasets, the author should also report correlations for RNA and ATAC data.

RESPONSE TO COMMENT 5

We greatly appreciate the insightful suggestions. In this revision, we have additionally evaluated the translated data by various correlation metrics. For the RNA data, we computed the average per-cell Pearson correlation coefficients and the average per-cell Spearman correlation coefficients, which are widely recognized indicators used to assess the performance of cross-modality translation in previous studies[45, 47]. To be specific, for each cell, we separately calculated the Pearson correlation coefficient and Spearman correlation coefficient between the original profiles and the translated profiles. We utilized the average of the correlation coefficients of all cells as the metric to evaluate the translation performance of the scButterfly and other baseline methods. However, the correlation coefficients appear to be sensitive to high noise and sparsity data[29]. Moreover, they are non-robust to outliers[31] and inaccurate for high-dimensional data[32]. For the ATAC data, given its binary feature and extreme sparsity, we serve the

translation task as the binary classification problem. We considered utilizing the auROC (the area under the ROC curve) as a measure of translation performance, which was also widely mentioned in previous studies about cross-modality translation[45, 46]. We calculated the auROC of the translated profiles for each cell and took the average of auROCs of all cells as the metrics. Additionally, deriving from the auPRC (area under the precision-recall curve), we have further considered auPRCnorm for each cell and served the average auPRCnorm of each cell as the metric to evaluate models' performance on ATAC data, as suggested in recent research[46]. auPRCnorm is normalized to correct for the skew in auPRC measurement since ATAC data is usually extremely sparse. The auPRCnorm for each cell could be calculated as follows:

$$auPRCnorm = \frac{(AUPR - PP)}{(1 - PP)}, PP = \frac{\#(peaks\ expressed\ for\ cell)}{\#peaks}$$

We performed five-fold cross-validation on the seven paired datasets (BMMC, MB, CL, MCC, MK, MDS, and PBMC) and evaluated the cross-modality translation performance based on the aforementioned four metrics. As shown in Fig. R21, scButterfly outperformed other baseline methods in terms of Spearman correlation coefficients and auROC. For the Pearson correlation coefficients and auPRCnorm,

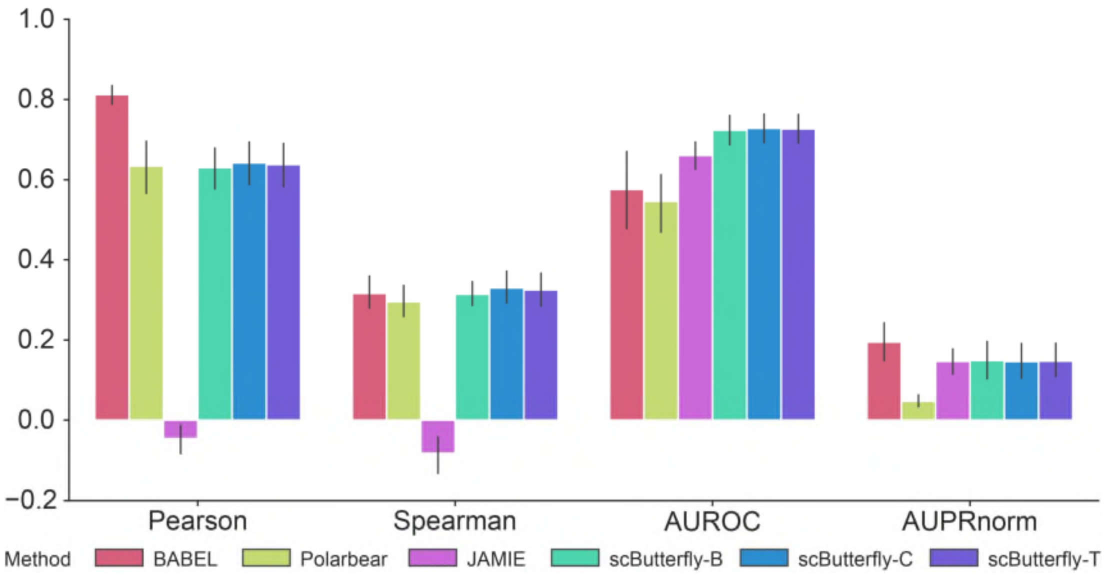

Figure R21 | Cross-modality translation performance evaluated by correlation. Quantitative evaluation of the translated profiles for numerical accuracy in five-fold cross-validation by cell on the BMMC, MB, CL, MCC, MK, MDS and PBMC datasets, via Pearson and Spearman correlation for RNA data and AUROC and AUPRnorm for ATAC data. The height of each bar denotes the median value of each metric and the error bars show 95% confidence interval. Note that the bars of JAMIE only contain 30 data points from six datasets since JAMIE encountered GPU memory errors on the BMMC dataset.

although BABEL also performed comparably, scButterfly showcased competitive performance compared to other baseline methods. Overall, we identified that scButterfly also demonstrates promising translation performance in terms of numerical accuracy. In the revised manuscript, we have added the above description in Section “scButterfly enables cross-modality translation while preserving cell heterogeneity”.

COMMENT 6

Line 224, Fig.2b should be Fig.3b.

RESPONSE TO COMMENT 6

We appreciate the reviewer's careful reading of our manuscript and apologize for the confusion caused by the original description. Our aim was to demonstrate that the translation performance of all methods in the cross-validation by cell types (Fig. 3a) significantly decreases compared to that of cross-validation by cell (Fig. 2b), indicating that translating on unseen cell types is a more challenging scenario. We have refined this description in the revised manuscript in Lines 247-249 to avoid confusion.

COMMENT 7

In Fig. 2a, 3c, it’s hard to find cell types mentioned in the main text. Please highlight.

RESPONSE TO COMMENT 7

We apologize for not labelling the mentioned cell types in Figs. 2a and 3c. We appreciate the reviewer's suggestion and have added colored boxes to indicate the cell types mentioned in the main text. Additionally, we have double-checked other figures to make the manuscript more reader-friendly.

COMMENT 8

Since the ADT data dimension is low, it would be beneficial to visualize the translated ADT data pattern (such as heatmap) and compare it with the true data pattern.

RESPONSE TO COMMENT 8

We greatly appreciate the valuable comment from the reviewer. Strictly following the reviewer's suggestion, we provided additional heatmaps for the original ADT data and translated ADT data. Given the low dimensionality of the ADT data in the CITE_BM dataset (25 surface proteins), we took it as an example to visualize the patterns in the translated profiles of scButterfly and compared it with the patterns in the original data. We presented the heatmap of the original profiles and scButterfly-B (-C, -T) translated profiles of the cells in the first fold of the five-fold cross-validation in Fig. R22. It could be observed that scButterfly accurately captured most of the patterns presented in the original data and effectively reduced the noise. Additionally, we noticed that scButterfly enhanced the expression of CD38 in Plasmablasts compared to the original data (marked with red boxes), where CD38 is a widely recognized marker for Plasmablasts[97]. This result suggested that scButterfly may hold the potential to enhance data.

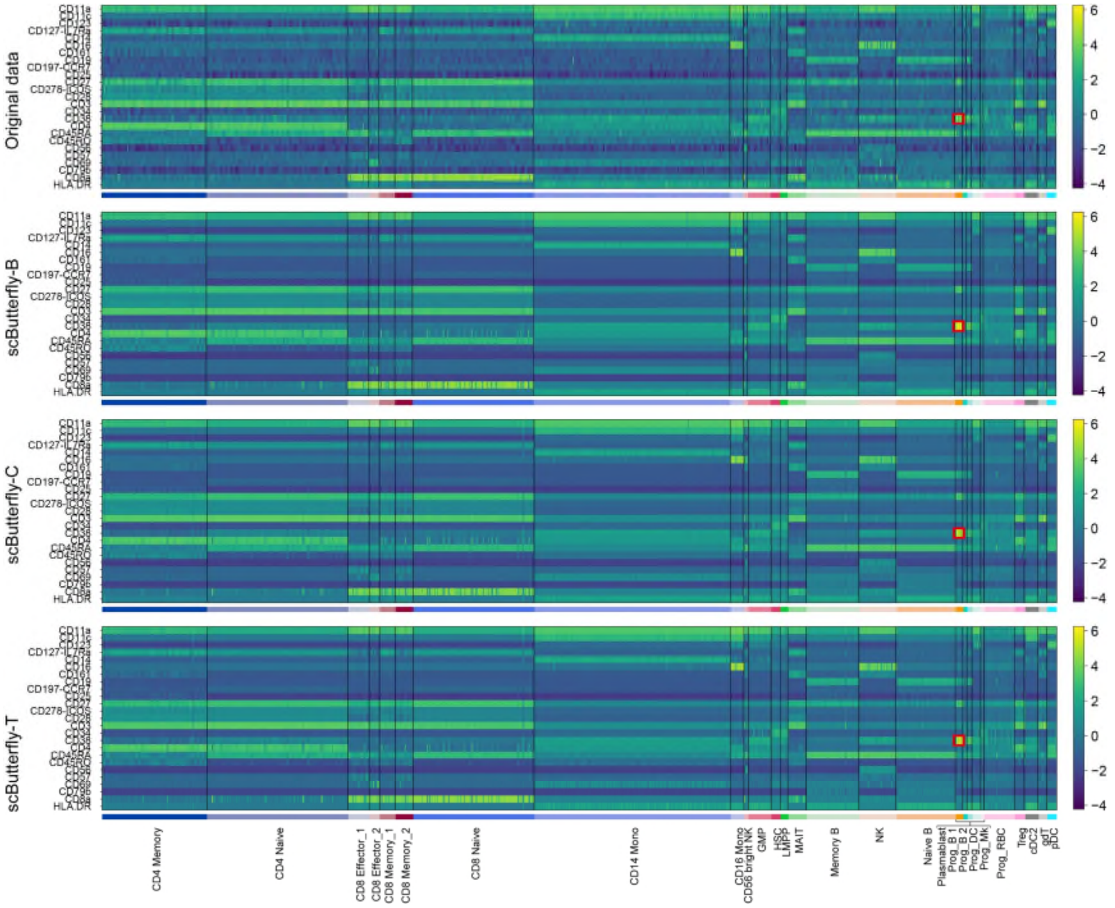

Figure R22 | Cell-protein heatmaps of the origianl data and the translation from scButterfly. Cell-protein heatmaps of the original data and the translation from scButterfly, for the cells in the first fold of five-fold cross-validation by cell on the CITE_BM dataset.

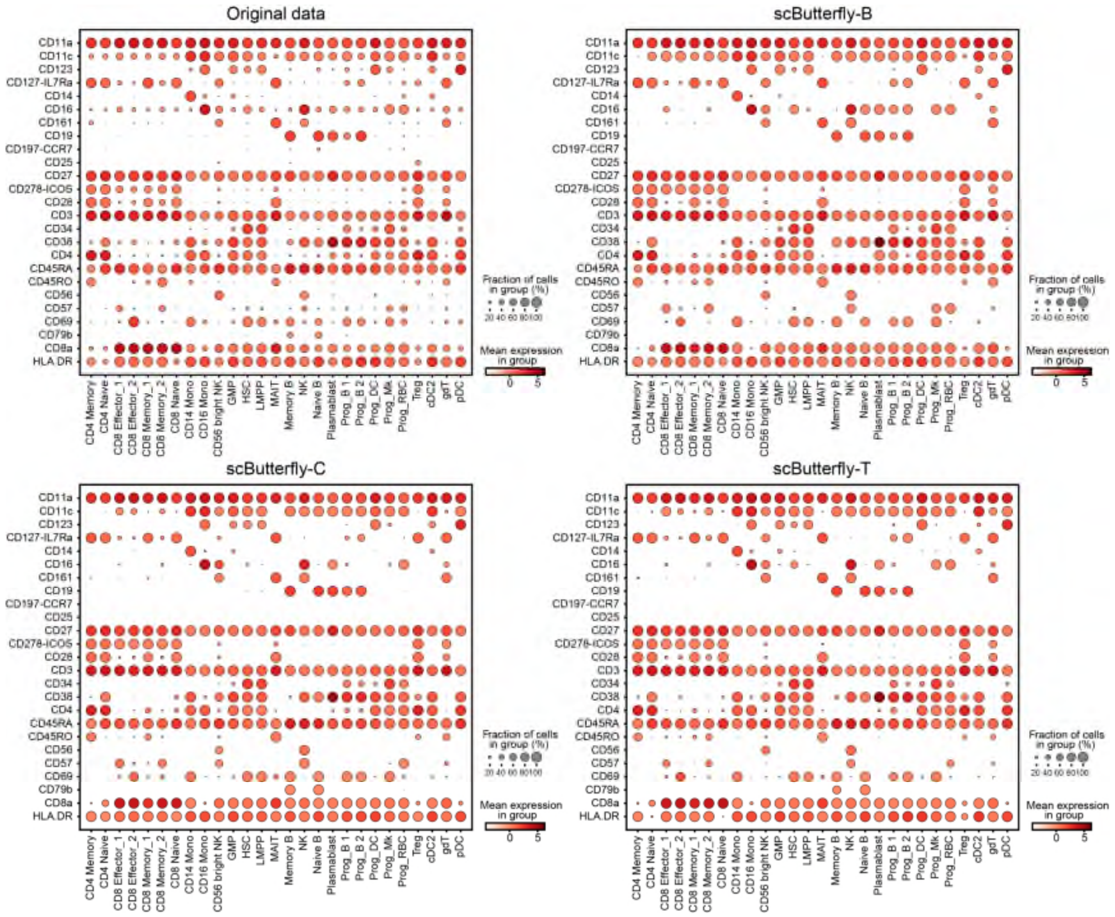

Figure R23 | Cell-protein dotplots of the origianl data and the translation from scButterfly. Cell-protein dotplots of the original data and the translation from scButterfly, for the cells in the first fold of five-fold cross-validation by cell on the CITE_BM dataset. The size of each dot represents the fraction of cells expressing the protein for each cell type, while the color of each dot represents the average expression level of the protein within the cell type.

To better investigate the patterns in the translated ADT data and original ADT data, we further visualized the cells of this fold with dotplots. For the dotplots in Fig. R23, the size of each dot represents the fraction of cells expressing the protein for each cell type, while the color of each dot represents the average expression level of the protein within the cell type. As shown in Fig. R23, scButterfly accurately captured similar patterns as the original data, demonstrating scButterfly's ability to accurately perform cross-modality translation from RNA to ADT.

In conclusion, we greatly appreciate the reviewer's comment, which helped us further improve the manuscript. We have added the visualizations above in the Supplementary Fig. 18.

Reference

1. Van Der Maaten, L. & Hinton, G. Visualizing data using t-SNE. *J. Mach. Learn. Res.* **9**, 2579-2625 (2008).
2. Traag, V.A., Waltman, L. & van Eck, N.J. From Louvain to Leiden: guaranteeing well-connected communities. *Sci. Rep.* **9** (2019).
3. Ashuach, T. et al. MultiVI: deep generative model for the integration of multimodal data. *Nat. Methods* **20**, 1222-1231 (2023).
4. Lütge, A. et al. CellMixS: quantifying and visualizing batch effects in single-cell RNA-seq data. *Life Sci Alliance* **4** (2021).
5. Luecken, M.D. et al. Benchmarking atlas-level data integration in single-cell genomics. *Nat. Methods* **19**, 41-50 (2022).
6. Tran, H.T.N. et al. A benchmark of batch-effect correction methods for single-cell RNA sequencing data. *Genome Biol.* **21**, 12 (2020).
7. Lotfollahi, M., Wolf, F.A. & Theis, F.J. scGen predicts single-cell perturbation responses. *Nature Methods* **16**, 715-721 (2019).
8. Behdenna, A. et al. pyComBat, a Python tool for batch effects correction in high-throughput molecular data using empirical Bayes methods. *BMC Bioinformatics* **24**, 459 (2023).
9. Fleming, S.J. et al. Unsupervised removal of systematic background noise from droplet-based single-cell experiments using CellBender. *Nat. Methods* **20**, 1323-1335 (2023).
10. Stoeckius, M. et al. Simultaneous epitope and transcriptome measurement in single cells. *Nature Methods* **14**, 865-868 (2017).
11. Dixit, A. et al. Perturb-Seq: Dissecting Molecular Circuits with Scalable Single-Cell RNA Profiling of Pooled Genetic Screens. *Cell* **167**, 1853-1866.e1817 (2016).
12. Liu, L. et al. Deconvolution of single-cell multi-omics layers reveals regulatory heterogeneity. *Nat. Commun.* **10** (2019).
13. Chen, S., Lake, B.B. & Zhang, K. High-throughput sequencing of the transcriptome and chromatin accessibility in the same cell. *Nat. Biotechnol.* **37**, 1452-1457 (2019).
14. Ma, S. et al. Chromatin Potential Identified by Shared Single-Cell Profiling of RNA and Chromatin. *Cell* **183**, 1103-1116.e1120 (2020).
15. Mulè, M.P., Martins, A.J. & Tsang, J.S. Normalizing and denoising protein expression data from droplet-based single cell profiling. *Nat. Commun.* **13**, 2099 (2022).
16. Govek, K.W. et al. Single-cell transcriptomic analysis of mIHC images via antigen mapping. *Sci. Adv.* **7** (2021).
17. Trong, T.N. et al. Semisupervised Generative Autoencoder for Single-Cell Data. *J. Comput. Biol.* **27**, 1190-1203 (2020).
18. Gayoso, A. et al. Joint probabilistic modeling of single-cell multi-omic data with totalVI. *Nature Methods* **18**, 272-282 (2021).

19. Gu, W., Tandon, A., Ahn, Y.Y. & Radicchi, F. Principled approach to the selection of the embedding dimension of networks. *Nat. Commun.* **12**, 3772 (2021).
20. Lucas, J., Tucker, G., Grosse, R. & Norouzi, M. in Proceedings of the 33rd International Conference on Neural Information Processing Systems Article 844 (Curran Associates Inc., 2019).
21. Persad, S. et al. SEACells infers transcriptional and epigenomic cellular states from single-cell genomics data. *Nat. Biotechnol.* **41**, 1746-1757 (2023).
22. Fang, R. et al. Comprehensive analysis of single cell ATAC-seq data with SnapATAC. *Nat. Commun.* **12**, 1337 (2021).
23. Zhang, W., Jiang, R., Chen, S. & Wang, Y. scIBD: a self-supervised iterative-optimizing model for boosting the detection of heterotypic doublets in single-cell chromatin accessibility data. *Genome Biol.* **24**, 225 (2023).
24. Chen, H. et al. Assessment of computational methods for the analysis of single-cell ATAC-seq data. *Genome Biol.* **20**, 241 (2019).
25. Shengquan, C., Boheng, Z., Xiaoyang, C., Xuegong, Z. & Rui, J. stPlus: a reference-based method for the accurate enhancement of spatial transcriptomics. *Bioinformatics* **37**, i299-i307 (2021).
26. Yu, X., Xu, X., Zhang, J. & Li, X. Batch alignment of single-cell transcriptomics data using deep metric learning. *Nat. Commun.* **14**, 960 (2023).
27. Chen, S. et al. RA3 is a reference-guided approach for epigenetic characterization of single cells. *Nat. Commun.* **12**, 2177 (2021).
28. Li, D., Ding, J. & Bar-Joseph, Z. Unsupervised cell functional annotation for single-cell RNA-seq. *Genome Res.* **32**, 1765-1775 (2022).
29. Baak, M., Koopman, R., Snoek, H. & Klous, S. A new correlation coefficient between categorical, ordinal and interval variables with Pearson characteristics. *Computational Statistics & Data Analysis* **152**, 107043 (2020).
30. Lähnemann, D. et al. Eleven grand challenges in single-cell data science. *Genome Biol.* **21**, 31 (2020).
31. Niven, E.B. & Deutsch, C.V. Calculating a robust correlation coefficient and quantifying its uncertainty. *Computers & Geosciences* **40**, 1-9 (2012).
32. Raymaekers, J. & Rousseeuw, P.J. Fast Robust Correlation for High-Dimensional Data. *Technometrics* **63**, 184-198 (2021).
33. Cusanovich, D.A. et al. A Single-Cell Atlas of In Vivo Mammalian Chromatin Accessibility. *Cell* **174**, 1309-1324.e1318 (2018).
34. Zhang, K. et al. A single-cell atlas of chromatin accessibility in the human genome. *Cell* **184**, 5985-6001.e5919 (2021).
35. Jones, R.C. et al. The Tabula Sapiens: A multiple-organ, single-cell transcriptomic atlas of humans. *Science* **376** (2022).
36. Elmentaite, R., Domínguez Conde, C., Yang, L. & Teichmann, S.A. Single-cell atlases: shared and tissue-specific cell types across human organs. *Nat. Rev. Genet.* **23**, 395-410 (2022).
37. Ashburner, M. et al. Gene ontology: tool for the unification of biology. The Gene Ontology Consortium. *Nat. Genet.* **25**, 25-29 (2000).

38. Gene Ontology, C. The Gene Ontology resource: enriching a GOld mine. *Nucleic Acids Res.* **49**, D325-D334 (2021).
39. Slowikowski, K., Hu, X. & Raychaudhuri, S. SNPsea: an algorithm to identify cell types, tissues and pathways affected by risk loci. *Bioinformatics* **30**, 2496-2497 (2014).
40. Li, H. et al. Inferring transcription factor regulatory networks from single-cell ATAC-seq data based on graph neural networks. *Nat. Mach. Intell.* **4**, 389-400 (2022).
41. Danese, A. et al. EpiScanpy: integrated single-cell epigenomic analysis. *Nat. Commun.* **12**, 5228 (2021).
42. He, B. et al. CD8(+) T Cells Utilize Highly Dynamic Enhancer Repertoires and Regulatory Circuitry in Response to Infections. *Immunity* **45**, 1341-1354 (2016).
43. Tran, D.Q., Ramsey, H. & Shevach, E.M. Induction of FOXP3 expression in naive human CD4+FOXP3 T cells by T-cell receptor stimulation is transforming growth factor-beta dependent but does not confer a regulatory phenotype. *Blood* **110**, 2983-2990 (2007).
44. Park, S.Y., Shin, H.M. & Han, T.H. Synergistic interaction of MEF2D and Sp1 in activation of the CD14 promoter. *Mol. Immunol.* **39**, 25-30 (2002).
45. Wu, K.E., Yost, K.E., Chang, H.Y. & Zou, J. BABEL enables cross-modality translation between multiomic profiles at single-cell resolution. *Proc. Natl. Acad. Sci. U. S. A.* **118** (2021).
46. Zhang, R., Meng-Papaxanthos, L., Vert, J.-P. & Noble, W.S. in Research in Computational Molecular Biology. (ed. I. Pe'er) 20-35 (Springer International Publishing, Cham; 2022).
47. Cohen Kalafut, N., Huang, X. & Wang, D. Joint variational autoencoders for multimodal imputation and embedding.
48. G. D, Y., Nair, N.G., Satpathy, P. & Christopher, J. Covariate Shift: A Review and Analysis on Classifiers.
49. Biswas, A. & Mukherjee, S. in Proceedings of the 2021 AAAI/ACM Conference on AI, Ethics, and Society 414–424 (Association for Computing Machinery, Virtual Event, USA; 2021).
50. Lu, J. et al. Learning under Concept Drift: A Review.
51. Heumos, L. et al. Best practices for single-cell analysis across modalities. *Nat Rev Genet* **24**, 550-572 (2023).
52. Heryanto, Y.D., Zhang, Y.Z. & Imoto, S. Predicting cell types with supervised contrastive learning on cells and their types. *Sci. Rep.* **14**, 430 (2024).
53. Wang, S. et al. Leveraging the Cell Ontology to classify unseen cell types. *Nat. Commun.* **12**, 5556 (2021).
54. Xiong, Y.X., Wang, M.G., Chen, L. & Zhang, X.F. Cell-type annotation with accurate unseen cell-type identification using multiple references. *PLoS Comput. Biol.* **19**, e1011261 (2023).
55. Zhang, Q. et al. Integrative analysis of multi-omics data to detect the underlying molecular mechanisms for obesity in vivo in humans. *Hum. Genomics* **16**, 15 (2022).

56. Subramanian, I., Verma, S., Kumar, S., Jere, A. & Anamika, K. Multi-omics Data Integration, Interpretation, and Its Application. *Bioinform Biol Insights* **14**, 1177932219899051 (2020).
57. Sun, Y.V. & Hu, Y.J. Integrative Analysis of Multi-omics Data for Discovery and Functional Studies of Complex Human Diseases. *Adv. Genet.* **93**, 147-190 (2016).
58. Abdelaal, T., Mourragui, S., Mahfouz, A. & Reinders, M.J.T. SpaGE: Spatial Gene Enhancement using scRNA-seq. *Nucleic Acids Res.* **48**, e107 (2020).
59. Liu, Y. et al. IsoCell: An Approach to Enhance Single Cell Clustering by Integrating Isoform-Level Expression Through Orthogonal Projection. *IEEE/ACM Trans. Comput. Biol. Bioinform.* **20**, 465-475 (2023).
60. Lakkis, J. et al. A multi-use deep learning method for CITE-seq and single-cell RNA-seq data integration with cell surface protein prediction and imputation. *Nat. Mach. Intell.* **4**, 940-952 (2022).
61. Chen, X. et al. Cell type annotation of single-cell chromatin accessibility data via supervised Bayesian embedding. *Nat. Mach. Intell.* **4**, 116-126 (2022).
62. Ma, W., Lu, J. & Wu, H. Cellcano: supervised cell type identification for single cell ATAC-seq data. *Nat. Commun.* **14**, 1864 (2023).
63. Buenrostro, J.D. et al. Single-cell chromatin accessibility reveals principles of regulatory variation. *Nature* **523**, 486-490 (2015).
64. Cusanovich, D.A. et al. Multiplex single-cell profiling of chromatin accessibility by combinatorial cellular indexing. *Science* **348**, 910-914 (2015).
65. Buenrostro, J.D. et al. Integrated Single-Cell Analysis Maps the Continuous Regulatory Landscape of Human Hematopoietic Differentiation. *Cell* **173**, 1535-1548.e1516 (2018).
66. Preissl, S. et al. Single-nucleus analysis of accessible chromatin in developing mouse forebrain reveals cell-type-specific transcriptional regulation. *Nat. Neurosci.* **21**, 432-439 (2018).
67. Pijuan-Sala, B. et al. Single-cell chromatin accessibility maps reveal regulatory programs driving early mouse organogenesis. *Nat. Cell Biol.* **22**, 487-497 (2020).
68. Rai, V. et al. Single-cell ATAC-Seq in human pancreatic islets and deep learning upscaling of rare cells reveals cell-specific type 2 diabetes regulatory signatures. *Mol Metab* **32**, 109-121 (2020).
69. Cheng, C., Chen, W., Jin, H. & Chen, X. A Review of Single-Cell RNA-Seq Annotation, Integration, and Cell-Cell Communication. *Cells* **12** (2023).
70. Abdelaal, T. et al. A comparison of automatic cell identification methods for single-cell RNA sequencing data. *Genome Biol.* **20**, 194 (2019).
71. Pasquini, G., Rojo Arias, J.E., Schäfer, P. & Busskamp, V. Automated methods for cell type annotation on scRNA-seq data. *Comput Struct Biotechnol J* **19**, 961-969 (2021).
72. Salimans, T. et al. in Proceedings of the 30th International Conference on Neural Information Processing Systems 2234–2242 (Curran Associates Inc., Barcelona, Spain; 2016).

73. Tu, X., Cao, Z.-J., chenrui, x., Mostafavi, S. & Gao, G., Vol. 35. (eds. S. Koyejo et al.) 15942-15955 (Curran Associates, Inc.
74. Xiong, L. et al. Online single-cell data integration through projecting heterogeneous datasets into a common cell-embedding space. *Nat. Commun.* **13**, 6118 (2022).
75. Cao, Z.J. & Gao, G. Multi-omics single-cell data integration and regulatory inference with graph-linked embedding. *Nat. Biotechnol.* **40**, 1458-1466 (2022).
76. Lopez, R., Regier, J., Cole, M.B., Jordan, M.I. & Yosef, N. Deep generative modeling for single-cell transcriptomics. *Nat. Methods* **15**, 1053-1058 (2018).
77. Luecken, M.D. et al. in Thirty-fifth Conference on Neural Information Processing Systems (2021).
78. Krassowski, M., Das, V., Sahu, S.K. & Misra, B.B. State of the Field in Multi-Omics Research: From Computational Needs to Data Mining and Sharing. *Front Genet* **11**, 610798 (2020).
79. Cantini, L. et al. Benchmarking joint multi-omics dimensionality reduction approaches for the study of cancer. *Nat. Commun.* **12**, 124 (2021).
80. Lee, J., Hyeon, D.Y. & Hwang, D. Single-cell multiomics: technologies and data analysis methods. *Exp Mol Med* **52**, 1428-1442 (2020).
81. Stuart, T. et al. Comprehensive Integration of Single-Cell Data. *Cell* **177**, 1888-1902.e1821 (2019).
82. Wolf, F.A., Angerer, P. & Theis, F.J. SCANPY: Large-scale single-cell gene expression data analysis. *Genome Biol.* **19** (2018).
83. Chen, S., Wang, R., Long, W. & Jiang, R. ASTER: accurately estimating the number of cell types in single-cell chromatin accessibility data. *Bioinformatics* **39** (2023).
84. Yang, K.D. et al. Multi-domain translation between single-cell imaging and sequencing data using autoencoders. *Nat. Commun.* **12** (2021).
85. Tang, X. et al. Explainable multi-task learning for multi-modality biological data analysis. *Nat. Commun.* **14**, 2546 (2023).
86. Dekker, J. & Misteli, T. Long-Range Chromatin Interactions. *Cold Spring Harb. Perspect. Biol.* **7**, a019356 (2015).
87. Cao, J. et al. Joint profiling of chromatin accessibility and gene expression in thousands of single cells. *Science* **361**, 1380-1385 (2018).
88. Bredikhin, D., Kats, I. & Stegle, O. MUON: multimodal omics analysis framework. *Genome Biol.* **23**, 42 (2022).
89. Muto, Y. et al. Single cell transcriptional and chromatin accessibility profiling redefine cellular heterogeneity in the adult human kidney. *Nat. Commun.* **12** (2021).
90. Yao, Z. et al. A transcriptomic and epigenomic cell atlas of the mouse primary motor cortex. *Nature* **598**, 103-110 (2021).
91. Cao, J. et al. A human cell atlas of fetal gene expression. *Science* **370** (2020).
92. Domcke, S. et al. A human cell atlas of fetal chromatin accessibility. *Science* **370** (2020).

93. Abadi, M. et al. in Proceedings of the 12th USENIX conference on Operating Systems Design and Implementation 265–283 (USENIX Association, Savannah, GA, USA; 2016).
94. Huang, M. et al. Cux1(+) proliferative basal cells promote epidermal hyperplasia in chronic dry skin disease identified by single-cell RNA transcriptomics. *J Pharm Anal* **13**, 745-759 (2023).
95. Haensel, D. et al. Defining Epidermal Basal Cell States during Skin Homeostasis and Wound Healing Using Single-Cell Transcriptomics. *Cell Rep* **30**, 3932-3947.e3936 (2020).
96. Büttner, M., Miao, Z., Wolf, F.A., Teichmann, S.A. & Theis, F.J. A test metric for assessing single-cell RNA-seq batch correction. *Nat. Methods* **16**, 43-49 (2019).
97. Fink, K. Origin and Function of Circulating Plasmablasts during Acute Viral Infections. *Front Immunol* **3**, 78 (2012).

Reviewer #2 (Remarks to the Author):

The authors have addressed most of my concerns. Here are two further comments:
1) I appreciate the authors' efforts to build the additional cell augmentation methods. For the new methods, I still have some questions: 1) For scButterfly-C, how do the authors select two clusters, from mRNA and ATAC, respectively, to build cell pairs? 2) Since many cell types are still mixed on the t-SNE plots, can the authors compare the randomly paired cells to the experimentally paired cells (such as SNARE-seq data) to see if there are any discrepancies? 3) Can the authors use CCA-based methods, such as the pipeline in Seurat, to detect anchors between the cells with mRNA and ATAC?
2) For the batch correction, I have two more comments: 1) The authors state that "the combination of scGen and pyComBat led to a decrease in translation performance, revealing that the batch correction before data augmentation may not be suitable for unpaired data translation." This result is very interesting since generally the signal will be enhanced after batch correction. The authors should carefully explain why the batch effects can benefit the performance of model; 2) The example data shown by the authors has a low batch effect, but it is not always true for all the datasets. So, I would suggest showing more examples with higher batch effects. It would be simulated datasets with controllable batch effects.

Reviewer #2 (Remarks on code availability):

The authors well provided the instructions to install and run the model.

Reviewer #3 (Remarks to the Author):

Our questions have been answered. We don't have further comments.

Reviewer #4 (Remarks to the Author):

Thank you for addressing my comments. I believe that the revised results and the addition of new figures have resolved my concerns and made the manuscript ready for publication.

Reviewer #4 (Remarks on code availability):

The code is very detailed and well-organized. The author also provided tutorials of the models used in the manuscript.

To the reviewers,

We want to further thank the three reviewers for their affirmation and further constructive comments on our manuscript entitled "scButterfy: a versatile single-cell cross-modality translation method via dual-aligned variational autoencoders". We have thoroughly revised and enhanced our manuscript based on their comments. In addition to the minor changes, we have made the following two major modifications in our revised manuscript, with the modifications highlighted in red. These changes are summarized in Supplementary Texts 1, 11, 12, Supplementary Figs. 22, 33, 34, as well as the Methods section.

1) We developed two variants of scButterfly, namely scButterfly-A and scButterfly-TA, to investigate the influence of data augmentation with the anchors detected by the CCA-based methods (Supplementary Text 12, Supplementary Fig. 34). Besides, we compared the augmented data with the experimental paired data and analysed their discrepancies (Supplementary Text 11, Supplementary Fig. 33).

2) We further evaluated the impact of adding a batch correction step before data augmentation on the simulated data with higher batch effects. (Supplementary Text 1, Supplementary Fig. 22). Additionally, we analyzed the possible reasons for the performance decline associated with incorporating the batch correction step. (Supplementary Text 1).

Next, we will clarify the comments raised by the reviewers point-by-point, with the reviewers' points in black and our responses in blue.

REVIEWER #2

The authors have addressed most of my concerns. Here are two further comments:

RESPONSE

We would like to express our gratitude to the reviewer for the affirmation of our work and the further valuable comments. These comments have played a crucial role in enhancing our manuscript. We have carefully considered and addressed each comment point-by-point.

COMMENT 1

I appreciate the authors' efforts to build the additional cell augmentation methods. For the new methods, I still have some questions: 1) For scButterfly-C, how do the authors select two clusters, from mRNA and ATAC, respectively, to build cell pairs? 2) Since many cell types are still mixed on the t-SNE plots, can the authors compare the randomly paired cells to the experimentally paired cells (such as SNARE-seq data) to see if there are any discrepancies? 3) Can the authors use CCA-based methods, such as the pipeline in Seurat, to detect anchors between the cells with mRNA and ATAC?

RESPONSE TO COMMENT 1

We appreciate the reviewer for these thoughtful comments. **For the first point**, we apologize for any confusion caused by our description. Regarding the three variants of scButterfly with data augmentation strategy (scButterfly-C, scButterfly-T, and scButterfly-TC), we would like to clarify that we paired the profiles of different modalities of the cells from the same cell type or cluster, rather than paired the profiles of the cells from different clusters. Specifically, for scButterfly-C (**C**luster), we first integrated the multi-omics profiles with MultiVI[1] and performed Leiden[2] clustering on the joint cell embeddings. Artificial samples were generated by randomly pairing profiles of different modalities **within each cluster** (e.g., pairing Modality #1 of Cell #1 and Modality #2 of Cell #2, where Cells #1 and #2 are from a same cluster). For scButterfly-T (**T**ype), we directly paired the profiles of different modalities of the cells **for each cell type** to generate artificial samples. For scButterfly-TC (**T**ype **C**luster), we integrated the multi-omics profiles for each cell type using MultiVI and obtained refined clusters with Leiden. We generated additional artificial samples by randomly

pairing profiles of different modalities of the cells **from each cluster in each cell type**, aiming to reduce the additional noise introduced in data augmentation.

For the second point, strictly following the reviewer's suggestion, we compared the differences between the artificial samples generated by randomly pairing and experimentally paired original data. Illustrating with the SNARE-seq[3] profiled MCC dataset as an example, we obtained the joint cell embeddings of augmented and experimental multi-omics profiles via MultiVI and visualized the embeddings with t-distributed stochastic neighbor embedding[4] (t-SNE). As shown in Fig. R1, the samples generated by randomly pairing (Column #2 in Fig. R1) do not exhibit significant discrepancies compared to the experimentally paired original samples (Column #3 in Fig. R1) in t-SNE visualizations. Furthermore, we observed that the augmented data can better distinguish different cell types in the visualizations compared to the original data, especially for scButterfly-T and scButetrfly-TC. For example, as shown in Fig. R1, the augmented dataset could distinguish the InN, InP, InS, and InV (marked with red boxes), while the original dataset mixed them in the visualizations. Additionally, we found that there are difference between the dataset augmented with different

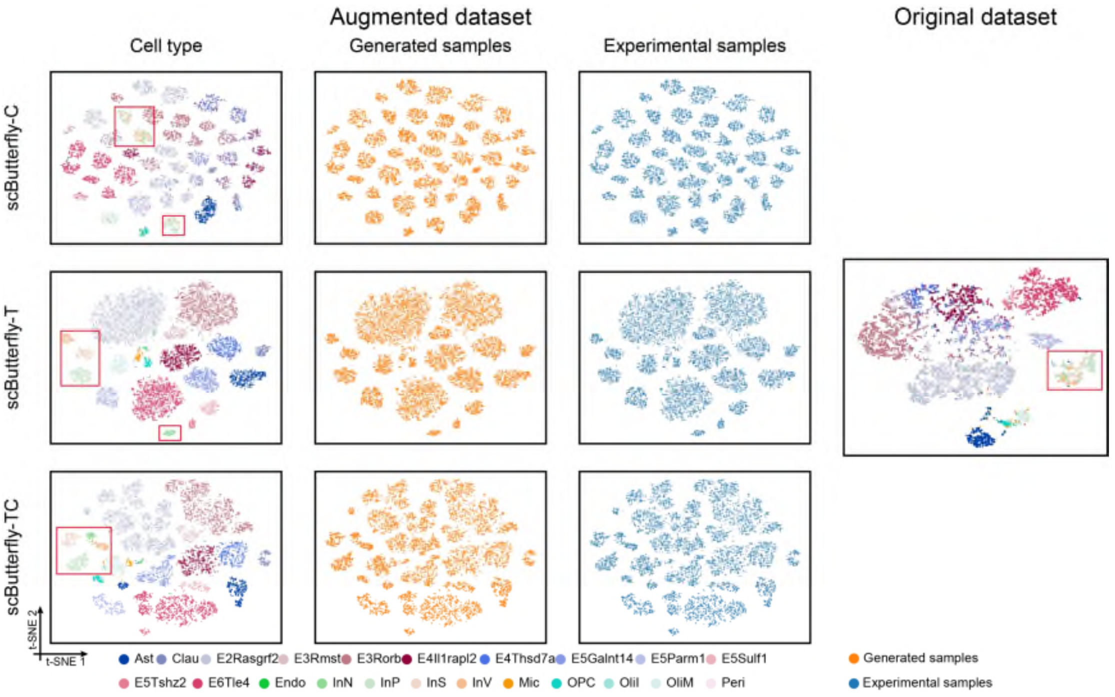

Fig. R1 | Visualization of cells in the original dataset and the augmented datasets. t-SNE visualizations of cells in the first training fold of five-fold cross-validation on the MCC dataset, using the original dataset and the datasets augmented by scButterfly-C, scButterfly-T, and scButterfly-TC, respectively. In the Columns #2 and #3, the generated artificial samples and the experimental original samples are colored with yellow and blue, respectively. Note that the cell types of cells generated by scButterfly-C are labeled as consistent with the cell types of RNA data in the pairs, since scButterfly-C may pair the profiles of the cells of different cell types.

strategies. Specifically, scButterfly-C augments data by randomly pairing the profiles of different modalities within the same cluster, resulting in the samples seeming to be tightly distributed into many clusters in the visualizations. scButterfly-T augments data by randomly pairing the profiles of different modalities of the same cell type, causing the samples of the same cell type to cluster together in the visualizations, while the samples of different cell types loosely distribute. scButterfly-TC augments data with the refined clusters within each cell type, leading to some cell types with more cells further distinguished into smaller parts in the visualizations, which aligns with expectations. In conclusion, we observed that the generated samples do not exhibit significant differences from the experimental samples in visualizations and the patterns of the augmented datasets are related to the strategies of data augmentation.

For the third point, we incorporated the canonical correlation analysis (CCA)-based method, namely Seurat[5], to detect anchors between two modalities and utilized them for data augmentation, resulting in a new variant of scButterfly named scButterfly-A (**A**nchor). For scButterfly-A, we first treated the RNA and ATAC training sets as the reference and query datasets, respectively, and obtained the CCA embeddings and the anchor pairs between the two modalities via Seurat. Subsequently, for the RNA profile of each cell, we identified the nearest RNA anchor under the Euclidean distance on the RNA CCA embeddings. We then paired this RNA profile with the ATAC profile of the ATAC anchor associated with the nearest RNA anchor to generate artificial samples. Since a single RNA anchor may exist in multiple anchor pairs with different ATAC anchors, we only selected the anchor pairs with the highest shared neighbor overlap score for data augmentation. After the above steps, we obtained a generated dataset of the same size as the original training set. Similarly, for the ATAC profile of each cell, we found the nearest ATAC anchor and paired it with the RNA profile of the corresponding RNA anchor with the highest score, resulting in a same number of artificial samples as the original training set. By merging these generated samples with the original data, we finally obtained an augmented dataset three times the size of the original data, which was used for model training.

To investigate the influence of the cell type-specific anchors, we further proposed the variant scButterfly-TA (**T**ype **A**nchor). We first performed Seurat for the multi-omics profiles of cells of each cell type to obtain cell type-specific CCA embeddings and anchors. Subsequently, for each cell type, similar to scButterfly-A, we identified the nearest RNA anchor for the RNA profiles of each cell and paired the RNA profiles with the ATAC profile of the highest-scoring ATAC anchor corresponding to the

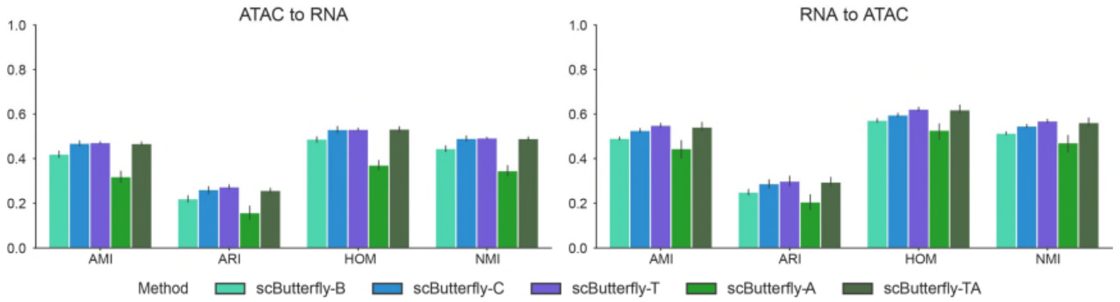

Fig. R2 | Cross-modality translation performance of scButterfly-A and scButterfly-TA. Quantitative evaluation of the translated profiles for preserving cell heterogeneity. We evaluated the cross-validation performance on the MCC datasets via cell clustering with metrics of AMI, ARI, HOM, and NMI. The height of each bar denotes the median value of each metric and the error bars show 95% confidence interval.

nearest RNA anchor. We also paired each ATAC profile with the RNA profile of the highest-scoring RNA anchor related to the nearest ATAC anchor. These artificial samples were mixed with the original training samples, resulting in a training dataset that is three times as large as the original dataset for model training.

To evaluate the effectiveness of these two variants, we conducted five-fold cross-validation on the MCC dataset to compare the performance of scButterfly-A, and scButterfly-TA with the basic scButterfly model (scButterfly-B) and the other two variants (scButterfly-C and scButterfly-T). As shown in Fig. R2, scButterfly-A struggled to achieve satisfactory cross-modality translation performance. The poor performance of scButterfly-A may be attributed to the anchors detected by Seurat not accurately linking the profiles of the same cell type. For example, for the cells in the first test fold, only 13.080% anchor pairs (203/1552) contain the RNA and ATAC anchors with the same cell type. These augmented training data may mislead the model and harm the cross-modality translation performance. This result suggests that directly using the detected anchors for data augmentation may not be suitable. For scButterfly-TA, it did not show a significant advantage compared to scButterfly-T (p-value=0.965) under one-side Wilcoxon signed-rank test. Considering that scButterfly-T is simpler and more user-friendly compared to scButterfly-TA, we still recommend that users perform cross-modality translation with scButterfly-T. We hope the reviewer can agree with us that incorporating the CCA-detected anchors may not lead to significant improvement in the translation performance.

We have added more details about scButterfly-C, the comparison between the generated samples and the experimental samples, and the discussions about data

augmentation with CCA-detected anchors in Section "The data augmentation strategy of scButterfly".

COMMENT 2

For the batch correction, I have two more comments: 1) The authors state that "the combination of scGen and pyComBat led to a decrease in translation performance, revealing that the batch correction before data augmentation may not be suitable for unpaired data translation." This result is very interesting since generally the signal will be enhanced after batch correction. The authors should carefully explain why the batch effects can benefit the performance of model; 2) The example data shown by the authors has a low batch effect, but it is not always true for all the datasets. So, I would suggest showing more examples with higher batch effects. It would be simulated datasets with controllable batch effects.

RESPONSE TO COMMENT 2

We appreciate the reviewer for the constructive comment. **For the first point**, we sincerely apologize for any confusion our description may have caused. We need to clarify that our intention was to represent that performing batch correction before data augmentation may not be suitable for scButterfly, rather than to emphasize batch effects can benefit the performance of the model. We fully agree with the reviewer that batch correction usually enhances the signal in data. However, we also found that existing batch correction methods may have some potential issues that make them less compatible with scButterfly. Specifically, firstly, many widely-used batch correction methods output dimensionality-reduced embeddings to correct batch effects[5-9], but scButterfly cannot directly apply these dimensionality-reduced embeddings for cross-modality translation. This means that scButterfly cannot be easily combined with these advanced batch correction methods to achieve the optimal batch correction performance. Secondly, recent researches have pointed out that batch correction methods need to balance between correcting batch effects and preserving biological heterogeneity[10-12]. Although the batch correction methods effectively reduce the batch effects, they also lead to some loss of bio-conservation. Besides, since the data augmentation strategy already enabled scButterfly to be less affected by batch effects, the improvement brought by adding a batch correction step may not be able to compensate for the impact of biological heterogeneity loss on the translation performance. Thus, it seems that adding a batch correction step led to some decrease in

cross-modality translation performance. Overall, we aimed to highlight batch correction may not be well-suited for training scButterfly, instead of emphasizing batch effects to enhance the model's performance. We believe that more powerful and robust batch correction methods will effectively enhance the performance of cross-modal translation in the future. However, we also hope that the reviewer could agree with us that this point is beyond the scope of our study.

For the second point, we further evaluated the effectiveness of performing batch correction before data augmentation on a simulated dataset, which contained higher batch effects compared to the original dataset. Based on the original CL dataset, we applied the advanced scDesign3[13] method to simulate RNA profiles and generated an artificial RNA dataset containing 3000 cells. The CL dataset consists of 549 cells from 4 different batches, with some batches lacking part of cell types (Columns #1 and #3 in Fig. R3a). We maintained the proportions of different cell types and the variations between cell types during generation, and further amplified the batch effects. For ATAC profiles, although scDesign3 could simulate ATAC profiles with differentially accessible peaks[13], it failed to tackle the original ATAC profiles with 157,358 peaks. Therefore, we performed the newer ATAC simulation method, simCAS[14], to generate the artificial ATAC profiles. Similar to the simulation of RNA, we generated a 3000-cell artificial ATAC dataset with increased batch effects and preserved the proportional discrepancy of cell types and batches. As shown in Columns #2 and #4 in Fig. R3a, the simulated RNA and ATAC profiles significantly magnified the differences between batches compared to the original dataset (Columns #1 and #3 in Fig. R3a). This simulated unpaired dataset enabled us to better assess the impact of incorporating batch correction step before data augmentation, in the scenarios with large batch effects.

We conducted a five-fold cross-validation on this simulated unpaired dataset and evaluated the translation performance of scButterfly-T and scButterfly-T-with-batch-correction. As described in our first revision, the batch correction step was performed by scGen[15] and pyComBat[16], respectively for RNA and ATAC profiles. As shown in Fig. R3b, scButterfly-T-with-batch-correction still performed worse compared to scButterfly-T. We also visualized the translated profiles from scButterfly-T and scButterfly-T-with-batch-correction. Although the RNA to ATAC translated profiles of scButterfly-T still contained some batch effects (Column #2 in Fig. R3c), the RNA to ATAC translated profiles of scButterfly-T-with-batch-correction were difficult to distinguish cell type HCT-116 and PDX2 (Column #4 in Fig. R3c), indicating that batch correction may lead to some biological heterogeneity loss, resulting in a decrease in

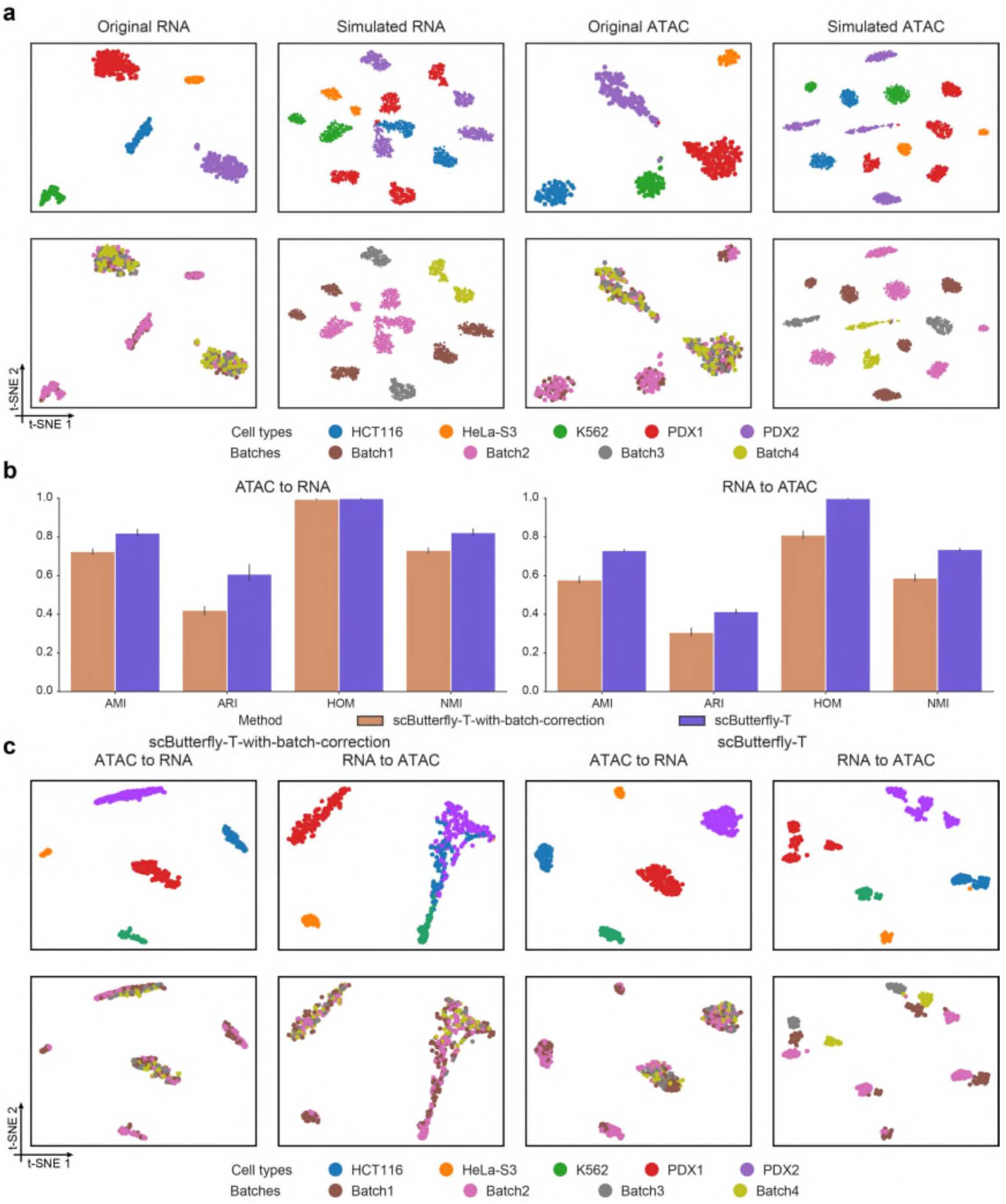

Fig. R3 | Translation performance of scButterfly-T and scButterfly-T-with-batch-correction on the simulated data with higher batch effects. a, t-SNE visualizations of cells in the CL dataset, using the original profiles and the profiles simulated by scDesign3 and simCAS, respectively for RNA and ATAC, colored by the cell types and batches. **b**, Evaluation of the cross-modality translation performance in five-fold cross-validation on the simulated CL dataset, via cell clustering with metrics of AMI, ARI, HOM and NMI. The height of each bar denotes the median value of each metric and the error bars show 95% confidence interval. **c**, t-SNE visualizations of cells in the simulated CL dataset, using the profiles translated by scButterfly-T and scButterfly-T-with-batch-correction, respectively for RNA and ATAC, colored by cell types and batches.

translation performance. These results demonstrate that even for the data with large batch effects, the existing batch correction methods may still not be suitable for use in the cross-modality translation of scButterfly.

In conclusion, we observed that even in data with higher batch effects, adding a batch correction step before data augmentation may still not be suitable for cross-modality translation with scButterfly. We analyzed the possible reasons for the performance decline associated with incorporating the batch correction step. We once again thank the reviewer for the constructive comments and have added detailed discussions about batch correction in Supplementary Text 1.

REMARKS ON CODE AVAILABILITY

The authors well provided the instructions to install and run the model.

RESPONSE

We deeply appreciate the reviewer for the acknowledgment of our code and tutorials.

REVIEWER #3

Our questions have been answered. We don't have further comments.

RESPONSE

We are extremely thankful to the reviewer for approval of the revised manuscript. The constructive comments provided by the reviewer have helped us significantly enhance our manuscript.

REVIEWER #4

Thank you for addressing my comments. I believe that the revised results and the addition of new figures have resolved my concerns and made the manuscript ready for publication.

RESPONSE

We sincerely appreciate the reviewer's recognition of the revised manuscript, and we once again express our gratitude for the valuable comments provided by the reviewer, which helped us improve our manuscript.

REMARKS ON CODE AVAILABILITY

The code is very detailed and well-organized. The author also provided tutorials of the models used in the manuscript.

RESPONSE

We are highly grateful for the reviewer’s affirmation of our code and tutorials.

Reference

1. Ashuach, T. et al. MultiVI: deep generative model for the integration of multimodal data. *Nat. Methods* **20**, 1222-1231 (2023).
2. Traag, V.A., Waltman, L. & van Eck, N.J. From Louvain to Leiden: guaranteeing well-connected communities. *Sci. Rep.* **9** (2019).
3. Chen, S., Lake, B.B. & Zhang, K. High-throughput sequencing of the transcriptome and chromatin accessibility in the same cell. *Nat. Biotechnol.* **37**, 1452-1457 (2019).
4. Van Der Maaten, L. & Hinton, G. Visualizing data using t-SNE. *J. Mach. Learn. Res.* **9**, 2579-2625 (2008).
5. Stuart, T. et al. Comprehensive Integration of Single-Cell Data. *Cell* **177**, 1888-1902.e1821 (2019).
6. Liu, J. et al. Jointly defining cell types from multiple single-cell datasets using LIGER. *Nat. Protoc.* **15**, 3632-3662 (2020).
7. Xu, C. et al. Probabilistic harmonization and annotation of single - cell transcriptomics data with deep generative models. *Mol. Syst. Biol.* **17**, e9620 (2021).
8. Lopez, R., Regier, J., Cole, M.B., Jordan, M.I. & Yosef, N. Deep generative modeling for single-cell transcriptomics. *Nat. Methods* **15**, 1053-1058 (2018).
9. Korsunsky, I. et al. Fast, sensitive and accurate integration of single-cell data with Harmony. *Nat. Methods* **16**, 1289-1296 (2019).
10. Luecken, M.D. et al. Benchmarking atlas-level data integration in single-cell genomics. *Nat. Methods* **19**, 41-50 (2022).
11. Tran, H.T.N. et al. A benchmark of batch-effect correction methods for single-cell RNA sequencing data. *Genome Biol.* **21**, 12 (2020).
12. Fleming, S.J. et al. Unsupervised removal of systematic background noise from droplet-based single-cell experiments using CellBender. *Nat. Methods* **20**, 1323-1335 (2023).
13. Song, D. et al. scDesign3 generates realistic in silico data for multimodal single-cell and spatial omics. *Nat. Biotechnol.* **42**, 247-252 (2024).
14. Li, C., Chen, X., Chen, S., Jiang, R. & Zhang, X. simCAS: an embedding-based method for simulating single-cell chromatin accessibility sequencing data. *Bioinformatics* **39**, btad453 (2023).
15. Lotfollahi, M., Wolf, F.A. & Theis, F.J. scGen predicts single-cell perturbation responses. *Nat. Methods* **16**, 715-721 (2019).
16. Behdenna, A. et al. pyComBat, a Python tool for batch effects correction in high-throughput molecular data using empirical Bayes methods. *BMC Bioinformatics* **24**, 459 (2023).

Reviewer #2 (Remarks to the Author):

The authors have addressed all of my concerns.

Reviewer #2 (Remarks on code availability):

The authors provided comprehensive codes to install and run the model but failed to explicitly indicate how to reproduce the results in the paper.
